# Cyclacene Stability: The Interplay of Strain, Aromaticity and Force Coupling

**DOI:** 10.1002/jcc.70325

**Published:** 2026-02-13

**Authors:** Ankit Somani, Divanshu Gupta, Jörg Grunenberg, Holger F. Bettinger

**Affiliations:** ^1^ Institut für Organische Chemie, Eberhard Karls Universität Tübingen Tübingen Germany; ^2^ Institut für Organische Chemie, Technische Universität Braunschweig Braunschweig Germany

## Abstract

The energy of cyclacenes arises from a subtle interplay between structural strain and aromatic stabilization. To disentangle these effects, we employ strain‐corrected heats of hydrogenation as a direct thermodynamic probe, supported by thermally‐assisted‐occupation density functional theory that is capable of capturing strong static correlation. Previous analyses of magnetic properties demonstrated a pronounced even–odd pattern: cyclacenes with an even number of fused rings fulfill magnetic criteria of aromaticity, whereas odd‐membered analogs do not according to diamagnetic susceptibility exaltation, nucleus‐independent chemical shifts, and anisotropy of induced current density (ACID). Our results demonstrate, however, that this predicted aromaticity does not translate into discernible thermodynamic stabilization. Instead, cyclacene stability is dictated primarily by strain energy, with aromatic contributions playing only a negligible role. These findings resolve a long‐standing question regarding the impact of aromaticity on cyclacene stability and clarify the fundamental factors that govern their reactivity and electronic behavior.

## Graphical abstract

Cyclacene stability arises from a balance between structural strain and aromaticity. Strain‐corrected heats of hydrogenation reveal that cyclacene thermodynamic stability is governed primarily by ring strain rather than aromatic stabilization. Pronounced even–odd aromaticity effects predicted by magnetic criteria do not result in significant differences in thermodynamic stability for cyclacenes.

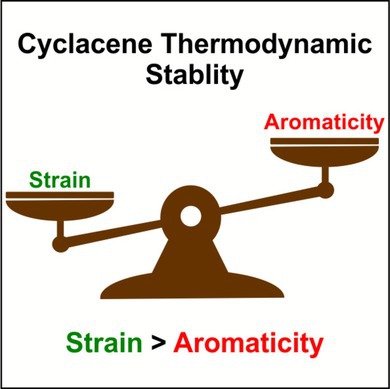

## 1 Introduction

**FIGURE 1:**
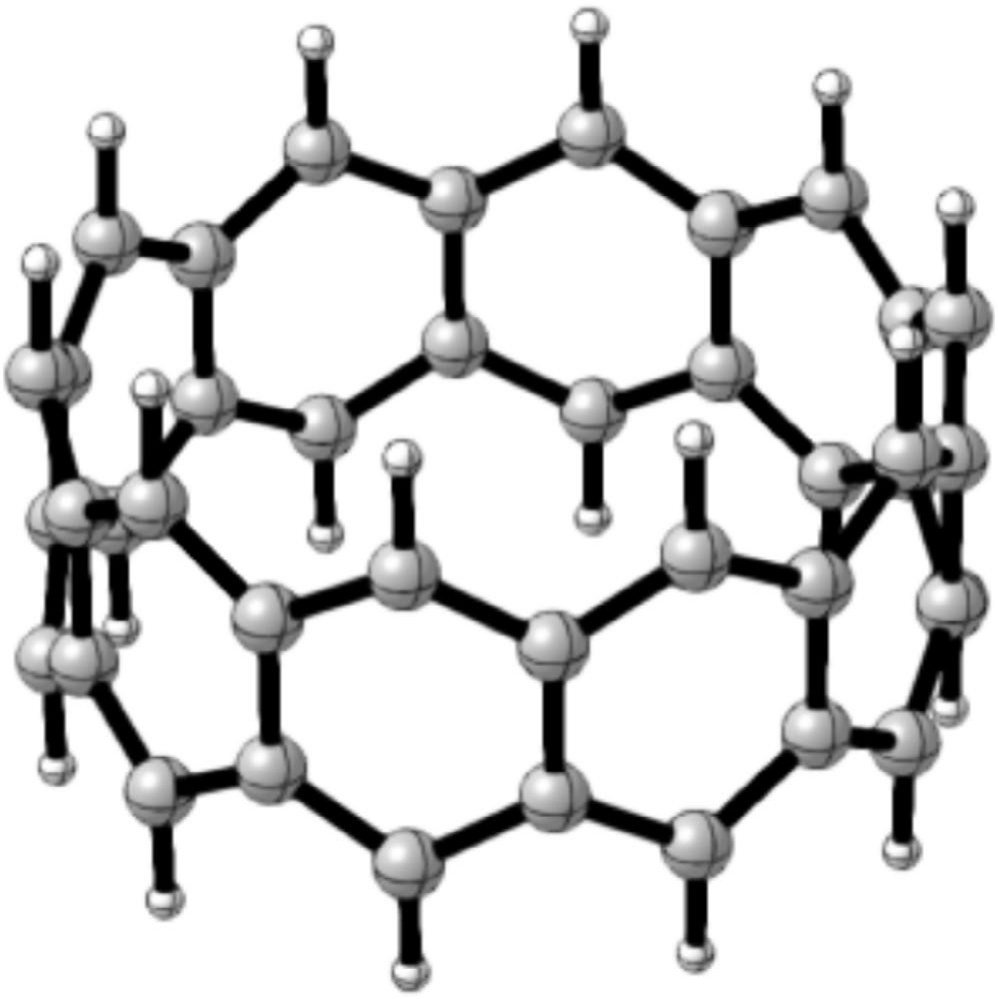
Structure of [10]‐cyclacene.

Among the diverse landscape of π‐conjugated nanocarbons, cyclacenes (C_4*n*_H_2*n*_) represent a structurally and electronically distinct subclass [1]. Originally proposed by Heilbronner in 1954, these hoop‐shaped hydrocarbon nanobelts consist of linearly fused benzene rings joined in a cyclic, belt‐like framework, formally related to acenes yet structurally reminiscent of carbon nanotubes (Figure 1) [2, 3, 4]. Their continuous π‐conjugated perimeter and unique topology have been predicted to impart distinctive electronic and magnetic properties, positioning cyclacenes as intriguing targets for molecular electronics and quantum materials [5, 6, 7, 8, 9, 10, 11].

The inherent combination of severe ring strain and electronic instability renders [*n*]‐cyclacenes (*n* is the number of fused six‐membered rings) exceptionally difficult synthetic targets. Their predicted diradical or polyradical character, together with the absence of Clar sextets, contributes to pronounced chemical reactivity that has repeatedly thwarted synthetic efforts [3, 4, 8, 12, 13, 14, 15, 16, 17, 18, 19, 20, 21, 22, 23, 24, 25, 26, 27, 28, 29]. Cyclacenes can be structurally described as cyclic arrays of *ortho*‐quinodimethane (o‐QDM) units [30, 31, 32]. The o‐QDM–like framework appears in highly reactive π‐conjugated architectures, including indenofluorenes such as indeno[1,2‐*a*]fluorene and indeno[2,1‐*a*]fluorene, highlighting a broader structural paradigm among destabilized nanocarbons [33, 34, 35, 36, 37, 38, 39, 40, 41].

Despite decades of attempts, the direct synthesis of [*n*]‐cyclacenes has remained elusive [3, 4, 17, 18]. Nevertheless, considerable progress has been achieved through the synthesis of precursor molecules and structurally related nanobelts. Key contributions along this trajectory include Stoddart's approach toward [12]‐cyclacene [17, 19, 20, 21, 22], Cory's preparative work on [8]‐cyclacene [23, 24], and Schlüter's investigations into [18]‐cyclacene [25, 26]. More recently, Itami and co‐workers reported the synthesis of carbon nanobelts with ring sizes of [12]‐, [16]‐, and [24]‐, which, although not cyclacenes, closely mimic their cyclic topology and provide valuable insights into their structural and electronic characteristics [27, 28]. Furthermore, Wang and colleagues reported evidence for transient [8]‐cyclacene generation via a retro‐Diels–Alder reaction under mass spectrometric conditions [29].

Owing to these persistent synthetic challenges, cyclacenes have been the focus of extensive theoretical study. Such investigations have been crucial for elucidating their structural, electronic, and aromatic properties, revealing pronounced size‐dependent behaviors governed by both strain and electronic effects [6, 8, 10, 11, 42, 43]. Computational studies show that the strain energy of cyclacenes decreases with increasing ring size, rendering smaller members relatively more unstable [11, 42, 44, 45, 46, 47]. Consequently, dimerization, one of their anticipated principal decomposition pathways, remains highly exothermic overall, though it is less favorable in larger systems [48]. Cyclacenes also exhibit oscillatory trends in properties such as heats of formation, singlet–triplet energy gaps, and dimerization energies for smaller cyclacenes, which have been rationalized in terms of the cryptoannulenic effect arising from their fused peripheral circuits [49, 50, 51, 52]. Even‐membered cyclacenes generally display greater aromaticity based on magnetic criteria than odd‐membered analogs, as demonstrated by magnetic response criteria such as nucleus‐independent chemical shift (NICS) values and anisotropy of the induced current density (ACID) analyses [5, 6, 43]. Despite extensive theoretical predictions of aromaticity, its actual impact on cyclacene stability and reactivity remains uncertain. In particular, computational studies of dimerization have not yielded convincing evidence of enhanced kinetic stability [53], leaving the practical significance of aromaticity in these systems an open question.

In the present study, we employ strain‐corrected heats of hydrogenation as a quantitative probe of cyclacene stability. By disentangling the energetic contributions of strain relief from those of π‐delocalization, this approach enables a direct evaluation of whether the inherent aromatic character inferred from NICS and ACID analyses translates into measurable thermodynamic stabilization. To accurately capture the pronounced static correlation and polyradical character that arise with increasing cyclacene size [7, 8, 11, 54, 55], we apply unrestricted hybrid Kohn–Sham density functional theory (KS‐DFT) [56] alongside thermally‐assisted‐occupation density functional theory (TAO‐DFT) that can successfully address systems with strong static correlation [8, 9, 13, 57, 58]. Our findings provide new insight into the delicate balance between electronic delocalization and structural strain in these elusive nanocarbon frameworks.

## 2 Methods

All structures were fully optimized in their singlet configuration using spin‐unrestricted density functional theory [59, 60] (DFT) with the B3LYP [61, 62] hybrid exchange–correlation functional, including Grimme's [63] D3 London dispersion correction with Becke–Johnson damping [UB3LYP‐D3(BJ)] [64]. The 6‐31G(d) basis set was employed for all computations reported in this work (Tables S7 and S8) [65]. Gibbs free energies were evaluated at *T* = 298.15, and *p* = 1 atm (Table S10). The expectation values of the Ŝ^2^ operator are given in the SI (Table S5). Harmonic vibrational frequencies were computed analytically to confirm that all stationary points correspond to minima. The UB3LYP‐D3(BJ) geometries were subsequently used for single‐point energy evaluations at the TAO‐B3LYP [58] level with D3 dispersion correction. For TAO‐B3LYP, a numerical grid consisting of 75 radial points in the Euler–Maclaurin quadrature and 302 angular points in the Lebedev grid was employed. In addition, all [*n*]‐cyclacene geometries (6 ≤ *n* ≤ 20) were optimized at the UCAM‐B3LYP‐D3(BJ)/6‐31G(d) and UωB97X‐D/6‐31G(d) levels of theory (Table S9) [66, 67, 68]. The resulting structures were compared with those obtained at the UB3LYP‐D3(BJ)/6‐31G(d) level to assess the influence of long‐range corrected functionals on the optimized geometries (Figure S5, Table S6) [69, 70, 71]. All UB3LYP computations were performed with Gaussian 16 [72], whereas TAO‐B3LYP calculations were carried out with Q‐Chem 6.2 [73] using its default parameters. In order to predict the coupling terms with their delicate interplay between electron correlation and bond strengths of adjacent bonds, we applied the (local, unrestricted) meta nonseparable gradient approximation (meta‐NGA) implemented as the UMN15‐L [74] functional in Gaussian 16 with the def2‐SVP basis set [75], which recently showed promising results for this kind of property [76]. The Cartesian force constants calculated at UMN15‐L/def2‐SVP using Gaussian 16 were employed as input for the COMPLIANCE [77, 78] (3.0.2 version) code in order to produce the relaxed force constants and their couplings.

## 3 Results and Discussion

### 3.1 Heats of Hydrogenation

**SCHEME 1:**
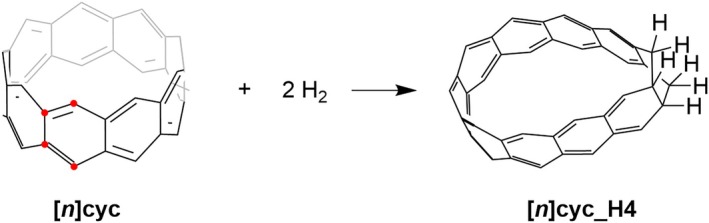
Heats of hydrogenation of the [*n*]‐cyclacene. Red dotted carbon atoms of the cyclacene (left) are being hydrogenated.

The heats of hydrogenation serve as a reliable indicator of the intrinsic thermodynamic stability and π‐electron delocalization in conjugated macrocyclic systems. By quantifying the energy released upon hydrogen addition, they provide insight into the extent of structural strain, the aromatic or nonaromatic character, and overall electronic stabilization within the molecular framework. Since cyclacenes do not possess a Clar sextet, two equivalents of hydrogen were introduced to avoid their formation in the hydrogenation product. The four hydrogen atoms were added at the two symmetry‐related sets of carbon atoms on opposite sides of the macrocycle—each set comprising an adjacent bridged (outer‐rim) carbon and a rung (inner‐rim) carbon of the trannulene subunit (Scheme 1).

To determine the heats of hydrogenation, we considered cyclacenes with 6 to 20 rings. The heats of hydrogenation were obtained according to Equation (1), where [*n*]cyc and [*n*]cycH_4_ are the cyclacene and its corresponding hydrogenated form.
(1)

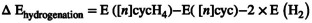




**FIGURE 2:**
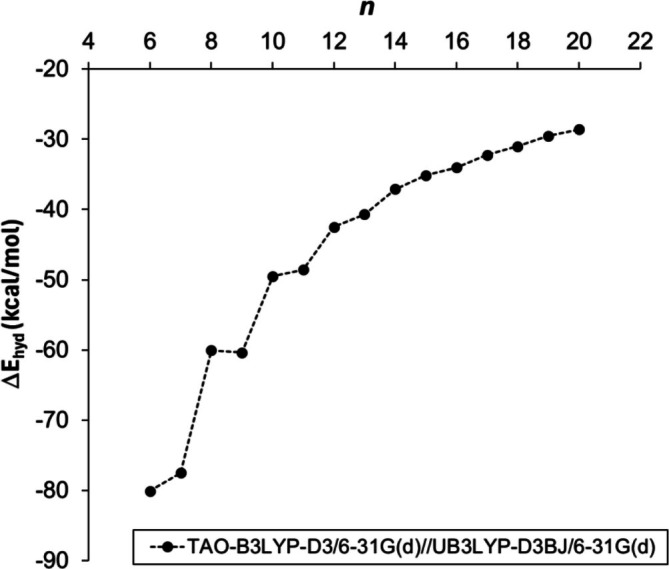
Heats of hydrogenation of [*n*]‐cyclacenes calculated at the TAO‐B3LYP‐D3/6‐31G(d)//UB3LYP‐D3(BJ)/6‐31G(d) level of theory as a function of the number *n* of fused hexagonal rings.

The heats of hydrogenation for all [*n*]‐cyclacenes (6 ≤ *n* ≤ 20), calculated at the TAO‐B3LYP‐D3/6‐31G(d) level of theory using geometries optimized at UB3LYP‐D3(BJ)/6‐31G(d), are negative, confirming that the hydrogenation process is exothermic. Overall, the heats of hydrogenation become progressively less exothermic with increasing *n*; however, for smaller cyclacenes (*n* < 11), an alternating (zigzag) variation is observed (Figures 2 and S1, Table S1), resembling the pattern observed for the dimerization energies [48]. In contrast to our previous study on dimerization energies at the UB3LYP‐D3(BJ)/6‐31G(d) level of theory, [6]‐ and [10]‐cyclacenes show more exothermic heats of hydrogenation than their neighboring [7]‐ and [11]‐cyclacenes, respectively, whereas [8]‐ and [9]‐cyclacenes exhibit nearly identical values (Figure 2). These findings suggest that, even though an oscillatory pattern is observed, the heats of hydrogenation do not indicate that even‐*n* [*n*]‐cyclacenes are particularly more stabilized than the similarly sized odd‐*n* [*n*]‐cyclacenes.

### 3.2 Strain Energy of Cyclacenes

The inherent strain in cyclacenes strongly influences their structural and electronic properties. The curved, macrocyclic architecture induces torsional and angular distortions in the carbon framework, which can perturb the π‐electron conjugation and, consequently, affect the energetic stability and electronic distribution within the macrocycle. An objective of our study is to separate the effects of structural strain from the energetic contributions reflected in the heats of hydrogenation.

**SCHEME 2:**
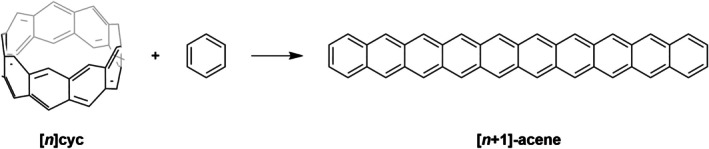
Homodesmotic reaction for [*n*]‐cyclacene.

**SCHEME 3:**
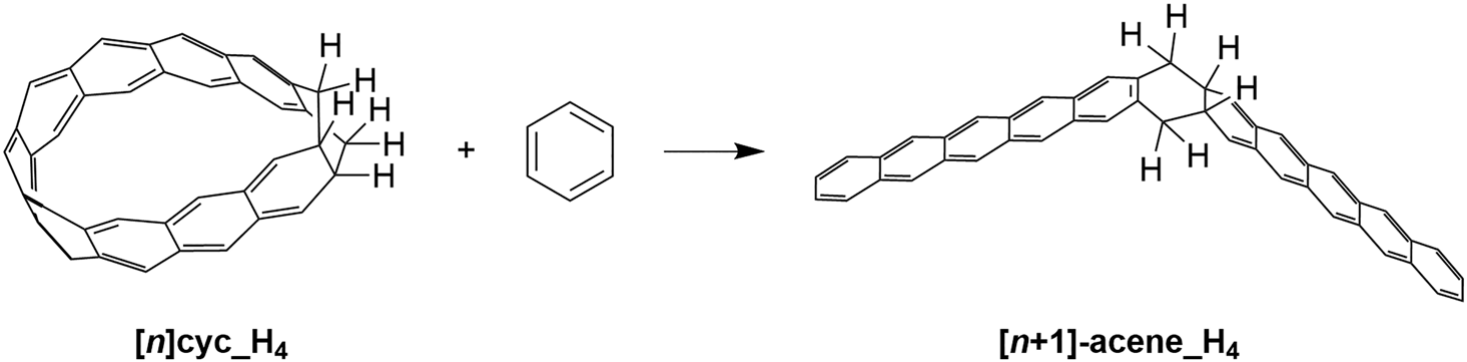
Homodesmotic reaction for tetrahydro‐[*n*]‐cyclacene.

The strain energies of cyclacenes have previously been evaluated using DFT‐based methods by Segawa et al. [45] and Sadowsky et al. [11], following the procedure introduced by Hopf and co‐workers for [*n*]circulenes [46]. In their approach, strain energy is estimated by extrapolating the normalized energy of the (C_4_H_2_) unit as a function of *n* ^−2^ to determine the energy of a hypothetical strain‐free fragment. An alternative method was proposed by Segawa et al. [42, 45] and utilized by Gupta et al. [44], in which strain energy is derived from the reaction enthalpy of a hypothetical homodesmotic reaction comparing the nanobelt to strain‐free acyclic subunits. It is noteworthy that the two approaches give quite similar strain energies [44]. A complementary approach calculates strain as the energy difference between the cyclacene and its linear acene counterpart of equivalent ring count [44]. In this work, we employed the method of Gupta et al. [44] to calculate the strain energies of cyclacenes (Scheme 2) and extended it to their hydrogenated products (Scheme 3).

**FIGURE 3:**
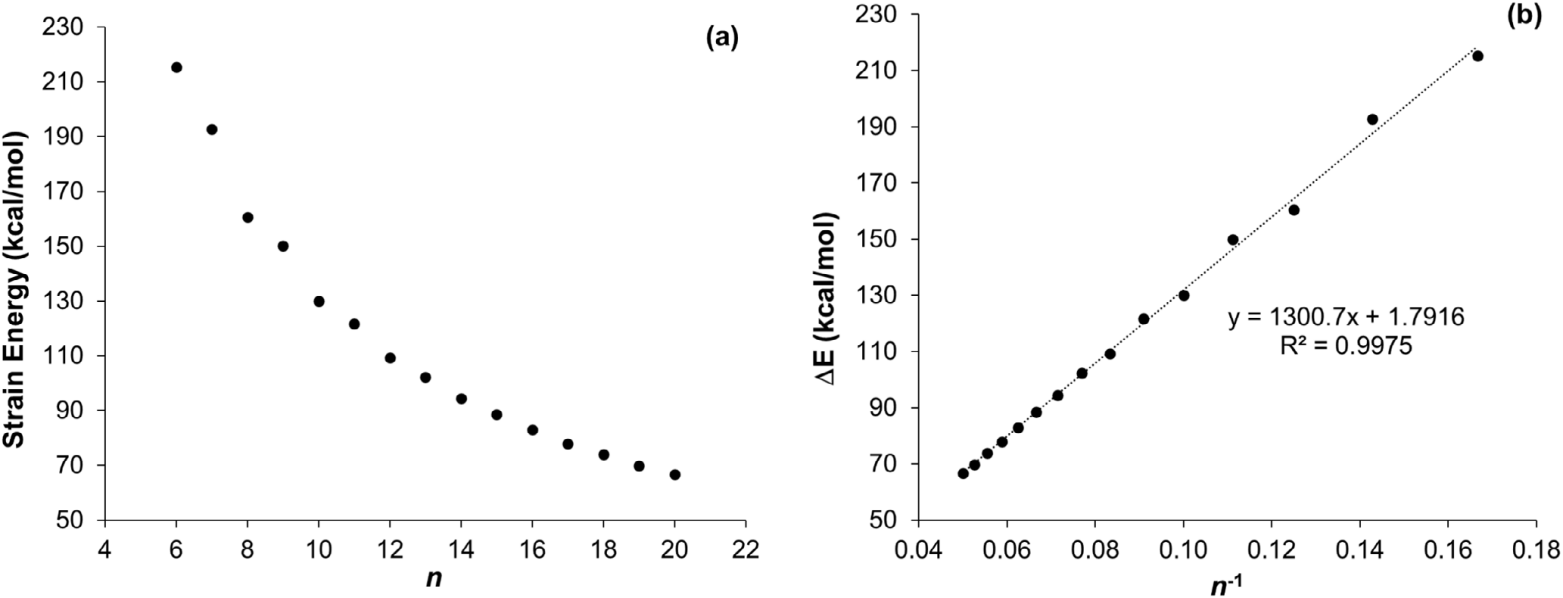
Strain energy of [*n*]‐cyclacenes calculated at the TAO‐B3LYP‐D3/6‐31G(d)//UB3LYP‐D3(BJ)/6‐31G(d) level of theory as a function of (a) *n* and (b) *n* ^−1^.

The strain energies, calculated at the TAO‐B3LYP‐D3/6‐31G(d)//UB3LYP‐D3(BJ)/6‐31G(d) level of theory from the reaction energies of the homodesmotic reactions (Scheme 2, Table S2), show a systematic decrease with increasing size of cyclacenes (Figures 3a and S2a). For smaller rings (*n* < 11), slight fluctuations in strain energy lead to an oscillatory trend. When plotted as a function of *n* ^−1^ (Figures 3b and S2b), the strain energy exhibits a linear correlation (*R* ^2^ = 0.9975), expressed by the equation 1300.7 × *n* ^−1^ + 1.7916 kcal mol^−1^ for [*n*]‐cyclacenes.

**FIGURE 4:**
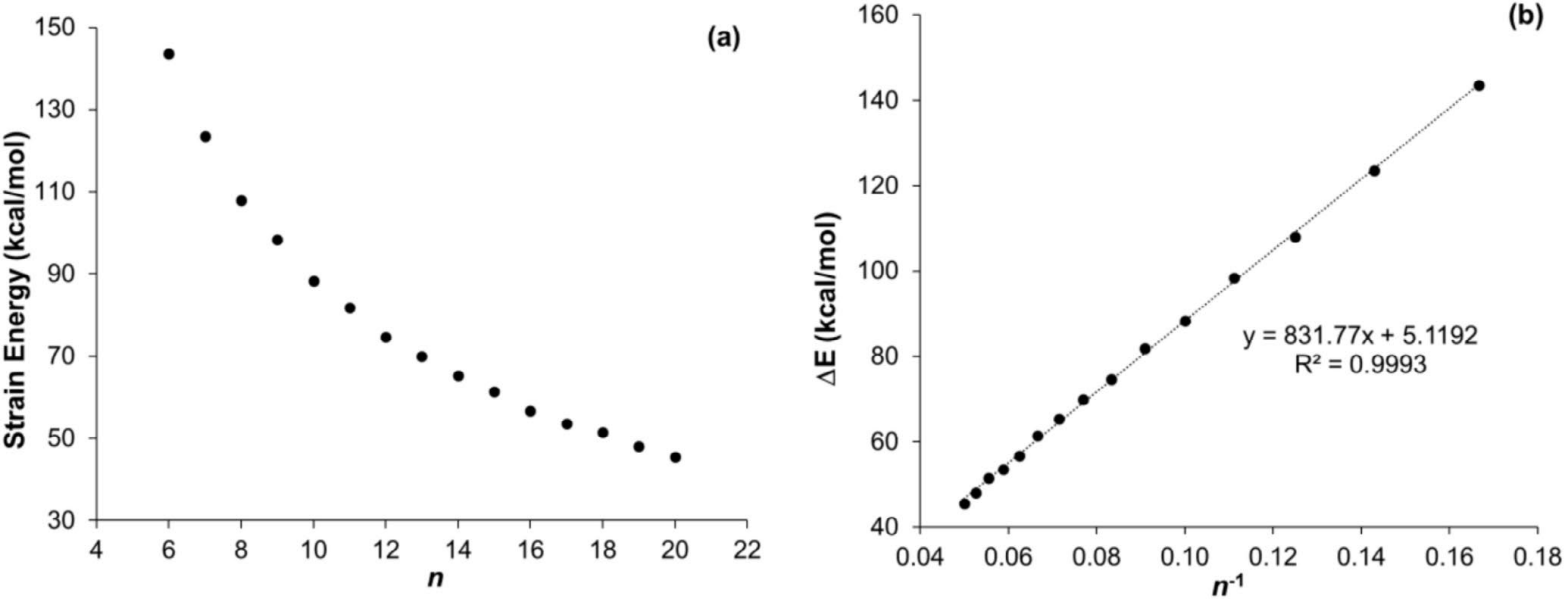
Strain energy of tetrahydro‐[*n*]‐cyclacenes calculated at the TAO‐B3LYP‐D3/6‐31G(d)//UB3LYP‐D3(BJ)/6‐31G(d) level of theory as a function of (a) *n* and (b) *n* ^−1^.

A similar strain analysis was conducted for the hydrogenated cyclacenes using the corresponding homodesmotic reaction (Scheme 3, Table S3), which also reveals a general decrease in strain energy with increasing ring size without any fluctuation in smaller cyclacenes (Figures 4a and S3a). When the strain energies are plotted as a function of *n* ^−1^ (Figures 4b and S3b), the data display a strong linear correlation (*R* ^2^ = 0.9993).

### 3.3 Strain‐Corrected Heats of Hydrogenation

As shown in Equation (1), the heats of hydrogenation are determined using the energy values of cyclacene, its corresponding hydrogenated form, and the hydrogen molecule. The total energy of cyclacene is primarily influenced by two opposing factors: destabilization arising from ring strain and stabilization due to cyclic π‐electron delocalization, which imparts aromatic character. Therefore, Equation (1) can be reformulated as Equations (2) and (3):
(2)

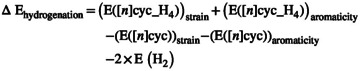



(3)

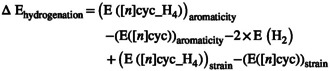




Since the strain contribution can be evaluated using a homodesmotic reaction, the residual molecular energy can be attributed to aromatic stabilization. We anticipate that this aromatic contribution will be the dominant component of the remaining energy. To determine the strain‐corrected heats of hydrogenation, the strain component in Equation (3) has to be compensated, which results in the following Equation (4):
(4)

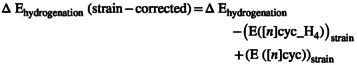




**FIGURE 5:**
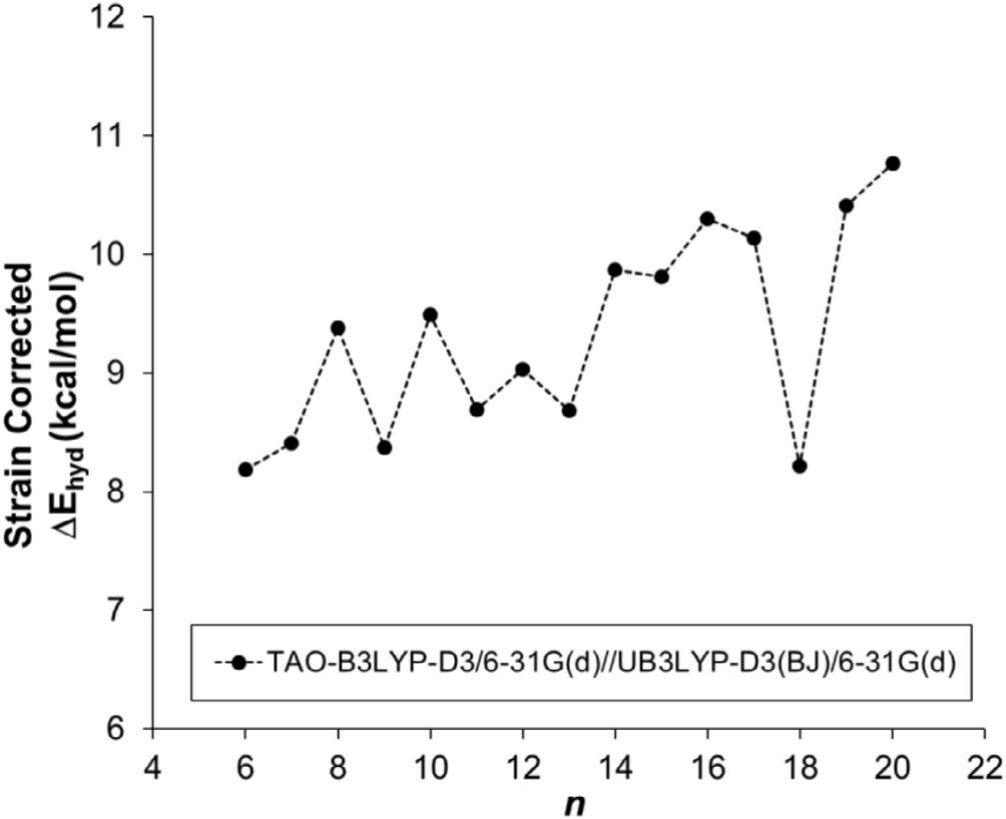
Strain‐corrected heats of hydrogenation of cyclacenes calculated at the TAO‐B3LYP‐D3/6‐31G(d)//UB3LYP‐D3(BJ)/6‐31G(d) level of theory as a function of the number of fused hexagonal rings.

The strain‐corrected heats of hydrogenation are found to be slightly endothermic compared to the highly exothermic uncorrected values, indicating that strain is the dominant factor governing the energy of cyclacenes (Figures 5 and S4, Table S4). A system that is stabilized by aromaticity is expected to have a smaller heat of strain corrected hydrogenation than one that is not stabilized or even destabilized.

The TAO‐B3LYP‐D3 data display only very small oscillations; however, the overall endothermicity of the strain‐corrected heats of hydrogenation remains nearly constant across all cyclacenes, ranging between 8 and 11 kcal/mol. These findings indicate that, although the cryptoannulenic effect induces an oscillatory pattern with even‐*n* [*n*]‐cyclacenes displaying slightly more favorable energies, its overall energetic contribution to cyclacene stability is essentially negligible. Moreover, the very similar strain‐corrected heats of hydrogenation show that the aromaticity indicated by magnetic criteria for even‐*n* cyclacenes does not translate into appreciable thermodynamic stabilization. These findings align with the recent reports by Nyvel et al. on the aromaticity of [*N*]annulenes [79].

### 3.4 Rigidity and Coupling Force Constants

In order to analyze the effect of ring strain and aromaticity on the individual C–C bonds and the electronic coupling between them, we additionally computed (a) all possible (relaxed) force constants between bonded *and* nonbonded carbon atoms, as well as (b) all individual coupling force constants applying the COMPLIANCE approach. We would like to point out that our computed force and coupling constants do not describe the thermodynamic stability of cyclacenes, but their rigidity.

**FIGURE 6:**
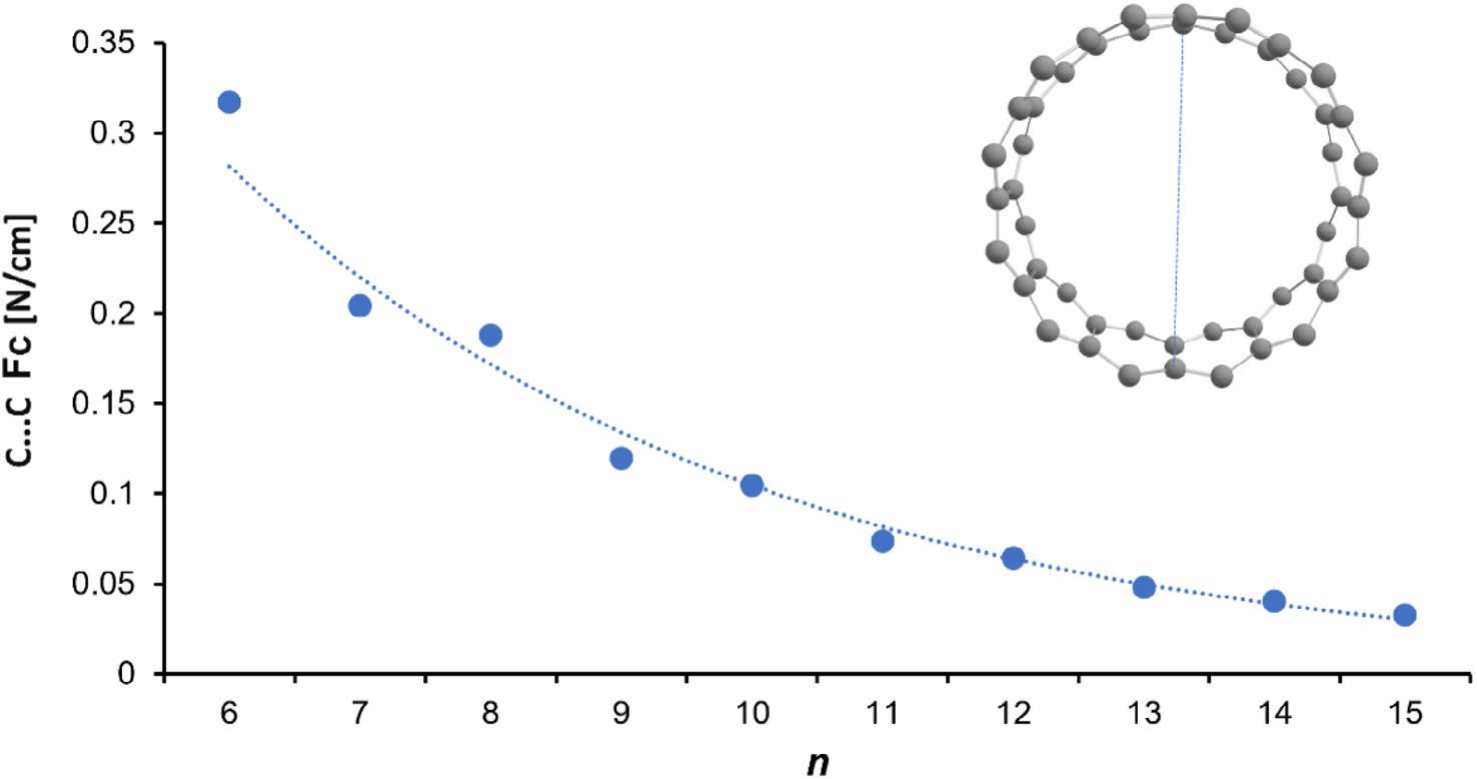
Relaxed force constants Fc between nonbonded opposed carbon atoms in cyclacenes calculated at the unrestricted MN15‐L/def2‐SVP level of theory as a function of the number *n* of fused benzene rings.

First of all, we looked at the force constants between opposed, nonbonded, carbon atoms (see Figure 6). Again, we see an smooth decline of the ring rigidity in combination with a weak oscillating pattern, more or less disappearing with [14]‐cyclacene.

There seems to be a general lower bound between 0.04 and 0.05 N/cm for ring sizes above 15 fused hexagons. If not even forceless, the ring deformation of the larger cyclacenes seems to be without much resistance, giving rise to an intense Raman active deformational band in the lower THz regime between 0.1 and 0.2 THz in addition to the radial breathing mode (RBM).

**FIGURE 7:**
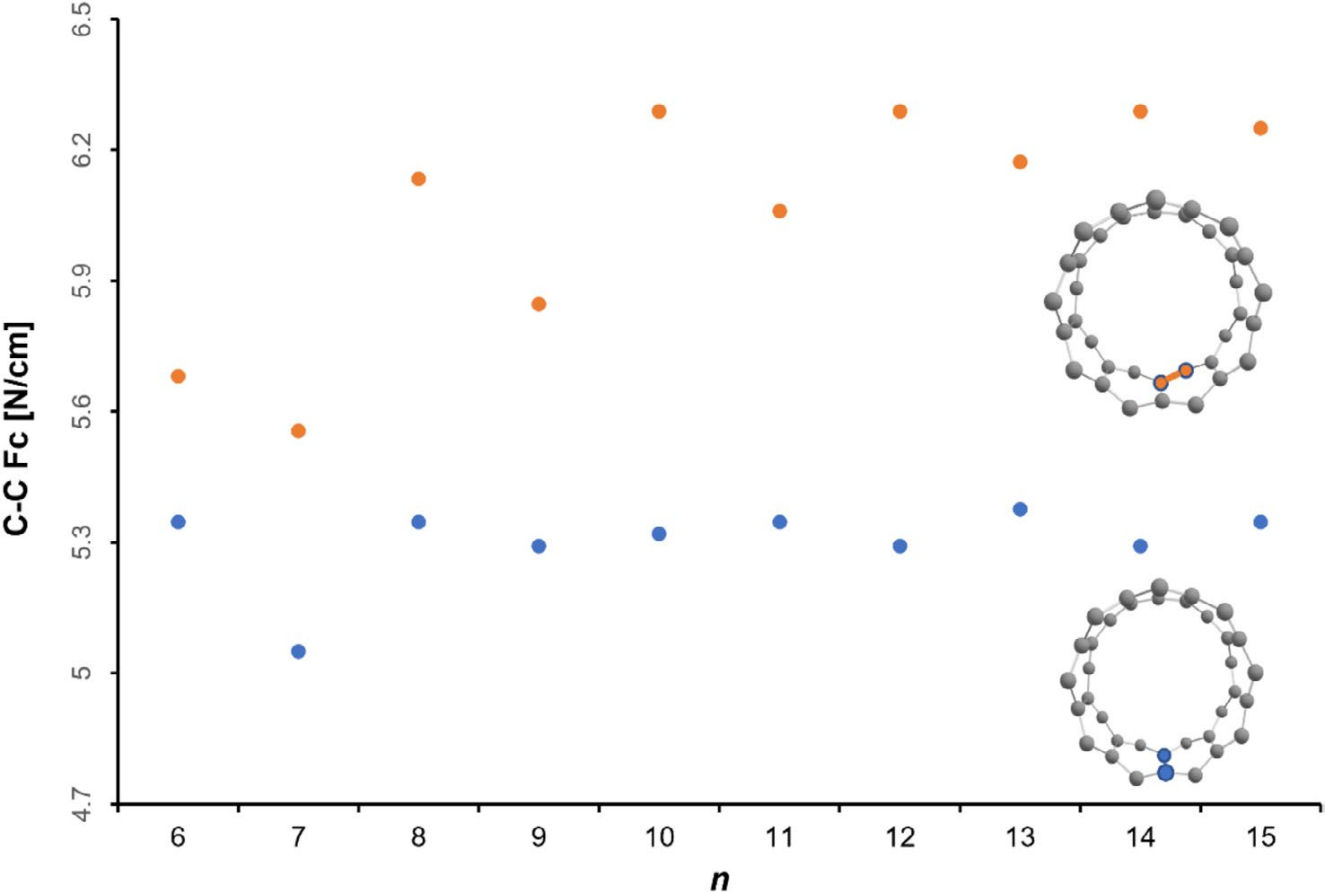
Relaxed force constants Fc between all zig‐zag edge bonds (orange) and rung bonds (blue) in cyclacenes calculated at the unrestricted MN15‐L/def2‐SVP level of theory as a function of the number *n* of fused benzene rings.

In a second step, we calculated the individual strengths for the edge as well as the rung C–C bonds. As expected, the bond strengths of all zig‐zag edge bonds (orange; Figure 7) seems to be stronger with a computed relaxed force constant of around 6.3 N/cm (6.6 N/cm for anthracene) in comparison with a low 5.3 N/cm (5.6 N/cm for anthracene) for the softer rung bonds (blue points; Figure 7).

Interestingly, the oscillation between even and odd numbered ring systems is pronounced only in the case of the zig‐zag edge bonds. It disappears for systems larger than the [14]‐cyclacene, while the rung bond rigidity is more or less independent of the ring size (with an exception of the [7]‐cyclacene). The pronounced ring strain for the smaller cyclacenes seems to be decoupled between the zig‐zag and rung bonds.

**FIGURE 8:**
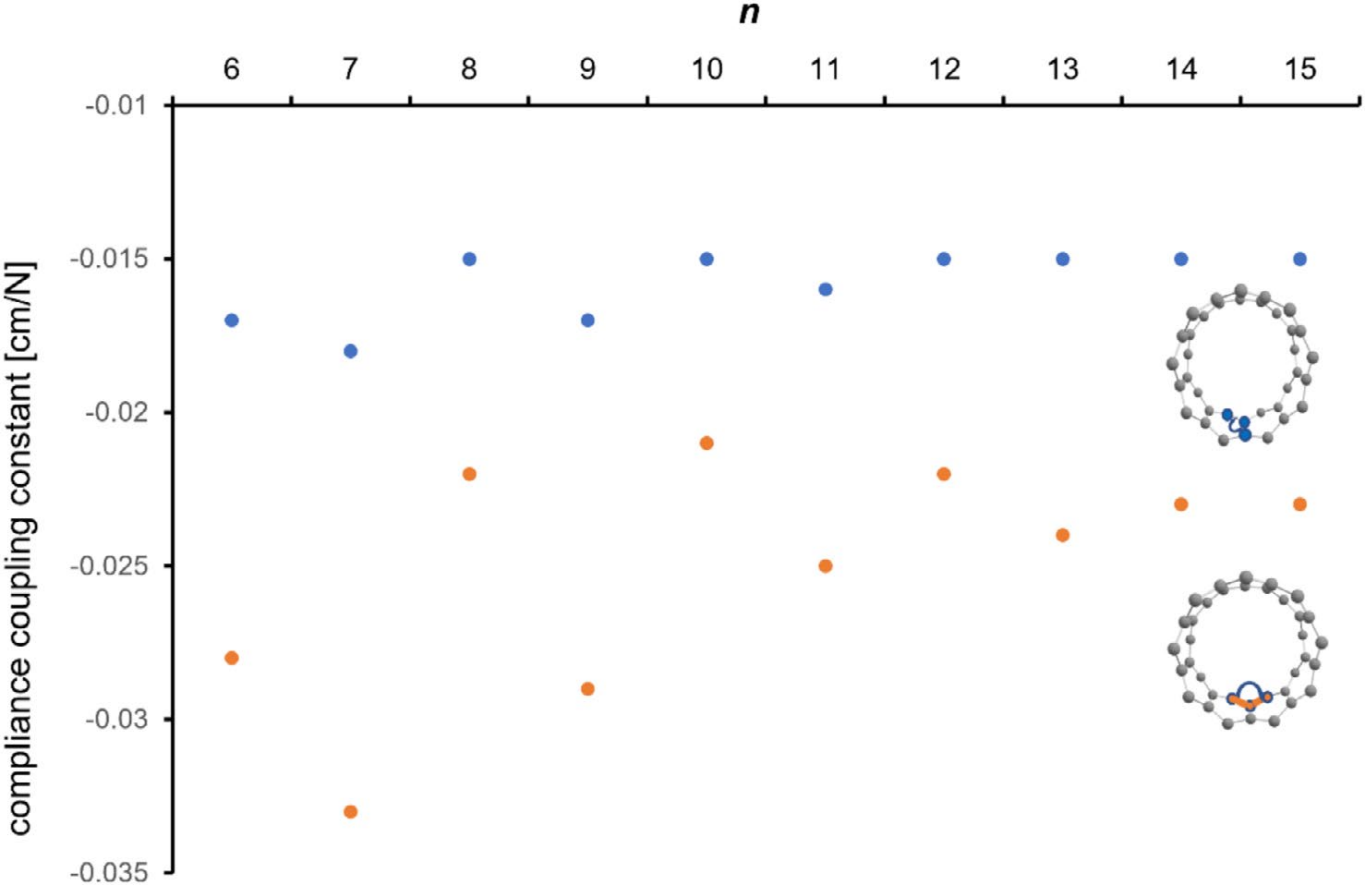
Coupling force constants (compliance constants) between all zig‐zag edge bonds (orange) and rung bonds (blue) in cyclacenes calculated at the unrestricted MN15‐L/def2‐SVP level of theory as a function of the number *n* of fused benzene rings.

In order to quantify the energetic zig‐zag/rung coupling, we further analyzed the force couplings between them. While traditional methods applied in order to predict force couplings between individual internal coordinates are generally error‐prone and semi‐empiric by nature due to numerous essential assumptions, the COMPLIANCE approach tries to compute the entire force field at the outset. The resulting coupling constants can therefore be used as unique and sensitive descriptors for electronic delocalization, in general. Looking at Figure 8, we see a weak coupling (blue points) between the rung and the zig‐zag perimeter approaching the anthracene or naphthalene value of −0.015 cm/N for cyclacenes larger than the [8]‐cyclacene. The oscillation is not very prominent (blue data points, Figure 8).

Turning to the electronic coupling between the zig‐zag bonds there seems to be a pronounced electronic coupling within the zig‐zag coordinates along the outer edge of cyclacenes, reaching a threshold of around −0.022 cm/N for larger systems, comparable with the aromatic coupling in benzene (orange data points, Figure 8). This time, the oscillating pattern is prominent, but again it disappears for systems with more than 14 fused rings.

## 4 Conclusion

Our computational analysis of the heats of hydrogenation confirms that the process is highly exothermic. Consistent with our previous findings on dimerization energies, the heats of hydrogenation exhibit a similar trend, with smaller cyclacenes (*n* < 11) displaying an oscillatory pattern followed by a monotonic increase for larger members. The overall energy of cyclacenes arises primarily from two competing factors: strain energy and aromatic stabilization. To eliminate the contribution of strain energy from the heats of hydrogenation, we employed a homodesmotic reaction approach. The resulting strain‐corrected heats of hydrogenation demonstrate that cyclacene stability is predominantly dictated by strain energy. Although an oscillatory trend persists, the overall values remain nearly constant (8–10 kcal/mol). Thus, the cryptoannulenic effect, while influencing the observed pattern, does not contribute significantly to thermodynamic stabilization.

According to our computed force constants between carbon atoms across the ring, the overall rigidity of cyclacenes does not differ substantially between the odd and even members of the series. The oscillating pattern disappears smoothly for larger cyclacenes with *n* > 10. Further, our calculations of all the couplings between individual force constants applying the COMPLIANCE approach showed that the electronic delocalisation in cyclacenes is (a) maximal between individual zig‐zag bonds along the edge and (b) weak between edge and the rung bonds, respectively.

## Funding

This work was supported by the H2020 European Research Council (101071420‐TACY‐ERC‐2022‐SYG) and the Deutsche Forschungsgemeinschaft (INST 40/575‐1 FUGG).

## Conflicts of Interest

The authors declare no conflicts of interest.

## Supporting information


**Figure S1:** Heats of hydrogenation of cyclacenes calculated at UB3LYP‐D3(BJ)/6‐31G(d) + ZPE level of theory as a function of the number of fused benzene rings.
**Figure S2:** Strain energy of cyclacenes calculated at the UB3LYP‐D3(BJ)/6‐31G(d) + ZPE level of theory as a function of (a) *n* and (b) *n* ^–1^.
**Figure S3:** Strain energy of tetrahydro‐cyclacenes calculated at the UB3LYP‐D3(BJ)/6‐31G(d) + ZPE level of theory as a function of (a) *n* and (b) *n* ^–1^.
**Figure S4:** Strain‐corrected heats of hydrogenation of cyclacenes calculated at UB3LYP‐D3(BJ)/6‐31G(d) + ZPE level of theory as a function of the number of fused benzene rings. Data point for *n* = 18 shows a deviation from the trend is highlighted with a blue oval in the figure.
**Figure S5:** Overlapped [10]‐cyclacene geometries optimized at various level of theory.
**Table S1:** Calculated zero‐point corrected energies (ZPE) of [*n*]‐cyclacene (for 6 ≤ *n* ≤ 20), their corresponding tetra‐hydro‐[*n*]‐cyclacene, and heats of hydrogenation (kcal/mol) at UB3LYP‐D3(BJ)/6‐31G(d) level of theory. The values in bold belong to UB3LYP, and the values in parentheses belong to the single‐point energies without ZPE at the TAO‐B3LYP‐D3/6‐31G(d)//UB3LYP‐D3(BJ)/6‐31G(d) level of theory.
**Table S2:** Calculated strain energy of [*n*]‐cyclacene (for 6 ≤ *n* ≤ 20) and (*n* + 1)_acene at B3LYP‐D3(BJ)/6‐31G(d) + ZPE level of theory (kcal/mol). The values in bold belong to UB3LYP, and the values in parentheses belong to the single‐point energies without ZPE at the TAO‐B3LYP‐D3/6‐31G(d)//UB3LYP‐D3(BJ)/6‐31G(d) level of theory.
**Table S3:** Calculated strain energy of tetrahydro‐[*n*]‐cyclacene (for 6 ≤ *n* ≤ 20) and (*n* + 1)_acene_H4 at B3LYP‐D3(BJ)/6‐31G(d) + ZPE level of theory (kcal/mol). The values in bold belong to UB3LYP, and the values in parentheses belong to the single‐point energies without ZPE at the TAO‐B3LYP‐D3/6‐31G(d)//UB3LYP‐D3(BJ)/6‐31G(d) level of theory.
**Table S4:** Calculated thermally corrected enthalpies of [*n*]‐cyclacenes (6 ≤ *n* ≤ 20), their corresponding tetrahydro‐[*n*]‐cyclacenes, and the resulting heats of hydrogenation (kcal mol⁻¹) at the UB3LYP‐D3(BJ)/6‐31G(d) level of theory.
**Table S5:** Ŝ^2^ operator values for [*n*]‐cyclacene, [*n*]‐cyclacene_H4, [*n* + 1]‐acene, [*n* + 1]‐acene_H_4_ and at UB3LYP‐D3(BJ)/6‐31G(d) level of theory.
**Table S6:** Bond lengths of [*n*]‐cyclacenes (Å) at UB3LYP‐D3(BJ)/6‐31G(d), UCAM‐B3LYP‐D3(BJ)/6‐31G(d), and UωB97X‐D/6‐31G(d) level of theory.
**Table S7:** Cartesian coordinates of the optimized geometries of [*n*]‐cyclacene and their corresponding tetra‐hydro‐[*n*]‐cyclacene at UB3LYP‐D3(BJ)/6‐31G(d) level of theory.
**Table S8:** Cartesian coordinates of the optimized geometries of [*n* + 1]‐acene and [*n* + 1]‐acene_H_4_ at UB3LYP‐D3(BJ)/6‐31G(d) level of theory.
**Table S9:** Cartesian coordinates of the optimized geometries of [*n*]‐cyclacene at UCAM‐B3LYP‐D3(BJ)/6‐31G(d) and UωB97X‐D/6‐31G(d) level of theory.
**Table S10:** Calculated Gibbs free energies (Δ*G*, 298.15 K) of [*n*]‐cyclacenes (6 ≤ *n* ≤ 20), their corresponding tetrahydro‐[*n*]‐cyclacenes, and the Gibbs free energy changes for their hydrogenation (ΔGhyd, kcal/mol), computed at the UB3LYP‐D3(BJ)/6‐31G(d) level of theory.

## Data Availability

The data that supports the findings of this study are available in the Supporting Information of this article.
