# Supplementary material for: Cyclacene Stability: The Interplay of Strain, Aromaticity and Force Coupling
Source: J Comput Chem. 2026 Feb 13;47(5):e70325. doi: 10.1002/jcc.70325 (PMC12903192; doi:10.1002/jcc.70325)
Supplement: Supplementary file 1 — Figure S1: Heats of hydrogenation of cyclacenes calculated at UB3LYP‐D3(BJ)/6‐31G(d) + ZPE level of theory as a function of the number of fused benzene rings. Figure S2: Strain energy of cyclacenes calculated at the UB3LYP‐D3(BJ)/6‐31G(d) + ZPE level of theory as a function of (a) n and (b) n –1. Figure S3: Strain energy of tetrahydro‐cyclacenes calculated at the UB3LYP‐D3(BJ)/6‐31G(d) + ZPE level of theory as a function of (a) n and (b) n –1. Figure S4: Strain‐corrected heats of hydrogenation of cyclacenes calculated at UB3LYP‐D3(BJ)/6‐31G(d) + ZPE level of theory as a function of the number of fused benzene rings. Data point for n = 18 shows a deviation from the trend is highlighted with a blue oval in the figure. Figure S5: Overlapped [10]‐cyclacene geometries optimized at various level of theory. Table S1: Calculated zero‐point corrected energies (ZPE) of [n]‐cyclacene (for 6 ≤ n ≤ 20), their corresponding tetra‐hydro‐[n]‐cyclacene, and heats of hydrogenation (kcal/mol) at UB3LYP‐D3(BJ)/6‐31G(d) level of theory. The values in bold belong to UB3LYP, and the values in parentheses belong to the single‐point energies without ZPE at the TAO‐B3LYP‐D3/6‐31G(d)//UB3LYP‐D3(BJ)/6‐31G(d) level of theory. Table S2: Calculated strain energy of [n]‐cyclacene (for 6 ≤ n ≤ 20) and (n + 1)_acene at B3LYP‐D3(BJ)/6‐31G(d) + ZPE level of theory (kcal/mol). The values in bold belong to UB3LYP, and the values in parentheses belong to the single‐point energies without ZPE at the TAO‐B3LYP‐D3/6‐31G(d)//UB3LYP‐D3(BJ)/6‐31G(d) level of theory. Table S3: Calculated strain energy of tetrahydro‐[n]‐cyclacene (for 6 ≤ n ≤ 20) and (n + 1)_acene_H4 at B3LYP‐D3(BJ)/6‐31G(d) + ZPE level of theory (kcal/mol). The values in bold belong to UB3LYP, and the values in parentheses belong to the single‐point energies without ZPE at the TAO‐B3LYP‐D3/6‐31G(d)//UB3LYP‐D3(BJ)/6‐31G(d) level of theory. Table S4: Calculated thermally corrected enthalpies of [n]‐cyclacenes (6 ≤ n ≤ 20), their corresponding tet [file JCC-47-0-s001.pdf]

Supporting Information

Cyclacene Stability: The Interplay of Strain and Aromaticity

Ankit Somani,[a] Divanshu Gupta,[a] Jörg Grunenberg,[b,*] Holger F. Bettinger[a,*]

[a] Institut für Organische Chemie, Eberhard Karls Universität Tübingen,
Auf der Morgenstelle 18, 72076 Tübingen, Germany

[b] Institut für Organische Chemie, Technische Universität Braunschweig,
Hagenring 30, 38106 Braunschweig, Germany

E-mail: holger.bettinger@uni-tuebingen.de

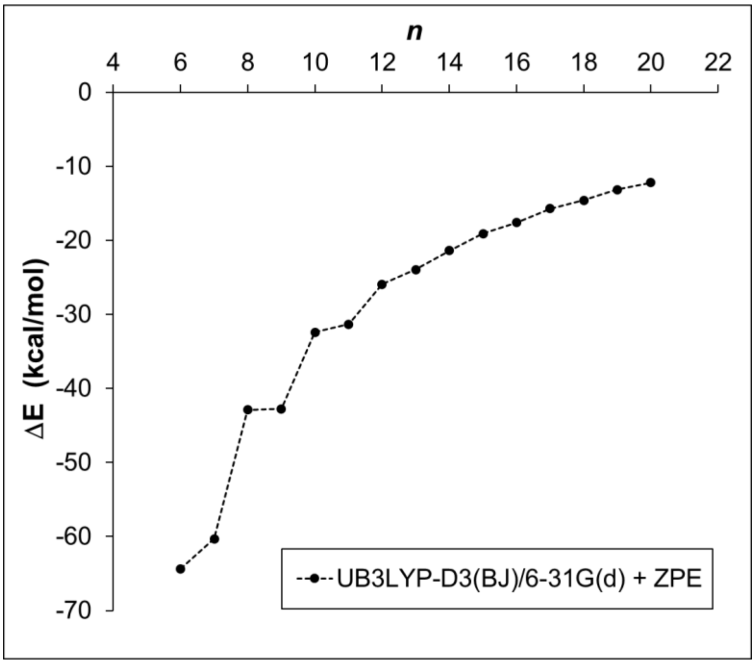

Figure S1. Heats of hydrogenation of cyclacenes calculated at UB3LYP-D3(BJ)/6-31G(d) + ZPE level of theory as a function of the number of fused benzene rings.

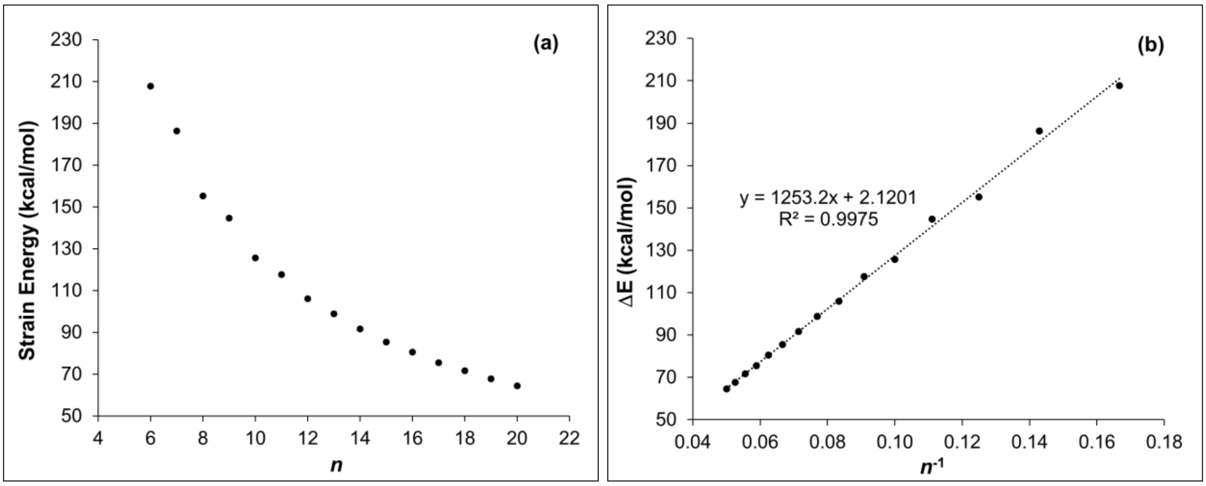

Figure S2. Strain energy of cyclacenes calculated at the UB3LYP-D3(BJ)/6-31G(d) + ZPE level of theory as a function of (a) *n* and (b) n^{-1}.

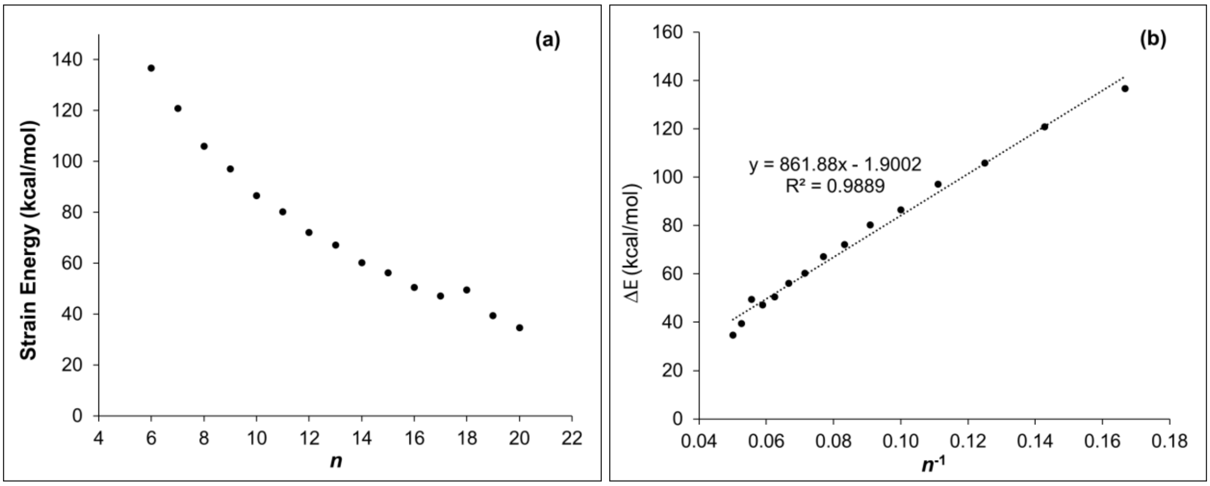

Figure S3. Strain energy of tetrahydro-cyclacenes calculated at the UB3LYP-D3(BJ)/6-31G(d) + ZPE level of theory as a function of (a) *n* and (b) n^{-1}.

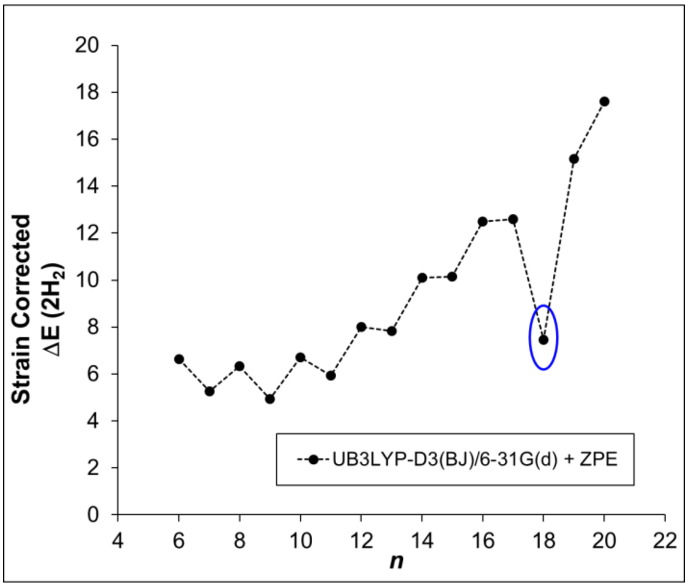

Figure S4. Strain-corrected heats of hydrogenation of cyclacenes calculated at UB3LYP-D3(BJ)/6-31G(d) + ZPE level of theory as a function of the number of fused benzene rings. Data point for $n = 18$ shows a deviation from the trend is highlighted with a blue oval in the figure.

Table S1. Calculated zero-point corrected energies (ZPE) of [*n*]-cyclacenes (for $6 \leq n \leq 20$), the corresponding tetra-hydro-[*n*]-cyclacene, and heats of hydrogenation (kcal/mol) at UB3LYP-D3(BJ)/6-31G(d) level of theory. The values in bold belong to UB3LYP, and the values in parentheses belong to the single-point energies without ZPE at the TAO-B3LYP-D3/6-31G(d)//UB3LYP-D3(BJ)/6-31G(d) level of theory.

n	**E+ZPE (Hartree)**	**E+ZPE (Hydrogenated) (Hartree)**	**ΔE_{hyd} (kcal/mol)**
6	**-921.3177** (-*921.4347*)	**-923.7512** (-*923.9045*)	**-64.3** (-*80.1*)
7	**-1074.9600** (-*1075.0999*)	**-1077.3871** (-*1077.5657*)	**-60.3** (-*77.5*)
8	**-1228.6182** (-*1228.7807*)	**-1231.0175** (-*1231.2186*)	**-42.9** (-*60.1*)
9	**-1382.2432** (-*1382.4269*)	**-1384.6423** (-*1384.8654*)	**-42.7** (-*60.4*)
10	**-1535.8819** (-*1536.0884*)	**-1538.2645** (-*1538.5095*)	**-32.4** (-*49.5*)
11	**-1689.5031** (-*1689.7309*)	**-1691.8839** (-*1692.1506*)	**-31.3** (-*48.6*)
12	**-1843.1296** (-*1843.3803*)	**-1845.5019** (-*1845.7902*)	**-25.9** (-*42.5*)
13	**-1996.7493** (-*1997.0208*)	**-1999.1184** (-*1999.4279*)	**-23.9** (-*40.7*)
14	**-2150.3689** (-*2150.6626*)	**-2152.7339** (-*2153.0640*)	**-21.4** (-*37.1*)
15	**-2303.9870** (-*2304.3016*)	**-2306.3484** (-*2306.6997*)	**-19.1** (-*35.1*)
16	**-2457.6031** (-*2457.9397*)	**-2459.9621** (-*2460.3362*)	**-17.6** (-*34.0*)
17	**-2611.2193** (-*2611.5771*)	**-2613.5753** (-*2613.9706*)	**-15.7** (-*32.2*)
18	**-2764.8337** (-*2765.2129*)	**-2767.1879** (-*2767.6045*)	**-14.6** (-*31.0*)
19	**-2918.4482** (-*2918.8487*)	**-2920.8001** (-*2921.2380*)	**-13.1** (-*29.5*)
20	**-3072.0615** (-*3072.4833*)	**-3074.4119** (-*3074.8711*)	**-12.2** (-*28.6*)
**H_2 energy (Hartree)** **-1.1655** (-*1.1711*)			

Table S2. Calculated strain energy of [*n*]-cyclacenes (for $6 \leq n \leq 20$) and (*n*+1)_acene at B3LYP-D3(BJ)/6-31G(d) + ZPE level of theory (kcal/mol). The values in bold belong to UB3LYP, and the values in parentheses belong to the single-point energies without ZPE at the TAO-B3LYP-D3/6-31G(d)//UB3LYP-D3(BJ)/6-31G(d) level of theory.

n	**E+ZPE (Hartree)**	**E+ZPE [(*n*+1)_acene] (Hartree)**	**Strain Energy (kcal/mol)**
6	**-921.3177**<br>(*-921.4347*)	**-1153.8154**<br>(*-1154.0011*)	**207.7**<br>(*215.3*)
7	**-1074.9600**<br>(*-1075.0999*)	**-1307.4238**<br>(*-1307.6304*)	**186.4**<br>(*192.8*)
8	**-1228.6182**<br>(*-1228.7807*)	**-1461.0322**<br>(*-1461.2598*)	**155.2**<br>(*160.6*)
9	**-1382.2432**<br>(*-1382.4269*)	**-1614.6406**<br>(*-1614.8893*)	**144.8**<br>(*150.1*)
10	**-1535.8819**<br>(*-1536.0884*)	**-1768.2489**<br>(*-1768.5188*)	**125.7**<br>(*130.0*)
11	**-1689.5031**<br>(*-1689.7309*)	**-1921.8571**<br>(*-1922.1481*)	**117.6**<br>(*121.7*)
12	**-1843.1296**<br>(*-1843.3803*)	**-2075.4653**<br>(*-2075.7776*)	**106.0**<br>(*109.3*)
13	**-1996.7493**<br>(*-1997.0208*)	**-2229.0735**<br>(*-2229.4070*)	**98.8**<br>(*102.3*)
14	**-2150.3689**<br>(*-2150.6626*)	**-2382.6817**<br>(*-2383.0364*)	**91.7**<br>(*94.5*)
15	**-2303.9870**<br>(*-2304.3016*)	**-2536.2899**<br>(*-2536.6658*)	**85.5**<br>(*88.5*)
16	**-2457.6031**<br>(*-2457.9397*)	**-2689.8981**<br>(*-2690.2952*)	**80.5**<br>(*83.0*)
17	**-2611.2193**<br>(*-2611.5771*)	**-2843.5063**<br>(*-2843.9245*)	**75.5**<br>(*77.9*)
18	**-2764.8337**<br>(*-2765.2129*)	**-2997.1145**<br>(*-2997.5540*)	**71.6**<br>(*74.0*)
19	**-2918.4482**<br>(*-2918.8487*)	**-3150.7228**<br>(*-3151.1833*)	**67.7**<br>(*69.9*)
20	**-3072.0615**<br>(*-3072.4833*)	**-3304.3310**<br>(*-3304.8128*)	**64.5**<br>(*66.7*)
**Benzene (Hartree)**<br>**-232.1667**<br>(*-232.2232*)			

Table S3. Calculated strain energy of tetrahydro-[*n*]-cyclacenes (for $6 \leq n \leq 20$) and (*n*+1)_acene_H_4 at B3LYP-D3(BJ)/6-31G(d) + ZPE level of theory (kcal/mol). The values in bold belong to UB3LYP, and the values in parentheses belong to the single-point energies without ZPE at the TAO-B3LYP-D3/6-31G(d)//UB3LYP-D3(BJ)/6-31G(d) level of theory.

n	**E+ZPE (Hartree)**	**E+ZPE [(*n*+1)_acene_H₄] (Hartree)**	**Strain Energy (kcal/mol)**
6	**-923.7512** (-*923.9045*)	**-1156.1358** (-*1156.3566*)	**136.7** (*143.6*)
7	**-1077.3871** (-*1077.5657*)	**-1309.7464** (-*1309.9857*)	**120.8** (*123.5*)
8	**-1231.0175** (-*1231.2186*)	**-1463.3531** (-*1463.6140*)	**106.0** (*108.0*)
9	**-1384.6423** (-*1384.8654*)	**-1616.9637** (-*1617.2453*)	**97.1** (*98.3*)
10	**-1538.2645** (-*1538.5095*)	**-1770.5692** (-*1770.8734*)	**86.6** (*88.3*)
11	**-1691.8839** (-*1692.1506*)	**-1924.1786** (-*1924.5042*)	**80.3** (*81.8*)
12	**-1845.5019** (-*1845.7902*)	**-2077.7835** (-*2078.1325*)	**72.1** (*74.7*)
13	**-1999.1184** (-*1999.4279*)	**-2231.392** (-*2231.7626*)	**67.1** (*69.9*)
14	**-2152.7339** (-*2153.0640*)	**-2384.9966** (-*2385.3913*)	**60.2** (*65.3*)
15	**-2306.3484** (-*2306.6997*)	**-2538.6047** (-*2539.0209*)	**56.2** (*61.4*)
16	**-2459.9621** (-*2460.3362*)	**-2692.2092** (-*2692.6497*)	**50.4** (*56.7*)
17	**-2613.5753** (-*2613.9706*)	**-2845.8172** (-*2846.2792*)	**47.2** (*53.6*)
18	**-2767.1879** (-*2767.6045*)	**-2999.4336** (-*2999.9099*)	**49.6** (*51.6*)
19	**-2920.8001** (-*2921.2380*)	**-3153.0296** (-*3153.5377*)	**39.4** (*48.0*)
20	**-3074.4119** (-*3074.8711*)	**-3306.6339** (-*3307.1668*)	**34.7** (*45.5*)
**Benzene (Hartree)** **-232.1667** (-*232.2232*)			

Table S4. Calculated thermally corrected enthalpies of [*n*]-cyclacenes ($6 \le n \le 20$), their corresponding tetrahydro-[*n*]-cyclacenes, and the resulting heats of hydrogenation (kcal mol^{-1}) at the UB3LYP-D3(BJ)/6-31G(d) level of theory.

n	**[*n*]-cyclacene (Hartree)**	**[*n*]-cyclacene_H$_4$ (Hartree)**	**ΔH_{hyd} (kcal/mol)**
6	-921.3027	-923.7355	-66.9
7	-1074.9425	-1077.3689	-62.8
8	-1228.5983	-1230.9967	-45.3
9	-1382.2204	-1384.6189	-45.3
10	-1535.8567	-1538.2383	-34.7
11	-1689.4750	-1691.8551	-33.8
12	-1843.0989	-1845.4703	-28.3
13	-1996.7158	-1999.0841	-26.4
14	-2150.3328	-2152.6968	-23.7
15	-2303.9480	-2306.3086	-21.6
16	-2457.5615	-2459.9196	-20.0
17	-2611.1749	-2613.5300	-18.1
18	-2764.7865	-2767.1398	-17.0
19	-2918.3983	-2920.7492	-15.5
20	-3072.0087	-3074.3582	-14.6
**H_2 energy (Hartree)**<br>-1.163128			

Table S5. $\hat{S}2$ operator values for [*n*]-cyclacene, [*n*]-cyclacene_H_4, [*n*+1]-acene, and [*n*+1]-acene_H_4 at UB3LYP-D3(BJ)/6-31G(d) level of theory.

**[*n*]**	**[*n*]-cyclacene**	**[*n*]-cyclacene_H_4**	**[*n*+1]-acene**	**[*n*+1]-acene_H_4**
6	2.0	2.2	0.6	0.3
7	4.9	2.5	1.4	0.9
8	2.9	3.1	2.3	0.9
9	7.0	4.1	3.2	1.4
10	5.1	5.1	4.2	1.4
11	8.2	6.2	5.2	2.0
12	7.7	7.2	6.3	2.0
13	9.4	8.1	7.2	2.7
14	9.8	4.5	8.1	2.7
15	10.7	4.6	9.0	3.7
16	11.5	10.4	9.8	3.7
17	12.0	11.2	10.5	4.8
18	12.9	11.9	11.3	10.4
19	13.4	12.6	12.0	6.0
20	14.2	13.3	12.7	6.0

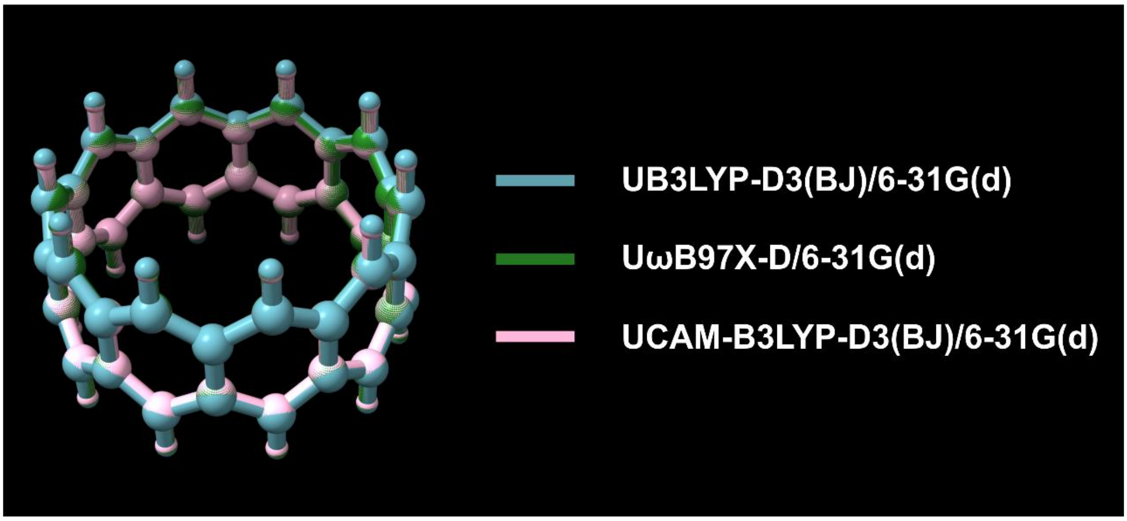

Figure S5. Overlapped [10]-cyclacene geometries optimized at various level of theory.

Table S6. Bond lengths of [*n*]-cyclacenes (Å) at UB3LYP-D3(BJ)/6-31G(d), UCAM-B3LYP-D3(BJ)/6-31G(d), and UωB97X-D/6-31G(d) level of theory.

n	**UB3LYP-D3(BJ)/6-31G(d)**			**UCAM-B3LYP-D3(BJ)/6-31G(d)**			**UωB97X-D/6-31G(d)**		
	Bridge	Rung	CH	Bridge	Rung	CH	Bridge	Rung	CH
6	1.419	1.451	1.087	1.416	1.444	1.087	1.417	1.445	1.088
7	1.416	1.457	1.087	1.413	1.447	1.087	1.415	1.448	1.088
8	1.412	1.457	1.087	1.409	1.449	1.087	1.411	1.450	1.088
9	1.412	1.455	1.088	1.409	1.448	1.087	1.411	1.449	1.088
10	1.409	1.459	1.088	1.407	1.450	1.087	1.408	1.451	1.088
11	1.410	1.456	1.087	1.407	1.449	1.087	1.408	1.450	1.088
12	1.408	1.459	1.088	1.406	1.450	1.087	1.407	1.451	1.088
13	1.408	1.457	1.087	1.405	1.450	1.087	1.407	1.451	1.088
14	1.407	1.459	1.088	1.405	1.450	1.087	1.407	1.451	1.088
15	1.407	1.458	1.088	1.405	1.450	1.087	1.406	1.451	1.088
16	1.407	1.459	1.088	1.404	1.450	1.087	1.406	1.451	1.088
17	1.407	1.459	1.088	1.404	1.451	1.087	1.406	1.451	1.088
18	1.407	1.459	1.088	1.404	1.451	1.087	1.406	1.452	1.088
19	1.406	1.459	1.088	1.404	1.451	1.087	1.405	1.452	1.088
20	1.406	1.459	1.088	1.404	1.451	1.087	1.405	1.452	1.088

Table S7. Cartesian coordinates of the optimized geometries of [*n*]-cyclacene and their corresponding tetra-hydro-[*n*]-cyclacene at UB3LYP-D3(BJ)/6-31G(d) level of theory.

UB3LYP-D3(BJ)/6-31G(d)				UB3LYP-D3(BJ)/6-31G(d)			
**[6]-cyclacene**				**[6]-cyclacene_H_4**			
C	0.00000000	2.39875200	0.72525800	C	2.00775300	-0.72264700	0.71767700
C	1.20702800	2.09063400	1.40518900	C	1.86639000	0.52244800	1.40395000
C	2.07738000	1.19937600	0.72525800	C	1.46461200	1.64532700	0.71412500
C	2.07738000	1.19937600	-0.72525800	C	1.46461200	1.64532700	-0.71412500
C	1.20702800	2.09063400	-1.40518900	C	1.86639000	0.52244800	-1.40395000
C	0.00000000	2.39875200	-0.72525800	C	2.00775300	-0.72264700	-0.71767700
C	2.41405600	0.00000000	1.40518900	C	0.69730000	2.77219900	1.35143300
C	2.41405600	0.00000000	-1.40518900	C	0.69730000	2.77219900	-1.35143300
C	2.07738000	-1.19937600	-0.72525800	C	-0.78308400	2.76808500	-0.78610800
C	2.07738000	-1.19937600	0.72525800	C	-0.78308400	2.76808500	0.78610800
C	1.20702800	-2.09063400	1.40518900	C	-1.51003400	1.60806100	1.41159600
H	1.18371600	-2.05025600	2.49165500	H	-1.53285800	1.59784300	2.50037800
C	0.00000000	-2.39875200	0.72525800	C	-1.85070500	0.46101300	0.73712000
C	0.00000000	-2.39875200	-0.72525800	C	-1.85070500	0.46101300	-0.73712000
C	1.20702800	-2.09063400	-1.40518900	C	-1.51003400	1.60806100	-1.41159600
H	2.36743200	0.00000000	2.49165500	H	0.66986500	2.67078300	2.44107100
H	1.18371600	2.05025600	2.49165500	H	1.79533000	0.50637200	2.48923700
H	1.18371600	2.05025600	-2.49165500	H	1.79533000	0.50637200	-2.48923700
H	2.36743200	0.00000000	-2.49165500	H	0.66986500	2.67078300	-2.44107100
H	1.18371600	-2.05025600	-2.49165500	H	-1.53285800	1.59784300	-2.50037800
C	-1.20702800	-2.09063400	1.40518900	C	-1.94851500	-0.79513600	1.41503600
C	-2.07738000	-1.19937600	0.72525800	C	-1.52159500	-1.93951800	0.73440300
C	-2.07738000	-1.19937600	-0.72525800	C	-1.52159500	-1.93951800	-0.73440300
C	-1.20702800	-2.09063400	-1.40518900	C	-1.94851500	-0.79513600	-1.41503600
C	-2.41405600	0.00000000	1.40518900	C	-0.62452200	-2.83831600	1.39263400
C	-2.41405600	0.00000000	-1.40518900	C	-0.62452200	-2.83831600	-1.39263400
C	-2.07738000	1.19937600	-0.72525800	C	0.67440200	-2.72071500	-0.71589900
C	-2.07738000	1.19937600	0.72525800	C	0.67440200	-2.72071500	0.71589900
C	-1.20702800	2.09063400	1.40518900	C	1.56500500	-1.88353500	1.40086700
H	-1.18371600	2.05025600	2.49165500	H	1.49595800	-1.83288500	2.48519400
C	-1.20702800	2.09063400	-1.40518900	C	1.56500500	-1.88353500	-1.40086700
H	-2.36743200	0.00000000	2.49165500	H	-0.63290400	-2.87104400	2.47982400
H	-2.36743200	0.00000000	-2.49165500	H	-0.63290400	-2.87104400	-2.47982400
H	-1.18371600	2.05025600	-2.49165500	H	1.49595800	-1.83288500	-2.48519400
H	-1.18371600	-2.05025600	2.49165500	H	-1.92428400	-0.78974700	2.50220100
H	-1.18371600	-2.05025600	-2.49165500	H	-1.92428400	-0.78974700	-2.50220100
				H	1.14397700	3.74973300	-1.12939500
				H	1.14397700	3.74973300	1.12939500
				H	-1.23713500	3.70533900	1.13436700
				H	-1.23713500	3.70533900	-1.13436700
**[7]-cyclacene**				**[7]-cyclacene_H_4**			
C	0.64160800	-2.70796500	0.72823300	C	-2.47797100	1.67018600	0.72194700
C	1.76013800	-2.16994800	1.41011000	C	-1.37161000	2.20956300	1.40460200
C	0.64164400	-2.70792500	-0.72829800	C	-0.14425700	2.30564000	0.72014300
C	-0.59926400	-2.72893800	1.41018100	C	-0.14429700	2.30557200	-0.72035400
C	2.51763100	-1.18686600	0.72826600	C	-1.37168700	2.20943000	-1.40473600
H	1.73047500	-2.13347600	2.49641300	C	-2.47801100	1.67011800	-0.72197000
C	1.76002700	-2.16992900	-1.41018400	C	1.06820000	1.97994400	1.40488300
C	-0.59938500	-2.72880400	-1.41021400	C	1.06812200	1.97981100	-1.40513000
C	-1.71735900	-2.19005100	0.72825800	C	2.14834700	1.48552600	-0.71648700

H	-0.58914800	-2.68289200	2.49646800	C	2.14838700	1.48559500	0.71622800
C	2.51764400	-1.18682000	-0.72830600	C	3.28328800	0.72650400	1.35444700
C	2.79453700	0.02307700	1.41011300	H	3.17495800	0.69496200	2.44354500
H	1.73038800	-2.13340000	-2.49648200	C	3.28321300	0.72637500	-1.35469700
C	-1.71735400	-2.19003400	-0.72828700	H	1.05115100	1.93260300	2.49158600
H	-0.58921200	-2.68273900	-2.49651100	H	-1.35072200	2.14656300	2.49015800
C	-2.50758900	-1.23306900	1.41013800	H	-1.35085900	2.14632600	-2.49028800
C	2.79448400	0.02303900	-1.41013100	H	1.05101300	1.93236600	-2.49182800
C	2.49779400	1.22812200	0.72831000	H	3.17482300	0.69472900	-2.44378600
H	2.74727000	0.02263200	2.49640000	C	3.30659700	-0.73731400	0.78619100
C	-2.50765600	-1.23291600	-1.41016000	C	2.16315100	-1.49279100	1.41194500
C	-2.78329800	-0.02292800	0.72829300	C	1.06659400	-1.97233700	0.73823000
H	-2.46525600	-1.21227600	2.49643900	C	1.06655300	-1.97240700	-0.73810100
C	2.49776500	1.22816700	-0.72828800	C	2.16307400	-1.49292600	-1.41192100
H	2.74718700	0.02264400	-2.49641300	C	3.30655300	-0.73738900	-0.78630300
C	1.72416700	2.19873400	1.41020200	C	-0.14272600	-2.30844000	1.41151800
C	-2.78331400	-0.02294300	-0.72827700	C	-0.14280300	-2.30857400	-1.41129000
H	-2.46529400	-1.21214600	-2.49645300	C	-1.37191900	-2.19436700	-0.73261800
C	-2.52766000	1.19152400	1.41016700	C	-1.37187900	-2.19429800	0.73290200
C	1.72423900	2.19874900	-1.41017300	C	-2.49310200	-1.67565000	1.40991900
H	1.69519700	2.16172300	2.49651000	H	-2.47745300	-1.65892000	2.49699800
C	0.59688300	2.71783500	0.72832100	C	-2.49317900	-1.67578400	-1.40962300
H	1.69525400	2.16180800	-2.49647900	H	-0.14376300	-2.29861700	2.49897900
C	0.59684800	2.71787400	-0.72828000	H	2.14760700	-1.50412800	2.50094400
C	-0.64425800	2.71862700	1.41025500	H	2.14747000	-1.50436700	-2.50091900
C	-0.64418100	2.71874800	-1.41020000	H	-0.14390100	-2.29885400	-2.49875300
C	-2.52765600	1.19168100	-1.41012000	H	-2.47759100	-1.65915700	-2.49670400
C	-1.75321500	2.16141700	0.72833000	C	-3.15661300	-0.60669300	0.72712000
H	-0.63340900	2.67295700	2.49655400	C	-3.17348000	0.62098400	1.40780400
C	-1.75323500	2.16140600	-0.72825800	C	-3.17355800	0.62085100	-1.40768900
H	-0.63342200	2.67311200	-2.49649800	C	-3.15665300	-0.60676200	-0.72688900
H	-2.48495700	1.17135400	2.49645300	H	-3.12708700	0.61009600	2.49410200
H	-2.48499500	1.17150000	-2.49640800	H	-3.12722400	0.60986000	-2.49398800
				H	4.25044700	1.20285500	1.14303600
				H	4.25038400	1.20274600	-1.14338400
				H	4.25033600	-1.18567300	-1.12796200
				H	4.25039800	-1.18556500	1.12784100
**[8]-cyclacene**				**[8]-cyclacene_H$_4$**			
C	0.00000000	3.16570400	0.72877900	C	-1.65098000	-2.04013900	0.73820900
C	-1.21592000	2.93549000	1.40788400	C	-0.47933500	-2.48589200	1.41020400
C	-2.23849100	2.23849100	0.72877900	C	0.74662100	-2.60089200	0.73283800
C	-2.23849100	2.23849100	-0.72877900	C	0.74662800	-2.60088400	-0.73285800
C	-1.21592000	2.93549000	-1.40788400	C	-0.47932100	-2.48587700	-1.41023500
C	0.00000000	3.16570400	-0.72877900	C	-1.65097200	-2.04013100	-0.73824600
C	-2.93549000	1.21592000	1.40788400	C	1.96288600	-2.39766200	1.41011700
C	-2.93549000	1.21592000	-1.40788400	C	1.96290000	-2.39764800	-1.41012300
C	-3.16570400	0.00000000	-0.72877900	C	2.99096100	-1.69259600	-0.72919100
C	-3.16570400	0.00000000	0.72877900	C	2.99095400	-1.69260300	0.72920300
C	-2.93549000	-1.21592000	1.40788400	C	3.62534700	-0.64089900	1.40970700
H	-2.90036900	-1.20137200	2.49479800	H	3.59127500	-0.63128300	2.49643000
C	-2.93549000	-1.21592000	-1.40788400	C	3.62536100	-0.64088400	-1.40967800
H	-2.90036900	1.20137200	2.49479800	H	1.95341600	-2.37574500	2.49728000
H	-1.20137200	2.90036900	2.49479800	H	-0.47718400	-2.48045700	2.49784200
H	-1.20137200	2.90036900	-2.49479800	H	-0.47715900	-2.48043200	-2.49787200
H	-2.90036900	1.20137200	-2.49479800	H	1.95344100	-2.37571900	-2.49728500
H	-2.90036900	-1.20137200	-2.49479800	H	3.59130000	-0.63125700	-2.49640100
C	-2.23849100	-2.23849100	0.72877900	C	3.61202800	0.61521800	0.72651600
C	-1.21592000	-2.93549000	1.40788400	C	3.01697600	1.68559200	1.40876700

C	0.00000000	-3.16570400	0.72877900	C	1.98388100	2.38395200	0.72389100
C	0.00000000	-3.16570400	-0.72877900	C	1.98388800	2.38395900	-0.72384700
C	-1.21592000	-2.93549000	-1.40788400	C	3.01699000	1.68560700	-1.40872000
C	-2.23849100	-2.23849100	-0.72877900	C	3.61203500	0.61522500	-0.72647400
C	1.21592000	-2.93549000	1.40788400	C	0.77271700	2.61172100	1.40525600
C	1.21592000	-2.93549000	-1.40788400	C	0.77273100	2.61173500	-1.40522100
C	2.23849100	-2.23849100	-0.72877900	C	-0.44628100	2.48701200	-0.72218800
C	2.23849100	-2.23849100	0.72877900	C	-0.44628800	2.48700500	0.72220900
C	2.93549000	-1.21592000	1.40788400	C	-1.62564400	2.05433100	1.40501300
H	2.90036900	-1.20137200	2.49479800	H	-1.60687000	2.01194200	2.49197100
C	2.93549000	-1.21592000	-1.40788400	C	-1.62563000	2.05434600	-1.40500800
H	1.20137200	-2.90036900	2.49479800	H	0.76832100	2.55994800	2.49166800
H	-1.20137200	-2.90036900	2.49479800	H	2.97512800	1.65093300	2.49476300
H	-1.20137200	-2.90036900	-2.49479800	H	2.97515300	1.65095900	-2.49471700
H	1.20137200	-2.90036900	-2.49479800	H	0.76834600	2.55997300	-2.49163400
H	2.90036900	-1.20137200	-2.49479800	H	-1.60684500	2.01196800	-2.49196600
C	3.16570400	0.00000000	0.72877900	C	-2.68485900	1.51963200	0.71728500
C	3.16570400	0.00000000	-0.72877900	C	-2.68485200	1.51964000	-0.71729500
H	2.90036900	1.20137200	2.49479800	H	-3.68481100	0.70748800	2.44625000
C	2.93549000	1.21592000	1.40788400	C	-3.80022300	0.74253700	1.35798900
C	2.93549000	1.21592000	-1.40788400	C	-3.80021000	0.74255100	-1.35801900
H	2.90036900	1.20137200	-2.49479800	H	-3.68478700	0.70751400	-2.44627900
C	2.23849100	2.23849100	0.72877900	C	-3.83877500	-0.72421100	0.78568400
C	1.21592000	2.93549000	1.40788400	C	-2.72495500	-1.51284900	1.41257100
C	1.21592000	2.93549000	-1.40788400	C	-2.72494100	-1.51283500	-1.41261400
C	2.23849100	2.23849100	-0.72877900	C	-3.83876800	-0.72420200	-0.78573000
H	1.20137200	2.90036900	2.49479800	H	-2.71693000	-1.53650400	2.50144000
H	1.20137200	2.90036900	-2.49479800	H	-2.71690500	-1.53647800	-2.50148200
				H	-4.77185200	1.21244000	1.15460600
				H	-4.77184000	1.21245200	-1.15464000
				H	-4.79322100	-1.14845100	1.12745000
				H	-4.79321000	-1.14843900	-1.12750900
**[9]-cyclacene**				**[9]-cyclacene_H$_4$**			
C	-0.61776200	3.50301400	0.72773400	C	4.06281900	-0.62097600	0.72831900
C	-1.78391200	3.08940900	1.40831100	C	3.51554400	-1.74249600	1.41002400
C	-0.61776200	3.50301400	-0.72773400	C	2.54327400	-2.49312000	0.72968600
C	0.61951700	3.51297900	1.40830900	C	2.54327400	-2.49312000	-0.72968600
C	-2.72547400	2.28665300	0.72773800	C	3.51554300	-1.74249600	-1.41002400
H	-1.76614900	3.05863000	2.49533700	C	4.06281900	-0.62097600	-0.72831900
C	-1.78391200	3.08940900	-1.40831100	C	1.36391100	-2.88058900	1.40917900
C	0.61951700	3.51297900	-1.40830900	C	1.36391100	-2.88058900	-1.40917900
C	1.77881200	3.08055200	0.72773200	C	0.13096700	-2.85722800	-0.73299300
H	0.61334400	3.47798600	2.49533700	C	0.13096700	-2.85722800	0.73299300
C	-2.72547400	2.28665300	-0.72773800	C	-1.06893700	-2.59153200	1.41010000
C	-3.35297400	1.22023300	1.40830900	H	-1.06579300	-2.58605700	2.49778100
H	-1.76614900	3.05863000	-2.49533700	C	-1.06893700	-2.59153200	-1.41010000
C	1.77881200	3.08055200	-0.72773200	H	1.36075100	-2.85985600	2.49653100
H	0.61334400	3.47798600	-2.49533700	H	3.48963800	-1.72213700	2.49688000
C	2.73326000	2.29318000	1.40831600	H	3.48963800	-1.72213700	-2.49688000
C	-3.35297400	1.22023300	-1.40830900	H	1.36075100	-2.85985700	-2.49653100
C	-3.55795500	0.00000000	0.72773400	H	-1.06579300	-2.58605700	-2.49778100
H	-3.31971200	1.20812100	2.49534200	C	-2.21200400	-2.07654600	0.73813400
C	-3.55795500	0.00000000	-0.72773400	C	-3.25731600	-1.49842000	1.41282100
H	-3.31971200	1.20812100	-2.49534200	C	-4.38123200	-0.71893200	0.78616500
C	-3.35297400	-1.22023300	1.40830900	C	-4.38123200	-0.71893300	-0.78616500
C	-3.35297400	-1.22023300	-1.40830900	C	-3.25731600	-1.49842000	-1.41282100
C	2.73326000	2.29318000	-1.40831600	C	-2.21200400	-2.07654600	-0.73813400
H	-3.31971200	-1.20812100	2.49534200	C	-4.34504400	0.73741800	1.35846700

C	-2.72547400	-2.28665300	0.72773800	C	-4.34504400	0.73741800	-1.35846700
H	-3.31971200	-1.20812100	-2.49534200	C	-3.22196400	1.50897900	-0.71833000
C	-2.72547400	-2.28665300	-0.72773800	C	-3.22196400	1.50897900	0.71833000
C	-1.78391200	-3.08940900	-1.40831100	C	-2.18726600	2.08525000	1.40598500
C	-1.78391200	-3.08940900	1.40831100	H	-2.17276700	2.05694400	2.49345000
C	-0.61776200	-3.50301400	0.72773400	C	-2.18726600	2.08525000	-1.40598500
H	-1.76614900	-3.05863000	2.49533700	H	-4.22945400	0.70251000	2.44680200
C	-0.61776200	-3.50301400	-0.72773400	H	-3.23977700	-1.50720200	2.50180100
H	-1.76614900	-3.05863000	-2.49533700	H	-3.23977700	-1.50720300	-2.50180100
C	0.61951700	-3.51297900	1.40830900	H	-4.22945400	0.70251000	-2.44680200
C	0.61951700	-3.51297900	-1.40830900	H	-2.17276700	2.05694300	-2.49345000
C	1.77881200	-3.08055200	0.72773200	C	-1.03644500	2.59116200	0.72314200
H	0.61334400	-3.47798600	2.49533700	C	0.15456800	2.86494200	1.40518300
C	1.77881200	-3.08055200	-0.72773200	C	1.38821800	2.87144800	0.72506600
H	0.61334400	-3.47798600	-2.49533700	C	1.38821800	2.87144800	-0.72506600
C	2.73326000	-2.29318000	1.40831600	C	0.15456800	2.86494200	-1.40518300
C	2.73326000	-2.29318000	-1.40831600	C	-1.03644500	2.59116200	-0.72314200
H	2.70614200	-2.27044200	2.49534900	C	2.56956300	2.49197400	1.40887000
C	3.34340900	-1.21673700	0.72774200	C	2.56956300	2.49197400	-1.40887000
H	2.70614200	-2.27044200	-2.49534900	C	3.52078100	1.72045600	-0.72713200
C	3.34340900	-1.21673700	-0.72774200	C	3.52078100	1.72045600	0.72713200
C	3.56821200	0.00000000	-1.40831900	C	4.08581600	0.60531000	1.41021800
C	3.56821200	0.00000000	1.40831900	H	4.04588000	0.59717900	2.49674600
C	3.34340900	1.21673700	0.72774200	C	4.08581600	0.60531000	-1.41021800
H	3.53270400	0.00000000	2.49534700	H	2.54436300	2.45012100	2.49519100
C	3.34340900	1.21673700	-0.72774200	H	0.15773200	2.82442900	2.49214500
H	3.53270400	0.00000000	-2.49534700	H	0.15773200	2.82442900	-2.49214500
H	2.70614200	2.27044200	2.49534900	H	2.54436300	2.45012100	-2.49519100
H	2.70614200	2.27044200	-2.49534900	H	4.04588000	0.59717900	-2.49674600
				H	-5.31162700	1.21987500	1.15664300
				H	-5.31162600	1.21987400	-1.15664400
				H	-5.33046100	-1.15841300	1.12521700
				H	-5.33046100	-1.15841200	-1.12521700
**[10]-cyclacene**				**[10]-cyclacene_H_4**			
C	0.00000000	3.94121100	0.72960900	C	2.77313600	-2.10566700	-0.73834300
C	0.00000000	3.94121100	-0.72960900	C	2.77313600	-2.10566700	0.73834200
C	-1.22073100	3.75702300	1.40894600	C	1.65523500	-2.67110000	-1.40998000
C	-1.22073100	3.75702300	-1.40894600	C	1.65523500	-2.67110000	1.40998000
H	-1.21099700	3.72706400	2.49603200	H	1.65231300	-2.66777000	-2.49768700
H	-1.21099700	3.72706400	-2.49603200	H	1.65231300	-2.66777000	2.49768700
C	-3.74831400	1.21790100	0.72960900	C	-1.96537700	-3.07944200	-0.72958000
C	-3.74831400	1.21790100	-0.72960900	C	-1.96537700	-3.07944200	0.72957900
C	-1.22073100	-3.75702300	1.40894600	C	-4.04190700	1.73040200	-1.41056200
C	-2.31658600	-3.18850700	0.72960900	C	-4.51947500	0.60073000	-0.72871500
C	-3.19591500	-2.32196800	1.40894600	C	-4.52529100	-0.64420900	-1.41023100
C	-2.31658600	-3.18850700	-0.72960900	C	-4.51947500	0.60073000	0.72871500
C	-3.19591500	-2.32196800	-1.40894600	C	-4.52529100	-0.64420900	1.41023100
C	-3.74831400	-1.21790100	-0.72960900	C	-4.00146200	-1.75631800	0.72863900
C	-3.74831400	-1.21790100	0.72960900	C	-4.00146200	-1.75631800	-0.72864000
C	0.00000000	-3.94121100	0.72960900	C	-3.13510000	2.58419800	-0.72808000
C	-1.22073100	-3.75702300	-1.40894600	C	-4.04190700	1.73040200	1.41056200
H	-3.17043000	-2.30345200	-2.49603200	H	-4.49036100	-0.63676900	2.49697700
C	-3.95036800	0.00000000	-1.40894600	C	-3.10223000	-2.61254200	1.40913200
C	-3.95036800	0.00000000	1.40894600	C	-3.10223000	-2.61254200	-1.40913300
H	-3.91886700	0.00000000	-2.49603200	H	-3.08474300	-2.58694800	2.49615200
H	-3.91886700	0.00000000	2.49603200	H	-3.08474300	-2.58694700	-2.49615200
C	0.00000000	-3.94121100	-0.72960900	C	-3.13510000	2.58419800	0.72808100
H	-1.21099700	-3.72706400	2.49603200	H	-4.00899300	1.70900800	-2.49717900

H	-3.17043000	-2.30345200	2.49603200	H	-4.49036100	-0.63676800	-2.49697700
H	-1.21099700	-3.72706400	-2.49603200	H	-4.00899300	1.70900800	2.49717900
C	-3.19591500	2.32196800	1.40894600	C	-0.73524900	-3.22304700	-1.40831000
C	-2.31658600	3.18850700	0.72960900	C	0.48417300	-3.04127500	-0.73285000
C	-2.31658600	3.18850700	-0.72960900	C	0.48417300	-3.04127500	0.73285000
C	-3.19591500	2.32196800	-1.40894600	C	-0.73524900	-3.22304700	1.40831000
H	-3.17043000	2.30345200	2.49603200	H	-0.73578700	-3.20491600	-2.49579500
H	-3.17043000	2.30345200	-2.49603200	H	-0.73578700	-3.20491600	2.49579500
C	1.22073100	-3.75702300	1.40894600	C	-2.01261700	3.07514200	-1.40877000
C	1.22073100	-3.75702300	-1.40894600	C	-2.01261700	3.07514100	1.40877100
H	1.21099700	-3.72706400	2.49603200	H	-2.00022100	3.03635300	-2.49548300
H	1.21099700	-3.72706400	-2.49603200	H	-2.00022100	3.03635200	2.49548300
C	3.74831400	-1.21790100	0.72960900	C	1.60283700	2.67887300	-0.72357100
C	3.74831400	-1.21790100	-0.72960900	C	1.60283700	2.67887300	0.72357200
C	1.22073100	3.75702300	1.40894600	C	3.80880300	-1.50981700	-1.41338200
C	2.31658600	3.18850700	0.72960900	C	4.91319400	-0.70875900	-0.78574900
C	3.19591500	2.32196800	1.40894600	C	4.86723100	0.75038400	-1.36070800
C	2.31658600	3.18850700	-0.72960900	C	4.91319400	-0.70875900	0.78574900
C	3.19591500	2.32196800	-1.40894600	C	4.86723100	0.75038500	1.36070800
C	3.74831400	1.21790100	-0.72960900	C	3.75728400	1.53373300	0.71828300
C	3.74831400	1.21790100	0.72960900	C	3.75728400	1.53373300	-0.71828300
C	1.22073100	3.75702300	-1.40894600	C	3.80880300	-1.50981600	1.41338200
H	3.17043000	2.30345200	-2.49603200	H	4.74651800	0.71332900	2.44838400
C	3.95036800	0.00000000	-1.40894600	C	2.73062800	2.12412900	1.40573300
C	3.95036800	0.00000000	1.40894600	C	2.73062800	2.12412900	-1.40573300
H	3.91886700	0.00000000	-2.49603200	H	2.71481700	2.09542300	2.49318300
H	3.91886700	0.00000000	2.49603200	H	2.71481600	2.09542300	-2.49318300
H	1.21099700	3.72706400	2.49603200	H	3.79824400	-1.52897300	-2.50231000
H	3.17043000	2.30345200	2.49603200	H	4.74651800	0.71332800	-2.44838500
H	1.21099700	3.72706400	-2.49603200	H	3.79824400	-1.52897200	2.50231000
C	3.19591500	-2.32196800	1.40894600	C	0.44008200	3.05120800	-1.40525800
C	2.31658600	-3.18850700	0.72960900	C	-0.78265900	3.21590500	-0.72596500
C	2.31658600	-3.18850700	-0.72960900	C	-0.78265900	3.21590500	0.72596600
C	3.19591500	-2.32196800	-1.40894600	C	0.44008300	3.05120800	1.40525800
H	3.17043000	-2.30345200	2.49603200	H	0.43439100	3.02005700	-2.49253000
H	3.17043000	-2.30345200	-2.49603200	H	0.43439200	3.02005700	2.49253000
				H	5.83708200	1.22783800	-1.16434100
				H	5.83708200	1.22783800	1.16434100
				H	5.86932900	-1.13180700	-1.12535400
				H	5.86932900	-1.13180700	1.12535400
**[11]-cyclacene**				**[11]-cyclacene_H$_4$**			
C	-0.80136100	4.26009000	0.72815100	C	0.16668000	3.45626700	-0.72627700
C	-0.80136100	4.26009000	-0.72815100	C	0.16668000	3.45626700	0.72627500
C	-1.97304800	3.86983100	1.40824400	C	-3.31948800	-2.12378600	-0.73806600
C	0.43202300	4.32247200	1.40825600	C	-3.31948800	-2.12378600	0.73806800
C	-1.97304800	3.86983100	-1.40824400	C	4.98834600	0.59744700	-1.41028400
C	0.43202300	4.32247200	-1.40825600	C	4.51786400	1.74678200	-0.72917700
C	-2.97668800	3.15020700	-0.72814500	C	3.69788600	2.65894800	-1.41021600
C	-3.75125200	2.18835400	-1.40826900	C	4.51786400	1.74678300	0.72917600
C	-4.20672500	1.04045700	-0.72814700	C	3.69788600	2.65894900	1.41021400
C	-4.20672500	1.04045700	0.72814700	C	2.60120900	3.24319400	0.72857200
C	-4.33866900	-0.18743300	1.40825900	C	2.60120900	3.24319300	-0.72857500
C	-4.10138500	-1.39938500	0.72814600	C	4.96426200	-0.63276400	-0.72861700
H	-4.31054600	-0.18621600	2.49535800	C	4.98834600	0.59744800	1.41028300
C	-3.75125200	2.18835400	1.40826900	H	3.67601800	2.63271800	2.49704900
C	-2.97668800	3.15020700	0.72814500	C	1.40256200	3.49745700	1.40827800
H	-3.72685500	2.17414500	2.49536200	C	1.40256200	3.49745600	-1.40828100
C	-4.33866900	-0.18743300	-1.40825900	H	1.39818500	3.46480900	2.49531100

H	-3.72685500	2.17414500	-2.49536200	H	1.39818500	3.46480800	-2.49531400
C	-4.10138500	-1.39938500	-0.72814600	C	4.96426200	-0.63276400	0.72861700
C	-3.54870600	-2.50376500	1.40825800	H	4.95541500	0.59143500	-2.49709400
H	-4.31054600	-0.18621600	-2.49535800	H	3.67601700	2.63271600	-2.49705100
H	-1.96021100	3.84470400	-2.49534300	H	4.95541500	0.59143700	2.49709300
H	-1.96021100	3.84470400	2.49534300	C	-2.22020900	-2.72301200	-1.40989700
C	-3.54870600	-2.50376500	-1.40825800	C	-1.07436400	-3.16571200	-0.73279100
C	-2.69403900	-3.39522900	0.72814700	C	0.12165300	-3.46087300	-1.40789100
C	-1.63205900	-4.02556900	1.40824900	C	-1.07436400	-3.16571100	0.73279300
C	-0.43108300	-4.31331900	0.72815100	C	0.12165300	-3.46087200	1.40789300
C	0.80308900	-4.26915800	1.40825800	C	1.35880600	-3.49776600	0.72947300
C	1.96888900	-3.86167000	0.72815800	C	1.35880600	-3.49776600	-0.72947100
C	2.98305600	-3.15693200	1.40826200	C	-2.22020900	-2.72301100	1.40989900
C	3.74325300	-2.18371500	0.72813900	C	2.56459100	-3.26211400	1.40828500
C	3.74325300	-2.18371500	-0.72813900	C	2.56459100	-3.26211500	-1.40828300
C	4.21562400	-1.04266600	1.40826000	H	-2.21712600	-2.72034400	-2.49760800
C	2.98305600	-3.15693200	-1.40826200	H	-2.21712600	-2.72034200	2.49761000
C	4.21562400	-1.04266600	-1.40826000	C	3.65436400	-2.67344400	-0.72838000
H	2.96366500	-3.13638600	2.49535900	C	3.65436400	-2.67344400	0.72838200
C	1.96888900	-3.86167000	-0.72815800	C	4.50418700	-1.78349700	1.40938900
H	0.79785000	-4.24132300	2.49535100	C	4.50418700	-1.78349800	-1.40938900
C	0.80308900	-4.26915800	-1.40825800	H	0.12435200	-3.44583300	2.49547500
C	-0.43108300	-4.31331900	-0.72815100	H	0.12435200	-3.44583400	-2.49547300
H	0.79785000	-4.24132300	-2.49535100	H	2.55505100	-3.23649700	-2.49548900
C	-1.63205900	-4.02556900	-1.40824900	H	2.55505200	-3.23649500	2.49549100
H	2.96366500	-3.13638600	-2.49535900	H	4.47578400	-1.76589100	2.49627500
C	-2.69403900	-3.39522900	-0.72814700	H	4.47578400	-1.76589300	-2.49627400
H	-1.62145300	-3.99942000	-2.49534800	C	-4.33888000	-1.50113900	1.41339400
H	-1.62145300	-3.99942000	2.49534800	C	-5.45004300	-0.70688000	0.78606600
H	-3.52565900	-2.48752700	-2.49535300	C	-5.40745900	0.74682400	1.36070400
H	-3.52565900	-2.48752700	2.49535300	C	-5.45004300	-0.70688000	-0.78606500
C	4.32934200	0.18704200	-0.72814600	C	-5.40745900	0.74682300	-1.36070400
H	4.18831200	-1.03591500	-2.49535700	C	-4.29364300	1.52768800	-0.71855200
C	4.11011200	1.40238300	-1.40826200	C	-4.29364300	1.52768800	0.71855100
C	4.32934200	0.18704200	0.72814600	C	-4.33888000	-1.50114000	-1.41339200
C	3.54117200	2.49848000	-0.72814500	C	-3.28048700	2.13978400	-1.40602700
H	4.08343500	1.39329100	-2.49535800	C	-3.28048700	2.13978500	1.40602500
C	4.11011200	1.40238300	1.40826200	H	-4.32197000	-1.51114200	2.50233200
C	3.54117200	2.49848000	0.72814500	H	-4.32197000	-1.51114300	-2.50233000
H	4.08343500	1.39329100	2.49535800	C	-2.17120800	2.73040300	0.72376800
C	2.69982900	3.40249400	1.40826200	C	-2.17120800	2.73040200	-0.72377000
C	2.69982900	3.40249400	-1.40826200	C	-1.03417600	3.17349300	-1.40504600
H	4.18831200	-1.03591500	2.49535700	C	-1.03417600	3.17349400	1.40504400
C	1.62861700	4.01702500	0.72815400	H	-5.28709700	0.71003200	-2.44849400
H	2.68229900	3.38039200	2.49536000	H	-5.28709700	0.71003400	2.44849500
C	1.62861700	4.01702500	-0.72815400	H	-3.26770000	2.11819000	2.49367400
H	2.68229900	3.38039200	-2.49536000	H	-3.26770100	2.11818900	-2.49367500
H	0.42920400	4.29428700	2.49535000	H	-1.02764100	3.14881500	-2.49251400
H	0.42920400	4.29428700	-2.49535000	H	-1.02764000	3.14881600	2.49251200
				H	-6.37515200	1.22972300	-1.16485000
				H	-6.37515200	1.22972400	1.16485000
				H	-6.40283100	-1.13981300	1.12381400
				H	-6.40283200	-1.13981400	-1.12381300
**[12]-cyclacene**				**[12]-cyclacene_H$_4$**			
C	0.00000000	4.72066800	0.72971900	C	2.72407400	2.77805400	0.72376900
C	0.00000000	4.72066800	-0.72971900	C	2.72407400	2.77805400	-0.72376900
C	1.22389400	4.56763400	1.40890900	C	0.48945000	-3.64013000	1.40759300
C	-1.22389400	4.56763400	1.40890900	C	-0.73626400	-3.81120200	0.72945300

C	1.22389400	4.56763400	-1.40890900
C	-1.22389400	4.56763400	-1.40890900
C	2.36033400	4.08821900	0.72971900
H	1.21704100	4.54205800	2.49617200
C	3.34374000	3.34374000	1.40890900
C	2.36033400	4.08821900	-0.72971900
C	4.08821900	2.36033400	0.72971900
H	3.32501700	3.32501700	2.49617200
C	3.34374000	3.34374000	-1.40890900
C	4.08821900	2.36033400	-0.72971900
H	3.32501700	3.32501700	-2.49617200
C	4.56763400	1.22389400	-1.40890900
C	4.56763400	1.22389400	1.40890900
H	1.21704100	4.54205800	-2.49617200
C	4.72066800	0.00000000	-0.72971900
H	4.54205800	1.21704100	-2.49617200
C	4.72066800	0.00000000	0.72971900
H	4.54205800	1.21704100	2.49617200
C	4.56763400	-1.22389400	1.40890900
C	4.08821900	-2.36033400	0.72971900
C	3.34374000	-3.34374000	1.40890900
C	2.36033400	-4.08821900	0.72971900
H	3.32501700	-3.32501700	2.49617200
C	1.22389400	-4.56763400	1.40890900
C	2.36033400	-4.08821900	-0.72971900
C	0.00000000	-4.72066800	0.72971900
H	1.21704100	-4.54205800	2.49617200
C	1.22389400	-4.56763400	-1.40890900
C	3.34374000	-3.34374000	-1.40890900
C	0.00000000	-4.72066800	-0.72971900
H	1.21704100	-4.54205800	-2.49617200
C	-1.22389400	-4.56763400	-1.40890900
C	-1.22389400	-4.56763400	1.40890900
C	4.08821900	-2.36033400	-0.72971900
H	3.32501700	-3.32501700	-2.49617200
H	-1.21704100	-4.54205800	-2.49617200
C	-2.36033400	-4.08821900	-0.72971900
H	-1.21704100	-4.54205800	2.49617200
C	-2.36033400	-4.08821900	0.72971900
C	4.56763400	-1.22389400	-1.40890900
C	-3.34374000	-3.34374000	1.40890900
C	-3.34374000	-3.34374000	-1.40890900
H	4.54205800	-1.21704100	-2.49617200
H	4.54205800	-1.21704100	2.49617200
C	-2.36033400	4.08821900	-0.72971900
C	-3.34374000	3.34374000	-1.40890900
C	-4.08821900	2.36033400	-0.72971900
C	-4.08821900	2.36033400	0.72971900
C	-4.56763400	1.22389400	1.40890900
C	-4.72066800	0.00000000	0.72971900
H	-4.54205800	1.21704100	2.49617200
C	-3.34374000	3.34374000	1.40890900
C	-2.36033400	4.08821900	0.72971900
H	-3.32501700	3.32501700	2.49617200
C	-4.56763400	1.22389400	-1.40890900
H	-3.32501700	3.32501700	-2.49617200
C	-4.72066800	0.00000000	-0.72971900
C	-4.56763400	-1.22389400	1.40890900
H	-4.54205800	1.21704100	-2.49617200
H	-1.21704100	4.54205800	-2.49617200
C	-1.96289800	-3.75656000	1.40769500
C	-0.73626400	-3.81120200	-0.72945300
C	-1.96289800	-3.75656000	-1.40769500
C	-3.14726400	-3.39835200	-0.72829600
C	-3.14726400	-3.39835200	0.72829600
C	1.66068700	-3.26099700	0.73278300
C	0.48945000	-3.64013100	-1.40759300
H	-1.95871000	-3.73299600	-2.49503200
C	-4.19154200	-2.74721800	-1.40869600
C	-4.19154200	-2.74721800	1.40869600
H	-4.17038800	-2.72397700	-2.49571600
H	-4.17038800	-2.72397700	2.49571600
C	1.66068700	-3.26099700	-0.73278300
H	0.48583700	-3.62799000	2.49522200
H	-1.95871000	-3.73299600	2.49503200
H	0.48583800	-3.62799100	-2.49522100
C	-4.97832400	-1.79419500	0.72837400
C	-4.97832400	-1.79419500	-0.72837400
C	3.81864400	2.16135900	1.40586700
C	4.82714700	1.54149700	0.71841600
C	5.93454200	0.75497900	1.36187600
C	4.82714700	1.54149700	-0.71841600
C	5.93454200	0.75497900	-1.36187600
C	5.98234000	-0.69973400	-0.78577800
C	5.98234000	-0.69973400	0.78577800
C	3.81864400	2.16135800	-1.40586700
C	4.88105600	-1.50490900	-1.41360000
C	4.88105600	-1.50490900	1.41360000
H	3.80533000	2.13974100	2.49350500
H	3.80533000	2.13974000	-2.49350500
C	3.86742400	-2.13746600	0.73822300
C	3.86742400	-2.13746600	-0.73822200
C	2.78405300	-2.76355300	-1.40994400
C	2.78405300	-2.76355200	1.40994400
H	5.81135500	0.71704500	-2.44928800
H	5.81135500	0.71704500	2.44928800
H	4.86797500	-1.52030900	2.50252700
H	4.86797600	-1.52031000	-2.50252700
H	2.78135900	-2.76213700	-2.49765100
H	2.78135900	-2.76213700	2.49765100
C	-3.20506700	3.38498300	1.40989900
C	-2.01554200	3.74027100	0.72895800
C	-0.79275600	3.81257300	1.40801600
C	-2.01554200	3.74027100	-0.72895800
C	-0.79275600	3.81257300	-1.40801600
C	0.43112400	3.63933900	-0.72650700
C	0.43112400	3.63933900	0.72650700
C	-4.22797200	2.70891500	0.72972700
C	-3.20506700	3.38498300	-1.40989900
H	-0.79253400	3.78535200	-2.49524100
C	1.60859300	3.27249800	-1.40499300
C	1.60859300	3.27249800	1.40499300
H	1.60221600	3.25273600	-2.49255900
H	1.60221600	3.25273600	2.49255900
C	-4.22797200	2.70891500	-0.72972700
H	-3.19170400	3.35891500	2.49693100
H	-0.79253400	3.78535200	2.49524100
H	-3.19170400	3.35891500	-2.49693100
C	-5.42416500	-0.64482700	-1.40972900
C	-5.42416500	-0.64482700	1.40972900

H	-1.21704100	4.54205800	2.49617200	C	-5.42181900	0.59210300	0.72918400
C	-4.56763400	-1.22389400	-1.40890900	C	-5.42181900	0.59210300	-0.72918400
C	-4.08821900	-2.36033400	-0.72971900	C	-5.02143500	1.75592800	-1.41033000
H	-3.32501700	-3.32501700	-2.49617200	C	-5.02143500	1.75592800	1.41033000
C	-4.08821900	-2.36033400	0.72971900	H	-5.39246100	-0.63935500	2.49663400
H	-3.32501700	-3.32501700	2.49617200	H	-5.39246100	-0.63935500	-2.49663400
H	-4.54205800	-1.21704100	-2.49617200	H	-4.99494700	1.74140900	-2.49726800
H	-4.54205800	-1.21704100	2.49617200	H	-4.99494700	1.74140900	2.49726800
				H	6.90362400	1.23601700	1.16902800
				H	6.90362400	1.23601700	-1.16902900
				H	6.93852500	-1.12459500	-1.12378900
				H	6.93852500	-1.12459500	1.12378900
**[13]-cyclacene**				**[13]-cyclacene_H$_4$**			
C	-4.30695500	2.75561700	0.72865900	C	-5.86374500	-0.64072200	-0.72873700
C	-4.30694200	2.75564800	-0.72880300	C	-5.86374500	-0.64072200	0.72873700
C	-4.84933400	1.64769600	1.40809200	C	1.08243000	-3.77081300	-1.40754800
C	-3.52762400	3.71180400	1.40811400	C	2.23084900	-3.32961400	-0.73283400
C	-4.84929800	1.64772300	-1.40825500	C	3.33608500	-2.79246200	-1.40989200
C	-3.52762600	3.71185400	-1.40824000	C	2.23084900	-3.32961400	0.73283500
C	-5.09486300	0.43882700	0.72864500	C	3.33608500	-2.79246200	1.40989200
H	-4.82684500	1.64002200	2.49532900	C	4.40793000	-2.14777600	0.73806000
C	-2.53253200	4.44080400	0.72867400	C	4.40793000	-2.14777600	-0.73806000
H	-3.51130600	3.69458100	2.49534900	C	-0.12517100	-4.04277200	-0.72957600
C	-5.09482700	0.43885200	-0.72882500	C	1.08243000	-3.77081300	1.40754800
H	-4.82677600	1.64008700	-2.49549100	H	3.33346300	-2.79145100	2.49760100
C	-2.53252200	4.44083400	-0.72879100	C	5.41266000	-1.50147800	1.41360000
H	-3.51129700	3.69468000	-2.49547500	C	5.41266000	-1.50147900	-1.41360000
C	-5.05990400	-0.79422900	1.40808300	H	5.39666100	-1.51279300	2.50251600
C	-1.39816000	4.92541200	1.40812600	H	5.39666100	-1.51279300	-2.50251600
C	-5.05980100	-0.79420400	-1.40826800	C	-0.12517100	-4.04277200	0.72957600
C	-1.39815300	4.92545000	-1.40824000	H	1.07854400	-3.76114000	-2.49522100
C	-4.71507900	-1.97861600	0.72864400	H	3.33346300	-2.79145200	-2.49760100
H	-5.03647300	-0.79056900	2.49531900	H	1.07854300	-3.76113900	2.49522100
C	-0.17831200	5.10874300	0.72866700	C	6.51624700	-0.69881800	-0.78592900
H	-1.39170000	4.90263700	2.49536100	C	6.51624700	-0.69881800	0.78592900
C	-4.71502600	-1.97858800	-0.72881300	C	-5.46867000	-1.80952600	-1.40904300
H	-5.03628800	-0.79050500	-2.49550200	C	-5.46867000	-1.80952600	1.40904300
C	-0.17829200	5.10874300	-0.72878300	C	-4.71804300	-2.78786200	0.72819700
H	-1.39167500	4.90271400	-2.49547600	C	-4.71804300	-2.78786200	-0.72819700
C	-4.11083100	-3.05402900	1.40811000	C	-3.72459700	-3.52090500	1.40813300
C	1.05136900	5.01109200	1.40813100	C	-3.72459700	-3.52090500	-1.40813300
C	-4.11071500	-3.05399700	-1.40824300	C	-2.57434600	-3.95590000	-0.72834200
C	1.05138000	5.01105000	-1.40824600	C	-2.57434600	-3.95590000	0.72834200
C	-3.25496600	-3.94238000	0.72868800	C	-1.34910700	-4.12767200	1.40738500
H	-4.09185600	-3.03991200	2.49534600	C	-1.34910700	-4.12767200	-1.40738500
C	-3.25492400	-3.94236100	-0.72876200	H	-5.44139300	-1.79547300	2.49604000
H	-4.09166300	-3.03983800	-2.49547700	H	-5.44139300	-1.79547300	-2.49604000
C	-2.22011600	-4.61382600	-1.40818400	H	-3.71083000	-3.49648900	-2.49529800
C	-2.22020200	-4.61382300	1.40818500	H	-3.71083000	-3.49648900	2.49529800
C	-1.04957500	-5.00293400	-0.72868500	H	-1.34845900	-4.10750500	2.49484100
H	-2.20986300	-4.59254600	-2.49541900	H	-1.34845900	-4.10750500	-2.49484100
C	-1.04960200	-5.00291700	0.72876900	C	-2.63114800	3.94940400	-1.40945100
H	-2.20999800	-4.59254900	2.49542000	C	-3.76833000	3.49227200	-0.72998200
C	0.17864400	-5.11685900	1.40826900	C	-4.76862100	2.76109200	-1.40995800
C	1.39603500	-4.91773800	0.72884500	C	-3.76833000	3.49227200	0.72998200
H	0.17779700	-5.09321600	2.49550600	C	-4.76862100	2.76109200	1.40995800
C	0.17871700	-5.11693700	-1.40810500	C	-5.48651900	1.76194200	0.72956200
C	1.39606400	-4.91779400	-0.72861500	C	-5.48651900	1.76194200	-0.72956200

C	2.53663000	-4.44803500	1.40832800	C	-1.40173400	4.11657900	-0.72913300
H	0.17790900	-5.09335600	-2.49534200	C	-2.63114800	3.94940400	1.40945100
C	2.53671900	-4.44815700	-1.40805300	H	-4.74962000	2.74276800	2.49703700
H	2.52486500	-4.42739900	2.49556300	C	-5.88612100	0.59269200	1.40972400
C	3.52213500	-3.70610800	0.72888200	C	-5.88612100	0.59269200	-1.40972400
H	2.52500900	-4.42762300	-2.49529100	H	-5.85753100	0.58808800	2.49672100
C	3.52217200	-3.70617700	-0.72859300	H	-5.85753100	0.58808800	-2.49672100
C	4.31376400	-2.76007400	1.40831600	C	-1.40173400	4.11657900	0.72913300
C	4.31384300	-2.76018400	-1.40804200	H	-2.62400700	3.92559500	-2.49663900
C	4.84143500	-1.64509200	0.72884700	H	-4.74962000	2.74276800	-2.49703700
H	4.29372100	-2.74720900	2.49555100	H	-2.62400700	3.92559500	2.49663900
C	4.84145900	-1.64515600	-0.72861600	C	6.47036600	0.75362900	-1.36199700
H	4.29385100	-2.74741700	-2.49527800	C	5.36254200	1.54033500	-0.71847300
C	5.10266700	-0.43959500	-1.40809000	C	4.36133700	2.17152800	-1.40595100
C	5.10264300	-0.43950100	1.40825900	C	5.36254200	1.54033500	0.71847200
C	5.05154500	0.79290900	-0.72868300	C	4.36133700	2.17152900	1.40595100
H	5.07901400	-0.43760200	-2.49532400	C	3.27847700	2.80813400	0.72383200
C	5.05154200	0.79297200	0.72878200	C	3.27847700	2.80813400	-0.72383200
H	5.07897600	-0.43741800	2.49549300	C	6.47036600	0.75362900	1.36199600
C	4.72243600	1.98183000	1.40818900	C	2.18098600	3.34095400	1.40491700
C	4.72241700	1.98172700	-1.40816000	C	2.18098600	3.34095400	-1.40491700
C	2.21678800	4.60672800	-0.72878300	H	6.34707500	0.71575900	-2.44943900
H	1.04654000	4.98788500	-2.49548300	H	6.34707500	0.71575900	2.44943800
C	2.21677600	4.60677300	0.72868000	C	1.02582600	3.77209400	-0.72657900
H	1.04650200	4.98796400	2.49536800	C	1.02582600	3.77209400	0.72657900
C	3.26025600	3.94882900	-1.40822300	C	-0.17915500	4.04616300	1.40773600
C	3.26027000	3.94892800	1.40814500	C	-0.17915500	4.04616300	-1.40773600
C	4.10424200	3.04921300	-0.72874200	H	4.34957800	2.15316200	2.49366500
H	3.24519500	3.93052200	-2.49545800	H	4.34957700	2.15316100	-2.49366500
C	4.10424400	3.04927800	0.72871000	H	2.17499100	3.32459100	-2.49255300
H	3.24520300	3.93071100	2.49538100	H	2.17499100	3.32459100	2.49255300
H	4.70056600	1.97270600	2.49542500	H	-0.18114200	4.02365100	2.49509600
H	4.70054100	1.97250400	-2.49539500	H	-0.18114200	4.02365100	-2.49509600
				H	7.43902600	1.23608400	1.16972300
				H	7.43902600	1.23608400	-1.16972400
				H	7.47108900	-1.12783200	-1.12298500
				H	7.47108900	-1.12783200	1.12298500
**[14]-cyclacene**				**[14]-cyclacene_H_4**			
C	0.00000000	5.50170700	0.72956800	C	-0.47759400	4.22100500	-0.72970400
C	0.00000000	5.50170700	-0.72956800	C	-0.47757500	4.22097700	0.72974400
C	1.22588100	5.37093500	1.40857500	C	-2.74583900	-3.39632800	-1.40493400
C	-1.22588100	5.37093500	1.40857500	C	-1.61026100	-3.87631700	-0.72665000
C	1.22588100	5.37093500	-1.40857500	C	-0.42560500	-4.22682800	-1.40759200
C	-1.22588100	5.37093500	-1.40857500	C	-1.61027100	-3.87632100	0.72667900
C	2.38710100	4.95686700	0.72956800	C	-0.42561800	-4.22685600	1.40761600
H	1.22087900	5.34902200	2.49596500	C	0.78533000	-4.40733700	0.72927700
C	3.43484200	4.30715500	1.40857500	C	0.78533900	-4.40732600	-0.72924700
C	2.38710100	4.95686700	-0.72956800	C	-3.82802500	-2.83362000	-0.72389100
C	4.30140800	3.43025800	0.72956800	C	-2.74583600	-3.39627300	1.40494600
H	3.42082800	4.28958200	2.49596500	H	-0.42255500	-4.20791500	2.49505600
C	3.43484200	4.30715500	-1.40857500	C	2.02554000	-4.38938200	1.40916300
C	4.30140800	3.43025800	-0.72956800	C	2.02554200	-4.38939000	-1.40914000
H	3.42082800	4.28958200	-2.49596500	H	2.02229300	-4.36831700	2.49646200
C	4.96349000	2.39029100	-1.40857500	H	2.02228400	-4.36831400	-2.49643900
C	4.96349000	2.39029100	1.40857500	C	-3.82802600	-2.83358100	0.72388500
H	1.22087900	5.34902200	-2.49596500	H	-2.74036800	-3.38268900	-2.49260800
C	5.36376800	1.22424500	-0.72956800	H	-0.42254900	-4.20788900	-2.49503100
H	4.94323900	2.38053900	-2.49596500	H	-2.74038400	-3.38265300	2.49262100

C	5.36376800	1.22424500	0.72956800	C	3.22003300	-4.11741000	-0.73017500
H	4.94323900	2.38053900	2.49596500	C	3.22002500	-4.11740100	0.73019500
C	5.50905800	0.00000000	1.40857500	C	-3.88374100	2.81593800	-1.40992400
C	5.36376800	-1.22424500	0.72956800	C	-4.94666600	2.15698900	-0.73810800
C	4.96349000	-2.39029100	1.40857500	C	-5.94691100	1.50405100	-1.41368000
C	4.30140800	-3.43025800	0.72956800	C	-4.94661000	2.15692800	0.73815900
H	4.94323900	-2.38053900	2.49596500	C	-5.94683900	1.50390300	1.41370600
C	3.43484200	-4.30715500	1.40857500	C	-7.04819400	0.69908900	0.78587300
C	4.30140800	-3.43025800	-0.72956800	C	-7.04822800	0.69910200	-0.78590900
C	2.38710100	-4.95686700	0.72956800	C	-3.88373200	2.81589700	1.40997500
H	3.42082800	-4.28958200	2.49596500	C	-7.00279800	-0.75321300	1.36242900
C	3.43484200	-4.30715500	-1.40857500	C	-7.00269600	-0.75317200	-1.36253800
C	4.96349000	-2.39029100	-1.40857500	H	-3.88130200	2.81535400	-2.49763000
C	2.38710100	-4.95686700	-0.72956800	H	-3.88126900	2.81530200	2.49768100
H	3.42082800	-4.28958200	-2.49596500	C	-5.89816000	-1.54335600	-0.71852400
C	1.22588100	-5.37093500	-1.40857500	C	-5.89820400	-1.54337500	0.71846000
C	1.22588100	-5.37093500	1.40857500	C	-4.90128700	-2.18109600	1.40594000
C	5.36376800	-1.22424500	-0.72956800	C	-4.90121000	-2.18105300	-1.40598100
H	4.94323900	-2.38053900	-2.49596500	H	-5.93127300	1.51566700	2.50262200
H	1.22087900	-5.34902200	-2.49596500	H	-5.93132400	1.51588000	-2.50260000
C	0.00000000	-5.50170700	-0.72956800	H	-6.87834800	-0.71508400	-2.44986000
H	1.22087900	-5.34902200	2.49596500	H	-6.87850600	-0.71518500	2.44975900
C	0.00000000	-5.50170700	0.72956800	H	-4.88977400	-2.16355400	2.49367100
C	5.50905800	0.00000000	-1.40857500	H	-4.88960200	-2.16341700	-2.49370800
C	-1.22588100	-5.37093500	1.40857500	C	5.24389200	2.83233900	-1.40865500
C	-1.22588100	-5.37093500	-1.40857500	C	4.28916700	3.61170000	-0.72829300
H	5.48658200	0.00000000	-2.49596500	C	3.17301100	4.13879300	-1.40782600
H	5.48658200	0.00000000	2.49596500	C	4.28918800	3.61169300	0.72828000
C	-2.38710100	4.95686700	-0.72956800	C	3.17302200	4.13877000	1.40783700
C	-3.43484200	4.30715500	-1.40857500	C	1.97039800	4.39104400	0.72854200
C	-4.30140800	3.43025800	-0.72956800	C	1.97037900	4.39106300	-0.72851900
C	-4.30140800	3.43025800	0.72956800	C	5.93822300	1.81406500	-0.72860400
C	-4.96349000	2.39029100	1.40857500	C	5.24391200	2.83234000	1.40862700
C	-5.36376800	1.22424500	0.72956800	H	3.16494500	4.11559100	2.49512700
H	-4.94323900	2.38053900	2.49596500	C	0.73328200	4.41396000	1.40725400
C	-3.43484200	4.30715500	1.40857500	C	0.73327500	4.41401400	-1.40721300
C	-2.38710100	4.95686700	0.72956800	H	0.73462700	4.39696600	2.49478300
H	-3.42082800	4.28958200	2.49596500	H	0.73461100	4.39706100	-2.49474400
C	-4.96349000	2.39029100	-1.40857500	C	5.93824100	1.81405700	0.72856200
H	-3.42082800	4.28958200	-2.49596500	H	5.22250800	2.81332200	-2.49575500
C	-5.36376800	1.22424500	-0.72956800	H	3.16493700	4.11566400	-2.49511800
C	-5.50905800	0.00000000	1.40857500	H	5.22251700	2.81329700	2.49572600
H	-4.94323900	2.38053900	-2.49596500	C	4.34255400	-3.59388800	-1.40963900
H	-1.22087900	5.34902200	-2.49596500	C	5.27448200	-2.79193500	-0.72989800
H	-1.22087900	5.34902200	2.49596500	C	5.97423000	-1.77411900	-1.40961400
C	-5.50905800	0.00000000	-1.40857500	C	5.27447800	-2.79193200	0.72989000
C	-2.38710100	-4.95686700	0.72956800	C	5.97424300	-1.77410100	1.40959000
H	-1.22087900	-5.34902200	2.49596500	C	4.34255500	-3.59386300	1.40964500
C	-3.43484200	-4.30715500	1.40857500	H	4.32964700	-3.57456200	-2.49683300
C	-2.38710100	-4.95686700	-0.72956800	H	4.32966400	-3.57453700	2.49684000
C	-4.30140800	-3.43025800	0.72956800	C	6.32273200	-0.59104000	-0.72920600
H	-3.42082800	-4.28958200	2.49596500	C	6.32274000	-0.59104500	0.72916700
C	-3.43484200	-4.30715500	-1.40857500	C	6.32465800	0.64169900	1.40925700
C	-4.30140800	-3.43025800	-0.72956800	C	6.32463300	0.64168600	-1.40930000
H	-3.42082800	-4.28958200	-2.49596500	H	5.95087100	-1.76299100	2.49669500
C	-4.96349000	-2.39029100	-1.40857500	H	5.95084500	-1.76301100	-2.49671900
C	-4.96349000	-2.39029100	1.40857500	H	6.29637300	0.63745900	2.49629900
H	-1.22087900	-5.34902200	-2.49596500	H	6.29635100	0.63745100	-2.49634300
C	-5.36376800	-1.22424500	-0.72956800	C	-1.66530900	3.87207600	1.40753100
H	-4.94323900	-2.38053900	-2.49596500	C	-2.79398500	3.38361400	0.73293300

C	-5.36376800	-1.22424500	0.72956800	C	-2.79399700	3.38361600	-0.73288600
H	-4.94323900	-2.38053900	2.49596500	C	-1.66531000	3.87210800	-1.40748200
H	-5.48658200	0.00000000	-2.49596500	H	-1.66147800	3.86435600	2.49522300
H	-5.48658200	0.00000000	2.49596500	H	-1.66149800	3.86441400	-2.49517500
				H	-7.97236400	-1.23408200	-1.17149200
				H	-7.97246500	-1.23408500	1.17129200
				H	-8.00347700	1.12735000	-1.12269900
				H	-8.00341400	1.12739800	1.12273300
**[15]-cyclacene**				**[15]-cyclacene_H$_4$**			
C	-5.65296200	-1.66740300	0.72921900	C	1.35666700	4.73368700	0.72874100
C	-5.65297800	-1.66739900	-0.72910800	C	1.35662700	4.73373200	-0.72866000
C	-5.18886100	-2.80940100	1.40823100	C	3.81798600	-4.27404600	1.40936300
C	-5.88347400	-0.45646700	1.40825500	C	2.62797700	-4.61651700	0.73033700
C	-5.18887900	-2.80937900	-1.40814000	C	1.40911100	-4.73583400	1.40897000
C	-5.88352400	-0.45645500	-1.40812800	C	2.62798900	-4.61654800	-0.73026100
C	-4.48558500	-3.82177400	0.72919800	C	1.40912900	-4.73587100	-1.40890200
H	-5.17077500	-2.79960400	2.49555800	C	0.17324700	-4.63499900	-0.72931400
C	-5.84272600	0.77555600	0.72923700	C	0.17322800	-4.63500300	0.72937300
H	-5.86293500	-0.45487600	2.49558200	C	4.88570900	-3.66594500	0.73007700
C	-4.48558500	-3.82176200	-0.72912900	C	3.81799400	-4.27411300	-1.40930300
H	-5.17080500	-2.79957400	-2.49546700	H	1.40835000	-4.71754700	-2.49628200
C	-5.84276000	0.77555800	-0.72910500	C	-1.02144600	-4.36797600	-1.40750500
H	-5.86302400	-0.45486500	-2.49545600	C	-1.02149600	-4.36801700	1.40753400
C	-3.59704100	-4.67619100	1.40821200	H	-1.01794500	-4.35202300	-2.49499900
C	-5.56045400	1.97549700	1.40825800	H	-1.01801300	-4.35206800	2.49502900
C	-3.59703700	-4.67615100	-1.40816200	C	4.88571500	-3.66597100	-0.73004100
C	-5.56052700	1.97551200	-1.40813200	H	3.80980000	-4.25534600	2.49665400
C	-2.54280000	-5.31499700	0.72918600	H	1.40832900	-4.71749500	2.49635000
H	-3.58451300	-4.65988000	2.49553900	H	3.80980000	-4.25544700	-2.49659400
C	-5.02180600	3.08426400	0.72922000	C	-2.18637300	-3.95734900	0.72672500
H	-5.54107200	1.96861800	2.49558600	C	-2.18633800	-3.95732300	-0.72671700
C	-2.54279500	-5.31497900	-0.72914900	C	4.84820700	3.69827700	1.40831400
H	-3.58449800	-4.65981200	-2.49548800	C	5.75145700	2.86024500	0.72853900
C	-5.02184600	3.08426800	-0.72911300	C	6.42001200	1.82302400	1.40887500
H	-5.54120500	1.96863400	-2.49546100	C	5.75140900	2.86023800	-0.72865300
C	-1.38359400	-5.73421700	-1.40817600	C	6.41994000	1.82299000	-1.40900100
C	-1.38359400	-5.73425000	1.40820600	C	6.76383800	0.64052400	-0.72899300
C	-0.16069900	-5.88923000	-0.72914600	C	6.76387100	0.64052900	0.72887300
H	-1.37877600	-5.71426100	-2.49550300	C	4.84814200	3.69826400	-1.40838800
C	-0.16070200	-5.88924100	0.72917400	C	6.78194200	-0.59379500	-1.40929700
H	-1.37878400	-5.71431800	2.49553400	C	6.78198700	-0.59375700	1.40920400
C	1.06882500	-5.80106900	-1.40817700	H	4.83289900	3.67748100	2.49551800
C	1.06882500	-5.80107400	1.40820800	H	4.83280100	3.67748900	-2.49559300
C	2.24913500	-5.44558400	0.72917700	C	6.43372000	-1.77599500	0.72942900
H	1.06511200	-5.78092800	2.49553400	C	6.43371100	-1.77600800	-0.72948400
C	2.24914200	-5.44558900	-0.72914400	C	5.79462500	-2.82995800	-1.40935800
H	1.06511900	-5.78091700	-2.49550400	C	5.79463900	-2.82990200	1.40935000
C	3.33664100	-4.86521700	1.40821400	H	6.39498800	1.81193800	-2.49611300
C	3.33665400	-4.86524700	-1.40818000	H	6.39509100	1.81197600	2.49598700
C	4.27047200	-4.06055600	0.72918300	H	6.75611400	-0.59010700	2.49632100
H	3.32503500	-4.84828900	2.49554100	H	6.75604000	-0.59016200	-2.49641300
C	4.27048700	-4.06057400	-0.72915600	H	5.77655500	-2.81531500	-2.49655600
H	3.32506700	-4.84833600	-2.49550800	H	5.77659000	-2.81522900	2.49654800
C	5.02790700	-3.08801500	1.40820100	C	-4.42755400	2.83377400	1.41004100
C	5.55358400	-1.97304500	0.72915700	C	-3.35068200	3.42532700	0.73303500
H	5.01042400	-3.07724700	2.49552900	C	-2.23904500	3.95086300	1.40756900
C	5.02793800	-3.08806200	-1.40818600	C	-3.35074200	3.42544500	-0.73286000
C	5.84981000	-0.77646700	1.40817100	C	-2.23912700	3.95107500	-1.40739000

C	5.55359900	-1.97306400	-0.72916500	C	-1.07090400	4.36045200	-0.72975400
H	5.01048700	-3.07732300	-2.49551500	C	-1.07085300	4.36035200	0.72991000
C	5.87631400	0.45593000	0.72913100	C	-5.48328900	2.16369800	0.73817900
H	5.82941800	-0.77375000	2.49549800	C	-4.42767700	2.83406200	-1.40987900
C	5.84983100	-0.77651000	-1.40820700	H	-2.23548800	3.94484700	-2.49509600
C	5.65997300	1.66950100	1.40814000	C	0.12290900	4.63821800	-1.40705700
C	5.87631700	0.45591200	-0.72920100	C	0.12296900	4.63807000	1.40719100
H	5.82945700	-0.77381700	-2.49553500	H	0.12530200	4.62403500	-2.49464000
C	5.18263700	2.80600700	0.72910500	H	0.12541700	4.62377400	2.49477100
H	5.64024600	1.66368500	2.49546900	C	-5.48340800	2.16389800	-0.73804800
C	5.65996400	1.66945400	-1.40824500	H	-4.42517300	2.83338500	2.49774400
C	4.49113100	3.82649600	1.40811200	H	-2.23532200	3.94447300	2.49527300
C	5.18262100	2.80598400	-0.72924100	H	-4.42540300	2.83387300	-2.49758200
H	5.64022800	1.66361300	-2.49557300	C	-3.30477300	-3.43875900	1.40493600
C	3.59264900	4.67041900	0.72907900	C	-4.37422600	-2.85262000	0.72388500
H	4.47551500	3.81319000	2.49544100	C	-5.43966500	-2.18759500	1.40595000
C	4.49108900	3.82643200	-1.40826900	C	-4.37418700	-2.85262000	-0.72396300
C	2.54591800	5.32147900	1.40811300	C	-5.43948700	-2.18744400	-1.40609000
C	3.59262100	4.67038600	-0.72924500	C	-6.43232700	-1.54360200	-0.71861200
H	4.47543600	3.81308300	-2.49559700	C	-6.43243200	-1.54369000	0.71842200
C	1.38191500	5.72720800	0.72909600	C	-3.30471500	-3.43875300	-1.40497300
H	2.53710500	5.30301300	2.49544100	C	-7.53561700	-0.75188900	-1.36287700
C	2.54587000	5.32140600	-1.40827400	C	-7.53584200	-0.75206300	1.36259900
C	1.38188800	5.72717600	-0.72923500	H	-3.29986500	-3.42717000	2.49263500
C	0.16091700	5.89649600	1.40814500	H	-3.29973500	-3.42709800	-2.49266900
H	2.53701100	5.30288700	-2.49560000	C	-7.58045000	0.69951500	0.78586900
C	0.16087400	5.89644500	-1.40825000	C	-7.58050400	0.69960000	-0.78591200
C	-1.06750600	5.79399000	0.72913700	C	-6.47936300	1.50470400	-1.41369300
H	0.16038200	5.87607000	2.49547500	C	-6.47916900	1.50429600	1.41376600
C	-1.06753400	5.79397200	-0.72919900	H	-5.42840500	-2.17100500	-2.49383400
H	0.16029700	5.87597800	-2.49558000	H	-5.42876700	-2.17133900	2.49370100
C	-2.25186700	5.45225800	1.40819000	H	-7.41110800	-0.71401300	2.44988200
C	-2.25191700	5.45224200	-1.40820300	H	-7.41067300	-0.71364500	-2.45013000
C	-3.33253100	4.85921200	0.72917800	H	-6.46335000	1.51598300	-2.50260500
H	-2.24404300	5.43335600	2.49551900	H	-6.46308100	1.51532200	2.50267500
C	-3.33256500	4.85921000	-0.72914400	C	2.58041200	4.63165400	-1.40761600
H	-2.24414100	5.43332300	-2.49553300	C	3.76497500	4.28432100	-0.72846900
C	-4.27566500	4.06550400	1.40823600	C	3.76501800	4.28431500	0.72844000
H	-4.26081100	4.05139700	2.49556400	C	2.58046500	4.63160700	1.40764900
C	-4.27573600	4.06551800	-1.40816000	H	2.57631700	4.61083500	-2.49500200
H	-4.26094500	4.05141500	-2.49548900	H	2.57640800	4.61072300	2.49503300
				H	-8.50552100	-1.23270500	-1.17260900
				H	-8.50573700	-1.23278100	1.17204000
				H	-8.53548000	1.12877600	-1.12240900
				H	-8.53533800	1.12882900	1.12250600
**[16]-cyclacene**				**[16]-cyclacene_H$_4$**			
C	0.00000000	6.28332500	0.72947700	C	0.74254600	-5.00718800	0.72885500
C	0.00000000	6.28332500	-0.72947700	C	0.74254600	-5.00718800	-0.72885500
C	-1.22712600	6.16918000	1.40826700	C	-5.97611100	2.19251900	1.40607000
C	1.22712600	6.16918000	1.40826700	C	-4.91705400	2.86755300	0.72396000
C	-1.22712600	6.16918000	-1.40826700	C	-3.85824500	3.47231200	1.40498500
C	1.22712600	6.16918000	-1.40826700	C	-4.91705400	2.86755300	-0.72396000
C	-2.40452500	5.80503600	-0.72947700	C	-3.85824500	3.47231200	-1.40498500
C	-3.49456000	5.22997800	-1.40826700	C	-2.75461100	4.02151600	-0.72677600
C	-4.44298200	4.44298200	-0.72947700	C	-2.75461100	4.02151600	0.72677600
C	-4.44298200	4.44298200	0.72947700	C	-6.96576000	1.54404900	0.71854900
C	-5.22997800	3.49456000	1.40826700	C	-5.97611200	2.19252000	-1.40606900
C	-5.80503600	2.40452500	0.72947700	H	-3.85381500	3.46226900	-2.49269800

H	-5.21379300	3.48374500	2.49574000
C	-3.49456000	5.22997800	1.40826700
C	-2.40452500	5.80503600	0.72947700
H	-3.48374500	5.21379300	2.49574000
C	-5.22997800	3.49456000	-1.40826700
H	-3.48374500	5.21379300	-2.49574000
C	-5.80503600	2.40452500	-0.72947700
C	-6.16918000	1.22712600	1.40826700
H	-5.21379300	3.48374500	-2.49574000
H	-1.22332900	6.15008900	-2.49574000
H	-1.22332900	6.15008900	2.49574000
C	-6.16918000	1.22712600	-1.40826700
C	-6.28332500	0.00000000	0.72947700
C	-6.16918000	-1.22712600	1.40826700
C	-5.80503600	-2.40452500	0.72947700
C	-5.22997800	-3.49456000	1.40826700
H	-5.21379300	-3.48374500	2.49574000
C	-4.44298200	-4.44298200	0.72947700
C	-5.80503600	-2.40452500	-0.72947700
C	-5.22997800	-3.49456000	-1.40826700
H	-5.21379300	-3.48374500	-2.49574000
C	-4.44298200	-4.44298200	-0.72947700
C	-6.16918000	-1.22712600	-1.40826700
C	-6.28332500	0.00000000	-0.72947700
H	-6.15008900	-1.22332900	-2.49574000
H	-6.15008900	-1.22332900	2.49574000
H	-6.15008900	1.22332900	-2.49574000
H	-6.15008900	1.22332900	2.49574000
C	2.40452500	5.80503600	-0.72947700
C	3.49456000	5.22997800	-1.40826700
C	4.44298200	4.44298200	-0.72947700
C	4.44298200	4.44298200	0.72947700
C	3.49456000	5.22997800	1.40826700
H	3.48374500	5.21379300	2.49574000
C	2.40452500	5.80503600	0.72947700
C	5.22997800	3.49456000	1.40826700
C	5.80503600	2.40452500	0.72947700
H	5.21379300	3.48374500	2.49574000
C	5.22997800	3.49456000	-1.40826700
C	5.80503600	2.40452500	-0.72947700
H	5.21379300	3.48374500	-2.49574000
C	6.16918000	1.22712600	-1.40826700
C	6.16918000	1.22712600	1.40826700
H	3.48374500	5.21379300	-2.49574000
C	6.28332500	0.00000000	-0.72947700
H	6.15008900	1.22332900	-2.49574000
C	6.28332500	0.00000000	0.72947700
H	6.15008900	1.22332900	2.49574000
C	6.16918000	-1.22712600	1.40826700
C	5.80503600	-2.40452500	0.72947700
C	5.22997800	-3.49456000	1.40826700
C	4.44298200	-4.44298200	0.72947700
H	5.21379300	-3.48374500	2.49574000
C	3.49456000	-5.22997800	1.40826700
C	4.44298200	-4.44298200	-0.72947700
C	2.40452500	-5.80503600	0.72947700
H	3.48374500	-5.21379300	2.49574000
C	3.49456000	-5.22997800	-1.40826700
C	5.22997800	-3.49456000	-1.40826700
C	2.40452500	-5.80503600	-0.72947700
C	-1.60789600	4.47975200	-1.40748000
C	-1.60789500	4.47975200	1.40748000
H	-1.60428900	4.46613300	-2.49501100
H	-1.60428800	4.46613300	2.49501100
C	-6.96576000	1.54404900	-0.71854900
H	-5.96555200	2.17709700	2.49382800
H	-3.85381500	3.46226900	2.49269800
H	-5.96555300	2.17709800	-2.49382800
C	-0.43076100	4.81560500	0.72940900
C	-0.43076100	4.81560500	-0.72941000
C	-2.80582500	-4.01318200	1.40747900
C	-3.90283700	-3.45803000	0.73298100
C	-4.96884400	-2.84741100	1.40999000
C	-3.90283600	-3.45803000	-0.73298100
C	-4.96884400	-2.84741100	-1.40999000
C	-6.01885800	-2.16856200	-0.73808700
C	-6.01885900	-2.16856200	0.73808700
C	-2.80582500	-4.01318100	-1.40747900
C	-7.01167600	-1.50481700	-1.41373000
C	-7.01167600	-1.50481800	1.41373100
H	-2.80230800	-4.00791400	2.49519500
H	-2.80230800	-4.00791300	-2.49519500
C	-8.11263200	-0.69969200	0.78586800
C	-8.11263200	-0.69969200	-0.78586800
C	-8.06816800	0.75131700	-1.36294200
C	-8.06816800	0.75131700	1.36294300
H	-4.96653800	-2.84724400	-2.49769000
H	-4.96653800	-2.84724500	2.49769100
H	-6.99553300	-1.51591500	2.50263200
H	-6.99553200	-1.51591400	-2.50263200
H	-7.94285300	0.71301600	-2.45015200
H	-7.94285200	0.71301500	2.45015300
C	6.26305100	-2.89101500	1.40871300
C	5.39052600	-3.76255700	0.72873800
C	4.34854800	-4.42020100	1.40816500
C	5.39052600	-3.76255700	-0.72873800
C	4.34854800	-4.42020100	-1.40816500
C	3.18891000	-4.83265900	-0.72864500
C	3.18891000	-4.83265900	0.72864500
C	6.88119200	-1.82630700	0.72890200
C	6.26305100	-2.89101500	-1.40871300
H	4.33830800	-4.39977900	-2.49546300
C	1.96993000	-5.02775000	-1.40750100
C	1.96993000	-5.02775000	1.40750100
H	1.96850600	-5.00945800	-2.49496000
H	1.96850600	-5.00945800	2.49496100
C	6.88119200	-1.82630700	-0.72890200
H	6.24285000	-2.87582100	2.49589900
H	4.33830800	-4.39977900	2.49546300
H	6.24285000	-2.87582100	-2.49589900
C	0.79329400	5.01174400	1.40879100
C	5.43786700	3.74064700	1.40909300
C	5.43786700	3.74064700	-1.40909400
C	6.28841100	2.84966800	-0.72961500
C	6.28841100	2.84966800	0.72961500
C	0.79329400	5.01174400	-1.40879100
C	6.91112600	1.78613300	-1.40908900
C	6.91112600	1.78613300	1.40908900
H	0.79403200	4.99585300	2.49622500
H	0.79403200	4.99585300	-2.49622600

H	3.48374500	-5.21379300	-2.49574000	C	7.21940000	0.59145300	0.72916800
C	1.22712600	-6.16918000	-1.40826700	C	7.21940000	0.59145200	-0.72916800
C	1.22712600	-6.16918000	1.40826700	C	7.22133100	-0.63983300	-1.40901000
C	5.80503600	-2.40452500	-0.72947700	C	7.22133100	-0.63983300	1.40901000
H	5.21379300	-3.48374500	-2.49574000	H	5.42459700	3.72450900	-2.49637400
H	1.22332900	-6.15008900	-2.49574000	H	5.42459700	3.72451000	2.49637300
C	0.00000000	-6.28332500	-0.72947700	H	6.88905500	1.77703400	2.49628100
H	1.22332900	-6.15008900	2.49574000	H	6.88905500	1.77703300	-2.49628200
C	0.00000000	-6.28332500	0.72947700	H	7.19597600	-0.63644400	-2.49615900
C	6.16918000	-1.22712600	-1.40826700	H	7.19597600	-0.63644400	2.49615900
C	-1.22712600	-6.16918000	1.40826700	C	-0.47998200	-4.81611000	-1.40706700
C	-1.22712600	-6.16918000	-1.40826700	C	-1.65578300	-4.47086000	-0.72992500
H	6.15008900	-1.22332900	-2.49574000	C	-1.65578300	-4.47086000	0.72992500
H	6.15008900	-1.22332900	2.49574000	C	-0.47998200	-4.81611000	1.40706800
H	-1.22332900	-6.15008900	2.49574000	H	-0.47700600	-4.80400900	-2.49468500
C	-2.40452500	-5.80503600	0.72947700	H	-0.47700600	-4.80401000	2.49468500
H	-1.22332900	-6.15008900	-2.49574000	C	4.39060300	4.39483300	-0.73018000
C	-2.40452500	-5.80503600	-0.72947700	C	3.24119000	4.82777900	-1.40910200
H	1.22332900	6.15008900	-2.49574000	C	2.01776200	5.01722300	-0.73038200
H	1.22332900	6.15008900	2.49574000	C	2.01776200	5.01722300	0.73038200
C	-3.49456000	-5.22997800	-1.40826700	C	3.24119000	4.82777900	1.40910100
H	-3.48374500	-5.21379300	-2.49574000	C	4.39060300	4.39483300	0.73018000
C	-3.49456000	-5.22997800	1.40826700	H	3.23639800	4.81048200	-2.49646500
H	-3.48374500	-5.21379300	2.49574000	H	3.23639800	4.81048200	2.49646500
				H	-9.03824400	1.23200900	1.17313100
				H	-9.03824500	1.23200900	-1.17313000
				H	-9.06745400	-1.12938400	1.12229400
				H	-9.06745400	-1.12938400	-1.12229300
**[17]-cyclacene**				**[17]-cyclacene_H$_4$**			
C	-2.92387200	6.00574700	1.40820400	C	-8.60111900	0.74899900	-1.36319800
C	-1.76871700	6.43448000	0.72939800	C	-8.64517300	-0.70187100	-0.78582100
C	-3.97389900	5.36106600	0.72939200	C	-7.54487200	-1.50752200	-1.41377600
H	-2.91595000	5.98949800	2.49558500	C	-8.64517500	-0.70185900	0.78581000
C	-0.55673800	6.65648000	1.40821000	C	-7.54487500	-1.50750100	1.41378000
C	-1.76873400	6.43447000	-0.72942000	C	-6.55368000	-2.17354100	0.73810000
C	0.67529300	6.63905900	0.72940600	C	-6.55367800	-2.17355200	-0.73808400
H	-0.55522100	6.63847800	2.49559300	C	-7.50025000	1.54338800	-0.71856700
C	-0.55674600	6.65647300	-1.40822700	C	-8.60112300	0.74901900	1.36316600
C	-2.92387800	6.00573300	-1.40823000	H	-7.52921100	-1.51929800	2.50267700
C	0.67527500	6.63905900	-0.72941700	C	-5.50796000	-2.85883100	1.41003200
H	-0.55525400	6.63846300	-2.49561000	C	-5.50795700	-2.85885200	-1.41000300
C	1.88562100	6.40826000	-1.40822300	H	-5.50578000	-2.85888800	2.49772800
C	1.88562900	6.40825700	1.40821900	H	-5.50577400	-2.85892500	-2.49770000
C	-3.97391100	5.36104700	-0.72942000	C	-7.50025200	1.54339800	0.71852500
C	-4.89630600	4.54415400	1.40820700	H	-8.47525800	0.71058900	-2.45033200
H	-2.91597900	5.98946400	-2.49561100	H	-7.52920500	-1.51933500	-2.50267300
H	1.88051400	6.39094600	-2.49560600	H	-8.47526400	0.71062500	2.45030100
C	3.02814200	5.94693400	-0.72941000	C	-4.45060100	-3.48404300	-0.73297000
H	1.88054600	6.39093700	2.49560200	C	-4.45060300	-3.48403200	0.73301000
C	3.02815700	5.94692300	0.72941200	C	0.18100000	5.23263900	-1.40873200
C	-4.89630500	4.54412800	-1.40823300	C	-1.02797300	4.95975200	-0.72949600
C	4.07336500	5.29444600	1.40822000	C	-2.18788700	4.56864400	-1.40749500
C	4.07336400	5.29446200	-1.40821200	C	-1.02797500	4.95976200	0.72942100
C	-5.64263100	3.56371900	-0.72942000	C	-2.18789100	4.56866500	1.40742200
H	-4.88307800	4.53183100	-2.49561500	C	-3.31833900	4.07216700	0.72678500
C	-5.64262300	3.56374300	0.72939900	C	-3.31833800	4.07215600	-0.72685300
C	-6.20759100	2.46872700	-1.40822100	C	0.18099700	5.23266000	1.40865600
C	-6.20758800	2.46875100	1.40820700	C	-4.40932000	3.49833700	1.40498200

C	-6.54926900	1.28493000	0.72940600
H	-6.19073700	2.46206700	2.49558700
C	-6.68046700	0.05978700	1.40821900
C	-6.54927400	1.28490400	-0.72941100
C	-6.57135800	-1.16751800	0.72941600
H	-6.66236600	0.05964500	2.49560100
C	-6.25099900	-2.35729300	1.40822800
C	-6.57135700	-1.16754300	-0.72940400
H	-6.23407900	-2.35088600	2.49560900
C	-5.70572300	-3.46222000	0.72942500
C	-6.68047200	0.05976300	-1.40821500
C	-6.25100100	-2.35731700	-1.40820900
H	-6.66237700	0.05958300	-2.49559700
H	-6.23407500	-2.35094600	-2.49559000
C	-5.70571700	-3.46224300	-0.72940100
H	-6.19075400	2.46200700	-2.49560100
H	-4.88306900	4.53189300	2.49558800
H	4.06232700	5.28006700	2.49560100
C	4.97209800	4.45155500	0.72941200
H	4.06231200	5.28011000	-2.49559300
C	4.97208900	4.45157400	-0.72940200
C	-4.97699900	-4.45581000	-1.40820500
H	-4.96350800	-4.44375800	-2.49558600
C	-4.06932400	-5.28905200	-0.72939200
C	-4.97699700	-4.45578700	1.40823100
H	-4.96351800	-4.44370400	2.49561300
C	-4.06933600	-5.28903500	0.72941800
C	5.71105000	3.46560200	-1.40820600
C	6.24460300	2.35496600	-0.72940200
H	5.69558200	3.45622400	-2.49558700
C	6.57744000	1.16861800	-1.40820900
C	6.24460800	2.35494400	0.72941000
C	6.67372000	-0.05976400	-0.72940600
H	6.55959200	1.16546400	-2.49558800
C	6.67371800	-0.05978700	0.72940700
C	6.55550300	-1.28622800	-1.40821300
C	6.57743800	1.16859900	1.40821400
C	6.55549900	-1.28624600	1.40821100
H	6.55959300	1.16541200	2.49559400
C	6.20154200	-2.46643900	-0.72940900
H	6.53771200	-1.28272100	-2.49559300
C	6.20153500	-2.46646100	0.72940300
H	6.53770000	-1.28277300	2.49559100
C	5.64824800	-3.56739400	1.40820700
C	5.64824900	-3.56737500	-1.40821500
C	4.89177700	-4.53998000	-0.72941600
H	5.63294500	-3.55768100	-2.49559700
C	4.89176600	-4.53999900	0.72940700
C	3.97800600	-5.36656500	-1.40821800
C	3.97800100	-5.36657900	1.40821000
C	2.92119400	-6.00009800	0.72940600
H	3.96720300	-5.35203600	2.49559200
C	1.77056300	-6.44080500	1.40821800
C	2.92120900	-6.00008700	-0.72941000
C	0.55628400	-6.64990100	0.72941200
H	1.76575900	-6.42336100	2.49560000
C	1.77057100	-6.44079800	-1.40821800
C	0.55630200	-6.64989800	-0.72940700
H	1.76579100	-6.42334400	-2.49560000
C	-0.67584600	-6.64529200	-1.40821000
C	-4.40931600	3.49831600	-1.40504500
H	0.18264000	5.21882500	-2.49620700
H	0.18263400	5.21886200	2.49613000
C	-5.45921500	2.87847900	-0.72399200
C	-5.45921600	2.87849000	0.72393600
C	-6.51295300	2.19529900	1.40607800
C	-6.51295000	2.19527800	-1.40612600
H	-2.18435100	4.55697500	2.49497900
H	-2.18434500	4.55693800	-2.49505200
H	-4.40534900	3.48954900	-2.49276600
H	-4.40535500	3.48958500	2.49270300
H	-6.50251800	2.18027700	2.49383500
H	-6.50251200	2.18024000	-2.49388300
C	7.35797400	-1.83156400	-1.40882000
C	7.66174500	-0.63859800	-0.72907200
C	7.67657000	0.59513800	-1.40890700
C	7.66174300	-0.63858700	0.72910000
C	7.67656700	0.59515800	1.40891700
C	7.36781000	1.78720800	0.72927600
C	7.36781200	1.78719700	-0.72928400
C	6.75775100	-2.90935200	-0.72896900
C	7.35797000	-1.83154300	1.40886600
H	7.65281100	0.59225300	2.49611600
C	6.79307100	2.87818200	1.40885200
C	6.79307400	2.87816100	-1.40887800
H	6.77508000	2.86564500	2.49611000
H	6.77508600	2.86560800	-2.49613600
C	6.75774900	-2.90934100	0.72902800
H	7.33542100	-1.82276900	-2.49602100
H	7.65281800	0.59221700	-2.49610600
H	7.33541500	-1.82273200	2.49606600
C	-3.36565600	-4.06227900	-1.40743000
C	1.35603300	-5.34571200	-1.40736000
C	1.35603000	-5.34569100	1.40744200
C	2.58864400	-5.27833300	0.72884200
C	2.58864600	-5.27834300	-0.72875900
C	-3.36565900	-4.06225800	1.40748100
C	3.79083800	-5.01682100	1.40806000
C	3.79084200	-5.01684100	-1.40797700
H	-3.36232500	-4.05782000	-2.49515300
H	-3.36233100	-4.05778300	2.49520400
C	4.92129200	-4.52653600	-0.72887500
C	4.92129100	-4.52652600	0.72895300
C	5.93471200	-3.82494400	1.40858500
C	5.93471500	-3.82496500	-1.40851400
H	1.35629800	-5.32971500	2.49495500
H	1.35630500	-5.32975200	-2.49487300
H	3.78444000	-4.99779400	-2.49535400
H	3.78443400	-4.99775700	2.49543700
H	5.91939800	-3.80792900	2.49584900
H	5.91940400	-3.80796600	-2.49577800
C	2.63683300	5.27853000	1.40888100
C	1.40064700	5.34006600	0.73040400
C	1.40064900	5.34005500	-0.73047900
C	2.63683700	5.27850900	-1.40895200
H	2.63442100	5.26286000	2.49629900
H	2.63442700	5.26282300	-2.49637000
C	0.13471600	-5.22502400	0.72899600
C	-1.07319300	-4.95710300	1.40704900
C	-2.23170900	-4.55811500	0.73000300

C	-0.67585500	-6.64529200	1.40822200	C	-2.23170700	-4.55812600	-0.72994100
H	3.96722700	-5.35200000	-2.49560000	C	-1.07319000	-4.95712300	-1.40697900
H	-0.67400500	-6.62729600	-2.49559200	C	0.13471700	-5.22503400	-0.72891900
C	-1.88367100	-6.40169800	-0.72939900	H	-1.06999200	-4.94679700	2.49469100
H	-0.67403900	-6.62729600	2.49560400	H	-1.06998600	-4.94683300	-2.49462100
C	-1.88368800	-6.40169100	0.72941600	C	5.96513600	3.79107200	0.72969300
H	5.63293300	-3.55773100	2.49558900	C	4.96739000	4.51248300	1.40883300
C	5.71105000	3.46558400	1.40821600	C	3.83237500	4.99822600	0.73022000
H	5.69559600	3.45617800	2.49559700	C	3.83237700	4.99821500	-0.73028400
C	-3.03098500	-5.95239200	-1.40820500	C	4.96739300	4.51246200	-1.40888700
H	-3.02278200	-5.93630900	-2.49558700	C	5.96513800	3.79106200	-0.72973400
C	-3.03099000	-5.95238100	1.40822700	H	4.95806800	4.49622600	2.49617900
H	-3.02280900	-5.93628000	2.49560900	H	4.95807500	4.49619000	-2.49623300
				H	-9.57164000	1.22898400	-1.17395400
				H	-9.57164300	1.22900100	1.17391200
				H	-9.59999900	-1.13150800	1.12226400
				H	-9.59999600	-1.13152400	-1.12227100
**[18]-cyclacene**				**[18]-cyclacene_H_4**			
C	-1.22791700	6.96386600	1.40807000	C	-3.20050400	-5.50872200	1.40788200
C	0.00000000	7.06512300	0.72948600	C	-4.38440300	-5.16726200	0.72905300
C	1.22791700	6.96386600	1.40807000	C	-5.49145100	-4.62649800	1.40840100
C	0.00000000	7.06512300	-0.72948600	C	-4.38440300	-5.16726200	-0.72905300
C	1.22791700	6.96386600	-1.40807000	C	-5.49145100	-4.62649800	-1.40840100
C	2.41641400	6.63904400	-0.72948600	C	-6.46187400	-3.86875600	-0.72916100
C	2.41641400	6.63904400	0.72948600	C	-6.46187400	-3.86875600	0.72916100
C	-2.41641400	6.63904400	0.72948600	C	-1.97775700	-5.64211000	0.72890500
C	-1.22791700	6.96386600	-1.40807000	C	-3.20050400	-5.50872200	-1.40788200
H	1.22494200	6.94699300	-2.49560000	H	-5.48037700	-4.60922600	-2.49573400
C	3.53564700	6.12392100	-1.40807000	C	-7.26153700	-2.93015900	-1.40872100
C	3.53564700	6.12392100	1.40807000	C	-7.26153700	-2.93015900	1.40872100
H	3.52708100	6.10908300	-2.49560000	H	-7.24283800	-2.91788100	-2.49597900
H	3.52708100	6.10908300	2.49560000	H	-7.24283800	-2.91788100	2.49598000
C	-2.41641400	6.63904400	-0.72948600	C	-1.97775700	-5.64211000	-0.72890400
H	-1.22494200	6.94699300	2.49560000	H	-3.19686200	-5.49133300	2.49531600
H	1.22494200	6.94699300	2.49560000	H	-5.48037700	-4.60922600	2.49573400
H	-1.22494200	6.94699300	-2.49560000	H	-3.19686300	-5.49133300	-2.49531600
C	4.54137400	5.41219800	0.72948600	C	-7.81778800	-1.83190700	0.72915500
C	4.54137400	5.41219800	-0.72948600	C	-7.81778800	-1.83190700	-0.72915500
C	-3.53564700	6.12392100	-1.40807000	C	6.04579100	-2.86802500	1.41003500
C	-4.54137400	5.41219800	-0.72948600	C	4.99568600	-3.50514300	0.73299900
C	-5.41692600	4.54534100	-1.40807000	C	3.92097400	-4.10202800	1.40747200
C	-6.11857600	3.53256100	-0.72948600	C	4.99568600	-3.50514300	-0.73299900
C	-6.64484400	2.41852500	-1.40807000	C	3.92097400	-4.10202700	-1.40747200
C	-6.95778800	1.22684600	-0.72948600	C	2.80114000	-4.62870500	-0.73000000
C	-6.95778800	1.22684600	0.72948600	C	2.80114000	-4.62870500	0.73000000
C	-7.07129500	0.00000000	1.40807000	C	6.04579100	-2.86802400	-1.41003500
C	-6.64484400	2.41852500	1.40807000	C	1.65856200	-5.07104600	-1.40697600
C	-6.11857600	3.53256100	0.72948600	C	1.65856200	-5.07104600	1.40697600
C	-7.07129500	0.00000000	-1.40807000	H	6.04359900	-2.86813000	2.49773000
C	-5.41692600	4.54534100	1.40807000	H	6.04359900	-2.86812900	-2.49773000
H	-7.05416200	0.00000000	2.49560000	C	0.46631600	-5.40127100	0.72901500
H	-7.05416200	0.00000000	-2.49560000	C	0.46631600	-5.40127100	-0.72901500
C	-4.54137400	5.41219800	0.72948600	C	-0.74400400	-5.60354400	-1.40730800
C	-3.53564700	6.12392100	1.40807000	C	-0.74400400	-5.60354400	1.40730800
H	-6.62874400	2.41266500	-2.49560000	H	3.91781300	-4.09820400	-2.49519600
H	-6.62874400	2.41266500	2.49560000	H	3.91781300	-4.09820400	2.49519600
H	-5.40380100	4.53432800	2.49560000	H	1.65531500	-5.06220400	2.49463500
H	-5.40380100	4.53432800	-2.49560000	H	1.65531500	-5.06220400	-2.49463500

H	-3.52708100	6.10908300	-2.49560000	H	-0.74531600	-5.58967100	-2.49485900
H	-3.52708100	6.10908300	2.49560000	H	-0.74531600	-5.58967100	2.49485900
C	5.41692600	4.54534100	1.40807000	C	0.42488500	5.41037800	1.40863900
C	5.41692600	4.54534100	-1.40807000	C	1.61786400	5.07517100	0.72950500
H	5.40380100	4.53432800	2.49560000	C	2.76167700	4.63934600	1.40745000
H	5.40380100	4.53432800	-2.49560000	C	1.61786400	5.07517100	-0.72950500
C	-6.95778800	-1.22684600	-0.72948600	C	2.76167700	4.63934600	-1.40745000
C	-6.64484400	-2.41852500	-1.40807000	C	3.87792200	4.11198800	-0.72683600
C	-6.95778800	-1.22684600	0.72948600	C	3.87792200	4.11198800	0.72683600
C	-6.64484400	-2.41852500	1.40807000	C	-0.78446700	5.60125400	0.73050700
H	-6.62874400	-2.41266500	-2.49560000	C	0.42488500	5.41037800	-1.40863900
H	-6.62874400	-2.41266500	2.49560000	H	2.75836500	4.62920900	-2.49502400
C	6.11857600	3.53256100	-0.72948600	C	4.95816900	3.51832600	-1.40503900
C	6.64484400	2.41852500	-1.40807000	C	4.95816900	3.51832600	1.40503900
C	6.95778800	1.22684600	-0.72948600	H	4.95462800	3.51059500	-2.49276800
C	7.07129500	0.00000000	-1.40807000	H	4.95462800	3.51059500	2.49276800
C	6.95778800	-1.22684600	-0.72948600	C	-0.78446700	5.60125400	-0.73050700
C	6.95778800	-1.22684600	0.72948600	H	0.42275100	5.39838700	2.49614000
C	7.07129500	0.00000000	1.40807000	H	2.75836500	4.62920900	2.49502400
C	6.64484400	-2.41852500	-1.40807000	H	0.42275100	5.39838700	-2.49614000
C	6.64484400	-2.41852500	1.40807000	C	-8.12080300	-0.63638000	1.40881900
C	6.11857600	-3.53256100	0.72948600	C	-6.49870300	3.84659200	1.40867400
C	6.11857600	-3.53256100	-0.72948600	C	-6.49870300	3.84659200	-1.40867400
C	5.41692600	-4.54534100	-1.40807000	C	-5.52372100	4.60017900	-0.72979400
C	4.54137400	-5.41219800	-0.72948600	C	-5.52372100	4.60017900	0.72979400
C	3.53564700	-6.12392100	-1.40807000	C	-8.12080300	-0.63638000	-1.40881900
C	2.41641400	-6.63904400	-0.72948600	C	-4.42743600	5.15994100	-1.40867700
C	2.41641400	-6.63904400	0.72948600	C	-4.42743600	5.15994100	1.40867700
C	3.53564700	-6.12392100	1.40807000	H	-8.09790100	-0.63360300	2.49604800
C	1.22791700	-6.96386600	1.40807000	H	-8.09790100	-0.63360300	-2.49604800
C	1.22791700	-6.96386600	-1.40807000	C	-3.23973500	5.49593200	0.73032900
C	0.00000000	-7.06512300	-0.72948600	C	-3.23973500	5.49593200	-0.73032900
C	0.00000000	-7.06512300	0.72948600	C	-2.02109900	5.64590200	-1.40879700
C	4.54137400	-5.41219800	0.72948600	C	-2.02109900	5.64590300	1.40879700
H	1.22494200	-6.94699300	-2.49560000	H	-6.48477100	3.83233000	-2.49599600
C	-1.22791700	-6.96386600	-1.40807000	H	-6.48477100	3.83233000	2.49599600
C	-1.22791700	-6.96386600	1.40807000	H	-4.42126500	5.14432900	2.49607800
H	-1.22494200	-6.94699300	-2.49560000	H	-4.42126500	5.14432900	-2.49607800
H	-1.22494200	-6.94699300	2.49560000	H	-2.02035900	5.63200100	-2.49625700
H	3.52708100	-6.10908300	2.49560000	H	-2.02035900	5.63200100	2.49625700
H	1.22494200	-6.94699300	2.49560000	C	8.07694700	-1.50833800	-1.41379700
H	3.52708100	-6.10908300	-2.49560000	C	7.08806100	-2.17757600	-0.73807700
C	5.41692600	-4.54534100	1.40807000	C	7.08806200	-2.17757600	0.73807600
H	7.05416200	0.00000000	-2.49560000	C	8.07694800	-1.50833800	1.41379700
H	7.05416200	0.00000000	2.49560000	H	8.06052600	-1.51907500	-2.50269000
H	6.62874400	-2.41266500	2.49560000	H	8.06052700	-1.51907600	2.50269000
H	6.62874400	-2.41266500	-2.49560000	C	-7.27892700	2.89237600	-0.72936600
H	5.40380100	-4.53432800	-2.49560000	C	-7.84065300	1.79650000	-1.40876100
H	5.40380100	-4.53432800	2.49560000	C	-8.11789000	0.59484400	-0.72918000
C	6.11857600	3.53256100	0.72948600	C	-8.11789000	0.59484400	0.72918000
C	6.64484400	2.41852500	1.40807000	C	-7.84065300	1.79650000	1.40876100
C	6.95778800	1.22684600	0.72948600	C	-7.27892700	2.89237600	0.72936600
H	6.62874400	2.41266500	-2.49560000	H	-7.81973500	1.78888400	-2.49601600
H	6.62874400	2.41266500	2.49560000	H	-7.81973500	1.78888400	2.49601600
C	-2.41641400	-6.63904400	-0.72948600	C	6.00077500	2.88647300	-0.72399800
C	-3.53564700	-6.12392100	-1.40807000	C	7.05024200	2.19684200	-1.40614100
C	-2.41641400	-6.63904400	0.72948600	C	7.05024200	2.19684200	1.40614100
C	-3.53564700	-6.12392100	1.40807000	C	6.00077500	2.88647200	0.72399800
H	-3.52708100	-6.10908300	-2.49560000	H	7.04026300	2.18264600	-2.49391400
H	-3.52708100	-6.10908300	2.49560000	H	7.04026300	2.18264500	2.49391400

C	-4.54137400	-5.41219800	-0.72948600	C	8.03520400	1.54156100	-0.71857200
C	-5.41692600	-4.54534100	-1.40807000	C	9.13559200	0.74643000	-1.36321600
C	-6.11857600	-3.53256100	-0.72948600	C	9.17822100	-0.70392300	-0.78586800
C	-6.11857600	-3.53256100	0.72948600	C	9.17822100	-0.70392300	0.78586800
C	-5.41692600	-4.54534100	1.40807000	C	9.13559200	0.74642900	1.36321600
C	-4.54137400	-5.41219800	0.72948600	C	8.03520400	1.54156000	0.71857200
H	-5.40380100	-4.53432800	-2.49560000	H	9.00972400	0.70815700	-2.45036800
H	-5.40380100	-4.53432800	2.49560000	H	9.00972400	0.70815700	2.45036800
				H	10.10632100	1.22611800	1.17406300
				H	10.10632100	1.22611800	-1.17406300
				H	10.13244600	-1.13517800	-1.12207300
				H	10.13244600	-1.13517800	1.12207200
**[19]-cyclacene**				**[19]-cyclacene_H_4**			
C	7.43783800	-0.61514600	1.40817100	C	8.61144500	1.50997100	1.41363600
C	7.43185200	0.61669300	0.72948600	C	9.71213500	0.70513500	0.78561900
C	7.23463200	1.83264900	1.40817400	C	9.66979800	-0.74524200	1.36312300
C	7.43185200	0.61669300	-0.72948600	C	9.71210100	0.70513000	-0.78608300
C	7.23463200	1.83264900	-1.40817400	C	9.66972400	-0.74524900	-1.36357900
C	6.82873900	2.99570000	-0.72948200	C	8.57045100	-1.54147300	-0.71875800
C	6.82873900	2.99570000	0.72948200	C	8.57049100	-1.54147000	0.71836600
C	7.22922800	-1.82920100	0.72948300	C	7.62348600	2.18050300	0.73789000
C	7.43783800	-0.61514600	-1.40817100	C	8.61139400	1.50997800	-1.41405600
H	7.21890900	1.82866700	-2.49557100	H	9.54353100	-0.70694400	-2.45069200
C	6.24721400	4.08164400	-1.40816500	C	7.58674700	-2.19860500	-1.40631700
C	6.24721400	4.08164400	1.40816500	C	7.58682200	-2.19859900	1.40598100
H	6.23367100	4.07277700	-2.49556400	H	7.57679100	-2.18459500	-2.49409000
H	6.23367100	4.07277700	2.49556400	H	7.57692500	-2.18458400	2.49375500
C	7.22922800	-1.82920100	-0.72948300	C	7.62345900	2.18050600	-0.73827100
H	7.42170400	-0.61381200	2.49556900	H	8.59538700	1.52118300	2.50252600
H	7.21890900	1.82866700	2.49557100	H	9.54366500	-0.70693300	2.45024300
H	7.42170400	-0.61381200	-2.49556900	H	8.59529800	1.52119700	-2.50294500
C	5.48549000	5.04976700	0.72948600	C	6.54076100	-2.89329700	0.72386700
C	5.48549000	5.04976700	-0.72948600	C	6.54072200	-2.89330200	-0.72414300
C	6.83468600	-2.99615200	-1.40817400	C	-0.13570000	5.81420000	1.40720500
C	6.24301900	-4.07660500	-0.72948800	C	1.06134700	5.54477800	0.72899500
C	5.49077300	-5.05210800	-1.40817600	C	2.23813500	5.16344200	1.40686400
C	4.58028900	-5.88181600	-0.72947800	C	1.06131500	5.54477300	-0.72911100
C	3.55218800	-6.56036500	-1.40817100	C	2.23807400	5.16343400	-1.40702900
C	2.42175000	-7.04981000	-0.72948400	C	3.36621600	4.68562600	-0.73012000
C	2.42175000	-7.04981000	0.72948400	C	3.36624700	4.68562800	0.72990900
C	1.22906300	-7.35794000	1.40817000	C	-0.13576300	5.81418700	-1.40726900
C	3.55218800	-6.56036500	1.40817100	C	4.47374900	4.13378700	-1.40760200
C	4.58028900	-5.88181600	0.72947800	C	4.47380700	4.13378800	1.40734600
C	1.22906300	-7.35794000	-1.40817000	H	-0.13762700	5.80205900	2.49478500
C	5.49077300	-5.05210800	1.40817600	H	-0.13773900	5.80203700	-2.49484900
H	1.22638900	-7.34193300	2.49556600	C	5.53988100	3.52176600	0.73285200
H	1.22638900	-7.34193300	-2.49556600	C	5.53985200	3.52176700	-0.73315100
C	6.24301900	-4.07660500	0.72948800	C	6.58399600	2.87508000	-1.41022200
C	6.83468600	-2.99615200	1.40817400	C	6.58404900	2.87507500	1.40988200
H	3.54448600	-6.54612500	-2.49556800	H	2.23485500	5.15578900	-2.49469900
H	3.54448600	-6.54612500	2.49556800	H	2.23496300	5.15580200	2.49453500
H	5.47887700	-5.04116700	2.49557300	H	4.47083900	4.13049100	2.49507100
H	5.47887700	-5.04116700	-2.49557300	H	4.47073700	4.13048800	-2.49532700
H	6.81983700	-2.98963200	-2.49557100	H	6.58187000	2.87535000	-2.49791400
H	6.81983700	-2.98963200	2.49557100	H	6.58196500	2.87534300	2.49757400
C	4.58269200	5.88786000	1.40817200	C	-6.08345500	-4.68821400	1.40866400
C	4.58269200	5.88786000	-1.40817200	C	-7.01555700	-3.88215500	0.72962400
H	4.57272200	5.87503400	2.49556900	C	-7.77551300	-2.91324800	1.40878900

H	4.57272200	5.87503400	-2.49556900	C	-7.01559100	-3.88216600	-0.72924100
C	3.54785500	6.55610300	-0.72948600	C	-7.77557900	-2.91326900	-1.40838500
C	2.42189200	7.05575500	-1.40817200	C	-8.29820100	-1.79679200	-0.72901400
C	1.22619000	7.35197300	-0.72948800	C	-8.29816700	-1.79678200	0.72942600
C	-0.00096800	7.45928100	-1.40817600	C	-5.00723300	-5.28790300	0.73001500
C	-1.22809800	7.35165500	-0.72948800	C	-6.08352200	-4.68823500	-1.40831400
C	-1.22809800	7.35165500	0.72948800	H	-7.75799600	-2.90248100	-2.49569200
C	-0.00096800	7.45928100	1.40817600	C	-8.57680200	-0.59735700	-1.40850500
C	-2.42372400	7.05512700	-1.40817200	C	-8.57673700	-0.59733700	1.40891200
C	-2.42372400	7.05512700	1.40817200	H	-8.55504900	-0.59500000	-2.49577100
C	-3.54955700	6.55518300	0.72948600	H	-8.55493500	-0.59496400	2.49617700
C	-3.54955700	6.55518300	-0.72948600	C	-5.00726700	-5.28791400	-0.72970600
C	-4.58422000	5.88667200	-1.40817200	H	-6.07297600	-4.67331900	2.49603900
C	-5.48680100	5.04834400	-0.72948600	H	-7.75787900	-2.90244500	2.49609500
C	-6.24827400	4.08002400	-1.40816500	H	-6.07309400	-4.67335600	-2.49569000
C	-6.82951700	2.99392900	-0.72948200	C	5.50415500	-3.53479700	1.40495200
C	-6.82951700	2.99392900	0.72948200	C	1.02239600	-5.55508700	1.40861200
C	-6.24827400	4.08002400	1.40816500	C	1.02232800	-5.55510400	-1.40858700
C	-7.23510800	1.83077200	1.40817400	C	-0.17413200	-5.81455400	-0.73051200
C	-7.23510800	1.83077200	-1.40817400	C	-0.17409700	-5.81454400	0.73059800
C	-7.43201100	0.61476500	-0.72948600	C	5.50408200	-3.53480700	-1.40517000
C	-7.43201100	0.61476500	0.72948600	C	-1.40420700	-5.94733900	-1.40863400
C	-5.48680100	5.04834400	0.72948600	C	-1.40414000	-5.94731900	1.40878000
H	-7.21938300	1.82679400	-2.49557100	H	5.50102100	-3.52791500	2.49268400
C	-7.43767800	-0.61707600	-1.40817100	H	5.50089200	-3.52793300	-2.49290200
C	-7.43767800	-0.61707600	1.40817100	C	-2.63126500	-5.90749600	0.73049600
H	-7.42154400	-0.61573800	-2.49556900	C	-2.63130000	-5.90750600	-0.73029100
H	-7.42154400	-0.61573800	2.49556900	C	-3.84800200	-5.70120200	-1.40840600
H	-6.23472900	4.07116000	2.49556400	C	-3.84793500	-5.70118100	1.40866600
H	-7.21938300	1.82679400	2.49557100	H	1.01993000	-5.54465300	-2.49610900
H	-6.23472900	4.07116000	-2.49556400	H	1.02005100	-5.54462200	2.49613300
C	-4.58422000	5.88667200	1.40817200	H	-1.40446900	-5.93499600	2.49627100
H	-0.00096600	7.44310500	-2.49557400	H	-1.40458900	-5.93503100	-2.49612500
H	-0.00096600	7.44310500	2.49557400	H	-3.84424200	-5.68671400	-2.49585200
H	-2.41848200	7.03985400	2.49557100	H	-3.84412400	-5.68667600	2.49611100
H	-2.41848200	7.03985400	-2.49557100	C	-4.97329300	5.30843600	-1.40818400
H	-4.57424800	5.87384800	-2.49556900	C	-4.97322900	5.30845500	1.40834500
H	-4.57424800	5.87384800	2.49556900	H	-4.96564900	5.29174800	-2.49557300
C	3.54785500	6.55610300	0.72948600	H	-4.96553600	5.29178200	2.49573400
C	2.42189200	7.05575500	1.40817200	C	2.19966400	-5.16901800	-0.72955600
C	1.22619000	7.35197300	0.72948800	C	3.32887500	-4.69680600	-1.40749500
H	2.41665500	7.04048100	-2.49557100	C	4.43286300	-4.14444200	-0.72693300
H	2.41665500	7.04048100	2.49557100	C	4.43290000	-4.14443600	0.72677500
C	-7.22875200	-1.83107700	-0.72948300	C	3.32894500	-4.69679200	1.40739800
C	-6.83390700	-2.99792500	-1.40817400	C	2.19970000	-5.16901000	0.72951900
C	-7.22875200	-1.83107700	0.72948300	H	3.32577000	-4.68792600	-2.49508100
C	-6.83390700	-2.99792500	1.40817400	H	3.32589400	-4.68790100	2.49498300
H	-6.81906000	-2.99140000	-2.49557100	C	-8.56455000	0.63540700	-0.72902200
H	-6.81906000	-2.99140000	2.49557100	C	-8.29212900	1.83621800	-1.40856200
C	-6.24196000	-4.07822400	-0.72948800	C	-8.29206500	1.83623800	1.40892200
C	-5.48946100	-5.05353200	-1.40817600	C	-8.56451700	0.63541700	0.72941100
C	-4.57876200	-5.88300400	-0.72947800	H	-8.27173800	1.82904000	-2.49583500
C	-3.55048500	-6.56128600	-1.40817100	H	-8.27162500	1.82907500	2.49619500
H	-3.54278600	-6.54704400	-2.49556800	C	-7.74772700	2.94233700	-0.72912800
C	-4.57876200	-5.88300400	0.72947800	C	-6.99265100	3.91472200	-1.40847000
C	-3.55048500	-6.56128600	1.40817100	C	-6.04576400	4.70388700	-0.72917900
H	-3.54278600	-6.54704400	2.49556800	C	-6.04573100	4.70389700	0.72939700
C	-5.48946100	-5.05353200	1.40817600	C	-6.99258700	3.91474200	1.40874200
C	-6.24196000	-4.07822400	0.72948800	C	-7.74769500	2.94234700	0.72944700
H	-5.47756800	-5.04258800	-2.49557300	H	-6.97795700	3.90070100	-2.49578700

H	-5.47756800	-5.04258800	2.49557300	H	-6.97784500	3.90073700	2.49605900
C	-2.41992000	-7.05043800	-0.72948400	C	-3.80668600	5.70383700	-0.72911100
C	-1.22715300	-7.35825800	-1.40817000	C	-2.59304000	5.91586000	-1.40774600
C	0.00096700	-7.45393600	-0.72948100	C	-1.36344300	5.94096300	-0.72898000
C	0.00096700	-7.45393600	0.72948100	C	-1.36341000	5.94097100	0.72897000
C	-1.22715300	-7.35825800	1.40817000	C	-2.59297700	5.91587700	1.40779200
C	-2.41992000	-7.05043800	0.72948400	C	-3.80665300	5.70384600	0.72921400
H	-1.22448300	-7.34225000	-2.49556600	H	-2.59136600	5.90028600	-2.49522800
H	-1.22448300	-7.34225000	2.49556600	H	-2.59125300	5.90031500	2.49527300
				H	10.64087900	-1.22429700	1.17425300
				H	10.64081400	-1.22430700	-1.17476100
				H	10.66643300	1.13621200	1.12180300
				H	10.66639000	1.13619300	-1.12231100
**[20]-cyclacene**				**[20]-cyclacene_H$_4$**			
C	4.76118000	-6.24471500	1.40813800	C	-2.81120500	5.23973700	1.40692700
C	0.18125500	-7.84989500	1.40814700	C	1.97947700	6.25578200	1.40768300
C	0.18125400	-7.84991500	-1.40816000	C	1.97947800	6.25578100	-1.40768200
C	-1.04828200	-7.77608400	-0.72953200	C	3.20727200	6.15517600	-0.72923500
C	-1.04827900	-7.77608400	0.72953200	C	3.20727100	6.15517500	0.72923700
C	5.67543600	-5.41925600	0.72953100	C	-3.92630500	4.73242900	0.73002400
C	4.76119300	-6.24473300	-1.40815500	C	-2.81120100	5.23972900	-1.40692800
H	0.18089800	-7.83452300	-2.49560400	H	1.97912200	6.24182800	-2.49519700
C	-2.25353200	-7.52194000	-1.40814300	C	4.40952700	5.88789100	-1.40814500
C	-2.25353600	-7.52196100	1.40815700	C	4.40952600	5.88788900	1.40814800
H	-2.24911200	-7.50716400	-2.49560500	H	4.40447000	5.87229100	-2.49558300
H	-2.24913100	-7.50724000	2.49560100	H	4.40446800	5.87228900	2.49558600
C	5.67543500	-5.41925300	-0.72953600	C	-3.92630200	4.73242400	-0.73002500
H	4.75178300	-6.23240100	2.49559900	H	-2.80811900	5.23309500	2.49460700
H	0.18090000	-7.83444900	2.49560800	H	1.97912000	6.24182900	2.49519900
H	4.75183200	-6.23246400	-2.49560000	H	-2.80811300	5.23308100	-2.49460800
C	-3.40013200	-7.07192100	0.72952800	C	5.54999000	5.42449200	0.72939300
C	-3.40013200	-7.07192400	-0.72952500	C	5.54999000	5.42449300	-0.72939000
C	7.40989900	2.59830500	1.40814700	C	-10.20442100	-0.74374400	1.36337200
C	7.71940300	1.40607100	0.72953300	C	-10.24613900	0.70626700	0.78586700
C	7.84991700	0.18125400	1.40816000	C	-9.14480200	1.51031300	1.41384900
C	7.71940400	1.40607100	-0.72953000	C	-10.24613800	0.70626300	-0.78586200
C	7.84989700	0.18125500	-1.40814800	C	-9.14479200	1.51029600	-1.41384600
C	7.77608600	-1.04827900	-0.72953200	C	-8.15810600	2.18257800	-0.73806300
C	7.77608600	-1.04828200	0.72953200	C	-8.15811100	2.18258700	0.73806500
C	7.40992200	2.59831500	-1.40815300	C	-10.20442900	-0.74375100	-1.36335900
C	7.52196300	-2.25353700	-1.40815700	C	-7.12091800	2.88042000	-1.41007200
C	7.52194200	-2.25353200	1.40814300	C	-7.12092700	2.88043600	1.41007300
H	7.39534500	2.59320600	2.49560800	H	-10.07827200	-0.70549500	2.45049300
H	7.39542400	2.59323700	-2.49559800	H	-10.07828700	-0.70550900	-2.45048100
C	7.07192600	-3.40013200	0.72952500	C	-6.08178300	3.53504200	0.73300400
C	7.07192300	-3.40013200	-0.72952800	C	-6.08177900	3.53503500	-0.73300400
C	6.45782700	-4.46787700	-1.40814200	C	-5.02308100	4.15963600	-1.40748200
C	6.45785000	-4.46789400	1.40814800	C	-5.02308700	4.15964800	1.40748100
H	7.83445100	0.18090000	-2.49560800	H	-9.12835900	1.52106200	-2.50273200
H	7.83452400	0.18089800	2.49560400	H	-9.12837800	1.52109400	2.50273500
H	7.50716700	-2.24911200	2.49560500	H	-7.11884500	2.88075900	2.49776200
H	7.50724300	-2.24913200	-2.49560200	H	-7.11883000	2.88073100	-2.49776100
H	6.44510100	-4.45906200	-2.49560400	H	-5.02024100	4.15676200	-2.49520800
H	6.44517200	-4.45911500	2.49559400	H	-5.02025200	4.15678300	2.49520700
C	-6.45784900	4.46789300	1.40814800	C	5.58697900	-5.41427100	1.40834500
C	-5.67543500	5.41925500	0.72953100	C	4.44358000	-5.87256000	0.72992300
C	-4.76117900	6.24471400	1.40813800	C	3.24609700	-6.15639200	1.40842200
C	-5.67543500	5.41925300	-0.72953600	C	4.44358000	-5.87255800	-0.72993200

C	-4.76119200	6.24473200	-1.40815500	C	3.24609500	-6.15638900	-1.40843000
C	-3.72291300	6.90743800	-0.72953000	C	2.01590900	-6.25129700	-0.73044400
C	-3.72291600	6.90743800	0.72952400	C	2.01591000	-6.25129800	0.73043700
C	-7.07192500	3.40013200	0.72952500	C	6.62727700	-4.75410200	0.72948200
C	-6.45782700	4.46787700	-1.40814200	C	5.58697700	-5.41426800	-1.40835500
H	-4.75183100	6.23246300	-2.49560000	H	3.24404100	-6.14312400	-2.49591000
C	-2.59830500	7.40989700	-1.40814600	C	0.78936200	-6.19797700	-1.40864000
C	-2.59831500	7.40992100	1.40815300	C	0.78936400	-6.19797900	1.40863400
H	-2.59320600	7.39534300	-2.49560800	H	0.79043300	-6.18712200	-2.49615400
H	-2.59323700	7.39542200	2.49559800	H	0.79043600	-6.18712400	2.49614900
C	-7.07192300	3.40013200	-0.72952800	C	6.62727700	-4.75410100	-0.72949100
H	-6.44517200	4.45911500	2.49559400	H	5.57949700	-5.39966600	2.49576500
H	-4.75178200	6.23240000	2.49559900	H	3.24404400	-6.14312900	2.49590200
H	-6.44510000	4.45906100	-2.49560400	H	5.57949400	-5.39966100	-2.49577500
C	-4.46787700	-6.45782600	1.40814200	C	6.60119000	4.78129800	1.40847500
C	-7.40989900	-2.59830500	1.40814700	C	9.02285100	0.63458100	1.40867000
C	-7.40992300	-2.59831500	-1.40815300	C	9.02285100	0.63458300	-1.40867200
C	-7.71940500	-1.40607100	-0.72953000	C	9.01952000	-0.59695000	-0.72923800
C	-7.71940300	-1.40607100	0.72953300	C	9.01952000	-0.59695100	0.72923400
C	-4.46789400	-6.45784800	-1.40814800	C	6.60119100	4.78130000	-1.40847200
C	-7.84989700	-0.18125600	-1.40814800	C	8.76790300	-1.80337700	-1.40858800
C	-7.84991700	-0.18125400	1.40816000	C	8.76790400	-1.80337900	1.40858300
H	-4.45906100	-6.44509900	2.49560400	H	6.58991900	4.76660700	2.49584300
H	-4.45911500	-6.44517000	-2.49559400	H	6.58992200	4.76661100	-2.49584000
C	-7.77608600	1.04828200	0.72953200	C	8.25575500	-2.92318100	0.72927200
C	-7.77608700	1.04827900	-0.72953200	C	8.25575400	-2.92318000	-0.72927900
C	-7.52196300	2.25353700	-1.40815700	C	7.53664300	-3.92409500	-1.40844500
C	-7.52194200	2.25353200	1.40814300	C	7.53664400	-3.92409800	1.40843600
H	-7.39542500	-2.59323700	-2.49559800	H	9.00208300	0.63227700	-2.49596300
H	-7.39534600	-2.59320600	2.49560800	H	9.00208200	0.63227400	2.49596200
H	-7.83452500	-0.18089800	2.49560400	H	8.74830700	-1.79698900	2.49588900
H	-7.83445200	-0.18090000	-2.49560800	H	8.74830600	-1.79698500	-2.49589500
H	-7.50724300	2.24913200	-2.49560200	H	7.52237000	-3.91147000	-2.49579900
H	-7.50716700	2.24911200	2.49560500	H	7.52237300	-3.91147500	2.49579000
C	4.46789400	6.45784900	-1.40814800	C	-6.04792000	-3.54730400	-1.40507200
C	4.46787700	6.45782600	1.40814200	C	-6.04791500	-3.54729800	1.40507800
H	4.45911500	6.44517100	-2.49559400	H	-6.04510000	-3.54113300	-2.49280700
H	4.45906200	6.44509900	2.49560400	H	-6.04509000	-3.54112300	2.49281300
C	-6.90744000	-3.72291600	-0.72952400	C	8.74907000	1.83615900	-0.72931100
C	-6.24471600	-4.76117900	-1.40813800	C	8.24289000	2.95871100	-1.40864500
C	-5.41925600	-5.67543500	-0.72953100	C	7.50623500	3.94678800	-0.72940100
C	-5.41925400	-5.67543400	0.72953600	C	7.50623400	3.94678700	0.72940300
C	-6.24473300	-4.76119200	1.40815500	C	8.24288900	2.95870900	1.40864700
C	-6.90744000	-3.72291400	0.72953000	C	8.74906900	1.83615800	0.72931000
H	-6.23240200	-4.75178300	-2.49559900	H	8.22564000	2.94862300	-2.49596400
H	-6.23246400	-4.75183200	2.49560000	H	8.22563900	2.94861900	2.49596500
C	-1.40607100	7.71940100	-0.72953300	C	-0.43030400	-5.99138500	-0.73059000
C	-0.18125400	7.84991500	-1.40816000	C	-1.61287300	-5.67468800	-1.40857200
C	-0.18125500	7.84989500	1.40814800	C	-1.61287000	-5.67468700	1.40857100
C	-1.40607100	7.71940300	0.72953000	C	-0.43030300	-5.99138500	0.73058700
H	-0.18089800	7.83452300	-2.49560400	H	-1.61041400	-5.66551300	-2.49610900
H	-0.18090000	7.83445000	2.49560800	H	-1.61041000	-5.66551100	2.49610700
C	1.04828200	7.77608400	-0.72953200	C	-2.77530000	-5.24617700	-0.72955800
C	2.25353200	7.52194100	-1.40814300	C	-3.89127500	-4.74355700	-1.40744300
C	3.40013200	7.07192400	-0.72952500	C	-4.98445000	-4.17028000	-0.72685900
C	3.40013200	7.07192200	0.72952800	C	-4.98444700	-4.17027800	0.72686300
C	2.25353700	7.52196100	1.40815700	C	-3.89127100	-4.74355400	1.40744600
C	1.04827900	7.77608500	0.72953200	C	-2.77529800	-5.24617600	0.72955800
H	2.24911200	7.50716600	-2.49560500	H	-3.88844800	-4.73570200	-2.49503800
H	2.24913200	7.50724100	2.49560100	H	-3.88844100	-4.73569700	2.49504000

C	5.41925600	5.67543500	-0.72953100	C	-7.07952400	-2.89785500	-0.72400200
C	6.24471500	4.76117900	-1.40813800	C	-8.12281600	-2.19912700	-1.40617900
C	6.90743900	3.72291600	-0.72952400	C	-9.10515800	-1.54009200	-0.71856500
C	6.90743900	3.72291300	0.72953000	C	-9.10515300	-1.54008800	0.71857700
C	6.24473300	4.76119200	1.40815500	C	-8.12280800	-2.19911900	1.40618900
C	5.41925400	5.67543400	0.72953600	C	-7.07952000	-2.89785200	0.72401000
H	6.23240100	4.75178200	-2.49559900	H	-8.11319100	-2.18565100	-2.49395800
H	6.23246400	4.75183200	2.49560000	H	-8.11317700	-2.18563700	2.49396800
C	1.40607100	-7.71940100	-0.72953300	C	0.75155400	6.18948400	-0.72902100
C	2.59830500	-7.40989800	-1.40814600	C	-0.46614500	5.98886800	-1.40718600
C	3.72291400	-6.90743800	-0.72953000	C	-1.64915600	5.66358100	-0.72907800
C	3.72291600	-6.90743800	0.72952400	C	-1.64915800	5.66358400	0.72907700
C	2.59831500	-7.40992100	1.40815300	C	-0.46614700	5.98887100	1.40718500
C	1.40607100	-7.71940200	0.72953000	C	0.75155300	6.18948500	0.72902200
H	2.59320600	-7.39534400	-2.49560800	H	-0.46382400	5.97818500	-2.49478700
H	2.59323700	-7.39542300	2.49559800	H	-0.46382700	5.97819200	2.49478600
				H	-11.17548200	-1.22292000	-1.17451400
				H	-11.17547400	-1.22291600	1.17453800
				H	-11.20005900	1.13828100	-1.12198100
				H	-11.20006500	1.13827700	1.12198200

Table S8. Cartesian coordinates of the optimized geometries of [*n*+1]-acene and [*n*+1]-acene_H_4 at UB3LYP-D3(BJ)/6-31G(d) level of theory.

**UB3LYP-D3(BJ)/6-31G(d)**				**UB3LYP-D3(BJ)/6-31G(d)**			
**[7]-acene**				**[7]-acene _H_4**			
C	-8.57876800	0.71313500	-0.00001000	C	-5.91998900	2.66716000	0.79274400
C	-7.39785500	1.40726700	0.00002500	C	-4.94411500	1.98354800	1.46856100
C	-6.14286100	0.72442000	0.00001600	C	-4.12700100	1.01974000	0.80315300
C	-6.14284600	-0.72440900	0.00001200	C	-4.34899200	0.77732400	-0.60253100
C	-7.39782700	-1.40727800	-0.00003100	C	-5.37665500	1.51120600	-1.26933700
C	-8.57875200	-0.71317200	-0.00005300	C	-6.13926600	2.42767900	-0.59484800
C	-4.92393600	1.40599600	0.00001400	C	-3.12172500	0.30586300	1.46672900
C	-4.92391900	-1.40597500	0.00003000	C	-3.55254200	-0.16480600	-1.26490200
C	-3.69101100	-0.72799700	0.00003400	C	-2.54893000	-0.87716100	-0.60143900
C	-3.69102200	0.72802300	0.00001200	C	-2.32734100	-0.63524600	0.80465600
C	-2.45966800	1.40873700	-0.00000800	C	-1.29891100	-1.36998500	1.47082600
H	-2.46109700	2.49629400	-0.00001100	H	-1.13180100	-1.18570100	2.52985600
C	-1.23154100	0.73100300	-0.00000500	C	-0.53598400	-2.28894700	0.80437900
C	-1.23153300	-0.73099100	0.00002700	C	-0.74503300	-2.51691700	-0.59946500
C	-2.45966200	-1.40871700	0.00004400	C	-1.71831600	-1.82867400	-1.26956000
H	-4.92516900	2.49374600	0.00002600	H	-2.95376900	0.48898800	2.52586600
H	-9.52500100	1.24649500	-0.00000700	H	-6.53382500	3.39785700	1.31174000
H	-7.39457200	2.49436300	0.00007500	H	-4.77351000	2.16381400	2.52704100
H	-7.39452200	-2.49437400	-0.00006900	H	-5.54088100	1.32603600	-2.32801300
H	-9.52497700	-1.24654600	-0.00011300	H	-6.91752600	2.97884400	-1.11506600
H	-4.92514600	-2.49372500	0.00001700	H	-3.71836800	-0.34607100	-2.32478000
H	-2.46109900	-2.49627300	0.00004300	H	-1.86457000	-1.98701500	-2.33607700
C	0.00008600	1.40968400	-0.00002900	C	0.53698200	-3.11919200	1.45088700
C	1.23136400	0.73099700	-0.00002400	C	1.89235700	-3.07740100	0.66156700
C	1.23137200	-0.73100900	0.00000800	C	1.67191200	-2.98977900	-0.88548500
C	0.00008900	-1.40968400	0.00003100	C	0.23606600	-3.44846600	-1.25964400
C	2.45981500	1.40868800	-0.00004100	C	2.76179600	-1.98493300	1.21449100
C	2.45982200	-1.40870800	0.00000800	C	1.90748500	-1.62179100	-1.46312500
C	3.69092100	-0.72812100	-0.00001100	C	2.54661400	-0.59763100	-0.80931600
C	3.69091000	0.72809500	-0.00003200	C	3.03553600	-0.80466300	0.56686000
C	4.92396700	1.40607500	-0.00002700	C	3.76580700	0.24869100	1.18613600
H	4.92507300	2.49382200	-0.00001300	H	4.14090200	0.09420800	2.19546500
C	6.14286100	0.72449700	-0.00001200	C	3.99336900	1.46954700	0.55656900
C	6.14287700	-0.72450800	-0.00001900	C	3.47601300	1.68776100	-0.78622200
C	4.92398400	-1.40609600	-0.00001600	C	2.77780000	0.66545600	-1.42315700
H	2.46082200	2.49626100	-0.00004000	H	3.11363500	-2.11663200	2.23631300
H	-0.00013600	2.49722800	-0.00003300	H	0.70042400	-2.80785100	2.48767900
H	-0.00014800	-2.49722800	0.00003600	H	0.08774300	-4.48536700	-0.92637000
H	2.46081900	-2.49628100	0.00001000	H	1.55207900	-1.45537600	-2.47849900
H	4.92509500	-2.49384300	-0.00003100	H	2.40161200	0.82687900	-2.43084500
C	7.39789900	1.40726300	0.00003200	C	4.71429100	2.52987300	1.18814400
C	8.57873400	0.71324700	0.00005000	C	4.91736500	3.72703700	0.55176500
C	8.57874900	-0.71321000	0.00000600	C	4.40888700	3.94143100	-0.76441300
C	7.39792700	-1.40725200	-0.00002900	C	3.71184400	2.95242400	-1.40890400
H	7.39449300	2.49438400	0.00007100	H	5.09958700	2.36494400	2.19135400
H	9.52497700	1.24655000	0.00011100	H	5.46740800	4.52187900	1.04733500
H	9.52500100	-1.24649900	0.00000100	H	4.57830200	4.89669000	-1.25291600
H	7.39454300	-2.49437300	-0.00008100	H	3.32323600	3.11341400	-2.41141200
				H	0.19828600	-4.16445900	1.48701900
				H	0.11024600	-3.44076500	-2.34774400
				H	2.37889100	-3.68374200	-1.36757500
				H	2.38593800	-4.03547600	0.86890300

[8]-acene				[8]-acene _H_4			
C	-7.37520100	0.72390100	-0.00000400	C	4.45107400	2.08971900	-0.58360100
C	-6.15476100	1.40549300	-0.00004200	C	4.08665900	0.92239800	-1.26551300
C	-4.92386400	0.72723800	-0.00006000	C	3.47254400	-0.15067400	-0.61245600
C	-4.92386100	-0.72725300	-0.00008000	C	3.20663700	-0.05272800	0.80340900
C	-6.15476500	-1.40550400	-0.00005600	C	3.56970800	1.11294600	1.48502500
C	-7.37520100	-0.72390600	-0.00001800	C	4.18476900	2.18783100	0.83187100
C	-3.68950400	1.40806200	-0.00006500	C	3.08167800	-1.34063700	-1.30000600
C	-3.68950300	-1.40807600	-0.00008500	C	2.57794800	-1.15560800	1.45881000
C	-2.46465200	-0.73032000	-0.00007300	C	2.23530200	-2.28809800	0.77273300
C	-2.46465700	0.73030800	-0.00005100	C	2.48586400	-2.37929400	-0.63949300
C	-1.22918100	1.40934700	-0.00002500	C	1.95357600	-3.61559700	-1.31264300
H	-1.22990300	2.49685000	-0.00006700	H	2.51360500	-4.50920800	-1.00274900
C	-0.00014100	0.73156400	0.00001000	C	0.45978800	-3.78533200	-0.91908800
C	-0.00013600	-0.73156400	-0.00001200	C	0.30849900	-3.95452700	0.62979500
C	-1.22917600	-1.40935300	-0.00004700	C	1.59469000	-3.49349300	1.40031100
H	-3.69061600	2.49561400	-0.00010600	H	3.25470300	-1.40134800	-2.37249600
H	-6.15547800	2.49323000	-0.00008500	H	4.28553800	0.84915600	-2.33263900
H	-6.15549600	-2.49324200	-0.00001700	H	3.36804500	1.18711800	2.55148400
H	-3.69062200	-2.49562800	-0.00004700	H	2.37491900	-1.07937800	2.52483100
H	-1.22990200	-2.49685700	-0.00000800	H	2.31406700	-4.32464800	1.38753300
C	1.22936500	1.40931000	0.00004600	C	-0.32003600	-2.63702300	-1.49305500
C	2.46441700	0.73050400	0.00007300	C	-1.29296000	-1.92491900	-0.82403400
C	2.46442200	-0.73049300	0.00004900	C	-1.61883800	-2.27920200	0.57028800
C	1.22937000	-1.40930400	0.00002200	C	-0.89166300	-3.26406400	1.20635400
C	3.68960600	1.40809300	0.00008700	C	-2.00915100	-0.87163400	-1.43547800
C	3.68960800	-1.40807900	0.00006300	C	-2.66637400	-1.58234800	1.21364000
C	4.92385500	-0.72733600	0.00005900	C	-3.38444100	-0.54085400	0.58947300
C	4.92385300	0.72735100	0.00008100	C	-3.03888900	-0.16775400	-0.77676400
C	6.15473200	1.40552900	0.00005900	C	-3.74627700	0.87345000	-1.39210300
H	6.15558800	2.49326200	0.00002100	H	-3.48928500	1.15403700	-2.41116100
C	7.37533100	0.72387700	0.00001900	C	-4.77754100	1.56736800	-0.73466000
C	7.37533000	-0.72387100	0.00000300	C	-5.12061300	1.19703700	0.61761800
C	6.15472800	-1.40551800	0.00004000	C	-4.41320300	0.15325800	1.23997800
H	3.69053900	2.49565200	0.00005000	H	-1.76157800	-0.59939500	-2.45884000
H	1.22947000	2.49683600	0.00000700	H	-0.09252300	-2.36600800	-2.52261500
H	1.22947100	-2.49683000	0.00006300	H	-1.13830800	-3.50863500	2.23823700
H	3.69053300	-2.49563800	0.00010400	H	-2.91723900	-1.84907600	2.23779200
H	6.15557000	-2.49325000	0.00008200	H	-4.67218700	-0.12343500	2.25966000
C	8.62932200	1.40679400	-0.00003500	C	-5.49788900	2.62506700	-1.35627300
C	9.81092300	0.71271300	-0.00008200	C	-6.50241200	3.28606900	-0.68884100
C	9.81092000	-0.71273500	-0.00007500	C	-6.84002400	2.92162300	0.64132500
C	8.62931000	-1.40680000	-0.00002900	C	-6.16553800	1.90423700	1.27492100
H	8.62592900	2.49389700	-0.00007800	H	-5.23686000	2.90209700	-2.37465800
H	10.75695000	1.24637000	-0.00013300	H	-7.04283300	4.09172300	-1.17749600
H	10.75694100	-1.24640200	-0.00009000	H	-7.63546400	3.45196900	1.15700800
H	8.62589900	-2.49390100	0.00000700	H	-6.42152600	1.62303900	2.29347300
C	-9.81083800	0.71281300	0.00007500	C	5.42271200	4.32203000	-0.54597000
C	-8.62934300	1.40684200	0.00002800	C	5.07845600	3.19217400	-1.23966900
C	-8.62935500	-1.40683600	0.00003800	C	4.55952900	3.38332300	1.51730400
C	-9.81084100	-0.71279200	0.00008400	C	5.15963700	4.41896500	0.85130000
H	-10.75692700	1.24635800	0.00008800	H	5.89924500	5.15308700	-1.05816700
H	-8.62582500	2.49394500	-0.00001100	H	5.27729700	3.11477000	-2.30574000
H	-8.62585500	-2.49394100	0.00008200	H	4.35666300	3.45376900	2.58306500
H	-10.75693700	-1.24632600	0.00013600	H	5.43888700	5.32269200	1.38548100
				H	2.04823200	-3.54037300	-2.40128100
				H	1.35152300	-3.30626800	2.45115100
				H	0.20826700	-5.02817000	0.83712000

				H	0.09747700	-4.70977900	-1.39604800
**[9]-acene**				**[9]-acene _H_4**			
C	-8.60596500	-0.72381800	0.00000600	C	5.84832600	1.65047000	-0.62407600
C	-7.38545700	-1.40535200	0.00008800	C	5.09083500	0.68793800	-1.28555300
C	-6.15508500	-0.72698200	0.00013100	C	4.10994600	-0.07272600	-0.62036400
C	-6.15509100	0.72698800	0.00013300	C	3.88613300	0.15649300	0.79494900
C	-7.38547000	1.40535400	0.00007500	C	4.65705600	1.13220200	1.45599400
C	-8.60597200	0.72381500	0.00000600	C	5.62437800	1.87981300	0.79033100
C	-4.91976800	-1.40773900	0.00016000	C	3.33680100	-1.04868600	-1.28160300
C	-4.91977900	1.40774600	0.00015200	C	2.90394800	-0.60512000	1.46023000
C	-3.69673900	0.72993000	0.00015100	C	2.14699200	-1.56594000	0.79882500
C	-3.69673400	-0.72992300	0.00014400	C	2.37020200	-1.79452500	-0.61614300
C	-2.45872500	-1.40890300	0.00011100	C	1.56740300	-2.77568700	-1.28309600
H	-2.45937800	-2.49642600	0.00015300	H	1.71183100	-2.92284700	-2.35140500
C	-1.23292700	-0.73137100	0.00005200	C	0.62636500	-3.49999200	-0.61058100
C	-1.23293200	0.73137500	0.00006700	C	0.41777600	-3.28639200	0.80026000
C	-2.45873300	1.40890900	0.00011700	C	1.14734600	-2.34467000	1.46658100
H	-4.92075900	-2.49530400	0.00020600	H	3.50296700	-1.21876800	-2.34311400
H	-7.38609500	-2.49309000	0.00013400	H	5.25846400	0.51606900	-2.34655900
H	-7.38611800	2.49309200	0.00003100	H	4.48860700	1.30435800	2.51678700
H	-4.92077800	2.49531100	0.00011300	H	2.73576400	-0.43274500	2.52097000
H	-2.45939200	2.49643200	0.00007700	H	0.97978400	-2.17169600	2.52740300
C	0.00011400	-1.40907100	-0.00001400	C	-0.32656800	-4.46335700	-1.26558100
C	1.23261100	-0.73137100	-0.00007600	C	-1.77507200	-4.05336800	-0.88233200
C	1.23260700	0.73136900	-0.00005500	C	-1.98494100	-4.14304600	0.66536400
C	0.00010600	1.40907200	0.00000700	C	-0.62244200	-4.15837200	1.44392700
C	2.45887500	-1.40887400	-0.00013200	C	-2.05943800	-2.69734500	-1.46283400
C	2.45886600	1.40886900	-0.00010300	C	-2.86780300	-3.06685700	1.22370100
C	3.69651700	0.73004000	-0.00013600	C	-3.17448100	-1.88587000	0.57948500
C	3.69652200	-0.73004700	-0.00016100	C	-2.71395700	-1.67654900	-0.80625700
C	4.91973800	-1.40779500	-0.00017200	C	-2.97993000	-0.43353200	-1.42299600
H	4.92049800	-2.49536600	-0.00014600	H	-2.62859900	-0.27114300	-2.43929100
C	6.15516900	-0.72720700	-0.00014300	C	-3.69312700	0.59901300	-0.77894900
C	6.15516300	0.72720200	-0.00011800	C	-4.18112800	0.37818000	0.57690100
C	4.91972800	1.40778900	-0.00013900	C	-3.90811500	-0.85400100	1.20704100
H	2.45900400	-2.49640900	-0.00010300	H	-1.72773000	-2.52974300	-2.48604000
H	-0.00015900	-2.49661500	0.00001700	H	-0.20806200	-4.45063800	-2.35439100
H	-0.00017100	2.49661600	-0.00002800	H	-0.25364100	-5.19386400	1.46029400
H	2.45899000	2.49640500	-0.00013500	H	-3.20550900	-3.20337200	2.24969700
H	4.92048000	2.49536000	-0.00017200	H	-4.26039400	-1.00994700	2.22417000
C	7.38543900	-1.40531700	-0.00009600	C	-3.95539200	1.82756900	-1.39951600
C	8.60617200	-0.72376800	-0.00001400	C	-4.67401900	2.85125500	-0.75705000
C	8.60616500	0.72377000	0.00000900	C	-5.15866500	2.63198700	0.58481500
C	7.38542700	1.40531400	-0.00006300	C	-4.89749700	1.40109900	1.21237500
H	7.38631000	-2.49308100	-0.00007000	H	-3.59112100	1.99478700	-2.41074400
H	7.38628700	2.49307800	-0.00009000	H	-5.26211600	1.23827600	2.22426600
C	-11.04152800	-0.71282100	-0.00016800	C	7.57670800	3.36779500	-0.61827200
C	-9.86010000	-1.40683000	-0.00006900	C	6.84763900	2.42877700	-1.29247300
C	-9.86011300	1.40682300	-0.00009200	C	6.41250800	2.87432500	1.45438800
C	-11.04153400	0.71280800	-0.00018100	C	7.35581100	3.59401300	0.77612400
H	-11.98764700	-1.24629000	-0.00022300	H	8.33129400	3.95072800	-1.13865000
H	-9.85650900	-2.49392400	-0.00003100	H	7.01290500	2.25435800	-2.35274000
H	-9.85652700	2.49392000	-0.00013500	H	6.24171500	3.04387900	2.51455100
H	-11.98765900	1.24626800	-0.00027000	H	7.94597800	4.34528300	1.29311500
C	9.86009500	-1.40675700	0.00007300	C	-4.94186700	4.10001500	-1.38391600
C	11.04170000	-0.71267500	0.00017300	C	-5.64789200	5.08330400	-0.73119000
C	11.04169400	0.71268700	0.00018300	C	-6.12489200	4.86748200	0.58870100
C	9.86008300	1.40676300	0.00009600	C	-5.88517400	3.67310400	1.22696400

H	9.85673400	-2.49384900	0.00009800	H	-4.57500100	4.26248900	-2.39435300
H	11.98768000	-1.24640900	0.00025200	H	-5.84379800	6.03147300	-1.22363200
H	11.98766800	1.24643000	0.00024600	H	-6.68115400	5.65258900	1.09277200
H	9.85671500	2.49385300	0.00007500	H	-6.24881300	3.50492500	2.23767200
				H	-0.78613100	-3.86811400	2.48666400
				H	-0.14145100	-5.49493000	-0.93419400
				H	-2.45952100	-4.77417900	-1.35728700
				H	-2.45891300	-5.11002800	0.87901400
**[10]-acene**				**[10]-acene _H$_4$**			
C	9.83608500	-0.72393700	0.00001800	C	5.70941400	3.18756300	-0.60412200
C	8.61599100	-1.40540200	-0.00017700	C	5.32428900	2.03023600	-1.27495900
C	7.38542200	-0.72697300	-0.00030300	C	4.67249600	0.97035000	-0.61535100
C	7.38544100	0.72699500	-0.00028900	C	4.39684000	1.09238100	0.80411200
C	8.61602400	1.40541100	-0.00015300	C	4.78997000	2.26677400	1.47462100
C	9.83610500	0.72393000	0.00002000	C	5.43355400	3.30967600	0.81444200
C	6.15024000	-1.40765000	-0.00039200	C	4.27743300	-0.20504200	-1.28601500
C	6.15026600	1.40767800	-0.00035500	C	3.74432400	0.03113100	1.46384300
C	4.92774100	0.72977200	-0.00037800	C	3.35933900	-1.12455800	0.79317200
C	4.92772500	-0.72974100	-0.00039200	C	3.63415200	-1.24619300	-0.62596400
C	3.68880000	-1.40867800	-0.00036000	C	3.21368700	-2.43696100	-1.30217600
H	3.68930400	-2.49621900	-0.00039300	H	3.39054900	-2.51363300	-2.37298300
C	2.46493400	-0.73105900	-0.00025800	C	2.58748800	-3.44924500	-0.63464500
C	2.46494700	0.73108000	-0.00025100	C	2.33096600	-3.33627400	0.77978900
C	3.68882000	1.40870400	-0.00033200	C	2.69645100	-2.20796300	1.45532600
H	6.15115600	-2.49522700	-0.00042300	H	4.48201700	-0.29546700	-2.35058600
H	8.61661200	-2.49314400	-0.00020500	H	5.53082800	1.93867300	-2.33906700
H	8.61665300	2.49315300	-0.00012700	H	4.58253900	2.35835000	2.53851700
H	6.15118000	2.49525500	-0.00032500	H	3.53712800	0.12298300	2.52770400
H	3.68931200	2.49624500	-0.00030800	H	2.48929300	-2.11519700	2.51919900
C	1.22931000	-1.40871100	-0.00013800	C	2.02282300	-4.67710700	-1.29658200
C	0.00021100	-0.73131600	0.00000800	C	0.52442100	-4.80019900	-0.90466000
C	0.00022400	0.73131500	0.00000700	C	0.36597800	-4.95932200	0.64376300
C	1.22932600	1.40872000	-0.00012500	C	1.65968800	-4.52041200	1.41511900
C	-1.22959200	-1.40873300	0.00014700	C	-0.21980000	-3.63001000	-1.48166400
C	-1.22957700	1.40872100	0.00014700	C	-0.82087200	-4.24108000	1.21299300
C	-2.46461500	0.73102400	0.00026200	C	-1.51925100	-3.24057700	0.57405800
C	-2.46462800	-0.73104600	0.00026800	C	-1.17577900	-2.89373600	-0.81909900
C	-3.68883700	-1.40868900	0.00035600	C	-1.86094500	-1.82065000	-1.43325900
H	-3.68874800	-2.49624300	0.00033700	H	-1.59918000	-1.55413800	-2.45462700
C	-4.92763500	-0.73004800	0.00039400	C	-2.87701900	-1.08957600	-0.78293800
C	-4.92761900	0.73001600	0.00038500	C	-3.24346600	-1.45772600	0.58560100
C	-3.68881700	1.40866200	0.00034700	C	-2.55457800	-2.51828800	1.21057000
H	-1.22930000	-2.49629500	0.00013300	H	0.02096500	-3.36486100	-2.50979200
H	1.22960100	-2.49628500	-0.00015700	H	2.12202800	-4.61494000	-2.38561700
H	1.22960000	2.49629500	-0.00010200	H	1.41748100	-4.31817000	2.46334900
H	-1.22930000	2.49628300	0.00016800	H	-1.07971300	-4.48049800	2.24316300
H	-3.68873900	2.49621500	0.00036100	H	-2.81886600	-2.78073100	2.23248700
C	-6.15020000	-1.40767400	0.00038700	C	-3.54865400	-0.03398300	-1.40070600
C	-7.38560800	-0.72717200	0.00030600	C	-4.57574600	0.69514400	-0.75106700
C	-7.38558900	0.72715000	0.00029200	C	-4.94069000	0.32854000	0.60603000
C	-6.15017300	1.40764600	0.00037100	C	-4.25475500	-0.74357700	1.22938600
H	-6.15108700	-2.49527800	0.00038200	H	-3.27700500	0.24198700	-2.41706800
H	-6.15106100	2.49524900	0.00038800	H	-4.52824800	-1.01567600	2.24636500
C	12.27166900	-0.71298800	0.00047700	C	6.73917100	5.39565100	-0.58336000
C	11.09051400	-1.40696600	0.00024100	C	6.37245500	4.27036800	-1.26677000
C	11.09054600	1.40694300	0.00023400	C	5.83645000	4.50758100	1.48816200
C	12.27168800	0.71294500	0.00046700	C	6.46705400	5.51610700	0.81512500
H	13.21787000	-1.24630800	0.00066100	H	7.24132700	6.20897300	-1.09944600

H	11.08687700	-2.49406000	0.00023200	H	6.57764800	4.17560400	-2.33016500
H	11.08691700	2.49403800	0.00023500	H	5.62768500	4.59592100	2.55139800
H	13.21789800	1.24624700	0.00063900	H	6.76664800	6.41906100	1.33942700
C	-8.61593000	-1.40535400	0.00018000	C	-5.24892800	1.75363300	-1.37123100
C	-9.83639200	-0.72382400	-0.00001300	C	-6.26619300	2.47079600	-0.72079600
C	-9.83637200	0.72383300	-0.00004000	C	-6.62915200	2.10615100	0.62771800
C	-8.61589700	1.40534700	0.00014800	C	-5.95468300	1.04441800	1.25221200
H	-8.61694000	-2.49311200	0.00017800	H	-4.97604100	2.02960400	-2.38743800
H	-8.61689800	2.49310400	0.00015600	H	-6.22875100	0.77059400	2.26871700
C	-11.09046400	-1.40682000	-0.00022100	C	-6.95555800	3.54915500	-1.34594100
C	-12.27190300	-0.71272900	-0.00047700	C	-7.94678800	4.23244500	-0.68500300
C	-12.27188400	0.71277500	-0.00051600	C	-8.30435200	3.87320600	0.64313200
C	-11.09043000	1.40684600	-0.00028600	C	-7.66222500	2.83910800	1.27918800
H	-11.08718600	-2.49390600	-0.00020100	H	-6.67957300	3.82151000	-2.36161500
H	-13.21790100	-1.24643200	-0.00064500	H	-8.46340200	5.05271700	-1.17509200
H	-13.21787100	1.24649700	-0.00071800	H	-9.09025600	4.42291600	1.15313500
H	-11.08714400	2.49393100	-0.00030000	H	-7.93313800	2.56189700	2.29493200
				H	0.13410300	-5.71453300	-1.37892100
				H	0.24324000	-6.02962200	0.85604200
				H	2.55677000	-5.58304300	-0.97664200
				H	2.35987700	-5.36787700	1.41228900

**[11]-acene**				**[11]-acene _H_4**			
C	-11.06615500	0.72406400	0.00005800	C	7.09928300	2.78612600	0.77888400
C	-9.84645200	1.40549500	-0.00021100	C	6.15667200	2.01545900	1.44555700
C	-8.61554100	0.72703700	-0.00037700	C	5.40441800	1.01794900	0.78519900
C	-8.61555500	-0.72707000	-0.00034000	C	5.62966800	0.79769400	-0.63616500
C	-9.84648600	-1.40550800	-0.00012800	C	6.59231800	1.58956700	-1.30190200
C	-11.06617300	-0.72405400	0.00009900	C	7.32439200	2.56605300	-0.64047000
C	-7.38079100	1.40768100	-0.00051800	C	4.45129400	0.23575800	1.45167300
C	-7.38081500	-1.40772300	-0.00045100	C	4.88651600	-0.18990900	-1.29716100
C	-6.15815600	-0.72974300	-0.00052000	C	3.93412500	-0.97101000	-0.63080500
C	-6.15814500	0.72969400	-0.00054300	C	3.70920900	-0.75102100	0.79074300
C	-4.91925100	1.40862500	-0.00053500	C	2.74589000	-1.54389900	1.45667200
H	-4.91965300	2.49616900	-0.00056200	H	2.57744600	-1.37920000	2.51854400
C	-3.69599900	0.73085100	-0.00043900	C	2.01426700	-2.51917400	0.79551800
C	-3.69600500	-0.73089000	-0.00043500	C	2.23826700	-2.73830300	-0.62441800
C	-4.91926500	-1.40867100	-0.00049800	C	3.17975700	-1.96858500	-1.29115100
H	-7.38167900	2.49525700	-0.00056500	H	4.28274000	0.40060500	2.51344500
H	-9.84708000	2.49323400	-0.00025900	H	5.98800800	2.18022300	2.50744300
H	-9.84712600	-2.49324700	-0.00007800	H	6.76060100	1.42505200	-2.36390500
H	-7.38170300	-2.49530000	-0.00041300	H	5.05457100	-0.35414400	-2.35914500
H	-4.91965400	-2.49621600	-0.00046000	H	3.34593900	-2.13131700	-2.35376600
C	-2.45933600	1.40849600	-0.00031200	C	1.03673100	-3.32761000	1.46444000
C	-1.23226900	0.73099800	-0.00014700	C	0.33086900	-4.28539900	0.79827900
C	-1.23227200	-0.73101000	-0.00016500	C	0.53922400	-4.48935600	-0.61643900
C	-2.45934100	-1.40852200	-0.00031800	C	1.45693000	-3.73965500	-1.29062800
C	0.00016100	1.40837500	0.00004000	C	-0.68448700	-5.18624400	1.44134900
C	0.00015700	-1.40837100	-0.00000500	C	-0.39100700	-5.47626100	-1.26858900
C	1.23182600	-0.73094100	0.00017200	C	-1.84852300	-5.10501300	-0.87946900
C	1.23183100	0.73096000	0.00020600	C	-2.05037500	-5.19614600	0.66918800
C	2.45951400	1.40856300	0.00037400	C	-2.94748000	-4.13391100	1.23079100
H	2.45903200	2.49613100	0.00036500	H	-3.27402000	-4.27394400	2.26001200
C	3.69579200	0.73100900	0.00048400	C	-3.28331000	-2.96375300	0.58657400
C	3.69578500	-0.73096700	0.00044300	C	-2.84041600	-2.75084700	-0.80544300
C	2.45950700	-1.40853200	0.00031200	C	-2.17199200	-3.75901700	-1.46255600
H	-0.00012700	2.49596100	0.00004500	H	-0.29188000	-6.21303800	1.44862400
H	-2.45949400	2.49608500	-0.00032900	H	0.86903800	-3.16233000	2.52644900
H	-2.45947800	-2.49611100	-0.00030400	H	1.60031000	-3.87960900	-2.36001900

H	-0.00010700	-2.49595700	-0.00000100	H	-0.27720600	-5.45959400	-2.35782300
H	2.45904400	-2.49610000	0.00030300	H	-1.85482700	-3.58942400	-2.49014300
C	4.91922300	1.40860400	0.00058000	C	-4.03275100	-1.94388100	1.21661300
C	6.15812200	0.72997200	0.00057100	C	-4.33841700	-0.71915200	0.58688200
C	6.15811000	-0.72992100	0.00053300	C	-3.86613000	-0.49308100	-0.78006300
C	4.91920900	-1.40855600	0.00050900	C	-3.13874000	-1.51552000	-1.42443500
H	4.91923000	2.49618100	0.00059800	H	-4.37231100	-2.10382200	2.23747300
H	4.91922900	-2.49613300	0.00051000	H	-2.80039100	-1.35100100	-2.44481800
C	-13.50178000	0.71312300	0.00064900	C	8.78742600	4.54401100	0.76309200
C	-12.32083500	1.40708700	0.00032400	C	7.86630200	3.80008600	1.44259400
C	-12.32087200	-1.40705100	0.00040800	C	8.30289400	3.37333900	-1.30961900
C	-13.50179900	-0.71305600	0.00069000	C	9.00922900	4.32718200	-0.63514700
H	-14.44803100	1.24635100	0.00086100	H	9.36084600	5.30860800	1.27933800
H	-12.31720000	2.49417900	0.00027800	H	7.69538800	3.96225000	2.50386300
H	-12.31725300	-2.49414300	0.00044500	H	8.46878500	3.20637700	-2.37095100
H	-14.44806300	-1.24626300	0.00093700	H	9.74715000	4.93100000	-1.15555100
C	7.38068800	1.40772500	0.00052500	C	-5.06680300	0.28573700	1.22472200
C	8.61579900	0.72720500	0.00037400	C	-5.36524800	1.52075900	0.59690800
C	8.61578300	-0.72717300	0.00034500	C	-4.89485000	1.74590300	-0.75857100
C	7.38066300	-1.40768300	0.00046700	C	-4.15663300	0.72161600	-1.40228700
H	7.38169200	2.49531700	0.00053300	H	-5.41839700	0.11951600	2.24051200
H	7.38166600	-2.49527400	0.00047000	H	-3.80548900	0.89209500	-2.41745700
C	9.84636200	1.40543000	0.00017700	C	-6.09523100	2.52835300	1.23729100
C	11.06650400	0.72390800	-0.00009500	C	-6.38584200	3.74998800	0.60854600
C	11.06648400	-0.72392300	-0.00011800	C	-5.91796500	3.97394800	-0.73835000
C	9.84632600	-1.40542000	0.00013600	C	-5.18544100	2.96401700	-1.38303200
H	9.84747000	2.49318300	0.00017800	H	-6.44797800	2.36067200	2.25258600
H	9.84742000	-2.49317200	0.00013900	H	-4.83306100	3.13412000	-2.39801400
C	12.32077900	1.40690300	-0.00039400	C	-7.12813500	4.78003900	1.25419700
C	13.50203800	0.71281400	-0.00072600	C	-7.39687300	5.96509700	0.61414900
C	13.50201800	-0.71288600	-0.00075100	C	-6.93592200	6.18575300	-0.71239100
C	12.32073900	-1.40694600	-0.00043500	C	-6.21714100	5.21620100	-1.36779400
H	12.31752200	2.49398500	-0.00038500	H	-7.47901500	4.60811800	2.26874000
H	14.44807800	1.24644300	-0.00096600	H	-7.96413900	6.74131900	1.11973000
H	14.44804500	-1.24653700	-0.00100500	H	-7.15602200	7.12817400	-1.20566400
H	12.31746500	-2.49402700	-0.00043400	H	-5.86297700	5.38188200	-2.38221200
				H	-0.84972900	-4.90698000	2.48682100
				H	-0.17769800	-6.50289400	-0.93891000
				H	-2.51445100	-5.84548200	-1.35015300
				H	-2.50705400	-6.17056500	0.88710500
**[12]-acene**				**[12]-acene _H$_4$**			
C	9.84573700	0.72727600	0.00027400	C	-5.64981300	2.20288600	0.79577300
C	8.61132400	1.40777300	0.00042700	C	-4.97769100	1.16696000	1.45877000
C	7.38831600	0.72976000	0.00048600	C	-4.56342500	0.00681400	0.79267200
C	7.38831600	-0.72978400	0.00047800	C	-4.83864900	-0.12730600	-0.63071700
C	8.61133200	-1.40779500	0.00037000	C	-5.51036600	0.90737800	-1.29361100
C	9.84573900	-0.72729400	0.00023500	C	-5.92551100	2.06856400	-0.62741600
C	6.14985500	1.40860700	0.00051200	C	-3.88504900	-1.04250600	1.45512700
C	6.14985900	-1.40863000	0.00051600	C	-4.41595300	-1.30144200	-1.29628300
C	4.92649600	-0.73070500	0.00049500	C	-3.75158400	-2.32218300	-0.63288200
C	4.92649800	0.73068400	0.00047200	C	-3.47761300	-2.18867900	0.78892200
C	3.68993800	1.40850700	0.00038500	C	-2.79085800	-3.25740600	1.45438600
H	3.68991800	2.49606100	0.00037700	H	-2.58534000	-3.15632600	2.51780100
C	2.46325300	0.73076400	0.00028500	C	-2.40325300	-4.37935300	0.78332000
C	2.46324900	-0.73077200	0.00033300	C	-2.65795000	-4.50320200	-0.63303700
C	3.68993900	-1.40852200	0.00044300	C	-3.30486600	-3.50828700	-1.30415300
H	6.14998900	2.49615100	0.00055000	H	-3.67901700	-0.94214600	2.51840200
H	8.61197800	2.49534500	0.00047100	H	-4.77140300	1.26749300	2.52191700

H	8.61198500	-2.49536700	0.00031700	H	-5.71603000	0.80726200	-2.35695600
H	6.14998600	-2.49617400	0.00050400	H	-4.61935100	-1.40066400	-2.36025300
H	3.68990800	-2.49607600	0.00042400	H	-3.47995500	-3.59276800	-2.37464400
C	1.22992500	1.40811500	0.00014000	C	-1.70861800	-5.54775900	1.42241400
C	0.00018600	0.73065600	0.00001000	C	-0.40635500	-5.96334000	0.65291600
C	0.00018200	-0.73064600	0.00007400	C	-0.56692300	-5.81162700	-0.89596600
C	1.22992300	-1.40811400	0.00023900	C	-2.06708500	-5.72153900	-1.28954800
C	-1.23017800	1.40813300	-0.00016500	C	0.76583100	-5.21991200	1.22054800
C	-1.23017800	-1.40811500	-0.00004700	C	0.15288700	-4.62700100	-1.47499300
C	-2.46299200	-0.73071200	-0.00021500	C	1.09189200	-3.87209300	-0.81448500
C	-2.46298900	0.73073600	-0.00028300	C	1.44316800	-4.20990200	0.57950600
C	-3.68996800	1.40849400	-0.00042900	C	2.46729600	-3.46510500	1.21337800
H	-3.68943000	2.49605700	-0.00044900	H	2.73796900	-3.72163400	2.23516500
C	-4.92633400	0.73093100	-0.00048300	C	3.13289100	-2.39517900	0.58727500
C	-4.92633400	-0.73089800	-0.00042100	C	2.75677100	-2.03507100	-0.78363800
C	-3.68996500	-1.40846400	-0.00031500	C	1.75671000	-2.78422100	-1.43038400
H	-1.22977400	2.49572500	-0.00017200	H	1.02955300	-5.45228300	2.25113100
H	1.23005200	2.49572100	0.00014600	H	-1.47101300	-5.33724900	2.47006500
H	1.23003700	-2.49572000	0.00026700	H	-2.58224900	-6.63717500	-0.96649200
H	-1.22978700	-2.49570800	-0.00001800	H	-0.09381400	-4.36855500	-2.50345800
H	-3.68943800	-2.49602700	-0.00031200	H	1.48794100	-2.52418400	-2.45164400
C	-6.14981200	1.40862200	-0.00053300	C	4.13238800	-1.66021000	1.22821200
C	-7.38839800	0.72999700	-0.00049000	C	4.79903600	-0.57552100	0.60459100
C	-7.38839600	-0.72996700	-0.00044700	C	4.42324500	-0.21573500	-0.75852100
C	-6.14980300	-1.40859000	-0.00043700	C	3.40820600	-0.96667600	-1.40329500
H	-6.14979800	2.49619100	-0.00053400	H	4.41227100	-1.92641400	2.24501300
H	-6.14979400	-2.49615800	-0.00042100	H	3.12926700	-0.69752900	-2.41951400
C	12.29641000	0.72417400	-0.00013600	C	-6.73631600	4.39799900	0.79814600
C	11.07698400	1.40551000	0.00012200	C	-6.07067500	3.37626600	1.46137800
C	11.07699700	-1.40552000	0.00001400	C	-6.60393800	3.11652200	-1.28960400
C	12.29641400	-0.72417400	-0.00019500	C	-7.01187800	4.26377000	-0.62302400
H	11.07764400	2.49327400	0.00019800	H	-5.86424100	3.47672300	2.52462100
H	11.07766100	-2.49328400	-0.00005100	H	-6.80993900	3.01618900	-2.35296200
C	-8.61124800	1.40776700	-0.00042300	C	5.79619600	0.15477300	1.24746800
C	-9.84600700	0.72726600	-0.00028200	C	6.45947900	1.23724400	0.62185100
C	-9.84600200	-0.72725200	-0.00027400	C	6.08515100	1.59568500	-0.73436500
C	-8.61123400	-1.40774400	-0.00038000	C	5.07123900	0.84911500	-1.38070300
H	-8.61227400	2.49535000	-0.00040800	H	6.07714700	-0.11264900	2.26364000
H	-8.61225700	-2.49532700	-0.00038700	H	4.79155900	1.11881600	-2.39658900
C	-11.07682600	1.40548300	-0.00009500	C	7.46365500	1.97357800	1.26567300
C	-12.29672000	0.72398500	0.00011700	C	8.11518400	3.04344300	0.63888200
C	-12.29671200	-0.72400200	0.00009500	C	7.74244500	3.40038700	-0.71112100
C	-11.07680600	-1.40548300	-0.00012100	C	6.73940500	2.66721800	-1.35792900
H	-11.07794400	2.49322800	-0.00005600	H	7.74457200	1.70554300	2.28182000
H	-11.07791500	-2.49322800	-0.00015900	H	6.45929400	2.93657700	-2.37393000
C	-13.55113600	1.40697300	0.00039000	C	9.13820600	3.79734800	1.28727800
C	-14.73229100	0.71289600	0.00063200	C	9.75911100	4.84010400	0.64866200
C	-14.73228100	-0.71295100	0.00058900	C	9.39183900	5.19181800	-0.68141300
C	-13.55111300	-1.40700900	0.00031500	C	8.41301200	4.49184600	-1.33916000
H	-13.54784800	2.49404500	0.00043600	H	9.41613000	3.52625000	2.30274300
H	-15.67836100	1.24644600	0.00085200	H	10.53639400	5.40495500	1.15531300
H	-15.67834300	-1.24651500	0.00075900	H	9.89353300	6.02058100	-1.17271000
H	-13.54781400	-2.49408100	0.00026400	H	8.13012900	4.75783200	-2.35459700
C	14.73212400	0.71314000	-0.00066300	C	-7.81949600	6.58130900	0.79100100
C	13.55124900	1.40713400	-0.00036000	C	-7.16636300	5.59146700	1.46719600
C	13.55126300	-1.40712200	-0.00050300	C	-7.70083100	5.33118900	-1.28854200
C	14.73212900	-0.71311400	-0.00074000	C	-8.09102900	6.44905200	-0.60902000
H	15.67833700	1.24642700	-0.00082900	H	-8.13910200	7.47969100	1.31127400
H	13.54771900	2.49421400	-0.00028600	H	-6.95852900	5.68879100	2.52980200
H	13.54773900	-2.49420200	-0.00057600	H	-7.90530300	5.22779100	-2.35123600

H	15.67834800	-1.24639200	-0.00098400	H	-8.61200600	7.24937800	-1.12670800
				H	-2.39191400	-6.40890700	1.42214100
				H	-2.16671000	-5.66566600	-2.37888200
				H	-0.26235900	-7.03032000	0.86831600
				H	-0.15643400	-6.71827100	-1.36780800
**[13]-acene**				**[13]-acene _H_4**			
C	8.61874000	0.73005700	0.00015700	C	5.06760700	0.64810300	-0.76251200
C	7.38057300	1.40871300	0.00020400	C	4.31496000	-0.36710400	-1.40498100
C	6.15676600	0.73075500	0.00025700	C	4.00020600	-1.57628100	-0.78158600
C	6.15675400	-0.73071300	0.00032500	C	4.46299100	-1.80740900	0.59061400
C	7.38055900	-1.40867900	0.00028700	C	5.20606000	-0.81229300	1.22902800
C	8.61872600	-0.73003200	0.00019200	C	5.52998900	0.41721300	0.60189800
C	4.92069300	1.40849600	0.00024600	C	3.26017800	-2.58484200	-1.42555500
C	4.92068500	-1.40845200	0.00039200	C	4.13460100	-3.02192000	1.22063000
C	3.69378500	-0.73054300	0.00036200	C	3.36904400	-4.03266900	0.59000400
C	3.69379400	0.73058700	0.00026800	C	2.93647400	-3.81573700	-0.80515900
C	2.46062400	1.40811600	0.00021100	C	2.25512800	-4.81151600	-1.46241300
H	2.46055900	2.49570100	0.00016300	H	1.94608400	-4.63919400	-2.49207600
C	1.23118700	0.73039500	0.00022400	C	1.90313600	-6.14974400	-0.87759900
C	1.23118000	-0.73035600	0.00032500	C	2.10014700	-6.24245900	0.67180800
C	2.46062100	-1.40807400	0.00039300	C	3.01083300	-5.19253800	1.23512500
H	4.92036100	2.49606200	0.00025400	H	2.92885700	-2.41728300	-2.44779400
H	7.38037500	2.49626300	0.00022300	H	3.97112700	-0.19371100	-2.42216100
H	7.38035500	-2.49622900	0.00027200	H	5.54989800	-0.98258500	2.24680000
H	4.92035300	-2.49601900	0.00043800	H	4.46703400	-3.18571500	2.24326600
H	2.46056000	-2.49565900	0.00042300	H	3.33001800	-5.33554000	2.26629100
C	-0.00008300	1.40785300	0.00016700	C	0.43917700	-6.49177900	-1.26965200
C	-1.23096500	0.73040600	0.00015900	C	-0.47288600	-5.48731200	-0.61885500
C	-1.23097000	-0.73037700	0.00025100	C	-0.26349800	-5.28952000	0.79788500
C	-0.00008300	-1.40781900	0.00034300	C	0.73307800	-6.21111400	1.44069800
C	-2.46068900	1.40804600	0.00007900	C	-1.37348800	-4.71912500	-1.29388900
C	-2.46068700	-1.40802600	0.00022800	C	-0.95091500	-4.31935100	1.46394800
C	-3.69358800	-0.73064700	0.00011600	C	-1.91142400	-3.48969200	0.79443100
C	-3.69358300	0.73065900	0.00004400	C	-2.13709500	-3.70260900	-0.62812700
C	-4.92066400	1.40851400	-0.00004000	C	-3.06025700	-2.91482100	-1.29558600
H	-4.92011800	2.49607900	-0.00006600	H	-3.22736200	-3.07277700	-2.35875500
C	-6.15676600	0.73093700	-0.00006300	C	-3.79790000	-1.90120000	-0.63583800
C	-6.15677200	-0.73094100	-0.00002800	C	-3.57111200	-1.68732300	0.78905900
C	-4.92066100	-1.40850900	0.00005600	C	-2.62339600	-2.50244500	1.45539500
H	-2.46018300	2.49565000	0.00009800	H	-1.51711800	-4.85457400	-2.36381400
H	0.00016300	2.49548000	0.00016900	H	0.32791800	-6.47240700	-2.35909600
H	0.00016900	-2.49544600	0.00040600	H	0.32198300	-7.23065500	1.44351400
H	-2.46017200	-2.49562900	0.00028800	H	-0.78218100	-4.15903300	2.52654200
H	-4.92010600	-2.49607500	0.00003800	H	-2.45384900	-2.34270700	2.51782800
C	-7.38052200	1.40865300	-0.00006900	C	-4.73012100	-1.10341700	-1.30290200
C	-8.61883800	0.73004400	-0.00009000	C	-5.45868000	-0.09781900	-0.64274200
C	-8.61884300	-0.73006000	-0.00010000	C	-5.23130800	0.11649900	0.78318000
C	-7.38052100	-1.40866300	-0.00005700	C	-4.29165100	-0.68996400	1.44979700
H	-7.38052200	2.49622600	-0.00000500	H	-4.89915300	-1.26290800	-2.36540600
H	-7.38051500	-2.49623500	-0.00005400	H	-4.12187900	-0.53010100	2.51208900
C	11.07632000	0.72737600	-0.00001800	C	6.13086800	2.86457500	-0.74356800
C	9.84200700	1.40778800	0.00008100	C	5.37895900	1.85340500	-1.38797600
C	9.84198700	-1.40777700	0.00009800	C	6.27103200	1.40774400	1.24271600
C	11.07630300	-0.72737900	-0.00002500	C	6.59146300	2.63456200	0.61394900
H	9.84280000	2.49538800	0.00012700	H	5.03423000	2.02709800	-2.40482000
H	9.84276700	-2.49537700	0.00008100	H	6.61621400	1.23663800	2.25988200
C	-9.84189800	1.40778800	-0.00007500	C	-6.39795000	0.70770200	-1.30919000
C	-11.07650900	0.72730700	-0.00010700	C	-7.11913800	1.70582100	-0.64830500

C	-11.07651500	-0.72732700	-0.00015800	C	-6.89174600	1.92006900	0.77637600
C	-9.84189900	-1.40780600	-0.00014800	C	-5.95875500	1.12155300	1.44342100
H	-9.84295200	2.49537600	0.00001200	H	-6.56749800	0.54824500	-2.37160000
H	-9.84295300	-2.49539500	-0.00018800	H	-5.78911300	1.28159800	2.50570800
C	-12.30743000	1.40549700	-0.00007900	C	-8.06647300	2.52005200	-1.31435400
C	-13.52725700	0.72402200	-0.00012300	C	-8.77860200	3.50842300	-0.65299500
C	-13.52726400	-0.72404300	-0.00020900	C	-8.55156500	3.72228500	0.76900700
C	-12.30743300	-1.40551800	-0.00022500	C	-7.62733100	2.93369600	1.43629900
H	-12.30855400	2.49324800	0.00002500	H	-8.23608400	2.36041000	-2.37685600
H	-12.30855900	-2.49327000	-0.00030800	H	-7.45758000	3.09361700	2.49872500
C	-14.78172600	1.40700400	-0.00007500	C	-9.74034100	4.33705300	-1.32259000
C	-15.96284800	0.71294100	-0.00013600	C	-10.42813600	5.30318400	-0.64806300
C	-15.96285300	-0.71296200	-0.00025500	C	-10.20456000	5.51377100	0.75217900
C	-14.78173000	-1.40702400	-0.00029200	C	-9.30043000	4.75136300	1.43242000
H	-14.77840000	2.49408000	0.00004100	H	-9.90756900	4.17498500	-2.38444900
H	-16.90893800	1.24645800	-0.00009000	H	-11.15295000	5.92259600	-1.16852100
H	-16.90894200	-1.24647900	-0.00033700	H	-10.76388200	6.28903800	1.26794500
H	-14.77840800	-2.49410000	-0.00040300	H	-9.12850200	4.90865400	2.49424000
C	13.52710300	0.72410500	-0.00026300	C	7.19025900	5.07395800	-0.72496700
C	12.30752000	1.40552300	-0.00011400	C	6.44547800	4.07845400	-1.37012500
C	12.30749500	-1.40554600	-0.00018500	C	7.33667600	3.63330200	1.25600500
C	13.52708500	-0.72414700	-0.00031400	C	7.64890900	4.84491500	0.62633700
H	12.30847500	2.49328100	-0.00004800	H	6.10044900	4.25167000	-2.38709100
H	12.30843500	-2.49330300	-0.00025100	H	7.68201300	3.46161900	2.27314700
C	15.96276100	0.71298700	-0.00054100	C	8.24520500	7.26872900	-0.69957900
C	14.78175600	1.40704100	-0.00036100	C	7.51422100	6.31185400	-1.35587800
C	14.78173000	-1.40710400	-0.00051900	C	8.40658500	5.86618400	1.27309800
C	15.96274500	-0.71307000	-0.00063400	C	8.69714000	7.04303700	0.63178000
H	16.90889800	1.24641800	-0.00059900	H	8.48396700	8.20650500	-1.19301700
H	14.77840200	2.49412000	-0.00027200	H	7.16752800	6.48101600	-2.37227700
H	14.77835700	-2.49418200	-0.00061100	H	8.74996900	5.69069200	2.28956000
H	16.90887300	-1.24651700	-0.00080400	H	9.27502700	7.81145700	1.13724300
				H	0.90070600	-5.93833300	2.48750600
				H	0.20502800	-7.51414000	-0.94093800
				H	2.54216500	-7.22309800	0.89200200
				H	2.55462400	-6.90410000	-1.34619900
**[14]-acene**				**[14]-acene _H_4**			
C	7.38709000	0.73083800	-0.00009900	C	4.95716300	-1.20561000	-0.63179500
C	6.15106800	1.40855100	-0.00008800	C	4.52548000	-2.38159100	-1.29324000
C	4.92411200	0.73065900	-0.00002800	C	3.84720200	-3.38990200	-0.62879200
C	4.92410700	-0.73053500	0.00005100	C	3.56522100	-3.24573800	0.79233500
C	6.15104700	-1.40841700	0.00001400	C	3.97949000	-2.10216600	1.45496200
C	7.38707500	-0.73069800	-0.00005900	C	4.67374600	-1.06069000	0.79163900
C	3.69108600	1.40808900	-0.00001900	C	3.39055000	-4.57526600	-1.29661200
C	3.69108100	-1.40798900	0.00014800	C	2.86229500	-4.30461400	1.45862000
C	2.46165900	-0.73014400	0.00016900	C	2.46650200	-5.42479700	0.79081500
C	2.46165900	0.73021800	0.00006200	C	2.72816100	-5.55846300	-0.62476600
C	1.23045700	1.40775700	0.00006200	C	2.12391900	-6.77138200	-1.27915900
H	1.23026100	2.49535800	0.00002500	H	2.62450000	-7.69297700	-0.95025500
C	0.00001100	0.72998000	0.00015000	C	0.62101700	-6.83783600	-0.89170200
C	0.00001400	-0.72997300	0.00027100	C	0.45270200	-6.98744100	0.65644300
C	1.23046000	-1.40771600	0.00027000	C	1.75553700	-6.58261900	1.43108900
H	3.69085600	2.49567700	-0.00003400	H	3.57058100	-4.66680200	-2.36570000
H	6.15100400	2.49611600	-0.00008600	H	4.73438800	-2.48866600	-2.35536000
H	6.15098700	-2.49598100	0.00001600	H	3.76770600	-1.99401900	2.51632000
H	3.69087000	-2.49557600	0.00018200	H	2.65160500	-4.19604000	2.52026800
H	1.23029200	-2.49531700	0.00031500	H	2.42939200	-7.45112700	1.43806800
C	-1.23045000	1.40773200	0.00013900	C	-0.07699600	-5.64040900	-1.47170600

C	-2.46167100	0.73016500	0.00021700	C	-1.00697500	-4.87470100	-0.81437500
C	-2.46167000	-0.73022600	0.00033500	C	-1.37282500	-5.21242900	0.57613100
C	-1.23044600	-1.40775900	0.00035800	C	-0.71470600	-6.23195800	1.21851500
C	-3.69108500	1.40799400	0.00019300	C	-1.65161900	-3.77209000	-1.42996800
C	-3.69108900	-1.40808200	0.00039200	C	-2.39245700	-4.45387500	1.20530600
C	-4.92410000	-0.73068100	0.00034500	C	-3.03414100	-3.37341500	0.57970800
C	-4.92409600	0.73056700	0.00024500	C	-2.64174600	-3.01252500	-0.78720700
C	-6.15104200	1.40842400	0.00020800	C	-3.27263600	-1.92788100	-1.40568600
H	-6.15100000	2.49599000	0.00018900	H	-2.98121200	-1.65829700	-2.41830700
C	-7.38708800	0.73071000	0.00022100	C	-4.27722400	-1.16467400	-0.76544800
C	-7.38710100	-0.73084500	0.00029000	C	-4.67027400	-1.52612600	0.59526700
C	-6.15106100	-1.40855000	0.00035900	C	-4.02836100	-2.62305600	1.21667500
H	-3.69081600	2.49558800	0.00016000	H	-1.37100200	-3.51159700	-2.44794500
H	-1.23034000	2.49534000	0.00010000	H	0.18028500	-5.38221900	-2.49764300
H	-1.23030800	-2.49536700	0.00041000	H	-0.98827300	-6.46395500	2.24661400
H	-3.69079900	-2.49567600	0.00043500	H	-2.67475900	-4.71097300	2.22382600
H	-6.15101200	-2.49611600	0.00037900	H	-4.32016200	-2.89015500	2.22991500
C	-8.61076300	1.40862200	0.00017000	C	-4.90190900	-0.08448400	-1.38587500
C	-9.84900300	0.72978200	0.00013200	C	-5.90843300	0.67932500	-0.74441500
C	-9.84902600	-0.72989500	0.00016100	C	-6.30124100	0.31806900	0.61251600
C	-8.61080200	-1.40874800	0.00025200	C	-5.65972400	-0.78161500	1.23462600
H	-8.61113000	2.49617400	0.00017900	H	-4.60935000	0.18572400	-2.39798000
H	-8.61118200	-2.49630000	0.00024900	H	-5.95270000	-1.05026900	2.24705900
C	9.84900500	0.72989900	-0.00013100	C	6.06562800	0.98106400	-0.63432400
C	8.61081900	1.40874400	-0.00012100	C	5.64102200	-0.18502200	-1.29597200
C	8.61077800	-1.40861500	-0.00010100	C	5.09303000	0.09522000	1.45388200
C	9.84898000	-0.72978500	-0.00013900	C	5.78145500	1.12634600	0.79010500
H	8.61115400	2.49629800	-0.00008400	H	5.85229000	-0.29314200	-2.35738700
H	8.61109700	-2.49616800	-0.00012400	H	4.88091300	0.20351500	2.51507100
C	-11.07209200	1.40776100	0.00006400	C	-6.53317200	1.76083900	-1.36569000
C	-12.30641400	0.72708500	-0.00002700	C	-7.53601100	2.51911000	-0.72264400
C	-12.30644600	-0.72712800	-0.00004600	C	-7.92768600	2.15889700	0.62912200
C	-11.07215000	-1.40784100	0.00006200	C	-7.29156400	1.06324400	1.25283400
H	-11.07308700	2.49533200	0.00010000	H	-6.24051200	2.03136800	-2.37767400
H	-11.07318200	-2.49541300	0.00002200	H	-7.58516500	0.79438500	2.26501000
C	-13.53751500	1.40554800	-0.00009800	C	-8.16720600	3.60865400	-1.34444800
C	-14.75710300	0.72400600	-0.00024000	C	-9.15927800	4.35423100	-0.70085600
C	-14.75713900	-0.72394100	-0.00031300	C	-9.54957100	3.99530200	0.64558600
C	-13.53758800	-1.40553800	-0.00020200	C	-8.92495700	2.91174500	1.27012000
H	-13.53824400	2.49325700	-0.00002000	H	-7.87415600	3.87899600	-2.35650900
H	-13.53837300	-2.49324800	-0.00028000	H	-9.21861700	2.64244800	2.28229600
C	-16.01169700	1.40718300	-0.00030200	C	-9.80555200	5.46354600	-1.32670100
C	-17.19272800	0.71315400	-0.00048900	C	-10.77499300	6.17661400	-0.67190300
C	-17.19276400	-0.71297500	-0.00062200	C	-11.15957200	5.82294500	0.65461600
C	-16.01177100	-1.40705900	-0.00052200	C	-10.56444100	4.76565300	1.29100000
H	-16.00822400	2.49424100	-0.00016800	H	-9.50989900	5.73063000	-2.33818400
H	-18.13898300	1.24639500	-0.00052800	H	-11.25757800	7.01798600	-1.16088300
H	-18.13904200	-1.24617500	-0.00081000	H	-11.93001200	6.39960400	1.15839400
H	-16.00835400	-2.49411800	-0.00063900	H	-10.85530200	4.49336400	2.30249500
C	12.30642800	0.72713400	-0.00012100	C	7.17293900	3.16829700	-0.63577800
C	11.07216900	1.40783900	-0.00010800	C	6.75380500	2.01118400	-1.29799300
C	11.07210700	-1.40776100	-0.00018800	C	6.20487300	2.29177200	1.45172900
C	12.30639400	-0.72709700	-0.00018300	C	6.88871300	3.31357600	0.78739200
H	11.07316700	2.49541200	-0.00003300	H	6.96561600	1.90317500	-2.35929200
H	11.07306600	-2.49533300	-0.00024700	H	5.99278100	2.40033500	2.51289500
C	14.75713000	0.72394300	-0.00007200	C	8.27901500	5.35455400	-0.63655000
C	13.53760100	1.40553600	-0.00005300	C	7.86622700	4.20912800	-1.29912000
C	13.53752400	-1.40555500	-0.00024300	C	7.31731300	4.48963900	1.44861300
C	14.75709300	-0.72402200	-0.00019100	C	7.99520600	5.49960000	0.78393400
H	13.53837300	2.49324600	0.00005900	H	8.07819500	4.10091100	-2.36049700

H	13.53823700	-2.49326500	-0.00035300	H	7.10511600	4.59809300	2.50990900
C	17.19276200	0.71297000	0.00001000	C	9.37731800	7.53073000	-0.62782400
C	16.01177800	1.40705400	0.00004200	C	8.98209500	6.41324500	-1.30345400
C	16.01170000	-1.40719600	-0.00026600	C	8.43217600	6.69424400	1.44862000
C	17.19272400	-0.71317100	-0.00016800	C	9.09782300	7.67356800	0.77092000
H	18.13904500	1.24616200	0.00012600	H	9.90862100	8.32373700	-1.14624200
H	16.00836000	2.49411300	0.00019200	H	9.19246700	6.30201700	-2.36418200
H	16.00822400	-2.49425400	-0.00043200	H	8.21858000	6.79959900	2.50929800
H	18.13898100	-1.24640800	-0.00023200	H	9.42224800	8.57227500	1.28760500
				H	1.51487800	-6.36488500	2.47655400
				H	2.22934700	-6.72096700	-2.36819500
				H	0.19709400	-7.73705700	-1.36579200
				H	0.29797600	-8.05281400	0.87176600
**[15]-acene**				**[15]-acene _H_4**			
C	-8.61757200	0.73086300	0.00103400	C	5.33926000	-1.00989800	-0.64697200
C	-7.38174400	1.40858300	0.00114200	C	4.62276600	-2.02785900	-1.30670700
C	-6.15449800	0.73068100	0.00111500	C	3.70432100	-2.83773300	-0.63936900
C	-6.15448300	-0.73053300	0.00103100	C	3.47499600	-2.62738900	0.78718300
C	-7.38171200	-1.40843700	0.00100900	C	4.17972600	-1.62153200	1.44778700
C	-8.61754900	-0.73072400	0.00097900	C	5.10933500	-0.79911900	0.78131000
C	-4.92180800	1.40813900	0.00110300	C	2.98057900	-3.86337700	-1.29861000
C	-4.92179200	-1.40800300	0.00092700	C	2.53899000	-3.45794400	1.45365700
C	-3.69201900	-0.73008600	0.00084900	C	1.84258500	-4.45507200	0.79303300
C	-3.69202600	0.73020700	0.00094500	C	2.07085900	-4.66454600	-0.63070100
C	-2.46114200	1.40778100	0.00083000	C	1.32263200	-5.69356500	-1.29577000
H	-2.46092200	2.49537800	0.00086700	H	1.46777300	-5.82683000	-2.36576100
C	-1.23057600	0.72986800	0.00059700	C	0.43535600	-6.47590200	-0.62009700
C	-1.23057400	-0.72979700	0.00050500	C	0.22362200	-6.28130100	0.79748700
C	-2.46113800	-1.40768400	0.00064900	C	0.89537100	-5.30035800	1.46297600
H	-4.92156700	2.49572000	0.00115400	H	3.14945300	-4.01870400	-2.36187500
H	-7.38166000	2.49614300	0.00116400	H	4.79345500	-2.18462500	-2.36932700
H	-7.38162100	-2.49599700	0.00098900	H	4.00826600	-1.46461800	2.51022400
H	-4.92156200	-2.49558400	0.00090500	H	2.36771700	-3.30107300	2.51622900
H	-2.46093900	-2.49528000	0.00061000	H	0.72490200	-5.14287100	2.52571000
C	0.00000400	1.40764900	0.00042800	C	-0.46012000	-7.49581400	-1.26988000
C	1.23053200	0.72987300	0.00016900	C	-1.92928600	-7.17903600	-0.87655300
C	1.23053200	-0.72986400	0.00008400	C	-2.12332100	-7.27510100	0.67316200
C	0.00000300	-1.40760900	0.00026100	C	-0.75667300	-7.21956500	1.44114400
C	2.46117400	1.40773200	-0.00001500	C	-2.30494500	-5.84689600	-1.46106600
C	2.46117400	-1.40775200	-0.00016400	C	-3.05214100	-6.24123400	1.23713200
C	3.69206100	-0.73020900	-0.00032800	C	-3.43196200	-5.09048600	0.59166700
C	3.69205900	0.73015900	-0.00025800	C	-3.00352700	-4.86555000	-0.80375000
C	4.92181400	1.40802400	-0.00041800	C	-3.35086900	-3.63880700	-1.42401600
H	4.92165800	2.49560800	-0.00040800	H	-3.02201300	-3.46493700	-2.44602400
C	6.15446600	0.73058600	-0.00059100	C	-4.10871300	-2.64772200	-0.78079500
C	6.15447400	-0.73068000	-0.00063300	C	-4.56773000	-2.88770600	0.59194100
C	4.92182100	-1.40809700	-0.00052800	C	-4.21786700	-4.09242600	1.22189200
H	2.46096400	2.49533600	0.00002300	H	-1.99885300	-5.66876100	-2.49063700
H	-0.00003400	2.49525800	0.00047100	H	-0.35012600	-7.47466000	-2.35941700
H	-0.00006200	-2.49521800	0.00023800	H	-0.32790800	-8.23179300	1.44430300
H	2.46093900	-2.49535600	-0.00017900	H	-3.36778300	-6.38970700	2.26863100
H	4.92164700	-2.49568100	-0.00054100	H	-4.54759300	-4.26264600	2.24438000
C	7.38166100	1.40847700	-0.00068700	C	-4.44791600	-1.44221300	-1.40422100
C	8.61761200	0.73076400	-0.00074900	C	-5.21895000	-0.44393900	-0.76355500
C	8.61762600	-0.73087600	-0.00075300	C	-5.67828000	-0.68400900	0.60322000
C	7.38168000	-1.40858100	-0.00072800	C	-5.33151000	-1.90422700	1.22960900
H	7.38157600	2.49604400	-0.00069300	H	-4.10682500	-1.26261800	-2.42127800
H	7.38158900	-2.49614800	-0.00070500	H	-5.67238900	-2.08130700	2.24724600

C	-11.07958600	0.72990200	0.00067300	C	6.97538800	0.81766600	-0.65317500
C	-9.84144200	1.40875000	0.00091100	C	6.26375900	-0.19420500	-1.31353200
C	-9.84139100	-1.40863200	0.00084000	C	5.82003900	0.21244600	1.44160000
C	-11.07955300	-0.72981100	0.00065100	C	6.74531500	1.02848200	0.77508900
H	-9.84176700	2.49630000	0.00091700	H	6.43483000	-0.35090200	-2.37605900
H	-9.84169000	-2.49618200	0.00085400	H	5.64870600	0.36943700	2.50402200
C	9.84139400	1.40864800	-0.00075900	C	-5.55389800	0.75534600	-1.38924000
C	11.07959100	0.72979700	-0.00069400	C	-6.32676400	1.75467400	-0.74743400
C	11.07961300	-0.72989300	-0.00066000	C	-6.78549800	1.51493000	0.61570300
C	9.84142800	-1.40875300	-0.00072700	C	-6.43922400	0.29248400	1.24312400
H	9.84175300	2.49619900	-0.00079200	H	-5.21185400	0.93506600	-2.40593200
H	9.84179800	-2.49630400	-0.00067300	H	-6.78133100	0.11440600	2.26011800
C	12.30268100	1.40777100	-0.00061100	C	-6.66180600	2.95505900	-1.37416300
C	13.53705500	0.72707500	-0.00042100	C	-7.43250800	3.94811500	-0.73076500
C	13.53708300	-0.72712000	-0.00035000	C	-7.88971200	3.70919000	0.62728500
C	12.30272800	-1.40784300	-0.00050800	C	-7.54728200	2.49222200	1.25647600
H	12.30370500	2.49533800	-0.00067400	H	-6.31978900	3.13492800	-2.39083200
H	12.30378200	-2.49541100	-0.00043100	H	-7.88981700	2.31422400	2.27331900
C	14.76810800	1.40553200	-0.00025600	C	-7.77191600	5.15783000	-1.35812000
C	15.98775500	0.72397300	0.00003800	C	-8.53536400	6.13591400	-0.71411300
C	15.98778500	-0.72393900	0.00014900	C	-8.99084500	5.89791100	0.63864900
C	14.76816600	-1.40553800	-0.00007600	C	-8.65631000	4.69565700	1.26869500
H	14.76885800	2.49324000	-0.00035300	H	-7.42969500	5.33733800	-2.37492400
H	14.76895900	-2.49324700	0.00002900	H	-8.99886100	4.51730600	2.28560300
C	-13.53707600	0.72711700	0.00002700	C	8.61188600	2.64404500	-0.65804100
C	-12.30276300	1.40782500	0.00038700	C	7.90546000	1.63865500	-1.31924400
C	-12.30269300	-1.40777700	0.00038000	C	7.46167000	2.04518100	1.43527900
C	-13.53703600	-0.72711600	0.00003900	C	8.38206800	2.85455900	0.76837600
H	-12.30376700	2.49539500	0.00036500	H	8.07678400	1.48212400	-2.38176800
H	-12.30365400	-2.49534700	0.00041200	H	7.29052100	2.20227400	2.49773700
C	-15.98779700	0.72390500	-0.00085500	C	10.24781200	4.46860300	-0.66201800
C	-14.76819700	1.40550700	-0.00040900	C	9.54918800	3.47240200	-1.32374100
C	-14.76811600	-1.40556500	-0.00034300	C	9.10573400	3.87851500	1.42843500
C	-15.98775600	-0.72402400	-0.00080300	C	10.01848200	4.67863200	0.76131900
H	-14.76898900	2.49321700	-0.00046400	H	9.72045200	3.31574100	-2.38640000
H	-14.76884900	-2.49327400	-0.00028500	H	8.93448800	4.03545300	2.49104900
C	-18.42341000	0.71292400	-0.00187400	C	11.87402000	6.28478600	-0.65633900
C	-17.24239100	1.40701400	-0.00140600	C	11.19885800	5.31070500	-1.33130000
C	-17.24231200	-1.40719400	-0.00124900	C	10.75473900	5.71738900	1.42499200
C	-18.42336900	-0.71316300	-0.00177600	C	11.64825600	6.49153800	0.74487500
H	-19.36967400	1.24614700	-0.00233100	H	12.59056800	6.91406300	-1.17638200
H	-17.23898700	2.49407100	-0.00149600	H	11.36775500	5.15161600	-2.39333000
H	-17.23885200	-2.49425100	-0.00115100	H	10.58135200	5.87168900	2.48700100
H	-19.36960600	-1.24643300	-0.00211500	H	12.19789100	7.27365600	1.26068500
C	17.24230900	1.40714100	0.00025100	C	-8.88438400	7.36854600	-1.34558600
C	18.42336100	0.71310000	0.00061100	C	-9.63404500	8.30955600	-0.69020900
C	18.42339100	-0.71298600	0.00076800	C	-10.08282700	8.07506000	0.64256100
C	17.24236600	-1.40706600	0.00052300	C	-9.76996400	6.90581400	1.28444300
H	17.23884900	2.49419800	0.00010900	H	-8.54051200	7.54422000	-2.36182000
H	19.36960600	1.24635700	0.00077200	H	-9.89156900	9.24243000	-1.18348600
H	19.36965300	-1.24621100	0.00109000	H	-10.67623500	8.83243800	1.14669100
H	17.23894500	-2.49412300	0.00065900	H	-10.11050600	6.72388500	2.30070600
				H	-0.20808000	-8.51394700	-0.94130200
				H	-0.92834800	-6.94916300	2.48792300
				H	-2.54800300	-8.26324500	0.89367700
				H	-2.56829000	-7.94416000	-1.34480200
**[16]-acene**				**[16]-acene _H$_4$**			
C	9.84837800	-0.73078500	0.00034700	C	5.82416500	-0.23299600	-0.74515600

C	8.61243000	-1.40859300	0.00037900
C	7.38514800	-0.73066100	0.00039600
C	7.38514000	0.73055300	0.00043300
C	8.61241400	1.40848500	0.00042100
C	9.84836400	0.73067900	0.00036000
C	6.15251700	-1.40815300	0.00037500
C	6.15251200	1.40805500	0.00045800
C	4.92256300	0.73011300	0.00042900
C	4.92256500	-0.73020000	0.00037200
C	3.69189000	-1.40781700	0.00031800
H	3.69175400	-2.49542200	0.00030300
C	2.46097500	-0.72982300	0.00029400
C	2.46097600	0.72977300	0.00036500
C	3.69189400	1.40774800	0.00043300
H	6.15236700	-2.49574500	0.00035600
H	8.61241900	-2.49616200	0.00039100
H	8.61240200	2.49605300	0.00044200
H	6.15237200	2.49564700	0.00048000
H	3.69177600	2.49535300	0.00048200
C	1.23069100	-1.40764800	0.00020500
C	-0.00000600	-0.72967600	0.00017200
C	-0.00000400	0.72967400	0.00024900
C	1.23070000	1.40762100	0.00033900
C	-1.23070700	-1.40762400	0.00007700
C	-1.23069700	1.40764500	0.00021500
C	-2.46098500	0.72982600	0.00011800
C	-2.46098600	-0.72978100	0.00004500
C	-3.69190100	-1.40775000	-0.00004300
H	-3.69179100	-2.49535500	-0.00006500
C	-4.92257100	-0.73011300	-0.00007900
C	-4.92257300	0.73019600	-0.00001800
C	-3.69189600	1.40781500	0.00008000
H	-1.23067600	-2.49523300	0.00003700
H	1.23063500	-2.49525700	0.00016500
H	1.23066500	2.49523000	0.00038100
H	-1.23064500	2.49525500	0.00025700
H	-3.69176900	2.49542000	0.00013000
C	-6.15251600	-1.40805800	-0.00016000
C	-7.38514600	-0.73055200	-0.00019200
C	-7.38515400	0.73065700	-0.00015000
C	-6.15251900	1.40815300	-0.00006800
H	-6.15238100	-2.49564900	-0.00018200
H	-6.15237500	2.49574400	-0.00004200
C	12.31041300	-0.72977200	0.00019500
C	11.07220600	-1.40874300	0.00028600
C	11.07217600	1.40865100	0.00028100
C	12.31039200	0.72969600	0.00018400
H	11.07259000	-2.49629000	0.00029800
H	11.07254600	2.49619800	0.00026700
C	-8.61241400	-1.40848800	-0.00024200
C	-9.84836700	-0.73068000	-0.00025900
C	-9.84838100	0.73078400	-0.00024300
C	-8.61242900	1.40859400	-0.00019500
H	-8.61240500	-2.49605700	-0.00024700
H	-8.61242100	2.49616200	-0.00018500
C	-11.07217500	-1.40865200	-0.00026700
C	-12.31039000	-0.72969600	-0.00026200
C	-12.31041100	0.72977200	-0.00027300
C	-11.07220300	1.40874300	-0.00027100
H	-11.07254600	-2.49620000	-0.00025300
C	4.82315100	-1.00262200	-1.38295100
C	4.20812900	-2.09157200	-0.76235100
C	4.60941800	-2.45215800	0.59652100
C	5.59644900	-1.69775400	1.23362400
C	6.22552500	-0.59372900	0.61201000
C	3.21063200	-2.86034700	-1.40019400
C	3.98229700	-3.55384700	1.21756800
C	2.99209300	-4.31492100	0.58222500
C	2.59120400	-3.95472500	-0.78222700
C	1.60854200	-4.72141400	-1.42372000
H	1.32139500	-4.46106300	-2.43991800
C	0.97871800	-5.83482600	-0.80962100
C	1.35273400	-6.17209500	0.57892600
C	2.36601900	-5.40231600	1.20721000
H	2.91292500	-2.59143500	-2.41118600
H	4.52441400	-0.73346800	-2.39354900
H	5.89524000	-1.96593300	2.24449900
H	4.28028100	-3.82044300	2.22915800
H	2.65452100	-5.65893300	2.22411800
C	0.05765000	-6.60994600	-1.46695900
C	-0.61882200	-7.82111900	-0.88949300
C	-0.44938800	-7.96951900	0.65850300
C	0.70895100	-7.20008300	1.22079900
C	-2.12208100	-7.78336100	-1.27817000
C	-1.75743900	-7.58267600	1.43338100
C	-2.48991000	-6.43980700	0.79042500
C	-2.75009500	-6.58162400	-0.62542200
C	-3.43366200	-5.61430100	-1.29834900
H	-3.61289700	-5.71180300	-2.36703700
C	-3.91554700	-4.43733800	-0.63235800
C	-3.63434500	-4.28408300	0.78904900
C	-2.90789700	-5.32700200	1.45629300
H	-2.22764500	-7.73583400	-2.36733600
H	-0.20672300	-6.35159200	-2.49103400
H	0.98728100	-7.43075000	2.24791000
H	-2.41684900	-8.46217400	1.44503000
H	-2.69871200	-5.21213700	2.51757300
C	-4.61708100	-3.44689400	-1.29745300
C	-5.07551800	-2.27870200	-0.63753300
C	-4.79235900	-2.12437600	0.78650400
C	-4.07239400	-3.14975400	1.45016700
H	-4.82547000	-3.56081800	-2.35895100
H	-3.86137100	-3.03494700	2.51096500
C	7.44043800	1.62211700	-0.72870600
C	6.43857400	0.85422200	-1.36717200
C	7.21259100	0.15838800	1.24956500
C	7.84137400	1.26171600	0.62645800
H	6.13981000	1.12346300	-2.37769200
H	7.51170500	-0.10998800	2.26022900
C	-5.78388800	-1.27796900	-1.30195600
C	-6.23675300	-0.11874400	-0.64169100
C	-5.95235200	0.03621900	0.78391900
C	-5.23633000	-0.97951200	1.44753300
H	-5.99490000	-1.39317400	-2.36265300
H	-5.02456800	-0.86436800	2.50804900
C	-6.94982300	0.88895600	-1.30530400
C	-7.39904300	2.04132400	-0.64454400
C	-7.11408000	2.19654800	0.78093700
C	-6.40058200	1.18816300	1.44456600
H	-7.16155600	0.77368100	-2.36580600

H	-11.07258700	2.49629100	-0.00028000	H	-6.18854000	1.30365100	2.50496400
C	-13.53352100	-1.40777900	-0.00021800	C	-8.11631900	3.05545400	-1.30766400
C	-14.76788400	-0.72701500	-0.00018500	C	-8.56167300	4.20049200	-0.64617200
C	-14.76791100	0.72703400	-0.00022400	C	-8.27677200	4.35565000	0.77739200
C	-13.53356300	1.40782700	-0.00027600	C	-7.56638000	3.35494000	1.44141800
H	-13.53442800	-2.49534200	-0.00018300	H	-8.32852100	2.94023800	-2.36809300
H	-13.53449700	2.49539100	-0.00030600	H	-7.35422400	3.47073800	2.50178200
C	14.76791800	-0.72703300	-0.00004600	C	9.05564200	3.47258300	-0.71177300
C	13.53356800	-1.40782600	0.00010100	C	8.05679900	2.71042000	-1.35146000
C	13.53352400	1.40777900	0.00004700	C	8.83062800	2.01476000	1.26397700
C	14.76789000	0.72701700	-0.00008200	C	9.45530200	3.11331500	0.63890800
H	13.53450300	-2.49538900	0.00013100	H	7.75827300	2.97993400	-2.36197400
H	13.53443200	2.49534200	0.00001400	H	9.13012200	1.74660100	2.27457600
C	17.21865500	-0.72391200	-0.00037100	C	10.66791000	5.31774700	-0.69504700
C	15.99906300	-1.40552000	-0.00017000	C	9.67876700	4.56790500	-1.33512400
C	15.99900700	1.40554500	-0.00027700	C	10.45156900	3.87319500	1.27657600
C	17.21862300	0.72398000	-0.00043600	C	11.06606600	4.95983100	0.65049800
H	15.99976600	-2.49323100	-0.00011800	H	9.38002400	4.83722500	-2.34577500
H	15.99967100	2.49325600	-0.00033100	H	10.75097500	3.60478500	2.28727900
C	19.65428600	-0.71298000	-0.00076600	C	12.27324300	7.14964700	-0.67119300
C	18.47332900	-1.40704400	-0.00052300	C	11.30576600	6.43196800	-1.32256900
C	18.47326500	1.40716400	-0.00068800	C	12.07953800	5.73640900	1.29232000
C	19.65425300	0.71315100	-0.00085600	C	12.66547100	6.79706100	0.65430500
H	20.60057800	-1.24621200	-0.00087700	H	12.74919800	7.99418900	-1.16118700
H	18.46993500	-2.49411900	-0.00043400	H	11.00453400	6.69805500	-2.33265500
H	18.46982400	2.49423900	-0.00075300	H	12.37611600	5.46511300	2.30240300
H	20.60052100	1.24642500	-0.00106100	H	13.43460700	7.37806200	1.15505600
C	-15.99900200	-1.40554400	-0.00008400	C	-9.28419400	5.22350600	-1.30893400
C	-17.21861500	-0.72397700	-0.00002200	C	-9.72387000	6.35792600	-0.64701500
C	-17.21864600	0.72391500	-0.00009100	C	-9.43944000	6.51280900	0.77343900
C	-15.99905600	1.40552300	-0.00019700	C	-8.73429700	5.52291700	1.43769900
H	-15.99966600	-2.49325500	-0.00002900	H	-9.49652900	5.10805100	-2.36945400
H	-15.99975800	2.49323400	-0.00025300	H	-8.52192500	5.63864400	2.49817500
C	-18.47325800	-1.40716100	0.00013300	C	-10.45648700	7.39755300	-1.31335200
C	-19.65424400	-0.71314800	0.00020700	C	-10.87831300	8.50507400	-0.63829200
C	-19.65427600	0.71298500	0.00011200	C	-10.59827100	8.65756200	0.76007500
C	-18.47332000	1.40704800	-0.00004100	C	-9.90561300	7.69747800	1.43735600
H	-18.46981800	-2.49423600	0.00020200	H	-10.66714000	7.27914600	-2.37323300
H	-20.60051400	-1.24642000	0.00034000	H	-11.43162100	9.28328700	-1.15608400
H	-20.60056900	1.24621700	0.00014900	H	-10.94453300	9.54849400	1.27602700
H	-18.46992500	2.49412400	-0.00013100	H	-9.69172100	7.81017700	2.49720700
				H	-1.51901300	-7.35674200	2.47762500
				H	-2.60541600	-8.71399300	-0.94886100
				H	-0.28083700	-9.03297100	0.87268100
				H	-0.17675300	-8.71129900	-1.36414800
**[17]-acene**				**[17]-acene _H$_4$**			
C	0.00000000	11.07921000	0.73071700	C	-6.84920500	-0.09806400	-0.65558700
C	0.00000000	9.84323300	1.40852800	C	-6.14843900	-1.12132400	-1.31540200
C	0.00000000	8.61587300	0.73060200	C	-5.23602900	-1.94689300	-0.64868500
C	0.00000000	8.61587300	-0.73060200	C	-5.00326400	-1.73862600	0.78079200
C	0.00000000	9.84323300	-1.40852800	C	-5.69945500	-0.71974900	1.44089100
C	0.00000000	11.07921000	-0.73071700	C	-6.61620600	0.11027300	0.77455300
C	0.00000000	7.38329900	1.40808500	C	-4.53148000	-2.97532300	-1.30796200
C	0.00000000	7.38329900	-1.40808500	C	-4.08320500	-2.57400500	1.44725200
C	0.00000000	6.15319000	-0.73016000	C	-3.39221800	-3.58797700	0.78689000
C	0.00000000	6.15319000	0.73016000	C	-3.62433300	-3.79581800	-0.64044500
C	0.00000000	4.92266700	1.40780200	C	-2.91318400	-4.83144400	-1.29926800
H	0.00000000	4.92228000	2.49539400	H	-3.08399900	-4.98489900	-2.36248800

C	0.00000000	3.69146300	0.72979600	C	-2.01421200	-5.64357300	-0.63116200
C	0.00000000	3.69146300	-0.72979600	C	-1.78318900	-5.43657200	0.79309500
C	0.00000000	4.92266700	-1.40780200	C	-2.46638800	-4.43092900	1.45344100
H	0.00000000	7.38312400	2.49567100	H	-4.70410800	-3.13016800	-2.37053900
H	0.00000000	9.84342300	2.49608600	H	-6.32140600	-1.27607300	-2.37788400
H	0.00000000	9.84342300	-2.49608600	H	-5.52611300	-0.56485500	2.50327100
H	0.00000000	7.38312400	-2.49567100	H	-3.90974100	-2.41912000	2.50965000
H	0.00000000	4.92228000	-2.49539400	H	-2.29312400	-4.27603600	2.51597300
C	0.00000000	2.46146900	1.40769600	C	-1.27891200	-6.68251500	-1.29583000
C	0.00000000	1.23040800	0.72957400	C	-0.40188200	-7.47577400	-0.61993100
C	0.00000000	1.23040800	-0.72957400	C	-0.18770700	-7.28349800	0.79802800
C	0.00000000	2.46146900	-1.40769600	C	-0.84670300	-6.29403100	1.46320300
C	0.00000000	0.00000000	1.40767500	C	0.48054300	-8.50727500	-1.26929700
C	0.00000000	0.00000000	-1.40767500	C	0.78031100	-8.23420000	1.44195900
C	0.00000000	-1.23040800	-0.72957400	C	2.14608300	-8.30768500	0.67408500
C	0.00000000	-1.23040800	0.72957400	C	1.95357500	-8.20930400	-0.87581900
C	0.00000000	-2.46146900	1.40769600	C	2.34631700	-6.88202500	-1.46048500
H	0.00000000	-2.46112200	2.49527700	H	2.04232600	-6.69986400	-2.48997000
C	0.00000000	-3.69146300	0.72979600	C	3.05737800	-5.91078200	-0.80317300
C	0.00000000	-3.69146300	-0.72979600	C	3.48298800	-6.14121200	0.59226900
C	0.00000000	-2.46146900	-1.40769600	C	3.08823900	-7.28576600	1.23817700
H	0.00000000	0.00000000	2.49525000	H	0.21532900	-9.52198500	-0.94053200
H	0.00000000	2.46112200	2.49527700	H	-1.42565600	-6.81419100	-2.36579600
H	0.00000000	2.46112200	-2.49527700	H	-0.67431500	-6.13852500	2.52591600
H	0.00000000	0.00000000	-2.49525000	H	0.95541600	-7.96569900	2.48866500
H	0.00000000	-2.46112200	-2.49527700	H	3.40163800	-7.43805900	2.26979900
C	0.00000000	-4.92266700	1.40780200	C	3.42134400	-4.68768500	-1.42365500
C	0.00000000	-6.15319000	0.73016000	C	4.19162600	-3.70888600	-0.78069800
C	0.00000000	-6.15319000	-0.73016000	C	4.64743700	-3.95464400	0.59189900
C	0.00000000	-4.92266700	-1.40780200	C	4.28279200	-5.15248000	1.22223900
H	0.00000000	-4.92228000	2.49539400	H	3.09443300	-4.50948300	-2.44554900
H	0.00000000	-4.92228000	-2.49539400	H	4.61048700	-5.32693600	2.24467400
C	0.00000000	13.54133900	0.72968000	C	-8.46337100	1.74987600	-0.66132500
C	0.00000000	12.30302300	1.40871600	C	-7.76584100	0.73132000	-1.32177200
C	0.00000000	12.30302300	-1.40871600	C	-7.31673200	1.13274500	1.43461000
C	0.00000000	13.54133900	-0.72968000	C	-8.23046500	1.95802700	0.76818700
H	0.00000000	12.30367500	2.49624300	H	-7.93899000	0.57673600	-2.38423800
H	0.00000000	12.30367500	-2.49624300	H	-7.14354900	1.28763800	2.49701400
C	0.00000000	-7.38329900	1.40808500	C	4.54827500	-2.50600900	-1.40442700
C	0.00000000	-8.61587300	0.73060200	C	5.33118500	-1.52098000	-0.76449800
C	0.00000000	-8.61587300	-0.73060200	C	5.78723400	-1.76676100	0.60269100
C	0.00000000	-7.38329900	-1.40808500	C	5.42547200	-2.97897100	1.22919000
H	0.00000000	-7.38312400	2.49567100	H	4.20951100	-2.32224500	-2.42153900
H	0.00000000	-7.38312400	-2.49567100	H	5.76394200	-3.16041300	2.24688300
C	0.00000000	-9.84323300	1.40852800	C	5.68394900	-0.32401600	-1.39057700
C	0.00000000	-11.07921000	0.73071700	C	6.46905900	0.66326100	-0.75032200
C	0.00000000	-11.07921000	-0.73071700	C	6.92502000	0.41756400	0.61527300
C	0.00000000	-9.84323300	-1.40852800	C	6.56264400	-0.79768700	1.24213800
H	0.00000000	-9.84342300	2.49608600	H	5.34429100	-0.14043900	-2.40740000
H	0.00000000	-9.84342300	-2.49608600	H	6.90205100	-0.98027000	2.25925800
C	0.00000000	-12.30302300	1.40871600	C	6.82086600	1.85875800	-1.37755500
C	0.00000000	-13.54133900	0.72968000	C	7.60629300	2.84507700	-0.73627800
C	0.00000000	-13.54133900	-0.72968000	C	8.06164400	2.59974700	0.62738400
C	0.00000000	-12.30302300	-1.40871600	C	7.70007900	1.38494400	1.25552100
H	0.00000000	-12.30367500	2.49624300	H	6.48129200	2.04227500	-2.39436900
H	0.00000000	-12.30367500	-2.49624300	H	8.03971300	1.20238400	2.27250600
C	0.00000000	15.99886800	0.72695900	C	-10.07774200	3.59638500	-0.66586600
C	0.00000000	14.76441100	1.40782300	C	-9.38453600	2.58351800	-1.32714000
C	0.00000000	14.76441100	-1.40782300	C	-8.93561200	2.98460800	1.42825000
C	0.00000000	15.99886800	-0.72695900	C	-9.84522700	3.80411900	0.76141700

H	0.00000000	14.76543100	2.49536600	H	-9.55783600	2.42915000	-2.38964000
H	0.00000000	14.76543100	-2.49536600	H	-8.76262400	3.13956200	2.49070900
C	0.00000000	18.44960400	0.72391800	C	-11.69142800	5.44095400	-0.66961200
C	0.00000000	17.22997100	1.40552000	C	-11.00545200	4.43686400	-1.33139000
C	0.00000000	17.22997100	-1.40552000	C	-10.55701300	4.83742400	1.42141200
C	0.00000000	18.44960400	-0.72391800	C	-11.45948400	5.64814200	0.75435300
H	0.00000000	17.23055600	2.49322300	H	-11.17863900	4.28239000	-2.39404500
H	0.00000000	17.23055600	-2.49322300	H	-10.38393400	4.99219500	2.48403600
C	0.00000000	20.88524600	0.71308400	C	-13.29539900	7.27693800	-0.66372400
C	0.00000000	19.70428900	1.40709800	C	-12.63236000	6.29496500	-1.33877200
C	0.00000000	19.70428900	-1.40709800	C	-12.18332200	6.69602600	1.41801500
C	0.00000000	20.88524600	-0.71308400	C	-13.06711000	7.48085000	0.73791000
H	0.00000000	21.83159600	1.24621300	H	-14.00423700	7.91503100	-1.18357400
H	0.00000000	19.70072000	2.49417300	H	-12.80318900	6.13806000	-2.40080700
H	0.00000000	19.70072000	-2.49417300	H	-12.00815000	6.84812500	2.48004000
H	0.00000000	21.83159600	-1.24621300	H	-13.60726900	8.26959700	1.25362000
C	0.00000000	-14.76441100	1.40782300	C	7.95905000	4.04242600	-1.36399000
C	0.00000000	-15.99886800	0.72695900	C	8.74211500	5.02290300	-0.72118300
C	0.00000000	-15.99886800	-0.72695900	C	9.19593200	4.77843700	0.63801800
C	0.00000000	-14.76441100	-1.40782300	C	8.83782700	3.56897600	1.26790300
H	0.00000000	-14.76543100	2.49536600	H	7.61974200	4.22616900	-2.38084900
H	0.00000000	-14.76543100	-2.49536600	H	9.17769000	3.38680800	2.28487200
C	0.00000000	-17.22997100	1.40552000	C	9.09924400	6.22894300	-1.34927500
C	0.00000000	-18.44960400	0.72391800	C	9.87524100	7.19546600	-0.70584900
C	0.00000000	-18.44960400	-0.72391800	C	10.32731000	6.95196800	0.64820700
C	0.00000000	-17.22997100	-1.40552000	C	9.97672100	5.75628500	1.27892700
H	0.00000000	-17.23055600	2.49322300	H	8.75984300	6.41239100	-2.36630400
H	0.00000000	-17.23055600	-2.49322300	H	10.31651000	5.57390000	2.29602400
C	0.00000000	-19.70428900	1.40709800	C	10.24195700	8.42348400	-1.33800700
C	0.00000000	-20.88524600	0.71308400	C	11.00404900	9.35388000	-0.68307800
C	0.00000000	-20.88524600	-0.71308400	C	11.44938200	9.11401800	0.65080800
C	0.00000000	-19.70428900	-1.40709800	C	11.12049300	7.95030200	1.29343400
H	0.00000000	-19.70072000	2.49417300	H	9.90091600	8.60319100	-2.35447800
H	0.00000000	-21.83159600	1.24621300	H	11.27483400	10.28283400	-1.17663400
H	0.00000000	-21.83159600	-1.24621300	H	12.05304500	9.86369100	1.15426200
H	0.00000000	-19.70072000	-2.49417300	H	11.45820100	7.76445200	2.30991700
				H	0.33840700	-9.24075600	1.44545000
				H	0.37098100	-8.48496900	-2.35885300
				H	2.55794200	-9.30116300	0.89473700
				H	2.58292000	-8.98237400	-1.34405700
**[18]-acene**				**[18]-acene _H_4**			
C	9.84629500	0.73063600	0.00165700	C	6.28825800	-1.17361400	-0.63565400
C	8.61368300	1.40813600	0.00169800	C	5.84547700	-2.34002300	-1.29300900
C	7.38361200	0.73021900	0.00158900	C	5.14000300	-3.34096500	-0.62813700
C	7.38359500	-0.73019800	0.00150800	C	4.84835600	-3.18230400	0.79457500
C	8.61366600	-1.40811400	0.00157400	C	5.28143300	-2.03315500	1.45305900
C	9.84627600	-0.73061500	0.00161100	C	5.99498700	-1.01413400	0.78895100
C	6.15315400	1.40784500	0.00149500	C	4.69042600	-4.51477800	-1.28564200
C	6.15313900	-1.40782600	0.00131700	C	4.12994300	-4.20955300	1.45846900
C	4.92185000	-0.72986300	0.00114900	C	3.70059800	-5.34774400	0.79942700
C	4.92186600	0.72987800	0.00125300	C	3.98987800	-5.50514400	-0.62051800
C	3.69207600	1.40771300	0.00106300	C	3.51460200	-6.68601100	-1.28494400
H	3.69199600	2.49531800	0.00114100	H	3.69885700	-6.78617400	-2.35252500
C	2.46078300	0.72969100	0.00072000	C	2.82979000	-7.65243800	-0.61243800
C	2.46076800	-0.72968600	0.00060300	C	2.56241100	-7.50683500	0.80213400
C	3.69206400	-1.40770300	0.00085000	C	2.97404900	-6.39105400	1.46651100
H	6.15302300	2.49544700	0.00154700	H	4.90466500	-4.63174000	-2.34563600
H	8.61354000	2.49572600	0.00174200	H	6.06266100	-2.45843900	-2.35209200

H	8.61354200	-2.49570400	0.00156900	H	5.06326700	-1.91480700	2.51190900
H	6.15303000	-2.49542800	0.00127500	H	3.91272600	-4.09161400	2.51766300
H	3.69200700	-2.49530700	0.00080900	H	2.75885500	-6.27314500	2.52624600
C	1.23071900	1.40767300	0.00045500	C	2.20412300	-8.85570400	-1.26468900
C	0.00000200	0.72963600	0.00006600	C	0.69850900	-8.88879700	-0.88374200
C	-0.00001300	-0.72964300	-0.00005700	C	0.52112100	-9.03343000	0.66386900
C	1.23070700	-1.40767400	0.00022400	C	1.82694800	-8.64839100	1.44375500
C	-1.23071700	1.40766600	-0.00021300	C	0.03002800	-7.67567400	-1.46665500
C	-1.23072900	-1.40768000	-0.00044800	C	-0.63615000	-8.25748300	1.21955400
C	-2.46079400	-0.72970300	-0.00071000	C	-1.26834200	-7.22452400	0.57427800
C	-2.46077800	0.72968400	-0.00058700	C	-0.88614500	-6.89180100	-0.81305000
C	-3.69207300	1.40769700	-0.00083000	C	-1.50312900	-5.77246900	-1.43013300
H	-3.69202100	2.49530200	-0.00078900	H	-1.20976500	-5.51543600	-2.44539100
C	-4.92186000	0.72985500	-0.00112500	C	-2.47836700	-4.99515600	-0.79147500
C	-4.92187700	-0.72988200	-0.00123900	C	-2.88791300	-5.35082500	0.57107400
C	-3.69208800	-1.40772000	-0.00105700	C	-2.27706300	-6.44480000	1.19828800
H	-1.23071200	2.49527000	-0.00014500	H	0.29927300	-7.42130600	-2.49044900
H	1.23068600	2.49527600	0.00052200	H	2.31560000	-8.81183400	-2.35339400
H	1.23070000	-2.49527700	0.00015700	H	2.48496000	-9.52885100	1.45845500
H	-1.23070000	-2.49528400	-0.00051900	H	-0.92094300	-8.48542000	2.24548000
H	-3.69201300	-2.49532500	-0.00114800	H	-2.57210500	-6.69824500	2.21411300
C	-6.15314400	1.40782300	-0.00126900	C	-3.08257400	-3.89154500	-1.41070300
C	-7.38360400	0.73019200	-0.00144900	C	-4.06947800	-3.11108300	-0.77468100
C	-7.38362200	-0.73022300	-0.00154400	C	-4.47967000	-3.46733400	0.58188100
C	-6.15316100	-1.40785200	-0.00147100	C	-3.87135700	-4.57674500	1.20371200
H	-6.15304000	2.49542400	-0.00121600	H	-2.77817200	-3.62575600	-2.42053300
H	-6.15303500	-2.49545400	-0.00153200	H	-4.17594300	-4.84009800	2.21419400
C	12.30955500	0.73074000	0.00139400	C	7.43545200	0.99532300	-0.64257700
C	11.07354600	1.40855200	0.00158000	C	6.99675500	-0.16605600	-1.30000800
C	11.07352700	-1.40853200	0.00153800	C	6.43041300	0.14185000	1.44670700
C	12.30953600	-0.73072300	0.00139700	C	7.14123000	1.15525300	0.78249400
H	11.07353300	2.49612000	0.00157700	H	7.21487600	-0.28468000	-2.35882100
H	11.07352900	-2.49610000	0.00154600	H	6.21177500	0.26059300	2.50538000
C	-8.61366600	1.40811200	-0.00148800	C	-4.66782500	-2.00981200	-1.39520600
C	-9.84628300	0.73061200	-0.00152800	C	-5.65544700	-1.22753200	-0.75867900
C	-9.84630300	-0.73063900	-0.00159000	C	-6.06611500	-1.58426500	0.59662800
C	-8.61368500	-1.40814200	-0.00164100	C	-5.45869700	-2.69695900	1.21742300
H	-8.61354700	2.49570300	-0.00145500	H	-4.36206400	-1.74370500	-2.40453000
H	-8.61354800	-2.49573200	-0.00168200	H	-5.76419000	-2.96212800	2.22711000
C	-11.07352500	1.40853200	-0.00145000	C	-6.25153400	-0.12592300	-1.37956200
C	-12.30953700	0.73072200	-0.00132700	C	-7.23899400	0.65720000	-0.74226800
C	-12.30955800	-0.73073900	-0.00134500	C	-7.65003300	0.30011000	0.61302300
C	-11.07354700	-1.40855400	-0.00152800	C	-7.04369600	-0.81421300	1.23357900
H	-11.07352900	2.49610000	-0.00144100	H	-5.94543600	0.14020000	-2.38872800
H	-11.07353700	-2.49612300	-0.00153700	H	-7.34950200	-1.07988200	2.24297200
C	-13.53328500	1.40868900	-0.00112300	C	-7.83353900	1.75904700	-1.36301300
C	-14.77155400	0.72972200	-0.00082700	C	-8.82054800	2.54226500	-0.72433400
C	-14.77157500	-0.72972600	-0.00079200	C	-9.23133300	2.18539100	0.62946500
C	-13.53330900	-1.40869900	-0.00110700	C	-8.62639800	1.07018200	1.25065000
H	-13.53367000	2.49623600	-0.00114600	H	-7.52749800	2.02541400	-2.37208600
H	-13.53368100	-2.49624700	-0.00107600	H	-8.93260200	0.80454600	2.25987700
C	14.77158000	0.72973000	0.00080500	C	8.58183600	3.16467500	-0.64891200
C	13.53331100	1.40869900	0.00113100	C	8.14642900	2.00794800	-1.30662500
C	13.53329000	-1.40868700	0.00119100	C	7.57879600	2.31641300	1.43984900
C	14.77156100	-0.72972200	0.00086400	C	8.28721900	3.32476800	0.77538700
H	13.53368200	2.49624700	0.00109100	H	8.36517800	1.88923100	-2.36528700
H	13.53367500	-2.49623400	0.00123700	H	7.35985300	2.43547000	2.49842100
C	17.22906400	0.72701600	-0.00009200	C	9.72730300	5.33345300	-0.65446700
C	15.99467500	1.40780000	0.00035400	C	9.29566900	4.18277700	-1.31275900
C	15.99465400	-1.40779700	0.00052200	C	8.72741800	4.49148700	1.43248600

C	17.22904500	-0.72701700	0.00002000	C	9.43281500	5.49344100	0.76750800
H	15.99558800	2.49536400	0.00026700	H	9.51487900	4.06408700	-2.37135600
H	15.99558100	-2.49536100	0.00060400	H	8.50831500	4.61085200	2.49102200
C	19.67978400	0.72393400	-0.00123700	C	10.87163900	7.50067400	-0.65948500
C	18.46016900	1.40552900	-0.00070600	C	10.44554700	6.35991100	-1.31813400
C	18.46014900	-1.40553500	-0.00044200	C	9.87734700	6.66854500	1.42437300
C	19.67976700	-0.72394500	-0.00108200	C	10.57765700	7.66036900	0.75912600
H	18.46084700	2.49324000	-0.00083200	H	10.66491200	6.24094800	-2.37682100
H	18.46084200	-2.49324500	-0.00032300	H	9.65797800	6.78784000	2.48301700
C	-15.99464500	1.40780200	-0.00049800	C	-9.41514600	3.64568200	-1.34477700
C	-17.22903200	0.72702200	-0.00003600	C	-10.39883400	4.42497100	-0.70428100
C	-17.22905200	-0.72701000	0.00005100	C	-10.80834900	4.06921000	0.64495100
C	-15.99467000	-1.40779700	-0.00037800	C	-10.20788900	2.95696100	1.26746200
H	-15.99557000	2.49536500	-0.00055800	H	-9.10936600	3.91245900	-2.35382700
H	-15.99558200	-2.49536100	-0.00030600	H	-10.51456600	2.69161800	2.27662500
C	-18.46013900	1.40554200	0.00040700	C	-10.99845400	5.53561100	-1.32471100
C	-19.67975200	0.72395400	0.00100200	C	-11.97244200	6.30356900	-0.68344100
C	-19.67977100	-0.72392800	0.00113100	C	-12.38038400	5.94918500	0.66040900
C	-18.46016300	-1.40552300	0.00062100	C	-11.79016200	4.84782400	1.28354200
H	-18.46082600	2.49325300	0.00030800	H	-10.69245900	5.80225400	-2.33388900
H	-18.46083800	-2.49323400	0.00072800	H	-12.09682900	4.58221500	2.29279300
C	-20.93440100	1.40712400	0.00153400	C	-12.58552400	7.43351200	-1.30775700
C	-22.11537400	0.71308500	0.00220300	C	-13.53837400	8.16926600	-0.65509500
C	-22.11539200	-0.71304200	0.00236100	C	-13.94020900	7.82018900	0.66858000
C	-20.93442400	-1.40709000	0.00182100	C	-13.37823000	6.74488300	1.30350500
H	-20.93098200	2.49419900	0.00140200	H	-12.27709200	7.69687700	-2.31637400
H	-23.06165300	1.24634000	0.00262200	H	-13.99537900	9.02562900	-1.14259000
H	-23.06167900	-1.24628100	0.00292200	H	-14.69753000	8.41567100	1.17031100
H	-20.93099900	-2.49416500	0.00194600	H	-13.68216600	6.47628000	2.31210900
C	22.11540300	0.71305100	-0.00254000	C	12.00794500	9.65760300	-0.65501500
C	20.93443100	1.40709800	-0.00197500	C	11.59948100	8.54342200	-1.32684000
C	20.93441200	-1.40711300	-0.00163700	C	11.03022900	8.85261000	1.41956600
C	22.11538700	-0.71307100	-0.00235600	C	11.71850500	9.81482500	0.74133000
H	23.06168700	1.24629500	-0.00314000	H	12.55765700	10.43792100	-1.17345100
H	20.93100800	2.49417200	-0.00211800	H	11.81721800	8.42149000	-2.38487700
H	20.93099700	-2.49418800	-0.00148300	H	10.80933000	8.96888700	2.47758100
H	23.06166500	-1.24633000	-0.00279300	H	12.05432400	10.71131200	1.25454100
				H	2.68392400	-9.78609300	-0.92972300
				H	1.58476700	-8.42122800	2.48685500
				H	0.25610300	-9.77832300	-1.35919600
				H	0.34797400	-10.09570100	0.87991000
**[19]-acene**				**[19]-acene _H_4**			
C	8.61419200	0.73047900	-0.00073500	C	-5.88378200	-2.82837600	0.60479900
C	7.38369700	1.40808500	-0.00059800	C	-5.50797900	-4.03290500	1.23376600
C	6.15240600	0.73011800	-0.00050300	C	-4.72551400	-5.00551700	0.59478400
C	6.15241900	-0.72967300	-0.00059400	C	-4.28183700	-4.76150800	-0.78157600
C	7.38371400	-1.40759600	-0.00075100	C	-4.65418400	-3.56333600	-1.40769600
C	8.61418500	-0.72995100	-0.00080500	C	-5.44014600	-2.58450400	-0.76603000
C	4.92264100	1.40791400	-0.00030500	C	-4.34658800	-6.19701300	1.22746200
C	4.92269200	-1.40754200	-0.00049100	C	-3.50764700	-5.73527300	-1.42621000
C	3.69127400	-0.72958500	-0.00027700	C	-3.12820400	-6.95271800	-0.80328400
C	3.69124700	0.72988100	-0.00017300	C	-3.54275200	-7.18163800	0.59563400
C	2.46133500	1.40781000	0.00006400	C	-3.13498700	-8.32079200	1.24296300
H	2.46126100	2.49541200	0.00012300	H	-3.44096000	-8.47208900	2.27695800
C	1.23037800	0.72972900	0.00020600	C	-2.18909600	-9.33792400	0.67643300
C	1.23041200	-0.72962300	0.00009600	C	-2.00194500	-9.24058900	-0.87425800
C	2.46140900	-1.40760800	-0.00014000	C	-2.41214700	-7.91961800	-1.46147900
H	4.92253900	2.49551800	-0.00026100	H	-4.66580900	-6.37027100	2.25277600

H	7.38356100	2.49568700	-0.00056200	H	-5.83752500	-4.21304300	2.25462500
H	7.38362800	-2.49519800	-0.00076500	H	-4.32443600	-3.38083900	-2.42800600
H	4.92267000	-2.49514500	-0.00052300	H	-3.18937800	-5.55821500	-2.45102900
H	2.46143200	-2.49520900	-0.00017000	H	-2.11669200	-7.73862100	-2.49367400
C	-0.00004100	1.40771500	0.00044000	C	-0.82114100	-9.25633600	1.44036800
C	-1.23041500	0.72962900	0.00057000	C	0.13904300	-8.30018800	0.79241900
C	-1.23038000	-0.72972700	0.00046100	C	0.34631400	-8.48327200	-0.61811700
C	0.00004000	-1.40771100	0.00023200	C	-0.52622500	-9.52096500	-1.27124100
C	-2.46141000	1.40761200	0.00078200	C	0.79974300	-7.30489000	1.46002200
C	-2.46133600	-1.40780700	0.00058400	C	1.21736900	-7.67432300	-1.29572000
C	-3.69125000	-0.72987900	0.00077900	C	1.94117500	-6.63912700	-0.62892800
C	-3.69127700	0.72959000	0.00087800	C	1.71995900	-6.44400800	0.78799400
C	-4.92269400	1.40754400	0.00104000	C	2.40835300	-5.42549200	1.45121000
H	-4.92267700	2.49514800	0.00108400	H	2.24050600	-5.27865700	2.51580400
C	-6.15242200	0.72967200	0.00107800	C	3.31301900	-4.58178900	0.78341300
C	-6.15240900	-0.73011200	0.00099400	C	3.53567200	-4.77804400	-0.63949100
C	-4.92264200	-1.40791100	0.00086500	C	2.83818200	-5.80468900	-1.29982700
H	-2.46144000	2.49521400	0.00083000	H	0.63186400	-7.15580900	2.52441300
H	-0.00009400	2.49531500	0.00049200	H	-0.99506800	-8.98851600	2.48747400
H	0.00009100	-2.49531100	0.00019100	H	-0.24961300	-10.53424700	-0.94732900
H	-2.46126800	-2.49540900	0.00054600	H	1.36094900	-7.80098300	-2.36675100
H	-4.92254600	-2.49551500	0.00084400	H	3.00276200	-5.95088100	-2.36510300
C	-7.38371400	1.40759900	0.00115400	C	4.01164000	-3.55199300	1.44614300
C	-8.61418800	0.72995300	0.00112300	C	4.91145600	-2.71438800	0.77920900
C	-8.61419400	-0.73047900	0.00106300	C	5.13524400	-2.91151900	-0.64961800
C	-7.38369500	-1.40808500	0.00101700	C	4.44302600	-3.93213100	-1.30949400
H	-7.38363000	2.49520000	0.00117300	H	3.84383000	-3.40548200	2.51068000
H	-7.38356000	-2.49568700	0.00099900	H	4.60898400	-4.07954700	-2.37423700
C	11.07688600	0.73089100	-0.00083700	C	-7.04034800	-0.65457100	0.61695400
C	9.84423200	1.40839100	-0.00078300	C	-6.66416600	-1.86100300	1.24591800
C	9.84420500	-1.40786000	-0.00088700	C	-5.80932900	-1.39099300	-1.39449600
C	11.07685500	-0.73036300	-0.00087700	C	-6.59675300	-0.41077200	-0.75292600
H	9.84409800	2.49598100	-0.00077200	H	-6.99443000	-2.04230300	2.26629400
H	9.84408200	-2.49544900	-0.00088800	H	-5.47876400	-1.20886800	-2.41459500
C	-9.84420500	1.40786000	0.00110500	C	5.61289200	-1.68289300	1.44037800
C	-11.07685700	0.73036300	0.00100100	C	6.51495900	-0.84398100	0.77391300
C	-11.07688700	-0.73089200	0.00097400	C	6.73921300	-1.04142600	-0.65780000
C	-9.84423000	-1.40839100	0.00102300	C	6.04492500	-2.06335900	-1.31766300
H	-9.84408500	2.49545000	0.00110100	H	5.44555200	-1.53620200	2.50492800
H	-9.84409800	-2.49598100	0.00102400	H	6.21130100	-2.21044900	-2.38233100
C	-12.30404400	1.40829800	0.00088000	C	7.21414400	0.18305900	1.43417600
C	-13.54009000	0.73051400	0.00070400	C	8.12126600	1.02659100	0.76766100
C	-13.54014500	-0.73094900	0.00071500	C	8.34543900	0.82928400	-0.66420900
C	-12.30411900	-1.40878300	0.00086700	C	7.64614400	-0.19723800	-1.32449900
H	-12.30402600	2.49586600	0.00084900	H	7.04726700	0.32995000	2.49876300
H	-12.30413200	-2.49635200	0.00089300	H	7.81279900	-0.34392800	-2.38916300
C	-14.76378700	1.40852100	0.00047800	C	8.81668500	2.04778200	1.42790600
C	-16.00208500	0.72960400	0.00023500	C	9.72787400	2.89469700	0.76040600
C	-16.00216600	-0.72984700	0.00028500	C	9.95151900	2.69790000	-0.66885600
C	-14.76391300	-1.40886400	0.00054000	C	9.24840700	1.66782900	-1.33046500
H	-14.76412800	2.49606800	0.00041800	H	8.65026800	2.19488400	2.49253400
H	-14.76433000	-2.49641200	0.00059400	H	9.41531100	1.52157500	-2.39514300
C	13.54014500	0.73094900	-0.00077900	C	-8.19687500	1.51853300	0.62936300
C	12.30412100	1.40878300	-0.00082200	C	-7.82056200	0.31161200	1.25888200
C	12.30404500	-1.40829900	-0.00086300	C	-6.96529200	0.78169500	-1.38257700
C	13.54008900	-0.73051600	-0.00078300	C	-7.75331900	1.76225800	-0.74072600
H	12.30413300	2.49635100	-0.00083600	H	-8.15082700	0.13019100	2.27916900
H	12.30402400	-2.49586700	-0.00084100	H	-6.63479100	0.96348500	-2.40270300
C	16.00216800	0.72984600	-0.00055300	C	-9.35273100	3.69086000	0.64076500
C	14.76391400	1.40886200	-0.00069700	C	-8.97681400	2.48403600	1.27150500

C	14.76378800	-1.40852300	-0.00066600	C	-8.12155000	2.95396000	-1.37086300
C	16.00208700	-0.72960800	-0.00052000	C	-8.90978200	3.93420100	-0.72797700
H	14.76433100	2.49641100	-0.00073800	H	-9.30714000	2.30294500	2.29177800
H	14.76412700	-2.49607000	-0.00061800	H	-7.79134300	3.13578700	-2.39103200
C	18.45965200	0.72699500	-0.00017600	C	-10.50619000	5.86006400	0.65047700
C	17.22530400	1.40784900	-0.00040900	C	-10.13322000	4.65754200	1.28294100
C	17.22513000	-1.40775000	-0.00030600	C	-9.27863000	5.12698000	-1.35825100
C	18.45955100	-0.72704300	-0.00010700	C	-10.06483500	6.10248900	-0.71375700
H	17.22628400	2.49541300	-0.00047800	H	-10.46370700	4.47697500	2.30325600
H	17.22599000	-2.49531300	-0.00023100	H	-8.94879700	5.30910600	-2.37849400
C	-17.22512900	1.40774800	-0.00008800	C	10.42188500	3.91317000	1.42106900
C	-18.45954700	0.72704100	-0.00038200	C	11.33214800	4.75765800	0.75182300
C	-18.45964800	-0.72699700	-0.00029600	C	11.55477300	4.56178800	-0.67150900
C	-17.22530300	-1.40785100	0.00004800	C	10.85306900	3.53377000	-1.33517200
H	-17.22598800	2.49531100	-0.00017600	H	10.25592400	4.06055400	2.48575700
H	-17.22628200	-2.49541500	0.00013000	H	11.02026900	3.38799300	-2.39989400
C	-19.69060500	1.40564600	-0.00078700	C	12.03111800	5.78127100	1.41320400
C	-20.91025800	0.72414900	-0.00110800	C	12.93219600	6.61433700	0.74321200
C	-20.91037300	-0.72374100	-0.00098900	C	13.15375500	6.41942300	-0.67364000
C	-19.69081600	-1.40542200	-0.00058300	C	12.46142900	5.40269900	-1.33831900
H	-19.69120700	2.49335600	-0.00090100	H	11.86502600	5.92838400	2.47806500
H	-19.69157400	-2.49313300	-0.00047200	H	12.62843500	5.25676000	-2.40320300
C	-22.16485300	1.40741600	-0.00157200	C	13.64608400	7.65636700	1.40928300
C	-23.34587800	0.71347400	-0.00188400	C	14.52363000	8.45806000	0.72731400
C	-23.34599600	-0.71266000	-0.00173200	C	14.74183300	8.26610500	-0.66819600
C	-22.16508600	-1.40680100	-0.00130000	C	14.07677900	7.27748400	-1.34511400
H	-22.16134000	2.49449000	-0.00170500	H	13.47739200	7.80017900	2.47354400
H	-24.29211900	1.24679500	-0.00224400	H	15.05948200	9.24670400	1.24764300
H	-24.29232200	-1.24583000	-0.00196200	H	15.44104500	8.91104100	-1.19260000
H	-22.16175300	-2.49387600	-0.00116500	H	14.24093100	7.12849700	-2.40937600
C	20.91037700	0.72373700	0.00031500	C	-11.65648300	8.02399300	0.65973300
C	19.69081600	1.40542000	0.00001700	C	-11.29079600	6.83451700	1.29301500
C	19.69060500	-1.40564800	0.00018800	C	-10.43766400	7.30308200	-1.34430800
C	20.91026300	-0.72415000	0.00041600	C	-11.21694400	8.26539600	-0.69912700
H	19.69157800	2.49313000	-0.00008000	H	-11.62115700	6.65377400	2.31349300
H	19.69121100	-2.49335800	0.00028900	H	-10.10778000	7.48494000	-2.36473800
C	23.34599900	0.71265500	0.00085600	C	-12.79729800	10.17627400	0.66145100
C	22.16508700	1.40679700	0.00053000	C	-12.45337600	9.01841400	1.30665600
C	22.16485300	-1.40741700	0.00076800	C	-11.59932200	9.48745200	-1.33378700
C	23.34588100	-0.71347500	0.00099200	C	-12.36437100	10.41403900	-0.67702900
H	24.29232300	1.24582900	0.00101500	H	-13.40355600	10.92295000	1.16622100
H	22.16175600	2.49387200	0.00040800	H	-12.78160200	8.83421800	2.32653200
H	22.16134300	-2.49449200	0.00088900	H	-11.26777100	9.66558000	-2.35366300
H	24.29212000	-1.24679900	0.00126800	H	-12.64709500	11.33839000	-1.17251400
				H	-0.37414600	-10.26059500	1.44306900
				H	-0.41911300	-9.49309000	-2.36093700
				H	-2.62284700	-10.02231200	-1.33934200
				H	-2.59476700	-10.33334000	0.89976000
**[20]-acene**				**[20]-acene _H$_4$**			
C	7.38298300	0.73004200	0.00005100	C	-4.39735900	-4.40654800	0.60968400
C	6.15320500	1.40785900	0.00011300	C	-3.78153000	-5.50840100	1.23262200
C	4.92179900	0.72986500	0.00020000	C	-2.81868700	-6.30682300	0.59949400
C	4.92182400	-0.72964000	0.00024600	C	-2.43604500	-5.97960400	-0.78591300
C	6.15324100	-1.40759700	0.00018300	C	-3.04723600	-4.88068100	-1.40575900
C	7.38299800	-0.72974600	0.00008200	C	-4.01209800	-4.07748700	-0.76923800
C	3.69193900	1.40781400	0.00025200	C	-2.20834800	-7.38670200	1.22637300
C	3.69198900	-1.40763700	0.00034800	C	-1.49222900	-6.77337500	-1.42649100
C	2.46089100	-0.72965100	0.00040200	C	-0.86662900	-7.89335300	-0.80734000

C	2.46086100	0.72978000	0.00034400	C	-1.21872200	-8.19575700	0.59763400
C	1.23062400	1.40778000	0.00038600	C	-0.58939200	-9.21871500	1.23972000
H	1.23059200	2.49538000	0.00035700	H	-0.85404300	-9.42746800	2.27536600
C	-0.00001500	0.72971600	0.00046400	C	0.53233100	-10.03139300	0.67129800
C	0.00001800	-0.72970100	0.00053100	C	0.69417500	-9.89880800	-0.87812100
C	1.23068200	-1.40770800	0.00050200	C	0.01804300	-8.69364500	-1.46282500
H	3.69190100	2.49541600	0.00021600	H	-2.48310500	-7.62040500	2.25279500
H	6.15314100	2.49546300	0.00009900	H	-4.06623200	-5.75059400	2.25435700
H	6.15320500	-2.49520100	0.00023500	H	-2.76307100	-4.63660400	-2.42715700
H	3.69199300	-2.49523800	0.00039800	H	-1.21944100	-6.53865200	-2.45309100
H	1.23069800	-2.49530700	0.00057600	H	0.26359200	-8.46216400	-2.49828800
C	-1.23068300	1.40772500	0.00047200	C	1.86082900	-9.68154300	1.43082800
C	-2.46089300	0.72966400	0.00053000	C	2.59847300	-8.54155900	0.78981600
C	-2.46086100	-0.72976200	0.00060400	C	2.83862600	-8.67513300	-0.63072600
C	-1.23062400	-1.40776700	0.00060200	C	2.19502600	-9.86960900	-1.28076200
C	-3.69198800	1.40765100	0.00051800	C	3.03258000	-7.43672400	1.45798000
C	-3.69193600	-1.40780000	0.00065700	C	3.51959200	-7.70838100	-1.30640300
C	-4.92180000	-0.72985300	0.00063300	C	4.01840200	-6.53893200	-0.63886200
C	-4.92182500	0.72965400	0.00055700	C	3.75752100	-6.39379800	0.78824500
C	-6.15324100	1.40760900	0.00052200	C	4.21131900	-5.26765700	1.45107300
H	-6.15320800	2.49521400	0.00047100	H	4.01546100	-5.15892400	2.51539600
C	-7.38300100	0.72975800	0.00053300	C	4.92870900	-4.24078700	0.78474900
C	-7.38298300	-0.73003200	0.00060700	C	5.19161000	-4.38709100	-0.64535800
C	-6.15320300	-1.40784600	0.00066300	C	4.71651500	-5.54904400	-1.30665200
H	-3.69199600	2.49525200	0.00046200	H	2.83772900	-7.32755700	2.52256300
H	-1.23070800	2.49532400	0.00042100	H	1.64322400	-9.46716600	2.48192200
H	-1.23060000	-2.49536600	0.00066400	H	2.67409800	-10.80487400	-0.95849000
H	-3.69190200	-2.49540200	0.00072200	H	3.68393100	-7.79973700	-2.37799800
H	-6.15313900	-2.49545100	0.00073400	H	4.90950400	-5.65684300	-2.37167500
C	-8.61425600	1.40769000	0.00046700	C	5.38723900	-3.10461300	1.44728700
C	-9.84477200	0.73005500	0.00044600	C	6.10073000	-2.08598500	0.78094600
C	-9.84476500	-0.73036800	0.00051300	C	6.36505100	-2.23307700	-0.65137800
C	-8.61423700	-1.40798500	0.00059800	C	5.89553200	-3.38756900	-1.31273200
H	-8.61415800	2.49529100	0.00041100	H	5.19061100	-2.99552900	2.51132500
H	-8.61412400	-2.49558600	0.00065700	H	6.09116100	-3.49677800	-2.37697800
C	9.84476500	0.73037400	-0.00009800	C	-5.97235200	-2.50954400	0.62052600
C	8.61424000	1.40799300	-0.00003500	C	-5.35092600	-3.62162000	1.24518100
C	8.61425500	-1.40768100	0.00000200	C	-4.61166300	-2.99041200	-1.39218500
C	9.84476900	-0.73004800	-0.00008600	C	-5.58603700	-2.17987000	-0.75451600
H	8.61412700	2.49559500	-0.00005100	H	-5.63727000	-3.86523600	2.26604800
H	8.61415200	-2.49528300	0.00002600	H	-4.32571400	-2.74542300	-2.41282200
C	-11.07475900	1.40796500	0.00035800	C	6.56226200	-0.94398800	1.44295000
C	-12.30743600	0.73047000	0.00030600	C	7.27388700	0.06941400	0.77667500
C	-12.30744300	-0.73077400	0.00035900	C	7.53905300	-0.07812900	-0.65656900
C	-11.07476500	-1.40827500	0.00046900	C	7.07249200	-1.22792100	-1.31809100
H	-11.07462900	2.49555500	0.00030900	H	6.36528500	-0.83455600	2.50684700
H	-11.07464100	-2.49586600	0.00051900	H	7.26889500	-1.33733000	-2.38211600
C	-13.53461400	1.40839800	0.00020000	C	7.73773000	1.21529800	1.43841800
C	-14.77066300	0.73060600	0.00011400	C	8.44793400	2.22467300	0.77208400
C	-14.77068400	-0.73085000	0.00014400	C	8.71352300	2.07691100	-0.66108600
C	-13.53464700	-1.40867500	0.00027700	C	8.24906000	0.93081300	-1.32287100
H	-13.53459500	2.49596700	0.00016300	H	7.54053600	1.32499000	2.50223600
H	-13.53465800	-2.49624300	0.00031200	H	8.44596600	0.82130200	-2.38677100
C	-15.99437100	1.40859500	-0.00000600	C	8.91395000	3.37412100	1.43366800
C	-17.23265800	0.72965800	-0.00012700	C	9.62248800	4.37930700	0.76711800
C	-17.23269300	-0.72978900	-0.00012600	C	9.88815700	4.23151100	-0.66495000
C	-15.99443300	-1.40878400	0.00002200	C	9.42587700	3.08934000	-1.32710700
H	-15.99472400	2.49614200	-0.00002400	H	8.71663100	3.48404000	2.49744800
H	-15.99484000	-2.49633200	0.00003700	H	9.62314200	2.97980200	-2.39094300
C	12.30744200	0.73077700	-0.00021100	C	-7.54450500	-0.61642800	0.63103700

C	11.07476800	1.40827900	-0.00015800
C	11.07475800	-1.40796000	-0.00016200
C	12.30743200	-0.73046900	-0.00022200
H	11.07464300	2.49587000	-0.00015800
H	11.07462100	-2.49555100	-0.00015300
C	14.77068500	0.73084900	-0.00027500
C	13.53465100	1.40867400	-0.00023400
C	13.53461300	-1.40839800	-0.00029100
C	14.77066100	-0.73060900	-0.00031700
H	13.53466100	2.49624300	-0.00021600
H	13.53458900	-2.49596700	-0.00030300
C	17.23269500	0.72978200	-0.00028800
C	15.99443500	1.40878000	-0.00025800
C	15.99436900	-1.40860000	-0.00037800
C	17.23265800	-0.72966600	-0.00036600
H	15.99484500	2.49632800	-0.00021700
H	15.99472000	-2.49614800	-0.00041600
C	-18.45572500	1.40777300	-0.00025500
C	-19.69012300	0.72703400	-0.00040900
C	-19.69016900	-0.72700000	-0.00043700
C	-18.45581200	-1.40782300	-0.00028500
H	-18.45660600	2.49533600	-0.00025200
H	-18.45677100	-2.49538600	-0.00029100
C	-20.92120600	1.40560200	-0.00054100
C	-22.14083600	0.72406400	-0.00071600
C	-22.14089000	-0.72382300	-0.00076200
C	-20.92131400	-1.40546500	-0.00062000
H	-20.92183700	2.49331100	-0.00051900
H	-20.92204300	-2.49317500	-0.00064300
C	-23.39545500	1.40728700	-0.00085200
C	-24.57645400	0.71330100	-0.00102600
C	-24.57651100	-0.71283100	-0.00107000
C	-23.39557600	-1.40692900	-0.00094800
H	-23.39198200	2.49436100	-0.00082400
H	-25.52271500	1.24658500	-0.00112600
H	-25.52281700	-1.24603800	-0.00120600
H	-23.39221000	-2.49400300	-0.00098300
C	19.69017200	0.72698800	-0.00025900
C	18.45581400	1.40781400	-0.00023100
C	18.45572100	-1.40778200	-0.00042000
C	19.69012300	-0.72704600	-0.00037000
H	18.45677700	2.49537700	-0.00016500
H	18.45660200	-2.49534500	-0.00048500
C	22.14089300	0.72380500	-0.00021000
C	20.92131600	1.40545000	-0.00017500
C	20.92120200	-1.40561600	-0.00041900
C	22.14083700	-0.72408000	-0.00034300
H	20.92205000	2.49316000	-0.00008800
H	20.92183400	-2.49332500	-0.00050600
C	24.57651400	0.71280800	-0.00017500
C	23.39557800	1.40690900	-0.00012200
C	23.39545200	-1.40730500	-0.00038900
C	24.57645400	-0.71332200	-0.00030500
H	25.52282000	1.24601500	-0.00011100
H	23.39221600	2.49398300	-0.00002200
H	23.39197700	-2.49437900	-0.00048400
H	25.52271300	-1.24660900	-0.00034500

C	-6.92246600	-1.73153400	1.25834900
C	-6.17982400	-1.09807100	-1.38058500
C	-7.15786500	-0.28671200	-0.74105800
H	-7.20980400	-1.97581300	2.27867200
H	-5.89293200	-0.85265900	-2.40075700
C	-9.11467100	1.27325600	0.64110900
C	-8.49574800	0.16055800	1.27059100
C	-7.75100000	0.79535700	-1.36946000
C	-8.72798000	1.60283000	-0.72846900
H	-8.78371000	-0.08415700	2.29053600
H	-7.46351200	1.04099900	-2.38931500
C	-10.68351400	3.16029100	0.65073800
C	-10.07013300	2.05350500	1.28172000
C	-9.32416000	2.68904800	-1.35833800
C	-10.29699600	3.48958700	-0.71642600
H	-10.35850300	1.80847800	2.30141000
H	-9.03626700	2.93486300	-2.37797200
C	10.09121900	5.53336500	1.42859500
C	10.79706600	6.53267800	0.76153400
C	11.06244000	6.38505200	-0.66800800
C	10.60332600	5.24849900	-1.33079000
H	9.89386600	5.64352900	2.49238200
H	10.80087400	5.13903200	-2.39461600
C	11.27021900	7.69453900	1.42286300
C	11.97104800	8.68410600	0.75551600
C	12.23585500	8.53679600	-0.67053000
C	11.78204100	7.40983600	-1.33368000
H	11.07268300	7.80459300	2.48678400
H	11.97962700	7.30012800	-2.39763100
C	12.45248400	9.86265100	1.42050500
C	13.14071600	10.82311600	0.74004000
C	13.40136000	10.67812300	-0.66352200
C	12.96510700	9.57750200	-1.33991600
H	12.25342500	9.96969100	2.48380900
H	13.49853800	11.70904900	1.25668300
H	13.95169900	11.45697100	-1.18348200
H	13.16098700	9.46487200	-2.40323400
C	-12.25118400	5.04517100	0.65975800
C	-11.64539200	3.94700100	1.29193100
C	-10.89890400	4.58280100	-1.34713300
C	-11.86516900	5.37395500	-0.70448900
H	-11.93407200	3.70180600	2.31147700
H	-10.61077800	4.82880000	-2.36664000
C	-13.81755600	6.92795100	0.66843300
C	-13.22211000	5.84200700	1.30121600
C	-12.47579900	6.47755500	-1.33557100
C	-13.43226100	7.25607700	-0.69267100
H	-13.51078200	5.59642200	2.32079500
H	-12.18728500	6.72343600	-2.35512000
C	-15.37362100	8.80307700	0.66836100
C	-14.80441800	7.74153500	1.31361600
C	-14.05671700	8.37823000	-1.32739000
C	-14.99403100	9.12633000	-0.67240200
H	-16.12072500	9.41095900	1.17056000
H	-15.09066500	7.49325900	2.33253800
H	-13.76600400	8.62122100	-2.34632000
H	-15.45898800	9.97447100	-1.16672200
H	2.50305000	-10.57360100	1.41767700
H	2.28993100	-9.82061400	-2.37078600
H	0.33170100	-11.08733600	0.89777100

				H	0.24884000	-10.79323200	-1.34193900
**[21]-acene**				**[21]-acene _H_4**			
C	8.61343300	0.73000300	0.00084300	C	-5.94502600	-3.90293000	0.59757800
C	7.38372700	1.40780500	0.00073000	C	-5.56475000	-5.10631400	1.22486300
C	6.15233500	0.72984100	0.00069300	C	-4.76814000	-6.06945100	0.58849300
C	6.15234100	-0.72964400	0.00078600	C	-4.31481300	-5.81566200	-0.78252800
C	7.38372900	-1.40761800	0.00090100	C	-4.69222900	-4.61785700	-1.40680600
C	8.61343400	-0.72982700	0.00093600	C	-5.49186300	-3.64936800	-0.76750500
C	4.92246800	1.40782300	0.00056100	C	-4.38403700	-7.26027400	1.21969300
C	4.92247700	-1.40762300	0.00072900	C	-3.52647600	-6.77984500	-1.42478900
C	3.69141200	-0.72962400	0.00057300	C	-3.14182900	-7.99643900	-0.80368800
C	3.69139900	0.72982500	0.00048300	C	-3.56565400	-8.23461600	0.59085000
C	2.46118500	1.40784600	0.00031500	C	-3.15151000	-9.37205700	1.23764600
H	2.46122400	2.49543200	0.00025400	H	-3.46383100	-9.53001500	2.26871900
C	1.23052700	0.72987400	0.00021800	C	-2.18927800	-10.37612300	0.67513300
C	1.23054300	-0.72968400	0.00030400	C	-1.99674200	-10.27474200	-0.87465500
C	2.46120000	-1.40765000	0.00047800	C	-2.41233300	-8.95474800	-1.46019300
H	4.92246700	2.49540900	0.00052000	H	-4.71032100	-7.44053000	2.24156000
H	7.38365400	2.49539600	0.00065000	H	-5.90156100	-5.29370700	2.24203400
H	7.38363800	-2.49520800	0.00094200	H	-4.35524800	-4.42799400	-2.42339600
H	4.92246700	-2.49520800	0.00081200	H	-3.20111700	-6.59561100	-2.44609500
H	2.46123800	-2.49523500	0.00053800	H	-2.10990600	-8.76700100	-2.48911200
C	-0.00003100	1.40786200	0.00004800	C	-0.82614100	-10.27601900	1.44468700
C	-1.23042800	0.72988100	-0.00004400	C	0.12439500	-9.30770900	0.80103300
C	-1.23040600	-0.72971400	0.00003700	C	0.34422300	-9.49757300	-0.61677400
C	-0.00001000	-1.40768200	0.00020300	C	-0.51801200	-10.54607100	-1.26608600
C	-2.46127200	1.40783600	-0.00019700	C	0.76308400	-8.30504300	1.46584700
C	-2.46124600	-1.40768700	-0.00005600	C	1.20674500	-8.68864900	-1.29241300
C	-3.69137100	-0.72971200	-0.00020200	C	1.92102600	-7.63454100	-0.62816200
C	-3.69139500	0.72984200	-0.00027400	C	1.68378900	-7.43010400	0.79598300
C	-4.92251000	1.40777800	-0.00039900	C	2.34561200	-6.41063500	1.45586800
H	-4.92250800	2.49536800	-0.00041800	H	2.16772700	-6.25775200	2.51792000
C	-6.15234600	0.72984900	-0.00046700	C	3.25572400	-5.54963000	0.78950100
C	-6.15232200	-0.72976100	-0.00040900	C	3.49412900	-5.75487500	-0.63794600
C	-4.92247800	-1.40767000	-0.00027900	C	2.80436000	-6.80608700	-1.29611500
H	-2.46119100	2.49542700	-0.00025600	H	0.58621200	-8.15159900	2.52811700
H	0.00005100	2.49545500	-0.00001200	H	-1.00763200	-10.00987600	2.49090600
H	0.00008100	-2.49527500	0.00025500	H	-0.40757000	-10.52283300	-2.35553000
H	-2.46115200	-2.49527700	-0.00001300	H	1.35740200	-8.81873300	-2.36202600
H	-4.92246400	-2.49525900	-0.00021200	H	2.97967400	-6.95759900	-2.35887500
C	-7.38370300	1.40775400	-0.00057400	C	3.92429500	-4.52170300	1.44930800
C	-8.61352000	0.72996100	-0.00061400	C	4.82875800	-3.66740900	0.78299600
C	-8.61349500	-0.72991700	-0.00057500	C	5.06790000	-3.87307300	-0.64696600
C	-7.38367000	-1.40768800	-0.00048600	C	4.38460600	-4.91786900	-1.30542600
H	-7.38365800	2.49535400	-0.00061500	H	3.74618900	-4.36888100	2.51122400
H	-7.38361000	-2.49528800	-0.00046700	H	4.56177800	-5.07072800	-2.36753100
C	11.07516800	0.73031800	0.00094500	C	-7.11965800	-1.73981700	0.60905500
C	9.84473400	1.40790300	0.00086900	C	-6.73984500	-2.94390000	1.23616100
C	9.84472700	-1.40774400	0.00103900	C	-5.86638900	-2.45508300	-1.39403600
C	11.07516300	-0.73017800	0.00103400	C	-6.66661600	-1.48639500	-0.75490200
H	9.84454600	2.49550100	0.00081600	H	-7.07736700	-3.13253400	2.25283500
H	9.84451300	-2.49534200	0.00110900	H	-5.52852800	-2.26565300	-2.41041300
C	-9.84473100	1.40783900	-0.00066200	C	5.50142300	-2.63489200	1.44248500
C	-11.07524400	0.73023500	-0.00066300	C	6.40309300	-1.78475500	0.77634400
C	-11.07522000	-0.73023600	-0.00064800	C	6.64254900	-1.99048900	-0.65491800
C	-9.84469800	-1.40781700	-0.00061700	C	5.96250400	-3.03122700	-1.31374100
H	-9.84461800	2.49543700	-0.00068300	H	5.32342600	-2.48214100	2.50438500
H	-9.84457100	-2.49541500	-0.00061200	H	6.13998800	-3.18395900	-2.37574800

C	-12.30524400	1.40810600	-0.00064800	C	7.07866900	-0.74961400	1.43578200
C	-13.53785600	0.73062200	-0.00062000	C	7.97851200	0.09761400	0.76972400
C	-13.53783300	-0.73066400	-0.00063600	C	8.21802100	-0.10800000	-0.66198500
C	-12.30521200	-1.40812700	-0.00065500	C	7.53993300	-1.14572300	-1.32122000
H	-12.30508500	2.49569000	-0.00063200	H	6.90081400	-0.59697300	2.49770200
H	-12.30504000	-2.49571100	-0.00065400	H	7.71754400	-1.29827000	-2.38320600
C	-14.76509400	1.40850800	-0.00054900	C	8.65652900	1.13473100	1.42919700
C	-16.00105800	0.73073900	-0.00048900	C	9.55474900	1.97931000	0.76312900
C	-16.00103700	-0.73081700	-0.00054500	C	9.79413000	1.77393700	-0.66824800
C	-14.76506400	-1.40856800	-0.00062200	C	9.11771900	0.73898100	-1.32793200
H	-14.76501800	2.49607000	-0.00051600	H	8.47883200	1.28727100	2.49115500
H	-14.76497700	-2.49612900	-0.00065400	H	9.29538400	0.58663800	-2.38993200
C	13.53780900	0.73070800	0.00091500	C	-8.29430900	0.42300000	0.62127200
C	12.30526100	1.40817100	0.00092000	C	-7.91488700	-0.78051100	1.24847000
C	12.30524500	-1.40805800	0.00107300	C	-7.04110500	-0.29171800	-1.38254200
C	13.53780000	-0.73062300	0.00099000	C	-7.84111700	0.67643200	-0.74354400
H	12.30503700	2.49575700	0.00089200	H	-8.25231900	-0.96938000	2.26507400
H	12.30499000	-2.49564400	0.00115600	H	-6.70324200	-0.10278400	-2.39897200
C	16.00105000	0.73079800	0.00069700	C	-9.46924900	2.58665100	0.63327600
C	14.76510000	1.40856500	0.00081000	C	-9.08942400	1.38221700	1.26090400
C	14.76507500	-1.40851400	0.00093000	C	-8.21550400	1.87088900	-1.37162900
C	16.00103800	-0.73078300	0.00075000	C	-9.01600200	2.84004100	-0.73235200
H	14.76502600	2.49612700	0.00080400	H	-9.42678100	1.19350100	2.27750400
H	14.76496400	-2.49607600	0.00100800	H	-7.87784900	2.05961600	-2.38811700
C	18.46308400	0.72977600	0.00028300	C	-10.64334200	4.75005700	0.64411900
C	17.22481600	1.40868200	0.00050400	C	-10.26339200	3.54450200	1.27287300
C	17.22478200	-1.40870800	0.00056900	C	-9.38958200	4.03295400	-1.36052000
C	18.46307000	-0.72984400	0.00030100	C	-10.19074400	5.00302400	-0.72015600
H	17.22519400	2.49622700	0.00051100	H	-10.60082400	3.35624800	2.28949400
H	17.22511900	-2.49625200	0.00061900	H	-9.05229500	4.22183500	-2.37706400
C	-17.22482000	1.40864700	-0.00034900	C	10.23536400	3.01862500	1.42266600
C	-18.46306800	0.72975500	-0.00025800	C	11.13146200	3.86000000	0.75643800
C	-18.46304900	-0.72986200	-0.00036400	C	11.37055600	3.65497000	-0.67370600
C	-17.22479300	-1.40874000	-0.00050900	C	10.69631100	2.62330600	-1.33392500
H	-17.22516200	2.49619200	-0.00028700	H	10.05782900	3.17110800	2.48467400
H	-17.22512800	-2.49628400	-0.00057800	H	10.87401300	2.47117300	-2.39595900
C	-19.68613700	1.40773700	-0.00004300	C	11.81558600	4.90272800	1.41603200
C	-20.92056000	0.72702200	0.00006600	C	12.70811200	5.73919800	0.74934600
C	-20.92054300	-0.72715200	-0.00009300	C	12.94670100	5.53466500	-0.67820600
C	-19.68611200	-1.40785600	-0.00030600	C	12.27615200	4.50788900	-1.33918200
H	-19.68713500	2.49530100	0.00006000	H	11.63823700	5.05524100	2.47810800
H	-19.68710700	-2.49542000	-0.00040900	H	12.45392900	4.35599300	-2.40127800
C	-22.15159600	1.40545800	0.00033800	C	13.39810600	6.78833600	1.40889900
C	-23.37127700	0.72387500	0.00044200	C	14.28392700	7.61643100	0.74196400
C	-23.37126100	-0.72402400	0.00024600	C	14.52187200	7.41248500	-0.68204900
C	-22.15157400	-1.40559800	-0.00001700	C	13.85807500	6.39410000	-1.34349000
H	-22.15237600	2.49316700	0.00049000	H	13.22066400	6.94064200	2.47114700
H	-22.15235300	-2.49330700	-0.00014100	H	14.03571000	6.24210400	-2.40575300
C	20.92058400	0.72700000	-0.00030900	C	-11.81466300	6.91031900	0.65326500
C	19.68613400	1.40773900	0.00001300	C	-11.43732200	5.70810000	1.28372500
C	19.68609300	-1.40785400	0.00000300	C	-10.56428700	6.19600900	-1.34831700
C	20.92056700	-0.72716200	-0.00033200	C	-11.36377900	7.16228500	-0.70625000
H	19.68717400	2.49530300	0.00004000	H	-11.77493500	5.52043300	2.30040600
H	19.68708600	-2.49541700	0.00001800	H	-10.22740600	6.38526700	-2.36494100
C	23.37129700	0.72380900	-0.00105100	C	-12.98246800	9.06506200	0.66202400
C	22.15160300	1.40541800	-0.00064500	C	-12.61241800	7.87549800	1.29329500
C	22.15155600	-1.40563100	-0.00073000	C	-11.74093900	8.36247100	-1.33468000
C	23.37127800	-0.72407200	-0.00110600	C	-12.53352500	9.31592000	-0.69187000
H	22.15241900	2.49312700	-0.00060200	H	-12.94990700	7.68766600	2.31014500
H	22.15232200	-2.49334000	-0.00075500	H	-11.40400100	8.55148700	-2.35149000

C	25.80687600	0.71282600	-0.00189000	C	-14.14050200	11.20806400	0.66340100
C	24.62583300	1.40691800	-0.00144000	C	-13.79263400	10.04998400	1.30650500
C	24.62578400	-1.40723600	-0.00156600	C	-12.92025400	10.53743100	-1.32444900
C	25.80685500	-0.71319400	-0.00195200	C	-13.69833400	11.45513000	-0.67012100
H	26.75310500	1.24611900	-0.00219900	H	-14.75696800	11.94757200	1.16634300
H	24.62247300	2.49397800	-0.00138300	H	-14.12794000	9.85869400	2.32275900
H	24.62237100	-2.49429500	-0.00161200	H	-12.58160500	10.72270500	-2.34070800
H	26.75306500	-1.24651900	-0.00231300	H	-13.98426100	12.37932300	-1.16404500
C	-24.62581900	1.40700400	0.00074700	C	14.98529200	8.68011600	1.40521500
C	-25.80686100	0.71293400	0.00085100	C	15.85315500	9.48221600	0.72519600
C	-25.80684700	-0.71310100	0.00064800	C	16.08731300	9.28152700	-0.67630400
C	-24.62579800	-1.40716200	0.00034300	C	15.44582600	8.28542800	-1.35103500
H	-24.62242800	2.49406300	0.00089900	H	14.80581900	8.82970800	2.46686600
H	-26.75308900	1.24622700	0.00109300	H	16.37626800	10.28262100	1.24049400
H	-26.75306700	-1.24640900	0.00073000	H	16.78334100	9.93373900	-1.19591700
H	-24.62240800	-2.49422200	0.00017500	H	15.62114100	8.13097600	-2.41268700
				H	-0.36517300	-11.27397700	1.44972100
				H	-0.23448000	-11.55534900	-0.93592800
				H	-2.58228600	-11.37718400	0.89568500
				H	-2.61153700	-11.05904600	-1.34353200

Table S9. Cartesian coordinates of the optimized geometries of [*n*]-cyclacene at UCAM-B3LYP-D3(BJ)/6-31G(d) and UωB97X-D/6-31G(d) level of theory.

**UCAM-B3LYP/D3(BJ)/6-31G(d)**				**UωB97X-D /6-31G(d)**			
**[6]-cyclcacene**				**[6]-cyclacene**			
C	0.00000000	2.39381600	0.72190500	C	-2.39400000	0.00000000	0.72258900
C	1.20385300	2.08510800	1.40101300	C	-2.08647900	1.20464900	1.40309300
C	0.00000000	2.39381600	-0.72190500	C	-2.39400000	0.00000000	-0.72258900
C	-1.20385300	2.08510800	1.40101300	C	-2.08647900	-1.20464900	1.40309300
C	2.07324600	1.19698000	0.72190600	C	-1.19714300	2.07348200	0.72257800
H	1.18117500	2.04581500	2.48667200	H	-2.05188800	1.18467800	2.48989400
C	1.20385300	2.08510800	-1.40101300	C	-2.08647900	1.20464900	-1.40309300
C	-1.20385300	2.08510800	-1.40101300	C	-2.08647900	-1.20464900	-1.40309300
C	-2.07324600	1.19698000	0.72190600	C	-1.19714300	-2.07348200	0.72257800
H	-1.18117500	2.04581500	2.48667200	H	-2.05188800	-1.18467800	2.48989400
C	2.07324600	1.19698000	-0.72190600	C	-1.19714300	2.07348200	-0.72257800
C	2.40757900	0.00000000	1.40102600	C	0.00000000	2.40917500	1.40305300
C	2.40757900	0.00000000	-1.40102600	C	0.00000000	2.40917500	-1.40305300
C	2.07324600	-1.19698000	0.72190600	C	1.19714300	2.07348200	0.72257800
H	2.36209400	0.00000000	2.48668000	H	0.00000000	2.36913400	2.48985100
H	1.18117500	2.04581500	-2.48667200	H	-2.05188800	1.18467800	-2.48989400
C	2.07324600	-1.19698000	-0.72190600	C	1.19714300	2.07348200	-0.72257800
H	2.36209400	0.00000000	-2.48668000	H	0.00000000	2.36913400	-2.48985100
C	-2.07324600	1.19698000	-0.72190600	C	-1.19714300	-2.07348200	-0.72257800
H	-1.18117500	2.04581500	-2.48667200	H	-2.05188800	-1.18467800	-2.48989400
C	1.20385300	-2.08510800	1.40101300	C	2.08647900	1.20464900	1.40309300
C	1.20385300	-2.08510800	-1.40101300	C	2.08647900	1.20464900	-1.40309300
C	0.00000000	-2.39381600	-0.72190500	C	2.39400000	0.00000000	-0.72258900
H	1.18117500	-2.04581500	-2.48667200	H	2.05188800	1.18467800	-2.48989400
H	1.18117500	-2.04581500	2.48667200	H	2.05188800	1.18467800	2.48989400
C	0.00000000	-2.39381600	0.72190500	C	2.39400000	0.00000000	0.72258900
C	-1.20385300	-2.08510800	1.40101300	C	2.08647900	-1.20464900	1.40309300
C	-1.20385300	-2.08510800	-1.40101300	C	2.08647900	-1.20464900	-1.40309300
C	-2.07324600	-1.19698000	0.72190600	C	1.19714300	-2.07348200	0.72257800
H	-1.18117500	-2.04581500	2.48667200	H	2.05188800	-1.18467800	2.48989400
C	-2.07324600	-1.19698000	-0.72190600	C	1.19714300	-2.07348200	-0.72257800
H	-1.18117500	-2.04581500	-2.48667200	H	2.05188800	-1.18467800	-2.48989400
C	-2.40757900	0.00000000	1.40102600	C	0.00000000	-2.40917500	1.40305300
C	-2.40757900	0.00000000	-1.40102600	C	0.00000000	-2.40917500	-1.40305300
H	-2.36209400	0.00000000	2.48668000	H	0.00000000	-2.36913400	2.48985100
H	-2.36209400	0.00000000	-2.48668000	H	0.00000000	-2.36913400	-2.48985100
**[7]-cyclcacene**				**[7]-cyclcacene**			
C	0.88520900	2.63285000	-0.72342800	C	0.37931800	2.75255600	-0.72415400
C	1.94827000	1.99654800	-1.40380600	C	1.54321000	2.32609400	-1.40592700
C	0.88520900	2.63282400	0.72351500	C	0.37932200	2.75253100	0.72424100
C	-0.34630900	2.76776800	-1.40381600	C	-0.85651300	2.65669500	-1.40592300
C	2.61060700	0.94959400	-0.72343800	C	2.38887500	1.41981400	-0.72418700
H	1.91853400	1.96609700	-2.48955700	H	1.52215400	2.29440000	-2.49279600
C	1.94822500	1.99651700	1.40388900	C	1.54317400	2.32605400	1.40601100
C	-0.34635800	2.76771400	1.40389100	C	-0.85654800	2.65663500	1.40599900
C	-1.50664200	2.33358800	-0.72344200	C	-1.91579800	2.01287900	-0.72419300
H	-0.34104400	2.72559100	-2.48957000	H	-0.84483300	2.62049900	-2.49278900
C	2.61060000	0.94956200	0.72349800	C	2.38887400	1.41978300	0.72424800
C	2.77590500	-0.27827800	-1.40383200	C	2.78099700	0.24385700	-1.40596500

H	1.91848200	1.96602100	2.48963800	H	1.52211900	2.29431700	2.49287800
C	-1.50664300	2.33357200	0.72348900	C	-1.91579700	2.01286300	0.72424100
H	-0.34108700	2.72550500	2.48964300	H	-0.84485900	2.62040800	2.49286400
C	-2.38018500	1.45504500	-1.40383100	C	-2.61139800	0.98688000	-1.40597400
C	2.77588200	-0.27829700	1.40385900	C	2.78097800	0.24383300	1.40599400
C	2.37013700	-1.44883900	-0.72348200	C	2.59952800	-0.98234200	-0.72422900
H	2.73361700	-0.27400600	-2.48958700	H	2.74304300	0.24055300	-2.49283000
C	-2.38022000	1.45497600	1.40384200	C	-2.61141800	0.98680800	1.40598600
C	-2.76423400	0.27713100	-0.72348900	C	-2.76831500	-0.24274700	-0.72423900
H	-2.34384800	1.43285000	-2.48958100	H	-2.57581000	0.97345100	-2.49284300
C	2.37012200	-1.44886900	0.72347000	C	2.59951900	-0.98237400	0.72421800
H	2.73357700	-0.27406600	2.48961400	H	2.74301500	0.24048500	2.49285800
C	1.51306000	-2.34349100	-1.40387800	C	1.92456000	-2.02206000	-1.40601000
C	-2.76424300	0.27712000	0.72346200	C	-2.76832200	-0.24276000	0.72421200
H	-2.34388600	1.43276800	2.48959200	H	-2.57583100	0.97336400	2.49285400
C	-2.62167400	-0.95355500	-1.40387800	C	-2.39980600	-1.42625200	-1.40602000
C	1.51306400	-2.34352700	1.40383100	C	1.92456900	-2.02209500	1.40596200
H	1.48998500	-2.30771600	-2.48962900	H	1.89836700	-1.99449900	-2.49287900
C	0.34489300	-2.75605800	-0.72351300	C	0.85263200	-2.64451100	-0.72426700
H	1.48996800	-2.30779100	2.48958300	H	1.89836200	-1.99457700	2.49283200
C	0.34487300	-2.75608200	0.72344000	C	0.85261700	-2.64453900	0.72419300
C	-0.88894400	-2.64396000	-1.40388800	C	-0.38104500	-2.76513200	-1.40604300
C	-0.88894100	-2.64402200	1.40380000	C	-0.38103300	-2.76519000	1.40595400
C	-2.62168800	-0.95362800	1.40381600	C	-2.39980800	-1.42632400	1.40595700
C	-1.93998500	-1.98806900	-0.72351300	C	-1.53615200	-2.31544100	-0.72426900
H	-0.87538800	-2.60367100	-2.48964200	H	-0.37585200	-2.72744700	-2.49290900
C	-1.94000300	-1.98808400	0.72343000	C	-1.53616500	-2.31545800	0.72418600
H	-0.87541800	-2.60376300	2.48955600	H	-0.37586400	-2.72754100	2.49282100
H	-2.58168300	-0.93900800	-2.48962900	H	-2.36716100	-1.40683000	-2.49289000
H	-2.58172100	-0.93909600	2.48956900	H	-2.36718200	-1.40692100	2.49282900
**[8]-cyclcacene**				**[8]-cyclcacene**			
C	-1.21306800	2.92860900	1.40363500	C	1.21404800	2.93098000	1.40570100
C	-2.23396400	2.23396400	0.72442700	C	0.00000000	3.16117900	0.72502800
H	-1.19899900	2.89462400	2.48966000	H	1.20160400	2.90094100	2.49282200
C	0.00000000	3.15933600	0.72442600	C	2.23529400	2.23529400	0.72503200
C	-2.23396400	2.23396400	-0.72442700	C	0.00000000	3.16117900	-0.72502800
C	-2.92860900	1.21306800	1.40363500	C	-1.21404800	2.93098000	1.40570100
C	0.00000000	3.15933600	-0.72442600	C	2.23529400	2.23529400	-0.72503200
C	1.21306800	2.92860900	1.40363500	C	2.93098000	1.21404800	1.40570100
C	-1.21306800	2.92860900	-1.40363500	C	1.21404800	2.93098000	-1.40570100
C	-2.92860900	1.21306800	-1.40363500	C	-1.21404800	2.93098000	-1.40570100
C	-3.15933600	0.00000000	0.72442600	C	-2.23529400	2.23529400	0.72503200
H	-2.89462400	1.19899900	2.48966000	H	-1.20160400	2.90094100	2.49282200
C	1.21306800	2.92860900	-1.40363500	C	2.93098000	1.21404800	-1.40570100
C	2.23396400	2.23396400	0.72442700	C	3.16117900	0.00000000	0.72502800
H	1.19899900	2.89462400	2.48966000	H	2.90094100	1.20160400	2.49282200
H	-1.19899900	2.89462400	-2.48966000	H	1.20160400	2.90094100	-2.49282200
C	-3.15933600	0.00000000	-0.72442600	C	-2.23529400	2.23529400	-0.72503200
H	-2.89462400	1.19899900	-2.48966000	H	-1.20160400	2.90094100	-2.49282200
C	-2.92860900	-1.21306800	1.40363500	C	-2.93098000	1.21404800	1.40570100
C	2.23396400	2.23396400	-0.72442700	C	3.16117900	0.00000000	-0.72502800
H	1.19899900	2.89462400	-2.48966000	H	2.90094100	1.20160400	-2.49282200
C	2.92860900	1.21306800	1.40363500	C	2.93098000	-1.21404800	1.40570100
C	-2.92860900	-1.21306800	-1.40363500	C	-2.93098000	1.21404800	-1.40570100
H	-2.89462400	-1.19899900	2.48966000	H	-2.90094100	1.20160400	2.49282200
C	-2.23396400	-2.23396400	0.72442700	C	-3.16117900	0.00000000	0.72502800
C	2.92860900	1.21306800	-1.40363500	C	2.93098000	-1.21404800	-1.40570100
C	3.15933600	0.00000000	0.72442600	C	2.23529400	-2.23529400	0.72503200

H	2.89462400	1.19899900	2.48966000	H	2.90094100	-1.20160400	2.49282200
H	-2.89462400	-1.19899900	-2.48966000	H	-2.90094100	1.20160400	-2.49282200
C	-2.23396400	-2.23396400	-0.72442700	C	-3.16117900	0.00000000	-0.72502800
C	-1.21306800	-2.92860900	1.40363500	C	-2.93098000	-1.21404800	1.40570100
C	-1.21306800	-2.92860900	-1.40363500	C	-2.93098000	-1.21404800	-1.40570100
C	3.15933600	0.00000000	-0.72442600	C	2.23529400	-2.23529400	-0.72503200
H	2.89462400	1.19899900	-2.48966000	H	2.90094100	-1.20160400	-2.49282200
C	0.00000000	-3.15933600	0.72442600	C	-2.23529400	-2.23529400	0.72503200
H	-1.19899900	-2.89462400	2.48966000	H	-2.90094100	-1.20160400	2.49282200
C	0.00000000	-3.15933600	-0.72442600	C	-2.23529400	-2.23529400	-0.72503200
H	-1.19899900	-2.89462400	-2.48966000	H	-2.90094100	-1.20160400	-2.49282200
C	1.21306800	-2.92860900	-1.40363500	C	-1.21404800	-2.93098000	-1.40570100
C	1.21306800	-2.92860900	1.40363500	C	-1.21404800	-2.93098000	1.40570100
C	2.23396400	-2.23396400	0.72442700	C	0.00000000	-3.16117900	0.72502800
H	1.19899900	-2.89462400	2.48966000	H	-1.20160400	-2.90094100	2.49282200
C	2.23396400	-2.23396400	-0.72442700	C	0.00000000	-3.16117900	-0.72502800
H	1.19899900	-2.89462400	-2.48966000	H	-1.20160400	-2.90094100	-2.49282200
C	2.92860900	-1.21306800	1.40363500	C	1.21404800	-2.93098000	1.40570100
C	2.92860900	-1.21306800	-1.40363500	C	1.21404800	-2.93098000	-1.40570100
H	2.89462400	-1.19899900	2.48966000	H	1.20160400	-2.90094100	2.49282200
H	2.89462400	-1.19899900	-2.48966000	H	1.20160400	-2.90094100	-2.49282200
**[9]-cyclcacene**				**[9]-cyclcacene**			
C	-0.61620500	3.49437900	0.72381900	C	3.50863200	0.73076200	-0.61713300
C	-1.77934100	3.08165900	1.40366700	C	3.11344900	1.40912900	-1.77538200
C	-0.61620500	3.49437900	-0.72381900	C	3.50863200	-0.73076200	-0.61713300
C	0.61794100	3.50424000	1.40366500	C	3.55297700	1.41050500	0.61880600
C	-2.71849700	2.28091700	0.72382100	C	2.28242800	0.72350500	-2.71415500
H	-1.76281300	3.05302300	2.48979200	H	3.09676900	2.49629300	-1.76821200
C	-1.77934100	3.08165900	-1.40366700	C	3.11344900	-1.40912900	-1.77538200
C	0.61794100	3.50424000	-1.40366500	C	3.55297700	-1.41050500	0.61880600
C	1.77430700	3.07292900	0.72381800	C	3.08161000	0.72810100	1.77490700
H	0.61220000	3.47168500	2.48979100	H	3.54428100	2.49771700	0.61805500
C	-2.71849700	2.28091700	-0.72382100	C	2.28242800	-0.72350500	-2.71415500
C	-3.34426200	1.21712000	1.40366900	C	1.21799600	1.40228900	-3.30404700
H	-1.76281300	3.05302300	-2.48979200	H	3.09676900	-2.49629300	-1.76821200
C	1.77430700	3.07292900	-0.72381800	C	3.08161000	-0.72810100	1.77490700
H	0.61220000	3.47168500	-2.48979100	H	3.54428100	-2.49771700	0.61805500
C	2.72619500	2.28737200	1.40367300	C	2.29881000	1.40597200	2.70540600
C	-3.34426200	1.21712000	-1.40366900	C	1.21799600	-1.40228900	-3.30404700
C	-3.54882100	0.00000000	0.72382100	C	0.00000000	0.71701800	-3.53242300
H	-3.31327900	1.20583900	2.48979800	H	1.19992900	2.48887700	-3.26002300
C	-3.54882100	0.00000000	-0.72382100	C	0.00000000	-0.71701800	-3.53242300
H	-3.31327900	1.20583900	-2.48979800	H	1.19992900	-2.48887700	-3.26002300
C	-3.34426200	-1.21712000	1.40366900	C	-1.21799600	1.40228900	-3.30404700
C	-3.34426200	-1.21712000	-1.40366900	C	-1.21799600	-1.40228900	-3.30404700
C	2.72619500	2.28737200	-1.40367300	C	2.29881000	-1.40597200	2.70540600
H	-3.31327900	-1.20583900	2.48979800	H	-1.19992900	2.48887700	-3.26002300
C	-2.71849700	-2.28091700	0.72382100	C	-2.28242800	0.72350500	-2.71415500
H	-3.31327900	-1.20583900	-2.48979800	H	-1.19992900	-2.48887700	-3.26002300
C	-2.71849700	-2.28091700	-0.72382100	C	-2.28242800	-0.72350500	-2.71415500
C	-1.77934100	-3.08165900	-1.40366700	C	-3.11344900	-1.40912900	-1.77538200
C	-1.77934100	-3.08165900	1.40366700	C	-3.11344900	1.40912900	-1.77538200
C	-0.61620500	-3.49437900	0.72381900	C	-3.50863200	0.73076200	-0.61713300
H	-1.76281300	-3.05302300	2.48979200	H	-3.09676900	2.49629300	-1.76821200
C	-0.61620500	-3.49437900	-0.72381900	C	-3.50863200	-0.73076200	-0.61713300
H	-1.76281300	-3.05302300	-2.48979200	H	-3.09676900	-2.49629300	-1.76821200
C	0.61794100	-3.50424000	1.40366500	C	-3.55297700	1.41050500	0.61880600
C	0.61794100	-3.50424000	-1.40366500	C	-3.55297700	-1.41050500	0.61880600

C	1.77430700	-3.07292900	0.72381800	C	-3.08161000	0.72810100	1.77490700
H	0.61220000	-3.47168500	2.48979100	H	-3.54428100	2.49771700	0.61805500
C	1.77430700	-3.07292900	-0.72381800	C	-3.08161000	-0.72810100	1.77490700
H	0.61220000	-3.47168500	-2.48979100	H	-3.54428100	-2.49771700	0.61805500
C	2.72619500	-2.28737200	1.40367300	C	-2.29881000	1.40597200	2.70540600
C	2.72619500	-2.28737200	-1.40367300	C	-2.29881000	-1.40597200	2.70540600
H	2.70092700	-2.26617700	2.48980100	H	-2.27528000	2.49292000	2.68161400
C	3.33481500	-1.21367400	0.72382300	C	-1.21166700	0.71893000	3.32255400
H	2.70092700	-2.26617700	-2.48980100	H	-2.27528000	-2.49292000	2.68161400
C	3.33481500	-1.21367400	-0.72382300	C	-1.21166700	-0.71893000	3.32255400
C	3.55892300	0.00000000	-1.40367300	C	0.00000000	-1.40058300	3.51047100
C	3.55892300	0.00000000	1.40367300	C	0.00000000	1.40058300	3.51047100
C	3.33481500	1.21367400	0.72382300	C	1.21166700	0.71893000	3.32255400
H	3.52589100	0.00000000	2.48980000	H	0.00000000	2.48698600	3.45736400
C	3.33481500	1.21367400	-0.72382300	C	1.21166700	-0.71893000	3.32255400
H	3.52589100	0.00000000	-2.48980000	H	0.00000000	-2.48698600	3.45736400
H	2.70092700	2.26617700	2.48980100	H	2.27528000	2.49292000	2.68161400
H	2.70092700	2.26617700	-2.48980100	H	2.27528000	-2.49292000	2.68161400
**[10]-cyclcacene**				**[10]-cyclcacene**			
C	0.00000000	3.93363800	0.72497100	C	1.08009200	3.78510600	-0.72580500
C	0.00000000	3.93363800	-0.72497100	C	1.08014600	3.78517700	0.72535300
C	-1.21830900	3.74970100	1.40399300	C	2.20220200	3.27392000	-1.40651400
C	1.21830900	3.74970100	1.40399300	C	-0.14289400	3.94295600	-1.40645200
C	-1.21830900	3.74970100	-1.40399300	C	2.20232800	3.27404900	1.40601900
C	1.21830900	3.74970100	-1.40399300	C	-0.14276700	3.94308900	1.40608700
H	-1.20907900	3.72128900	2.49027000	H	2.18749900	3.25205400	-2.49383600
C	-2.31204700	3.18239400	0.72497400	C	3.09884500	2.42742500	-0.72581000
H	-1.20907900	3.72128900	-2.49027000	H	2.18771100	3.25229100	2.49334500
C	-2.31204700	3.18239400	-0.72497400	C	3.09890100	2.42750000	0.72532300
C	-3.18952900	2.31741700	1.40398400	C	3.70623100	1.35430800	-1.40646500
C	-3.74081300	1.21551900	0.72496700	C	3.93396600	0.14245500	-0.72573500
C	-3.74081300	1.21551900	-0.72496700	C	3.93402600	0.14253100	0.72539800
C	-3.94235500	0.00000000	1.40398900	C	3.79449000	-1.08274300	-1.40636500
C	-3.94235500	0.00000000	-1.40398900	C	3.79460200	-1.08261900	1.40615800
C	-3.18952900	2.31741700	-1.40398400	C	3.70635200	1.35443300	1.40603100
C	-3.74081300	-1.21551900	-0.72496700	C	3.26644100	-2.19690300	0.72554900
C	-3.18952900	-2.31741700	-1.40398400	C	2.43342500	-3.10603200	1.40631900
C	-2.31204700	-3.18239400	-0.72497400	C	1.35119600	-3.69710000	0.72570400
C	-2.31204700	-3.18239400	0.72497400	C	1.35113100	-3.69717400	-0.72545900
C	-1.21830900	-3.74970100	1.40399300	C	0.14277700	-3.94308900	-1.40608600
C	0.00000000	-3.93363800	0.72497100	C	-1.08014900	-3.78517700	-0.72535300
H	-1.20907900	-3.72128900	2.49027000	H	0.14177400	-3.91682400	-2.49340800
C	-3.18952900	-2.31741700	1.40398400	C	2.43332100	-3.10616400	-1.40622000
C	-3.74081300	-1.21551900	0.72496700	C	3.26637800	-2.19697900	-0.72560600
H	-3.16540900	-2.29988300	2.49026300	H	2.41704200	-3.08548200	-2.49354100
C	-1.21830900	-3.74970100	-1.40399300	C	0.14288000	-3.94295300	1.40645300
H	-3.16540900	-2.29988300	-2.49026300	H	2.41723700	-3.08524000	2.49364000
C	0.00000000	-3.93363800	-0.72497100	C	-1.08008400	-3.78510900	0.72580500
C	1.21830900	-3.74970100	1.40399300	C	-2.20231900	-3.27405200	-1.40602000
H	-1.20907900	-3.72128900	-2.49027000	H	0.14197000	-3.91658300	2.49377300
H	-3.91253000	0.00000000	-2.49026800	H	3.76937100	-1.07534600	2.49348200
H	-3.91253000	0.00000000	2.49026800	H	3.76916700	-1.07558000	-2.49368800
C	1.21830900	-3.74970100	-1.40399300	C	-2.20221600	-3.27391300	1.40651300
H	-3.16540900	2.29988300	2.49026300	H	3.68149900	1.34522900	-2.49378700
H	-3.16540900	2.29988300	-2.49026300	H	3.68171000	1.34546400	2.49335500
H	1.20907900	-3.72128900	2.49027000	H	-2.18770100	-3.25229200	-2.49334500
C	2.31204700	-3.18239400	0.72497400	C	-3.09890200	-2.42749700	-0.72532400
H	1.20907900	-3.72128900	-2.49027000	H	-2.18750600	-3.25205300	2.49383600

C	2.31204700	-3.18239400	-0.72497400	C	-3.09884000	-2.42743300	0.72581000
C	2.31204700	3.18239400	0.72497400	C	-1.35118800	3.69710300	-0.72570400
H	1.20907900	3.72128900	2.49027000	H	-0.14197700	3.91659000	-2.49377300
C	3.18952900	2.31741700	1.40398400	C	-2.43343800	3.10603100	-1.40631800
C	2.31204700	3.18239400	-0.72497400	C	-1.35113400	3.69716900	0.72546200
C	3.74081300	1.21551900	0.72496700	C	-3.26643600	2.19690800	-0.72555000
H	3.16540900	2.29988300	2.49026300	H	-2.41725100	3.08524900	-2.49363900
C	3.18952900	2.31741700	-1.40398400	C	-2.43331200	3.10616800	1.40622100
C	3.74081300	1.21551900	-0.72496700	C	-3.26638000	2.19697100	0.72560800
H	3.16540900	2.29988300	-2.49026300	H	-2.41703500	3.08548300	2.49354300
C	3.94235500	0.00000000	-1.40398900	C	-3.79449000	1.08275400	1.40636600
C	3.74081300	-1.21551900	-0.72496700	C	-3.93396500	-0.14246400	0.72573500
C	3.74081300	-1.21551900	0.72496700	C	-3.93402300	-0.14252700	-0.72540000
C	3.94235500	0.00000000	1.40398900	C	-3.79460600	1.08261100	-1.40615800
C	3.18952900	-2.31741700	1.40398400	C	-3.70634700	-1.35444000	-1.40603200
C	3.18952900	-2.31741700	-1.40398400	C	-3.70624000	-1.35429700	1.40646400
H	1.20907900	3.72128900	-2.49027000	H	-0.14176400	3.91682500	2.49341000
H	3.91253000	0.00000000	-2.49026800	H	-3.76917300	1.07558600	2.49368800
H	3.91253000	0.00000000	2.49026800	H	-3.76937600	1.07534300	-2.49348300
H	3.16540900	-2.29988300	2.49026300	H	-3.68170400	-1.34547000	-2.49335700
H	3.16540900	-2.29988300	-2.49026300	H	-3.68151000	-1.34522800	2.49378600
**[11]-cyclcacene**				**[11]-cyclcacene**			
C	4.31451900	0.27340600	0.72444700	C	-0.82699000	-4.24658000	0.72514200
C	4.31451900	0.27340600	-0.72444700	C	-0.82697400	-4.24659300	-0.72507500
C	4.07091400	1.48086900	1.40369500	C	0.40379000	-4.31647200	1.40598200
C	4.22523100	-0.95514700	1.40369000	C	-1.99406300	-3.84953000	1.40594900
C	4.07091400	1.48086900	-1.40369500	C	0.40382000	-4.31649600	-1.40588600
C	4.22523100	-0.95514700	-1.40369000	C	-1.99403300	-3.84955600	-1.40591600
C	3.48184400	2.56267700	-0.72444700	C	1.60029100	-4.01971200	-0.72505600
C	2.62411400	3.44676300	-1.40368400	C	2.67351100	-3.41309800	-1.40586100
C	1.54367300	4.03833900	-0.72444800	C	3.51946800	-2.51644600	-0.72505800
C	1.54367300	4.03833900	0.72444800	C	3.51945100	-2.51643100	0.72518900
C	0.34411400	4.31829100	1.40368600	C	4.09428300	-1.42587600	1.40599700
C	-0.88463800	4.23178200	0.72444600	C	4.32127800	-0.21420300	0.72518100
H	0.34197900	4.29148600	2.49002100	H	4.07181800	-1.41806000	2.49339200
C	2.62411400	3.44676300	1.40368400	C	2.67348000	-3.41307200	1.40599000
C	3.48184400	2.56267700	0.72444700	C	1.60027600	-4.01969800	0.72517300
H	2.60780600	3.42535400	2.49001800	H	2.65882200	-3.39438800	2.49338600
C	0.34411400	4.31829100	-1.40368600	C	4.09431700	-1.42590500	-1.40587600
H	2.60780600	3.42535400	-2.49001800	H	2.65887700	-3.39443500	-2.49325800
C	-0.88463800	4.23178200	-0.72444600	C	4.32129500	-0.21421800	-0.72507900
C	-2.04514500	3.81884700	1.40368400	C	4.21526300	1.01399400	1.40597400
H	0.34197900	4.29148600	-2.49002100	H	4.07187800	-1.41811200	-2.49327200
H	4.04566700	1.47168100	-2.49002900	H	0.40161600	-4.29285300	-2.49328100
H	4.04566700	1.47168100	2.49002900	H	0.40156100	-4.29281200	2.49337700
C	-2.04514500	3.81884700	-1.40368400	C	4.21529700	1.01396600	-1.40589900
C	-3.03209000	3.08177100	0.72444900	C	3.75110500	2.15603900	0.72514300
C	-3.78504600	2.10691000	1.40369400	C	2.99795800	3.13196000	1.40592400
C	-4.21677000	0.95325100	0.72444700	C	1.99000100	3.84172900	0.72509900
C	-4.32317100	-0.27393700	1.40369200	C	0.82873900	4.25540800	1.40590200
C	-4.06271400	-1.47787400	0.72444400	C	-0.40292400	4.30756100	0.72506000
C	-3.48881100	-2.56780500	1.40369000	C	-1.60354700	4.02789300	1.40587300
C	-2.61883800	-3.43984700	0.72445100	C	-2.66794900	3.40597300	0.72506400
C	-2.61883800	-3.43984700	-0.72445100	C	-2.66793300	3.40595800	-0.72519300
C	-1.54676200	-4.04645700	1.40368600	C	-3.52678300	2.52159400	1.40587700
C	-3.48881100	-2.56780500	-1.40369000	C	-1.60351700	4.02786100	-1.40599200
C	-1.54676200	-4.04645700	-1.40368600	C	-3.52675500	2.52156500	-1.40600600
H	-3.46715900	-2.55185600	2.49002500	H	-1.59477200	4.00588300	2.49327000

C	-4.06271400	-1.47787400	-0.72444400	C	-0.40290800	4.30754500	-0.72515900
H	-4.29638800	-0.27224300	2.49002700	H	0.82418200	4.23211300	2.49329700
C	-4.32317100	-0.27393700	-1.40369200	C	0.82876900	4.25537600	-1.40597200
C	-4.21677000	0.95325100	-0.72444700	C	1.99001700	3.84171300	-0.72513400
H	-4.29638800	-0.27224300	-2.49002700	H	0.82423600	4.23205600	-2.49336700
C	-3.78504600	2.10691000	-1.40369400	C	2.99798900	3.13193000	-1.40592100
H	-3.46715900	-2.55185600	-2.49002500	H	-1.59471600	4.00582600	-2.49338800
C	-3.03209000	3.08177100	-0.72444900	C	3.75112100	2.15602400	-0.72510300
H	-3.76156900	2.09384600	-2.49002900	H	2.98158300	3.11478500	-2.49331800
H	-3.76156900	2.09384600	2.49002900	H	2.98152700	3.11483800	2.49332100
H	-2.03246400	3.79517200	-2.49002000	H	4.19220400	1.00840900	-2.49329500
H	-2.03246400	3.79517200	2.49002000	H	4.19214300	1.00845900	2.49336900
C	-0.34342000	-4.30966200	-0.72444900	C	-4.08594900	1.42294700	-0.72517900
H	-1.53715700	-4.02132600	-2.49002000	H	-3.50738700	2.50772700	-2.49340100
C	0.88642400	-4.24037200	-1.40368900	C	-4.33034100	0.21464600	-1.40596800
C	-0.34342000	-4.30966200	0.72444900	C	-4.08596400	1.42296100	0.72506000
C	2.04103500	-3.81121200	-0.72445000	C	-4.20665800	-1.01188500	-0.72515600
H	0.88092500	-4.21403900	-2.49002300	H	-4.30661500	0.21346100	-2.49336400
C	0.88642400	-4.24037200	1.40368900	C	-4.33036600	0.21467500	1.40586900
C	2.04103500	-3.81121200	0.72445000	C	-4.20667300	-1.01187000	0.72508400
H	0.88092500	-4.21403900	2.49002300	H	-4.30666000	0.21351100	2.49326500
C	3.03815600	-3.08795900	1.40368600	C	-3.75889400	-2.16041500	1.40592200
C	3.03815600	-3.08795900	-1.40368600	C	-3.75886700	-2.16044300	-1.40596100
H	-1.53715700	-4.02132600	2.49002000	H	-3.50743900	2.50777600	2.49327200
C	3.77737600	-2.10264000	0.72444300	C	-2.99173200	-3.12540200	0.72512700
H	3.01934000	-3.06882500	2.49002100	H	-3.73827800	-2.14855600	2.49331700
C	3.77737600	-2.10264000	-0.72444300	C	-2.99171600	-3.12541700	-0.72513000
H	3.01934000	-3.06882500	-2.49002100	H	-3.73822900	-2.14860900	-2.49335600
H	4.19905000	-0.94922900	2.49002600	H	-1.98315500	-3.82848100	2.49334600
H	4.19905000	-0.94922900	-2.49002600	H	-1.98309900	-3.82852800	-2.49331300
**[12]-cyclcacene**				**[12]-cyclcacene**			
C	3.33099700	3.33099700	0.72505300	C	-3.99008400	-2.51110200	0.72563300
C	3.33099700	3.33099700	-0.72505300	C	-3.99008400	-2.51110200	-0.72563300
C	4.08654700	2.35937000	1.40387400	C	-4.51180500	-1.39519500	1.40614900
C	2.35937000	4.08654700	1.40387400	C	-3.20983400	-3.46432900	1.40614000
C	4.08654700	2.35937000	-1.40387400	C	-4.51180500	-1.39519500	-1.40614900
C	2.35937000	4.08654700	-1.40387400	C	-3.20983400	-3.46432900	-1.40614000
C	4.55023000	1.21922900	0.72505500	C	-4.71110700	-0.17957800	0.72563100
H	4.06509000	2.34698300	2.49030200	H	-4.49087500	-1.38872600	2.49359600
C	4.71873500	0.00000000	1.40387800	C	-4.60508300	1.04770500	1.40615600
C	4.55023000	1.21922900	-0.72505500	C	-4.71110700	-0.17957800	-0.72563100
C	4.55023000	-1.21922900	0.72505500	C	-4.16982100	2.20006700	0.72563400
H	4.69394800	0.00000000	2.49030600	H	-4.58374300	1.04284000	2.49360400
C	4.71873500	0.00000000	-1.40387800	C	-4.60508300	1.04770500	-1.40615600
C	4.55023000	-1.21922900	-0.72505500	C	-4.16982100	2.20006700	-0.72563400
H	4.69394800	0.00000000	-2.49030600	H	-4.58374300	1.04284000	-2.49360400
C	4.08654700	-2.35937000	-1.40387400	C	-3.46427200	3.20987900	-1.40614000
C	4.08654700	-2.35937000	1.40387400	C	-3.46427200	3.20987900	1.40614000
H	4.06509000	2.34698300	-2.49030200	H	-4.49087500	-1.38872600	-2.49359600
C	3.33099700	-3.33099700	-0.72505300	C	-2.51109800	3.99013200	-0.72563800
H	4.06509000	-2.34698300	-2.49030200	H	-3.44825700	3.19500500	-2.49358800
C	3.33099700	-3.33099700	0.72505300	C	-2.51109800	3.99013200	0.72563800
H	4.06509000	-2.34698300	2.49030200	H	-3.44825700	3.19500500	2.49358800
C	2.35937000	-4.08654700	1.40387400	C	-1.39519200	4.51193000	1.40615400
C	1.21922900	-4.55023000	0.72505500	C	-0.17957500	4.71119100	0.72564700
C	0.00000000	-4.71873500	1.40387800	C	1.04769900	4.60522100	1.40616200
C	-1.21922900	-4.55023000	0.72505500	C	2.20007500	4.16986900	0.72564400
H	0.00000000	-4.69394800	2.49030600	H	1.04286800	4.58393600	2.49361100

C	-2.35937000	-4.08654700	1.40387400	C	3.20982800	3.46433600	1.40614000
C	-1.21922900	-4.55023000	-0.72505500	C	2.20007500	4.16986900	-0.72564400
C	-3.33099700	-3.33099700	0.72505300	C	3.99009400	2.51109400	0.72563000
H	-2.34698300	-4.06509000	2.49030100	H	3.19499200	3.44829300	2.49358900
C	-2.35937000	-4.08654700	-1.40387400	C	3.20982800	3.46433600	-1.40614000
C	0.00000000	-4.71873500	-1.40387800	C	1.04769900	4.60522100	-1.40616200
C	-3.33099700	-3.33099700	-0.72505300	C	3.99009400	2.51109400	-0.72563000
H	-2.34698300	-4.06509000	-2.49030100	H	3.19499200	3.44829300	-2.49358900
C	-4.08654700	-2.35937000	-1.40387400	C	4.51179900	1.39520100	-1.40614900
C	-4.08654700	-2.35937000	1.40387400	C	4.51179900	1.39520100	1.40614900
C	1.21922900	-4.55023000	-0.72505500	C	-0.17957500	4.71119100	-0.72564700
H	0.00000000	-4.69394800	-2.49030600	H	1.04286800	4.58393600	-2.49361100
H	-4.06509000	-2.34698300	-2.49030200	H	4.49087200	1.38872000	-2.49359600
C	-4.55023000	-1.21922900	-0.72505500	C	4.71110800	0.17956900	-0.72563000
H	-4.06509000	-2.34698300	2.49030200	H	4.49087200	1.38872000	2.49359600
C	-4.55023000	-1.21922900	0.72505500	C	4.71110800	0.17956900	0.72563000
C	2.35937000	-4.08654700	-1.40387400	C	-1.39519200	4.51193000	-1.40615400
C	-4.71873500	0.00000000	1.40387800	C	4.60508600	-1.04769800	1.40615600
C	-4.71873500	0.00000000	-1.40387800	C	4.60508600	-1.04769800	-1.40615600
H	2.34698300	-4.06509000	-2.49030200	H	-1.38875400	4.49104800	-2.49360200
H	2.34698300	-4.06509000	2.49030200	H	-1.38875400	4.49104800	2.49360200
C	1.21922900	4.55023000	-0.72505500	C	-2.20006600	-4.16987700	-0.72564000
C	0.00000000	4.71873500	-1.40387800	C	-1.04770000	-4.60520800	-1.40616100
C	-1.21922900	4.55023000	-0.72505500	C	0.17957400	-4.71120200	-0.72563800
C	-1.21922900	4.55023000	0.72505500	C	0.17957400	-4.71120200	0.72563800
C	-2.35937000	4.08654700	1.40387400	C	1.39519400	-4.51191900	1.40615300
C	-3.33099700	3.33099700	0.72505300	C	2.51108900	-3.99014100	0.72563600
H	-2.34698300	4.06509000	2.49030100	H	1.38873700	-4.49102500	2.49360100
C	0.00000000	4.71873500	1.40387800	C	-1.04770000	-4.60520800	1.40616100
C	1.21922900	4.55023000	0.72505500	C	-2.20006600	-4.16987700	0.72564000
H	0.00000000	4.69394800	2.49030600	H	-1.04285400	-4.58390600	2.49360900
C	-2.35937000	4.08654700	-1.40387400	C	1.39519400	-4.51191900	-1.40615300
H	0.00000000	4.69394800	-2.49030600	H	-1.04285400	-4.58390600	-2.49360900
C	-3.33099700	3.33099700	-0.72505300	C	2.51108900	-3.99014100	-0.72563600
C	-4.08654700	2.35937000	1.40387400	C	3.46427900	-3.20987400	1.40613900
H	-2.34698300	4.06509000	-2.49030100	H	1.38873700	-4.49102500	-2.49360100
H	2.34698300	4.06509000	-2.49030200	H	-3.19497900	-3.44829700	-2.49358900
H	2.34698300	4.06509000	2.49030200	H	-3.19497900	-3.44829700	2.49358900
C	-4.08654700	2.35937000	-1.40387400	C	3.46427900	-3.20987400	-1.40613900
C	-4.55023000	1.21922900	-0.72505500	C	4.16981100	-2.20007500	-0.72563700
H	-4.69394900	0.00000000	-2.49030600	H	4.58374300	-1.04284300	-2.49360400
C	-4.55023000	1.21922900	0.72505500	C	4.16981100	-2.20007500	0.72563700
H	-4.69394900	0.00000000	2.49030600	H	4.58374300	-1.04284300	2.49360400
H	-4.06509000	2.34698300	-2.49030200	H	3.44824900	-3.19501300	-2.49358800
H	-4.06509000	2.34698300	2.49030200	H	3.44824900	-3.19501300	2.49358800
**[13]-cyclcacene**				**[13]-cyclcacene**			
C	2.67110100	4.34517500	0.72483300	C	-4.34383300	2.68057600	0.72544300
C	2.67110100	4.34517500	-0.72483300	C	-4.34383300	2.68057600	-0.72544300
C	1.55615000	4.86565700	1.40380900	C	-4.86641600	1.56541300	1.40590900
C	3.63852700	3.58480900	1.40380500	C	-3.58165400	3.64790900	1.40589600
C	1.55615000	4.86565700	-1.40380900	C	-4.86641600	1.56541300	-1.40590900
C	3.63852700	3.58480900	-1.40380500	C	-3.58165400	3.64790900	-1.40589600
C	0.34616700	5.08916400	0.72482300	C	-5.09183500	0.35469400	0.72545100
H	1.54927300	4.84396700	2.49024500	H	-4.84710500	1.55921100	2.49337500
C	4.38376800	2.60572700	0.72483500	C	-2.60072600	4.39250700	0.72544400
H	3.62239200	3.56892500	2.49024700	H	-3.56742000	3.63340900	2.49336200
C	0.34616700	5.08916400	-0.72482300	C	-5.09183500	0.35469400	-0.72545100
H	1.54927300	4.84396700	-2.49024500	H	-4.84710500	1.55921100	-2.49337500

C	4.38376800	2.60572700	-0.72483500	C	-2.60072600	4.39250700	-0.72544400
H	3.62239200	3.56892500	-2.49024700	H	-3.56742000	3.63340900	-2.49336200
C	-0.88295400	5.03165800	1.40380200	C	-5.03645500	-0.87559600	1.40592000
C	4.88712800	1.48294200	1.40380600	C	-1.47630200	4.89485100	1.40590200
C	-0.88295400	5.03165800	-1.40380200	C	-5.03645500	-0.87559600	-1.40592000
C	4.88712800	1.48294200	-1.40380600	C	-1.47630200	4.89485100	-1.40590200
C	-2.05813600	4.66701000	0.72485100	C	-4.67354100	-2.05243600	0.72545000
H	-0.87894700	5.00935900	2.49024100	H	-5.01647800	-0.87211600	2.49338600
C	5.09223000	0.26969500	0.72483100	C	-0.26167700	5.09832100	0.72545600
H	4.86551200	1.47639800	2.49024900	H	-1.47043700	4.87536600	2.49336700
C	-2.05813600	4.66701000	-0.72485100	C	-4.67354100	-2.05243600	-0.72545000
H	-0.87894700	5.00935900	-2.49024100	H	-5.01647800	-0.87211600	-2.49338600
C	5.09223000	0.26969500	-0.72483100	C	-0.26167700	5.09832100	-0.72545600
H	4.86551200	1.47639800	-2.49024900	H	-1.47043700	4.87536600	-2.49336700
C	-3.11963200	4.04471500	1.40380200	C	-4.05278200	-3.11607700	1.40591200
C	5.01634400	-0.95841200	1.40381700	C	0.96739100	5.02042000	1.40591800
C	-3.11963200	4.04471500	-1.40380200	C	-4.05278200	-3.11607700	-1.40591200
C	5.01634400	-0.95841200	-1.40381700	C	0.96739100	5.02042000	-1.40591800
C	-3.99063500	3.17559900	0.72482800	C	-3.18453400	-3.98946700	0.72544600
H	-3.10589800	4.02683300	2.49024600	H	-4.03670100	-3.10371800	2.49337800
C	-3.99063500	3.17559900	-0.72482800	C	-3.18453400	-3.98946700	-0.72544600
H	-3.10589800	4.02683300	-2.49024600	H	-4.03670100	-3.10371800	-2.49337800
C	-4.64136200	2.13130100	-1.40381200	C	-2.14059900	-4.64285100	-1.40590200
C	-4.64136200	2.13130100	1.40381200	C	-2.14059900	-4.64285100	1.40590200
C	-5.00883200	0.95699400	-0.72482400	C	-0.96588500	-5.01271000	-0.72545000
H	-4.62083600	2.12180900	-2.49025200	H	-2.13209100	-4.62440100	-2.49336700
C	-5.00883200	0.95699400	0.72482400	C	-0.96588500	-5.01271000	0.72545000
H	-4.62083600	2.12180900	2.49025200	H	-2.13209100	-4.62440100	2.49336700
C	-5.09986200	-0.27009300	1.40381000	C	0.26210400	-5.10603800	1.40590400
C	-4.87973300	-1.48068900	0.72483100	C	1.47410100	-4.88749200	0.72545200
H	-5.07726200	-0.26892800	2.49025000	H	0.26106200	-5.08573700	2.49337000
C	-5.09986200	-0.27009300	-1.40381000	C	0.26210400	-5.10603800	-1.40590400
C	-4.87973300	-1.48068900	-0.72483100	C	1.47410100	-4.88749200	-0.72545200
C	-4.39036900	-2.60964600	1.40380400	C	2.60475200	-4.39931100	1.40590800
H	-5.07726200	-0.26892800	-2.49025000	H	0.26106200	-5.08573700	-2.49337000
C	-4.39036900	-2.60964600	-1.40380400	C	2.60475200	-4.39931100	-1.40590800
H	-4.37092300	-2.59811100	2.49024400	H	2.59439300	-4.38182500	2.49337400
C	-3.63303600	-3.57942900	0.72483300	C	3.57626700	-3.64244700	0.72545200
H	-4.37092300	-2.59811100	-2.49024400	H	2.59439300	-4.38182500	-2.49337400
C	-3.63303600	-3.57942900	-0.72483300	C	3.57626700	-3.64244700	-0.72545200
C	-2.67511500	-4.35171200	1.40380700	C	4.35047200	-2.68469500	1.40591300
C	-2.67511500	-4.35171200	-1.40380700	C	4.35047200	-2.68469500	-1.40591300
C	-1.55381800	-4.85838000	0.72482200	C	4.85905700	-1.56307000	0.72545000
H	-2.66325500	-4.33237300	2.49024500	H	4.33318500	-2.67403000	2.49337800
C	-1.55381800	-4.85838000	-0.72482200	C	4.85905700	-1.56307000	-0.72545000
H	-2.66325500	-4.33237300	-2.49024500	H	4.33318500	-2.67403000	-2.49337800
C	-0.34668200	-5.09684500	-1.40380700	C	5.09956900	-0.35524700	-1.40590000
C	-0.34668200	-5.09684500	1.40380700	C	5.09956900	-0.35524700	1.40590000
C	0.88161600	-5.02413500	-0.72483600	C	5.02871600	0.87423700	-0.72544800
H	-0.34523200	-5.07420200	-2.49024500	H	5.07933200	-0.35384400	-2.49336600
C	0.88161600	-5.02413500	0.72483600	C	5.02871600	0.87423700	0.72544800
H	-0.34523200	-5.07420200	2.49024500	H	5.07933200	-0.35384400	2.49336600
C	2.06121900	-4.67402700	1.40379600	C	4.68051900	2.05553900	1.40590900
C	2.06121900	-4.67402700	-1.40379600	C	4.68051900	2.05553900	-1.40590900
C	4.63437600	-2.12807800	-0.72482000	C	2.13736900	4.63594400	-0.72545000
H	4.99412200	-0.95412500	-2.49025600	H	0.96353500	5.00042600	-2.49338400
C	4.63437600	-2.12807800	0.72482000	C	2.13736900	4.63594400	0.72545000
H	4.99412200	-0.95412500	2.49025600	H	0.96353500	5.00042600	2.49338400
C	3.99662300	-3.18036800	-1.40380300	C	3.18937400	3.99564900	-1.40591400
C	3.99662300	-3.18036800	1.40380300	C	3.18937400	3.99564900	1.40591400

C	3.11491400	-4.03861700	-0.72484600	C	4.04656100	3.11139300	-0.72545300
H	3.97901000	-3.16623900	-2.49024300	H	3.17669800	3.97978700	-2.49338000
C	3.11491400	-4.03861700	0.72484600	C	4.04656100	3.11139300	0.72545300
H	3.97901000	-3.16623900	2.49024300	H	3.17669800	3.97978700	2.49338000
H	2.05206200	-4.65338600	2.49023800	H	4.66195200	2.04738200	2.49337600
H	2.05206200	-4.65338600	-2.49023800	H	4.66195200	2.04738200	-2.49337600
**[14]-cyclcacene**				**[14]-cyclcacene**			
C	-0.00009500	5.48895300	0.72511500	C	-5.49450100	0.00000000	0.72563200
C	-0.00009500	5.48895300	-0.72511500	C	-5.49450100	0.00000000	-0.72563200
C	1.22296600	5.35834500	1.40373500	C	-5.36377800	1.22413500	1.40582000
C	-1.22315100	5.35830300	1.40373500	C	-5.36377800	-1.22413600	1.40582000
C	1.22296600	5.35834500	-1.40373500	C	-5.36377800	1.22413500	-1.40582000
C	-1.22315100	5.35830300	-1.40373500	C	-5.36377800	-1.22413600	-1.40582000
C	2.38161000	4.94544800	0.72511700	C	-4.95032600	2.38372800	0.72564000
H	1.21827500	5.33778800	2.49025300	H	-5.34538900	1.21993700	2.49336000
C	3.42691400	4.29715400	1.40373300	C	-4.30131700	3.42986600	1.40582100
C	2.38161000	4.94544800	-0.72511700	C	-4.95032600	2.38372800	-0.72564000
C	4.29169000	3.42245600	0.72511400	C	-3.42562800	4.29516400	0.72563900
H	3.41376800	4.28065900	2.49025200	H	-4.28655900	3.41809700	2.49336300
C	3.42691400	4.29715400	-1.40373300	C	-4.30131700	3.42986600	-1.40582100
C	4.29169000	3.42245600	-0.72511400	C	-3.42562800	4.29516400	-0.72563900
H	3.41376800	4.28065900	-2.49025200	H	-4.28655900	3.41809700	-2.49336300
C	4.95222800	2.38484700	-1.40373300	C	-2.38692600	4.95602900	-1.40581100
C	4.95222800	2.38484700	1.40373300	C	-2.38692600	4.95602900	1.40581100
H	1.21827500	5.33778800	-2.49025300	H	-5.34538900	1.21993700	-2.49336000
C	5.35184300	1.22155300	-0.72511700	C	-1.22251900	5.35570400	-0.72562500
H	4.93322500	2.37569600	-2.49025200	H	-2.37873000	4.93903000	-2.49335200
C	5.35184300	1.22155300	0.72511700	C	-1.22251900	5.35570400	0.72562500
H	4.93322500	2.37569600	2.49025200	H	-2.37873000	4.93903000	2.49335200
C	5.49669300	0.00009500	1.40373500	C	0.00000000	5.50064600	1.40580500
C	5.35188500	-1.22136900	0.72511700	C	1.22251900	5.35570400	0.72562500
C	4.95231000	-2.38467600	1.40373300	C	2.38692600	4.95602900	1.40581100
C	4.29180800	-3.42230800	0.72511400	C	3.42562800	4.29516400	0.72563900
H	4.93330700	-2.37552600	2.49025200	H	2.37872900	4.93903000	2.49335200
C	3.42706200	-4.29703600	1.40373300	C	4.30131700	3.42986600	1.40582100
C	4.29180800	-3.42230800	-0.72511400	C	3.42562800	4.29516400	-0.72563900
C	2.38178000	-4.94536600	0.72511700	C	4.95032600	2.38372900	0.72564000
H	3.41391600	-4.28054200	2.49025200	H	4.28655900	3.41809700	2.49336300
C	3.42706200	-4.29703600	-1.40373300	C	4.30131700	3.42986600	-1.40582100
C	4.95231000	-2.38467600	-1.40373300	C	2.38692600	4.95602900	-1.40581100
C	2.38178000	-4.94536600	-0.72511700	C	4.95032600	2.38372900	-0.72564000
H	3.41391600	-4.28054200	-2.49025200	H	4.28655900	3.41809700	-2.49336300
C	1.22315100	-5.35830300	-1.40373500	C	5.36377800	1.22413600	-1.40582000
C	1.22315100	-5.35830300	1.40373500	C	5.36377800	1.22413600	1.40582000
C	5.35188500	-1.22136900	-0.72511700	C	1.22251900	5.35570400	-0.72562500
H	4.93330700	-2.37552600	-2.49025200	H	2.37872900	4.93903000	-2.49335200
H	1.21845900	-5.33774600	-2.49025300	H	5.34538900	1.21993700	-2.49336000
C	0.00009500	-5.48895300	-0.72511500	C	5.49450100	0.00000000	-0.72563200
H	1.21845900	-5.33774600	2.49025300	H	5.34538900	1.21993700	2.49336000
C	0.00009500	-5.48895300	0.72511500	C	5.49450100	0.00000000	0.72563200
C	5.49669300	0.00009500	-1.40373500	C	0.00000000	5.50064600	-1.40580500
C	-1.22296600	-5.35834500	1.40373500	C	5.36377800	-1.22413500	1.40582000
C	-1.22296600	-5.35834500	-1.40373500	C	5.36377800	-1.22413500	-1.40582000
H	5.47561400	0.00009400	-2.49025500	H	0.00000000	5.48179900	-2.49334600
H	5.47561400	0.00009400	2.49025500	H	0.00000000	5.48179900	2.49334600
C	-2.38178000	4.94536600	-0.72511700	C	-4.95032600	-2.38372900	-0.72564000
C	-3.42706200	4.29703600	-1.40373300	C	-4.30131700	-3.42986600	-1.40582100
C	-4.29180800	3.42230800	-0.72511400	C	-3.42562800	-4.29516400	-0.72563900

C	-4.29180800	3.42230800	0.72511400	C	-3.42562800	-4.29516400	0.72563900
C	-4.95231000	2.38467600	1.40373300	C	-2.38692600	-4.95602900	1.40581100
C	-5.35188500	1.22136900	0.72511700	C	-1.22251900	-5.35570400	0.72562500
H	-4.93330700	2.37552600	2.49025200	H	-2.37872900	-4.93903000	2.49335200
C	-3.42706200	4.29703600	1.40373300	C	-4.30131700	-3.42986600	1.40582100
C	-2.38178000	4.94536600	0.72511700	C	-4.95032600	-2.38372900	0.72564000
H	-3.41391600	4.28054200	2.49025200	H	-4.28655900	-3.41809700	2.49336300
C	-4.95231000	2.38467600	-1.40373300	C	-2.38692600	-4.95602900	-1.40581100
H	-3.41391600	4.28054200	-2.49025200	H	-4.28655900	-3.41809700	-2.49336300
C	-5.35188500	1.22136900	-0.72511700	C	-1.22251900	-5.35570400	-0.72562500
C	-5.49669300	-0.00009500	1.40373500	C	0.00000000	-5.50064600	1.40580500
H	-4.93330700	2.37552600	-2.49025200	H	-2.37872900	-4.93903000	-2.49335200
H	-1.21845900	5.33774600	-2.49025300	H	-5.34538900	-1.21993700	-2.49336000
H	-1.21845900	5.33774600	2.49025300	H	-5.34538900	-1.21993700	2.49336000
C	-5.49669300	-0.00009500	-1.40373500	C	0.00000000	-5.50064600	-1.40580500
C	-2.38161000	-4.94544800	0.72511700	C	4.95032600	-2.38372800	0.72564000
H	-1.21827500	-5.33778800	2.49025300	H	5.34538900	-1.21993700	2.49336000
C	-3.42691400	-4.29715400	1.40373300	C	4.30131700	-3.42986600	1.40582100
C	-2.38161000	-4.94544800	-0.72511700	C	4.95032600	-2.38372800	-0.72564000
C	-4.29169000	-3.42245600	0.72511400	C	3.42562800	-4.29516400	0.72563900
H	-3.41376800	-4.28065900	2.49025200	H	4.28655900	-3.41809700	2.49336300
C	-3.42691400	-4.29715400	-1.40373300	C	4.30131700	-3.42986600	-1.40582100
C	-4.29169000	-3.42245600	-0.72511400	C	3.42562800	-4.29516400	-0.72563900
H	-3.41376800	-4.28065900	-2.49025200	H	4.28655900	-3.41809700	-2.49336300
C	-4.95222800	-2.38484700	-1.40373300	C	2.38692600	-4.95602900	-1.40581100
C	-4.95222800	-2.38484700	1.40373300	C	2.38692600	-4.95602900	1.40581100
H	-1.21827500	-5.33778800	-2.49025300	H	5.34538900	-1.21993700	-2.49336000
C	-5.35184300	-1.22155300	-0.72511700	C	1.22251900	-5.35570400	-0.72562500
H	-4.93322500	-2.37569600	-2.49025200	H	2.37873000	-4.93903000	-2.49335200
C	-5.35184300	-1.22155300	0.72511700	C	1.22251900	-5.35570400	0.72562500
H	-4.93322500	-2.37569600	2.49025200	H	2.37873000	-4.93903000	2.49335200
H	-5.47561400	-0.00009400	-2.49025500	H	0.00000000	-5.48179900	-2.49334600
H	-5.47561400	-0.00009400	2.49025500	H	0.00000000	-5.48179900	2.49334600
**[15]-cyclcacene**				**[15]-cyclcacene**			
C	0.19834500	5.87444500	0.72511400	C	5.09433300	2.94489400	0.72570500
C	0.19834100	5.87445900	-0.72500600	C	5.09434300	2.94490500	-0.72559700
C	-1.02872200	5.79407500	1.40384200	C	4.37562700	3.94407100	1.40580400
C	1.41720500	5.71151500	1.40383500	C	5.60195200	1.82362600	1.40579300
C	-1.02872900	5.79410100	-1.40372900	C	4.37564600	3.94409000	-1.40569100
C	1.41719800	5.71154100	-1.40373700	C	5.60197000	1.82364500	-1.40569400
C	-2.20852100	5.44733600	0.72511700	C	3.45584000	4.76194300	0.72572000
H	-1.02530900	5.77485200	2.49036200	H	4.36259700	3.93231100	2.49337000
C	2.57093700	5.28602100	0.72509900	C	5.85201200	0.61849000	0.72569800
H	1.41251700	5.69257800	2.49035400	H	5.58524800	1.81818100	2.49335700
C	-2.20852500	5.44735000	-0.72500400	C	3.45584900	4.76195400	-0.72560600
H	-1.02532300	5.77489800	-2.49024900	H	4.36262900	3.93234600	-2.49325700
C	2.57093300	5.28603500	-0.72501500	C	5.85202100	0.61850100	-0.72561300
H	1.41250300	5.69262400	-2.49025600	H	5.58528100	1.81821600	-2.49325900
C	-3.29688800	4.87495700	1.40383400	C	2.39289100	5.38246800	1.40579400
C	3.61826000	4.64160800	1.40382000	C	5.85946500	-0.61230300	1.40578300
C	-3.29689500	4.87498400	-1.40372600	C	2.39291000	5.38248700	-1.40568500
C	3.61825300	4.64163400	-1.40375400	C	5.85948400	-0.61228400	-1.40571600
C	-4.23374200	4.07843000	0.72511000	C	1.21998800	5.75551200	0.72570700
H	-3.28592900	4.85874400	2.49035200	H	2.38574600	5.36640800	2.49335900
C	4.49928400	3.78373000	0.72508700	C	5.59749200	-1.81490300	0.72568400
H	3.60626500	4.62620900	2.49034000	H	5.84202900	-0.61048700	2.49334900
C	-4.23374600	4.07844400	-0.72501100	C	1.21999700	5.75552200	-0.72560800
H	-3.28594300	4.85879000	-2.49024500	H	2.38577800	5.36644300	-2.49325100

C	4.49928000	3.78374400	-0.72504100	C	5.59750100	-1.81489200	-0.72563900
H	3.60625200	4.62625500	-2.49027400	H	5.84206200	-0.61045100	-2.49328300
C	-4.99527500	3.11293000	-1.40373900	C	-0.00344200	5.89007900	-1.40569600
C	-4.99526800	3.11290400	1.40382400	C	-0.00346200	5.89006000	1.40578000
C	-5.52713100	2.00418500	-0.72503100	C	-1.22670100	5.75394100	-0.72561400
H	-4.97866100	3.10258200	-2.49025800	H	-0.00343300	5.87253100	-2.49326100
C	-5.52712700	2.00417200	0.72509700	C	-1.22671000	5.75393100	0.72568000
H	-4.97864700	3.10253600	2.49034300	H	-0.00346600	5.87249600	2.49334600
C	-5.82994500	0.81235600	-1.40375600	C	-2.39915200	5.37950500	-1.40571200
C	-5.82993700	0.81233000	1.40380300	C	-2.39917100	5.37948400	1.40575800
C	-5.86462300	-0.41687600	0.72507400	C	-3.46139600	4.75777500	0.72566000
H	-5.81052100	0.80961100	2.49032100	H	-2.39203400	5.36345800	2.49332400
C	-5.86462700	-0.41686300	-0.72505000	C	-3.46138800	4.75778500	-0.72563700
H	-5.81053400	0.80965700	-2.49027500	H	-2.39200200	5.36349400	-2.49327800
C	-5.65628500	-1.62880700	1.40378100	C	-4.38031300	3.93891100	1.40573100
C	-5.65629200	-1.62878100	-1.40378100	C	-4.38029500	3.93893300	-1.40573100
C	-5.18784200	-2.76578300	0.72504900	C	-5.09789200	2.93890000	0.72564600
H	-5.63747100	-1.62339800	2.49030100	H	-4.36725300	3.92715200	2.49329500
C	-5.18784600	-2.76577000	-0.72507300	C	-5.09788300	2.93891000	-0.72567000
H	-5.63748400	-1.62335200	-2.49030100	H	-4.36722100	3.92718900	-2.49329500
C	-4.50447200	-3.78812800	1.40376100	C	-5.60420500	1.81705100	1.40571300
C	-3.61402000	-4.63620700	0.72502500	C	-5.85282900	0.61162100	0.72562400
H	-4.48953300	-3.77557300	2.49028100	H	-5.58750900	1.81162500	2.49327900
C	-4.50448000	-3.78810100	-1.40380800	C	-5.60418700	1.81707300	-1.40576000
C	-2.57392300	-5.29222800	1.40374400	C	-5.85882800	-0.61920500	1.40569200
C	-3.61402400	-4.63619400	-0.72509200	C	-5.85282000	0.61163200	-0.72569100
H	-4.48954600	-3.77552600	-2.49032700	H	-5.58747800	1.81166100	-2.49332600
C	-1.41554600	-5.70490900	0.72501100	C	-5.59542600	-1.82151400	0.72559800
H	-2.56539000	-5.27467900	2.49026300	H	-5.84137200	-0.61737100	2.49325700
C	-2.57393100	-5.29220100	-1.40382800	C	-5.85881000	-0.61918400	-1.40577700
C	-0.19856800	-5.88132600	1.40373300	C	-5.10019200	-2.94828900	1.40568600
C	-1.41554900	-5.70489600	-0.72511000	C	-5.59541700	-1.82150500	-0.72569700
H	-2.56540300	-5.27463200	-2.49034700	H	-5.84134100	-0.61733500	-2.49334200
C	1.02753000	-5.78736300	0.72500400	C	-4.37050300	-3.93945600	0.72559300
H	-0.19790800	-5.86183500	2.49025200	H	-5.08501500	-2.93951700	2.49325100
C	-0.19857500	-5.88129900	-1.40384100	C	-5.10017400	-2.94826800	-1.40579400
C	2.21109800	-5.45370900	1.40372500	C	-3.45979700	-4.76741300	1.40568400
C	1.02752600	-5.78734900	-0.72511600	C	-4.37049300	-3.93944600	-0.72570600
H	-0.19792100	-5.86178800	-2.49036000	H	-5.08498300	-2.93948100	-2.49335900
C	3.29305800	-4.86931700	0.72500400	C	-2.39008600	-5.37619900	0.72560600
H	2.20376100	-5.43560400	2.49024300	H	-3.44952200	-4.75322700	2.49325200
C	2.21109100	-5.45368300	-1.40383800	C	-3.45977900	-4.76739200	-1.40579700
C	4.23867900	-4.08322100	1.40372800	C	-1.22137800	-5.76219500	1.40569700
C	3.29305500	-4.86930300	-0.72511200	C	-2.39007600	-5.37618900	-0.72571300
H	2.20374800	-5.43555800	-2.49035600	H	-3.44948900	-4.75319100	-2.49336400
C	4.98947300	-3.10931700	0.72502200	C	0.00346600	-5.88331000	0.72562100
H	4.22456900	-4.06963600	2.49024700	H	-1.21774600	-5.74500300	2.49326200
C	4.23867200	-4.08319500	-1.40382700	C	-1.22136100	-5.76217400	-1.40579500
C	4.98946900	-3.10930300	-0.72510600	C	0.00347500	-5.88330000	-0.72570400
C	5.53352400	-2.00651500	1.40374400	C	1.22815500	-5.76071400	1.40571600
H	4.22455600	-4.06959000	-2.49034400	H	-1.21771400	-5.74496600	-2.49336000
C	5.53351700	-2.00648900	-1.40381000	C	1.22817200	-5.76069400	-1.40578200
C	5.82311600	-0.81139500	0.72503700	C	2.39642400	-5.37336800	0.72563000
H	5.51510800	-1.99983600	2.49026300	H	1.22449200	-5.74355100	2.49328100
C	5.82311300	-0.81138200	-0.72508300	C	2.39643400	-5.37335800	-0.72567700
H	5.51509500	-1.99979000	-2.49032900	H	1.22452400	-5.74351500	-2.49334700
C	5.87142300	0.41735100	1.40376600	C	3.46546100	-4.76340200	1.40572700
C	5.87141600	0.41737700	-1.40379000	C	3.46547800	-4.76338200	-1.40575100
C	5.64971700	1.62690900	0.72506200	C	4.37522200	-3.93439600	0.72565900
H	5.85188200	0.41594900	2.49028500	H	3.45512200	-4.74920000	2.49329300

C	5.64971300	1.62692300	-0.72506200
H	5.85186900	0.41599500	-2.49030800
C	5.19392300	2.76901000	1.40379900
H	5.17667300	2.75979800	2.49031800
C	5.19391500	2.76903600	-1.40377500
H	5.17666000	2.75984400	-2.49029400

[16]-cyclcacene

C	4.43232600	4.43232500	0.72519700
C	4.43232600	4.43232500	-0.72519700
C	3.48604500	5.21726300	1.40363400
C	5.21726400	3.48604400	1.40363400
C	3.48604500	5.21726300	-1.40363400
C	5.21726400	3.48604400	-1.40363400
C	2.39877100	5.79121000	-0.72519700
C	1.22416000	6.15433800	-1.40363200
C	0.00000000	6.26850600	-0.72520200
C	0.00000000	6.26850600	0.72520200
C	-1.22415900	6.15433800	1.40363200
C	-2.39877100	5.79121000	0.72519700
H	-1.22058100	6.13632000	2.49021300
C	1.22416000	6.15433800	1.40363200
C	2.39877100	5.79121000	0.72519700
H	1.22058100	6.13632000	2.49021300
C	-1.22415900	6.15433800	-1.40363200
H	1.22058100	6.13632000	-2.49021300
C	-2.39877100	5.79121000	-0.72519700
C	-3.48604400	5.21726400	1.40363400
H	-1.22058100	6.13632000	-2.49021300
H	3.47584300	5.20199800	-2.49021300
H	3.47584300	5.20199800	2.49021300
C	-3.48604400	5.21726400	-1.40363400
C	-4.43232500	4.43232600	0.72519700
C	-5.21726300	3.48604500	1.40363400
C	-5.79121000	2.39877200	0.72519700
C	-6.15433800	1.22416000	1.40363200
H	-6.13632000	1.22058200	2.49021300
C	-6.26850600	0.00000000	0.72520200
C	-5.79121000	2.39877200	-0.72519700
C	-6.15433800	1.22416000	-1.40363200
H	-6.13632000	1.22058200	-2.49021300
C	-6.26850600	0.00000000	-0.72520200
C	-5.21726300	3.48604500	-1.40363400
C	-4.43232500	4.43232600	-0.72519700
H	-5.20199900	3.47584300	-2.49021300
H	-5.20199900	3.47584300	2.49021300
H	-3.47584300	5.20199900	-2.49021300
H	-3.47584300	5.20199900	2.49021300
C	5.79121000	2.39877100	-0.72519700
C	6.15433800	1.22415900	-1.40363200
C	6.26850600	0.00000000	-0.72520200
C	6.26850600	0.00000000	0.72520200
C	6.15433800	1.22415900	1.40363200
H	6.13632000	1.22058100	2.49021300
C	5.79121000	2.39877100	0.72519700
C	6.15433800	-1.22416000	1.40363200
C	5.79121000	-2.39877200	0.72519700
H	6.13632000	-1.22058200	2.49021300
C	6.15433800	-1.22416000	-1.40363200

C	4.37523100	-3.93438600	-0.72566000
H	3.45515300	-4.74916400	-2.49331600
C	5.10365100	-2.94229900	1.40576700
H	5.08845900	-2.93354700	2.49333400
C	5.10366900	-2.94228000	-1.40574400
H	5.08849200	-2.93351200	-2.49331100

[16]-cyclcacene

C	0.00000000	6.27400600	0.72571200
C	0.00000000	6.27400600	-0.72571200
C	-1.22525600	6.15979200	1.40572300
C	1.22525600	6.15979200	1.40572300
C	-1.22525600	6.15979200	-1.40572300
C	1.22525600	6.15979200	-1.40572300
C	-2.40095000	5.79643400	-0.72572100
C	-3.48919500	5.22196600	-1.40573600
C	-4.43632000	4.43632000	-0.72572400
C	-4.43632000	4.43632000	0.72572400
C	-5.22196700	3.48919500	1.40573600
C	-5.79643500	2.40095000	0.72572100
H	-5.20830800	3.48006800	2.49332900
C	-3.48919500	5.22196600	1.40573600
C	-2.40095000	5.79643400	0.72572100
H	-3.48006800	5.20830800	2.49332900
C	-5.22196700	3.48919500	-1.40573600
H	-3.48006800	5.20830800	-2.49332900
C	-5.79643500	2.40095000	-0.72572100
C	-6.15979300	1.22525600	1.40572300
H	-5.20830800	3.48006800	-2.49332900
H	-1.22204800	6.14367200	-2.49331600
H	-1.22204800	6.14367200	2.49331600
C	-6.15979300	1.22525600	-1.40572300
C	-6.27400600	0.00000000	0.72571200
C	-6.15979300	-1.22525600	1.40572300
C	-5.79643500	-2.40095000	0.72572100
C	-5.22196700	-3.48919500	1.40573600
H	-5.20830800	-3.48006800	2.49332900
C	-4.43632000	-4.43632000	0.72572400
C	-5.79643500	-2.40095000	-0.72572100
C	-5.22196700	-3.48919500	-1.40573600
H	-5.20830800	-3.48006800	-2.49332900
C	-4.43632000	-4.43632000	-0.72572400
C	-6.15979300	-1.22525600	-1.40572300
C	-6.27400600	0.00000000	-0.72571200
H	-6.14367300	-1.22204800	-2.49331600
H	-6.14367300	-1.22204800	2.49331600
H	-6.14367300	1.22204800	-2.49331600
H	-6.14367300	1.22204800	2.49331600
C	2.40095000	5.79643400	-0.72572100
C	3.48919500	5.22196600	-1.40573600
C	4.43632000	4.43632000	-0.72572400
C	4.43632000	4.43632000	0.72572400
C	3.48919500	5.22196600	1.40573600
H	3.48006800	5.20830800	2.49332900
C	2.40095000	5.79643400	0.72572100
C	5.22196700	3.48919500	1.40573600
C	5.79643500	2.40095000	0.72572100
H	5.20830800	3.48006800	2.49332900
C	5.22196700	3.48919500	-1.40573600

C	5.79121000	-2.39877200	-0.72519700	C	5.79643500	2.40095000	-0.72572100
H	6.13632000	-1.22058200	-2.49021300	H	5.20830800	3.48006800	-2.49332900
C	5.21726300	-3.48604500	-1.40363400	C	6.15979300	1.22525600	-1.40572300
C	5.21726300	-3.48604500	1.40363400	C	6.15979300	1.22525600	1.40572300
H	6.13632000	1.22058100	-2.49021300	H	3.48006800	5.20830800	-2.49332900
C	4.43232500	-4.43232600	-0.72519700	C	6.27400600	0.00000000	-0.72571200
H	5.20199900	-3.47584300	-2.49021300	H	6.14367300	1.22204800	-2.49331600
C	4.43232500	-4.43232600	0.72519700	C	6.27400600	0.00000000	0.72571200
H	5.20199900	-3.47584300	2.49021300	H	6.14367300	1.22204800	2.49331600
C	3.48604400	-5.21726400	1.40363400	C	6.15979300	-1.22525600	1.40572300
C	2.39877100	-5.79121000	0.72519700	C	5.79643500	-2.40095000	0.72572100
C	1.22415900	-6.15433800	1.40363200	C	5.22196700	-3.48919500	1.40573600
C	0.00000000	-6.26850600	0.72520200	C	4.43632000	-4.43632000	0.72572400
H	1.22058100	-6.13632000	2.49021300	H	5.20830800	-3.48006800	2.49332900
C	-1.22416000	-6.15433800	1.40363200	C	3.48919500	-5.22196600	1.40573600
C	0.00000000	-6.26850600	-0.72520200	C	4.43632000	-4.43632000	-0.72572400
C	-2.39877100	-5.79121000	0.72519700	C	2.40095000	-5.79643400	0.72572100
H	-1.22058100	-6.13632000	2.49021300	H	3.48006800	-5.20830800	2.49332900
C	-1.22416000	-6.15433800	-1.40363200	C	3.48919500	-5.22196600	-1.40573600
C	1.22415900	-6.15433800	-1.40363200	C	5.22196700	-3.48919500	-1.40573600
C	-2.39877100	-5.79121000	-0.72519700	C	2.40095000	-5.79643400	-0.72572100
H	-1.22058100	-6.13632000	-2.49021300	H	3.48006800	-5.20830800	-2.49332900
C	-3.48604500	-5.21726300	-1.40363400	C	1.22525600	-6.15979200	-1.40572300
C	-3.48604500	-5.21726300	1.40363400	C	1.22525600	-6.15979200	1.40572300
C	2.39877100	-5.79121000	-0.72519700	C	5.79643500	-2.40095000	-0.72572100
H	1.22058100	-6.13632000	-2.49021300	H	5.20830800	-3.48006800	-2.49332900
H	-3.47584300	-5.20199800	-2.49021300	H	1.22204800	-6.14367200	-2.49331600
C	-4.43232600	-4.43232500	-0.72519700	C	0.00000000	-6.27400600	-0.72571200
H	-3.47584300	-5.20199800	2.49021300	H	1.22204800	-6.14367200	2.49331600
C	-4.43232600	-4.43232500	0.72519700	C	0.00000000	-6.27400600	0.72571200
C	3.48604400	-5.21726400	-1.40363400	C	6.15979300	-1.22525600	-1.40572300
C	-5.21726400	-3.48604400	1.40363400	C	-1.22525600	-6.15979200	1.40572300
C	-5.21726400	-3.48604400	-1.40363400	C	-1.22525600	-6.15979200	-1.40572300
H	3.47584300	-5.20199900	-2.49021300	H	6.14367300	-1.22204800	-2.49331600
H	3.47584300	-5.20199900	2.49021300	H	6.14367300	-1.22204800	2.49331600
H	-5.20199900	-3.47584200	2.49021300	H	-1.22204800	-6.14367200	2.49331600
C	-5.79121000	-2.39877100	0.72519700	C	-2.40095000	-5.79643400	0.72572100
H	-5.20199900	-3.47584200	-2.49021300	H	-1.22204800	-6.14367200	-2.49331600
C	-5.79121000	-2.39877100	-0.72519700	C	-2.40095000	-5.79643400	-0.72572100
H	5.20199900	3.47584200	-2.49021300	H	1.22204800	6.14367200	-2.49331600
H	5.20199900	3.47584200	2.49021300	H	1.22204800	6.14367200	2.49331600
C	-6.15433800	-1.22415900	-1.40363200	C	-3.48919500	-5.22196600	-1.40573600
H	-6.13632000	-1.22058100	-2.49021300	H	-3.48006800	-5.20830800	-2.49332900
C	-6.15433800	-1.22415900	1.40363200	C	-3.48919500	-5.22196600	1.40573600
H	-6.13632000	-1.22058100	2.49021300	H	-3.48006800	-5.20830800	2.49332900
**[17]-cyclcacene**				**[17]-cyclcacene**			
C	-2.95466400	5.97252200	1.40360000	C	-3.23860200	-5.83120000	1.40584300
C	-1.80504200	6.40771200	0.72524500	C	-4.25103400	-5.13209600	0.72572400
C	-3.99838800	5.32317600	0.72524300	C	-2.11001100	-6.32115500	0.72572400
H	-2.94703200	5.95708200	2.49020300	H	-3.23112400	-5.81773700	2.49339600
C	-0.59726700	6.63640400	1.40360000	C	-5.12643700	-4.26756000	1.40584100
C	-1.80504300	6.40771100	-0.72525500	C	-4.25103300	-5.13209600	-0.72573500
C	0.63193800	6.62709700	0.72524600	C	-5.81798600	-3.24994900	0.72572800
H	-0.59572500	6.61925800	2.49020400	H	-5.11459500	-4.25770400	2.49339400
C	-0.59726500	6.63640200	-1.40361100	C	-5.12643500	-4.26755900	-1.40585300
C	-2.95466200	5.97252100	-1.40361000	C	-3.23860000	-5.83119900	-1.40585300
C	0.63193700	6.62709600	-0.72525800	C	-5.81798400	-3.24994900	-0.72573900
H	-0.59572400	6.61925400	-2.49021600	H	-5.11459200	-4.25770200	-2.49340600

C	1.84079200	6.40417200	-1.40361400	C	-6.32193700	-2.12752600	-1.40584800
C	1.84079000	6.40417500	1.40360300	C	-6.32193900	-2.12752700	1.40583600
C	-3.99838900	5.32317500	-0.72525100	C	-2.11001000	-6.32115400	-0.72573300
C	-4.91334600	4.50229400	1.40360600	C	-0.91340000	-6.60731400	1.40584100
H	-2.94703100	5.95707800	-2.49021300	H	-3.23112100	-5.81773500	-2.49340600
H	1.83604000	6.38763000	-2.49021800	H	-6.30732800	-2.12260400	-2.49340000
C	2.98366300	5.95156000	-0.72525600	C	-6.59913800	-0.92880100	-0.72572500
H	1.83603900	6.38763400	2.49020700	H	-6.30733200	-2.12260600	2.49338900
C	2.98366400	5.95156100	0.72524600	C	-6.59913900	-0.92880100	0.72571400
C	-4.91334500	4.50229400	-1.40361300	C	-0.91339800	-6.60731200	-1.40584900
C	4.03041400	5.30710500	1.40360000	C	-6.66360600	0.29985600	1.40583900
C	4.03041600	5.30710000	-1.40361000	C	-6.66360400	0.29985700	-1.40584900
C	-5.65213900	3.51983100	-0.72525700	C	0.31597300	-6.65649100	-0.72572200
H	-4.90065300	4.49066200	-2.49021700	H	-0.91128600	-6.59205200	-2.49340100
C	-5.65213800	3.51983300	0.72525200	C	0.31597200	-6.65649200	0.72571600
C	-6.20867100	2.42379400	-1.40360900	C	1.53515700	-6.49108400	-1.40584700
C	-6.20867200	2.42379400	1.40360500	C	1.53515500	-6.49108500	1.40584300
C	-6.54244400	1.24074000	0.72525100	C	2.69928200	-6.09287000	0.72572800
H	-6.19260900	2.41751800	2.49020900	H	1.53160600	-6.47609500	2.49339500
C	-6.66525900	0.01765300	1.40360800	C	3.77638700	-5.49821100	1.40584800
C	-6.54244400	1.24073800	-0.72525300	C	2.69928300	-6.09286900	-0.72573000
C	-6.54893200	-1.20606400	0.72525300	C	4.71804900	-4.70635500	0.72573200
H	-6.64802000	0.01760900	2.49021300	H	3.76766400	-5.48551600	2.49340100
C	-6.22143800	-2.39087500	1.40361000	C	5.50763000	-3.76279000	1.40585000
C	-6.54893200	-1.20606600	-0.72525100	C	4.71805000	-4.70635400	-0.72572900
H	-6.20535500	-2.38468600	2.49021400	H	5.49491100	-3.75410300	2.49340300
C	-5.67071900	-3.48984400	0.72525700	C	6.09967500	-2.68424700	0.72573600
C	-6.66525900	0.01765300	-1.40360800	C	3.77638900	-5.49821000	-1.40584800
C	-6.22143900	-2.39087500	-1.40360600	C	5.50763200	-3.76278900	-1.40584600
H	-6.64802000	0.01760600	-2.49021200	H	3.76766800	-5.48551400	-2.49340100
H	-6.20535600	-2.38468900	-2.49021000	H	5.49491400	-3.75410100	-2.49339900
C	-5.67071900	-3.48984600	-0.72525100	C	6.09967600	-2.68424700	-0.72573000
H	-6.19260900	2.41751600	-2.49021300	H	1.53161000	-6.47609200	-2.49339900
H	-4.90065300	4.49066500	2.49021000	H	-0.91128900	-6.59205500	2.49339300
H	4.01997900	5.29335500	2.49020300	H	-6.64821000	0.29916100	2.49339200
C	4.93267100	4.47225900	0.72524800	C	-6.48907800	1.51775800	0.72572000
H	4.01998000	5.29335000	-2.49021300	H	-6.64820600	0.29916300	-2.49340100
C	4.93267100	4.47225800	-0.72525700	C	-6.48907700	1.51775800	-0.72572800
C	-4.93714300	-4.47620900	-1.40360500	C	6.49504900	-1.51913800	-1.40583900
H	-4.92438800	-4.46464500	-2.49020900	H	6.48004200	-1.51562200	-2.49339100
C	-4.02654600	-5.30192600	-0.72524300	C	6.65747200	-0.29954800	-0.72571500
C	-4.93714100	-4.47620800	1.40361300	C	6.49504700	-1.51913900	1.40584700
H	-4.92438700	-4.46464200	2.49021600	H	6.48003800	-1.51562400	2.49339900
C	-4.02654700	-5.30192400	0.72525200	C	6.65747100	-0.29954900	0.72572400
C	5.67590200	3.49315100	-1.40360700	C	-6.10536500	2.68675500	-1.40584500
C	6.21552200	2.38868900	-0.72525200	C	-5.50265400	3.75938600	-0.72573200
H	5.66123500	3.48411100	-2.49021100	H	-6.09125400	2.68054200	-2.49339800
C	6.55473700	1.20718000	-1.40360600	C	-4.72252100	4.71078100	-1.40584200
C	6.21552300	2.38869000	0.72524700	C	-5.50265500	3.75938600	0.72572700
C	6.65883700	-0.01763900	-0.72525000	C	-3.77299100	5.49318600	-0.72573100
H	6.53776400	1.20405200	-2.49020900	H	-4.71160800	4.69989400	-2.49339500
C	6.65883700	-0.01763900	0.72524900	C	-3.77299200	5.49318500	0.72573100
C	6.54825000	-1.24189400	-1.40360300	C	-2.70179900	6.09846700	-1.40583900
C	6.55473600	1.20718500	1.40360300	C	-4.72252300	4.71078000	1.40583900
C	6.54825000	-1.24188800	1.40360500	C	-2.70180100	6.09846600	1.40584100
H	6.53776400	1.20405900	2.49020600	H	-4.71161200	4.69989200	2.49339300
C	6.20278100	-2.42158600	-0.72524800	C	-1.53373700	6.48500900	-0.72571800
H	6.53129200	-1.23867700	-2.49020600	H	-2.69555000	6.08437300	-2.49339100
C	6.20278100	-2.42158500	0.72525200	C	-1.53373800	6.48500800	0.72572200
H	6.53129300	-1.23867100	2.49020800	H	-2.69555400	6.08437100	2.49339300

C	5.65731300	-3.52317000	1.40360700	C	-0.31626900	6.66252000	1.40584600
C	5.65731200	-3.52317500	-1.40360100	C	-0.31626600	6.66252100	-1.40584000
C	4.90890100	-4.49832300	-0.72524800	C	0.91254100	6.60110500	-0.72571600
H	5.64270000	-3.51406400	-2.49020500	H	-0.31553500	6.64713000	-2.49339200
C	4.90890100	-4.49832200	0.72525600	C	0.91254000	6.60110400	0.72572400
C	4.00223500	-5.32837800	-1.40360100	C	2.11196300	6.32692000	-1.40583500
C	4.00223700	-5.32837400	1.40361000	C	2.11196000	6.32691900	1.40584500
C	2.95208500	-5.96727900	0.72525700	C	3.23566600	5.82581600	0.72573700
H	3.99188000	-5.31457200	2.49021400	H	2.10707400	6.31229700	2.49339700
C	1.80683000	-6.41382600	1.40361300	C	4.25503700	5.13687000	1.40584800
C	2.95208600	-5.96728000	-0.72524600	C	3.23566700	5.82581700	-0.72572700
C	0.59681200	-6.63034300	0.72525800	C	5.12179300	4.26366200	0.72573500
H	1.80216300	-6.39724700	2.49021700	H	4.24520700	5.12499900	2.49340100
C	1.80682700	-6.41382900	-1.40360200	C	4.25503900	5.13687100	-1.40583700
C	0.59681200	-6.63034400	-0.72524600	C	5.12179400	4.26366200	-0.72572300
H	1.80216200	-6.39725200	-2.49020600	H	4.24521100	5.12500100	-2.49339000
C	-0.63242600	-6.63314000	-1.40360000	C	5.82343200	3.25297000	-1.40583600
C	-0.63242300	-6.63313800	1.40361100	C	5.82343000	3.25296900	1.40584700
H	3.99187900	-5.31457800	-2.49020400	H	2.10707800	6.31229900	-2.49338800
H	-0.63079300	-6.61599900	-2.49020400	H	5.80997800	3.24545000	-2.49338800
C	-1.83897200	-6.39805700	-0.72524400	C	6.31617600	2.12558900	-0.72571800
H	-0.63079100	-6.61599500	2.49021600	H	5.80997400	3.24544800	2.49340000
C	-1.83897300	-6.39805600	0.72525600	C	6.31617500	2.12558800	0.72573000
H	5.64270100	-3.51405800	2.49021100	H	-0.31553900	6.64712800	2.49339800
C	5.67590000	3.49315600	1.40360000	C	-6.10536700	2.68675300	1.40583900
H	5.66123400	3.48411700	2.49020400	H	-6.09125800	2.68053900	2.49339100
C	-2.98627500	-5.95679000	-1.40360000	C	6.60528200	0.92969400	-1.40583800
H	-2.97856100	-5.94139100	-2.49020300	H	6.59002000	0.92754500	-2.49339000
C	-2.98627300	-5.95678800	1.40361000	C	6.60528000	0.92969300	1.40584900
H	-2.97856000	-5.94138800	2.49021400	H	6.59001600	0.92754300	2.49340100
**[18]-cyclcacene**				**[18]-cyclcacene**			
C	-1.22477600	6.94650800	1.40356700	C	6.95321600	1.22600600	1.40566400
C	0.00011400	7.04778500	0.72527900	C	7.05463300	0.00000000	0.72578700
C	1.22500100	6.94646800	1.40356700	C	6.95321600	-1.22600600	1.40566400
C	0.00011400	7.04778500	-0.72527900	C	7.05463300	0.00000000	-0.72578700
C	1.22500100	6.94646800	-1.40356700	C	6.95321600	-1.22600600	-1.40566400
C	2.41066500	6.62271200	-0.72528000	C	6.62926100	-2.41277400	-0.72579600
C	2.41066500	6.62271200	0.72528000	C	6.62926100	-2.41277400	0.72579600
C	-2.41045100	6.62279000	0.72528000	C	6.62926100	2.41277400	0.72579600
C	-1.22477600	6.94650800	-1.40356700	C	6.95321600	1.22600600	-1.40566400
H	1.22217500	6.93044700	-2.49019000	H	6.93887000	-1.22347400	-2.49329500
C	3.52703400	6.10856900	-1.40356600	C	6.11458100	-3.53013300	-1.40566400
C	3.52703400	6.10856900	1.40356600	C	6.11458100	-3.53013300	1.40566400
H	3.51891700	6.09451400	-2.49019000	H	6.10196700	-3.52284900	-2.49329600
H	3.51891700	6.09451400	2.49019000	H	6.10196700	-3.52284900	2.49329600
C	-2.41045100	6.62279000	-0.72528000	C	6.62926100	2.41277400	-0.72579600
H	-1.22195100	6.93048600	2.49019000	H	6.93887000	1.22347400	2.49329500
H	1.22217500	6.93044700	2.49019000	H	6.93887000	-1.22347400	2.49329500
H	-1.22195100	6.93048600	-2.49019000	H	6.93887000	1.22347400	-2.49329500
C	4.53046600	5.39882400	0.72527800	C	5.40418000	-4.53446600	0.72579400
C	4.53046600	5.39882400	-0.72527800	C	5.40418000	-4.53446600	-0.72579400
C	-3.52683600	6.10868300	-1.40356600	C	6.11458100	3.53013300	-1.40566400
C	-4.53029100	5.39897000	-0.72527800	C	5.40418000	4.53446600	-0.72579400
C	-5.40357500	4.53411900	-1.40356900	C	4.53837200	5.40837800	-1.40566400
C	-6.10386500	3.52405900	-0.72528400	C	3.52731200	6.10918200	-0.72580400
C	-6.62867000	2.41266300	-1.40356900	C	2.41477300	6.63425100	-1.40565600
C	-6.94115900	1.22398000	-0.72528200	C	1.22497600	6.94696600	-0.72578700
C	-6.94115900	1.22398000	0.72528200	C	1.22497600	6.94696600	0.72578700

C	-7.05413500	0.00011400	1.40357000	C	0.00000000	7.05991400	1.40566200
C	-6.62867000	2.41266300	1.40356900	C	2.41477300	6.63425100	1.40565600
C	-6.10386500	3.52405900	0.72528400	C	3.52731200	6.10918200	0.72580400
C	-7.05413500	0.00011400	-1.40357000	C	0.00000000	7.05991400	-1.40566200
C	-5.40357500	4.53411900	1.40356900	C	4.53837200	5.40837800	1.40566400
H	-7.03788500	0.00011400	2.49019400	H	0.00000000	7.04533800	2.49329300
H	-7.03788500	0.00011400	-2.49019400	H	0.00000000	7.04533800	-2.49329300
C	-4.53029100	5.39897000	0.72527800	C	5.40418000	4.53446600	0.72579400
C	-3.52683600	6.10868300	1.40356600	C	6.11458100	3.53013300	1.40566400
H	-6.61340800	2.40710400	-2.49019400	H	2.40978200	6.62056000	-2.49328700
H	-6.61340800	2.40710400	2.49019400	H	2.40978200	6.62056000	2.49328700
H	-5.39111200	4.52367000	2.49019300	H	4.52900500	5.39720300	2.49329600
H	-5.39111200	4.52367000	-2.49019300	H	4.52900500	5.39720300	-2.49329600
H	-3.51872000	6.09462800	-2.49019000	H	6.10196700	3.52284900	-2.49329600
H	-3.51872000	6.09462800	2.49019000	H	6.10196700	3.52284900	2.49329600
C	5.40372100	4.53394500	1.40356900	C	4.53837200	-5.40837800	1.40566400
C	5.40372100	4.53394500	-1.40356900	C	4.53837200	-5.40837800	-1.40566400
H	5.39125800	4.52349600	2.49019300	H	4.52900500	-5.39720300	2.49329600
H	5.39125800	4.52349600	-2.49019300	H	4.52900500	-5.39720300	-2.49329600
C	-6.94119900	-1.22375600	-0.72528200	C	-1.22497600	6.94696600	-0.72578700
C	-6.62874800	-2.41244900	-1.40356900	C	-2.41477300	6.63425100	-1.40565600
C	-6.94119900	-1.22375600	0.72528200	C	-1.22497600	6.94696600	0.72578700
C	-6.62874800	-2.41244900	1.40356900	C	-2.41477300	6.63425100	1.40565600
H	-6.61348600	-2.40689100	-2.49019400	H	-2.40978200	6.62056000	-2.49328700
H	-6.61348600	-2.40689100	2.49019400	H	-2.40978200	6.62056000	2.49328700
C	6.10397900	3.52386100	-0.72528400	C	3.52731200	-6.10918200	-0.72580400
C	6.62874800	2.41244900	-1.40356900	C	2.41477300	-6.63425100	-1.40565600
C	6.94119900	1.22375600	-0.72528200	C	1.22497600	-6.94696600	-0.72578700
C	7.05413500	-0.00011400	-1.40357000	C	0.00000000	-7.05991400	-1.40566200
C	6.94115900	-1.22398000	-0.72528200	C	-1.22497600	-6.94696600	-0.72578700
C	6.94115900	-1.22398000	0.72528200	C	-1.22497600	-6.94696600	0.72578700
C	7.05413500	-0.00011400	1.40357000	C	0.00000000	-7.05991400	1.40566200
C	6.62867000	-2.41266300	-1.40356900	C	-2.41477300	-6.63425100	-1.40565600
C	6.62867000	-2.41266300	1.40356900	C	-2.41477300	-6.63425100	1.40565600
C	6.10386500	-3.52405900	0.72528400	C	-3.52731200	-6.10918200	0.72580400
C	6.10386500	-3.52405900	-0.72528400	C	-3.52731200	-6.10918200	-0.72580400
C	5.40357500	-4.53411900	-1.40356900	C	-4.53837200	-5.40837800	-1.40566400
C	4.53029100	-5.39897000	-0.72527800	C	-5.40418000	-4.53446600	-0.72579400
C	3.52683600	-6.10868300	-1.40356600	C	-6.11458100	-3.53013300	-1.40566400
C	2.41045100	-6.62279000	-0.72528000	C	-6.62926100	-2.41277400	-0.72579600
C	2.41045100	-6.62279000	0.72528000	C	-6.62926100	-2.41277400	0.72579600
C	3.52683600	-6.10868300	1.40356600	C	-6.11458100	-3.53013300	1.40566400
C	1.22477600	-6.94650800	1.40356700	C	-6.95321600	-1.22600600	1.40566400
C	1.22477600	-6.94650800	-1.40356700	C	-6.95321600	-1.22600600	-1.40566400
C	-0.00011400	-7.04778500	-0.72527900	C	-7.05463300	0.00000000	-0.72578700
C	-0.00011400	-7.04778500	0.72527900	C	-7.05463300	0.00000000	0.72578700
C	4.53029100	-5.39897000	0.72527800	C	-5.40418000	-4.53446600	0.72579400
H	1.22195100	-6.93048600	-2.49019000	H	-6.93887000	-1.22347400	-2.49329500
C	-1.22500100	-6.94646800	-1.40356700	C	-6.95321600	1.22600600	-1.40566400
C	-1.22500100	-6.94646800	1.40356700	C	-6.95321600	1.22600600	1.40566400
H	-1.22217500	-6.93044700	-2.49019000	H	-6.93887000	1.22347400	-2.49329500
H	-1.22217500	-6.93044700	2.49019000	H	-6.93887000	1.22347400	2.49329500
H	3.51872000	-6.09462800	2.49019000	H	-6.10196700	-3.52284900	2.49329600
H	1.22195100	-6.93048600	2.49019000	H	-6.93887000	-1.22347400	2.49329500
H	3.51872000	-6.09462800	-2.49019000	H	-6.10196700	-3.52284900	-2.49329600
C	5.40357500	-4.53411900	1.40356900	C	-4.53837200	-5.40837800	1.40566400
H	7.03788500	-0.00011400	-2.49019400	H	0.00000000	-7.04533800	-2.49329300
H	7.03788500	-0.00011400	2.49019400	H	0.00000000	-7.04533800	2.49329300
H	6.61340800	-2.40710400	2.49019400	H	-2.40978200	-6.62056000	2.49328700
H	6.61340800	-2.40710400	-2.49019400	H	-2.40978200	-6.62056000	-2.49328700

H	5.39111200	-4.52367000	-2.49019300	H	-4.52900500	-5.39720300	-2.49329600
H	5.39111200	-4.52367000	2.49019300	H	-4.52900500	-5.39720300	2.49329600
C	6.10397900	3.52386100	0.72528400	C	3.52731200	-6.10918200	0.72580400
C	6.62874800	2.41244900	1.40356900	C	2.41477300	-6.63425100	1.40565600
C	6.94119900	1.22375600	0.72528200	C	1.22497600	-6.94696600	0.72578700
H	6.61348600	2.40689100	-2.49019400	H	2.40978200	-6.62056000	-2.49328700
H	6.61348600	2.40689100	2.49019400	H	2.40978200	-6.62056000	2.49328700
C	-2.41066500	-6.62271200	-0.72528000	C	-6.62926100	2.41277400	-0.72579600
C	-3.52703400	-6.10856900	-1.40356600	C	-6.11458100	3.53013300	-1.40566400
C	-2.41066500	-6.62271200	0.72528000	C	-6.62926100	2.41277400	0.72579600
C	-3.52703400	-6.10856900	1.40356600	C	-6.11458100	3.53013300	1.40566400
H	-3.51891700	-6.09451400	-2.49019000	H	-6.10196700	3.52284900	-2.49329600
H	-3.51891700	-6.09451400	2.49019000	H	-6.10196700	3.52284900	2.49329600
C	-4.53046600	-5.39882400	-0.72527800	C	-5.40418000	4.53446600	-0.72579400
C	-5.40372100	-4.53394500	-1.40356900	C	-4.53837200	5.40837800	-1.40566400
C	-6.10397900	-3.52386100	-0.72528400	C	-3.52731200	6.10918200	-0.72580400
C	-6.10397900	-3.52386100	0.72528400	C	-3.52731200	6.10918200	0.72580400
C	-5.40372100	-4.53394500	1.40356900	C	-4.53837200	5.40837800	1.40566400
C	-4.53046600	-5.39882400	0.72527800	C	-5.40418000	4.53446600	0.72579400
H	-5.39125800	-4.52349600	-2.49019300	H	-4.52900500	5.39720300	-2.49329600
H	-5.39125800	-4.52349600	2.49019300	H	-4.52900500	5.39720300	2.49329600
**[19]-cyclcacene**				**[19]-cyclcacene**			
C	0.62840500	7.41777600	1.40355600	C	0.61529300	7.42721100	1.40565100
C	-0.60050600	7.41449100	0.72532300	C	-0.61473500	7.42163200	0.72582600
C	-1.81392000	7.21994400	1.40355700	C	-1.82886300	7.22441300	1.40565200
C	-0.60050600	7.41449100	-0.72532300	C	-0.61473500	7.42163200	-0.72582600
C	-1.81392000	7.21994400	-1.40355700	C	-1.82886300	7.22441300	-1.40565200
C	-2.97513400	6.81769700	-0.72532200	C	-2.99033600	6.81945400	-0.72584300
C	-2.97513400	6.81769700	0.72532200	C	-2.99033600	6.81945400	0.72584300
C	1.83922000	7.20764800	0.72532200	C	1.82760800	7.21918600	0.72583300
C	0.62840500	7.41777600	-1.40355600	C	0.61529300	7.42721100	-1.40565100
H	-1.81017600	7.20503700	-2.49019900	H	-1.82546700	7.21104000	-2.49330100
C	-4.05959000	6.23961500	-1.40355200	C	-4.07451000	6.23844700	-1.40566200
C	-4.05959000	6.23961500	1.40355200	C	-4.07451000	6.23844700	1.40566200
H	-4.05121000	6.22675000	-2.49019500	H	-4.06698100	6.22693300	-2.49331200
H	-4.05121000	6.22675000	2.49019500	H	-4.06698100	6.22693300	2.49331200
C	1.83922000	7.20764800	-0.72532200	C	1.82760800	7.21918600	-0.72583300
H	0.62710900	7.40247600	2.49019800	H	0.61415000	7.41345600	2.49330000
H	-1.81017600	7.20503700	2.49019900	H	-1.82546700	7.21104000	2.49330100
H	0.62710900	7.40247600	-2.49019800	H	0.61415000	7.41345600	-2.49330000
C	-5.02721900	5.48202600	0.72532400	C	-5.04141800	5.47813300	0.72584200
C	-5.02721900	5.48202600	-0.72532400	C	-5.04141800	5.47813300	-0.72584200
C	3.00257200	6.81163100	-1.40355700	C	2.99271300	6.82479100	-1.40564700
C	4.07951400	6.21966700	-0.72532500	C	4.07169700	6.23414300	-0.72584200
C	5.05118800	5.46727500	-1.40355900	C	5.04538800	5.48252400	-1.40564200
C	5.87751600	4.55767300	-0.72532000	C	5.87389700	4.57336700	-0.72583500
C	6.55232500	3.53061700	-1.40355400	C	6.55073200	3.54628700	-1.40565200
C	7.03870600	2.40205300	-0.72532300	C	7.03928400	2.41742300	-0.72583900
C	7.03870600	2.40205300	0.72532300	C	7.03928400	2.41742300	0.72583900
C	7.34354900	1.21155000	1.40355400	C	7.34621400	1.22629400	1.40565100
C	6.55232500	3.53061700	1.40355400	C	6.55073200	3.54628700	1.40565200
C	5.87751600	4.55767300	0.72532000	C	5.87389700	4.57336700	0.72583500
C	7.34354900	1.21155000	-1.40355400	C	7.34621400	1.22629400	-1.40565100
C	5.05118800	5.46727500	1.40355900	C	5.04538800	5.48252400	1.40564200
H	7.32836200	1.20904500	2.49019500	H	7.33264100	1.22402900	2.49330000
H	7.32836200	1.20904500	-2.49019500	H	7.33264100	1.22402900	-2.49330000
C	4.07951400	6.21966700	0.72532500	C	4.07169700	6.23414300	0.72584200
C	3.00257200	6.81163100	1.40355700	C	2.99271300	6.82479100	1.40564700

H	6.53879100	3.52332800	-2.49019600	H	6.53858800	3.53972300	-2.49330200
H	6.53879100	3.52332800	2.49019600	H	6.53858800	3.53972300	2.49330200
H	5.04077000	5.45599600	2.49020000	H	5.03603100	5.47236500	2.49329200
H	5.04077000	5.45599600	-2.49020000	H	5.03603100	5.47236500	-2.49329200
H	2.99636600	6.79756500	-2.49019800	H	2.98714800	6.81212800	-2.49329600
H	2.99636600	6.79756500	2.49019800	H	2.98714800	6.81212800	2.49329600
C	-5.86511100	4.58305000	1.40355500	C	-5.87815500	4.57655000	1.40566200
C	-5.86511100	4.58305000	-1.40355500	C	-5.87815500	4.57655000	-1.40566200
H	-5.85297600	4.57357500	2.49019700	H	-5.86727200	4.56809100	2.49331100
H	-5.85297600	4.57357500	-2.49019700	H	-5.86727200	4.56809100	-2.49331100
C	-6.53427700	3.55230200	-0.72532400	C	-6.54591200	3.54354800	-0.72584700
C	-7.03492300	2.42999400	-1.40355500	C	-7.04475200	2.41917200	-1.40564500
C	-7.33323800	1.23783900	-0.72532500	C	-7.34114000	1.22536000	-0.72583000
C	-7.44250200	0.01379600	-1.40355600	C	-7.44812200	0.00000100	-1.40564800
C	-7.33777400	-1.21064200	-0.72532500	C	-7.34114000	-1.22535700	-0.72583000
C	-7.33777400	-1.21064200	0.72532500	C	-7.34114000	-1.22535700	0.72583000
C	-7.44250200	0.01379600	1.40355600	C	-7.44812200	0.00000100	1.40564800
C	-7.04388000	-2.40389500	-1.40355500	C	-7.04475200	-2.41916900	-1.40564500
C	-7.04388000	-2.40389500	1.40355500	C	-7.04475200	-2.41916900	1.40564500
C	-6.54739600	-3.52805100	0.72532400	C	-6.54591300	-3.54354600	0.72584700
C	-6.54739600	-3.52805100	-0.72532400	C	-6.54591300	-3.54354600	-0.72584700
C	-5.88205500	-4.56127100	-1.40355500	C	-5.87815500	-4.57654800	-1.40566200
C	-5.04750100	-5.46334700	-0.72532400	C	-5.04141900	-5.47813100	-0.72584200
C	-4.08268700	-6.22451900	-1.40355200	C	-4.07451100	-6.23844500	-1.40566200
C	-3.00038200	-6.80661800	-0.72532200	C	-2.99033700	-6.81945300	-0.72584300
C	-3.00038200	-6.80661800	0.72532200	C	-2.99033700	-6.81945300	0.72584300
C	-4.08268700	-6.22451900	1.40355200	C	-4.07451100	-6.23844500	1.40566200
C	-1.84066800	-7.21316800	1.40355700	C	-1.82886400	-7.22441200	1.40565200
C	-1.84066800	-7.21316800	-1.40355700	C	-1.82886400	-7.22441200	-1.40565200
C	-0.62798400	-7.41221500	-0.72532300	C	-0.61473600	-7.42163100	-0.72582600
C	-0.62798400	-7.41221500	0.72532300	C	-0.61473600	-7.42163100	0.72582600
C	-5.04750100	-5.46334700	0.72532400	C	-5.04141900	-5.47813100	0.72584200
H	-1.83686900	-7.19827600	-2.49019900	H	-1.82546900	-7.21103900	-2.49330100
C	0.60090600	-7.42005600	-1.40355600	C	0.61529200	-7.42721100	-1.40565100
C	0.60090600	-7.42005600	1.40355600	C	0.61529200	-7.42721100	1.40565100
H	0.59966700	-7.40475200	-2.49019800	H	0.61414800	-7.41345600	-2.49330000
H	0.59966700	-7.40475200	2.49019800	H	0.61414800	-7.41345600	2.49330000
H	-4.07426000	-6.21168500	2.49019500	H	-4.06698200	-6.22693100	2.49331200
H	-1.83686900	-7.19827600	2.49019900	H	-1.82546900	-7.21103900	2.49330100
H	-4.07426000	-6.21168500	-2.49019500	H	-4.06698200	-6.22693100	-2.49331200
C	-5.88205500	-4.56127100	1.40355500	C	-5.87815500	-4.57654800	1.40566200
H	-7.42712700	0.01376800	-2.49019800	H	-7.43431600	0.00000100	-2.49329700
H	-7.42712700	0.01376800	2.49019800	H	-7.43431600	0.00000100	2.49329700
H	-7.02934800	-2.39893800	2.49019800	H	-7.03171500	-2.41468900	2.49329400
H	-7.02934800	-2.39893800	-2.49019800	H	-7.03171500	-2.41468900	-2.49329400
H	-5.86988500	-4.55184200	-2.49019700	H	-5.86727300	-4.56808900	-2.49331100
H	-5.86988500	-4.55184200	2.49019700	H	-5.86727300	-4.56808900	2.49331100
C	-6.53427700	3.55230200	0.72532400	C	-6.54591200	3.54354800	0.72584700
C	-7.03492300	2.42999400	1.40355500	C	-7.04475200	2.41917200	1.40564500
C	-7.33323800	1.23783900	0.72532500	C	-7.34114000	1.22536000	0.72583000
H	-7.02040900	2.42498300	-2.49019800	H	-7.03171500	2.41469100	-2.49329400
H	-7.02040900	2.42498300	2.49019800	H	-7.03171500	2.41469100	2.49329400
C	1.81249200	-7.21442000	-0.72532200	C	1.82760600	-7.21918700	-0.72583300
C	2.97730400	-6.82271800	-1.40355700	C	2.99271200	-6.82479200	-1.40564700
C	1.81249200	-7.21442000	0.72532200	C	1.82760600	-7.21918700	0.72583300
C	2.97730400	-6.82271800	1.40355700	C	2.99271200	-6.82479200	1.40564700
H	2.97115100	-6.80862900	-2.49019800	H	2.98714700	-6.81212900	-2.49329600
H	2.97115100	-6.80862900	2.49019800	H	2.98714700	-6.81212900	2.49329600
C	4.05643300	-6.23475100	-0.72532500	C	4.07169600	-6.23414500	-0.72584200
C	5.03089000	-5.48596700	-1.40355900	C	5.04538700	-5.48252700	-1.40564200

C	5.86058400	-4.57943400	-0.72532000	C	5.87389700	-4.57336900	-0.72583500
C	6.53919500	-3.55488600	-1.40355400	C	6.55073100	-3.54628900	-1.40565200
H	6.52568800	-3.54754600	-2.49019600	H	6.53858700	-3.53972500	-2.49330200
C	5.86058400	-4.57943400	0.72532000	C	5.87389700	-4.57336900	0.72583500
C	6.53919500	-3.55488600	1.40355400	C	6.55073100	-3.54628900	1.40565200
H	6.52568800	-3.54754600	2.49019600	H	6.53858700	-3.53972500	2.49330200
C	5.03089000	-5.48596700	1.40355900	C	5.04538700	-5.48252700	1.40564200
C	4.05643300	-6.23475100	0.72532500	C	4.07169600	-6.23414500	0.72584200
H	5.02051500	-5.47464900	-2.49020000	H	5.03603100	-5.47236800	-2.49329200
H	5.02051500	-5.47464900	2.49020000	H	5.03603100	-5.47236800	2.49329200
C	7.02975600	-2.42813200	-0.72532300	C	7.03928400	-2.41742600	-0.72583800
C	7.33900900	-1.23876800	-1.40355400	C	7.34621300	-1.22629600	-1.40565100
C	7.43718600	-0.01378600	-0.72532100	C	7.44214000	-0.00000100	-0.72582900
C	7.43718600	-0.01378600	0.72532100	C	7.44214000	-0.00000100	0.72582900
C	7.33900900	-1.23876800	1.40355400	C	7.34621300	-1.22629600	1.40565100
C	7.02975600	-2.42813200	0.72532300	C	7.03928400	-2.41742600	0.72583800
H	7.32383100	-1.23620600	-2.49019500	H	7.33264100	-1.22403100	-2.49330000
H	7.32383100	-1.23620600	2.49019500	H	7.33264100	-1.22403100	2.49330000
**[20]-cyclcacene**				**[20]-cyclcacene**			
C	7.70914200	-1.38934900	-1.40352300	C	7.67857000	1.58457100	-1.40562700
C	7.05345100	3.40734500	-1.40352100	C	5.28093500	5.79560200	-1.40562100
C	7.05345000	3.40734500	1.40352300	C	5.28093500	5.79560200	1.40562200
C	6.42921500	4.46576200	0.72534600	C	4.30642900	6.54599900	0.72585800
C	6.42921400	4.46576300	-0.72534400	C	4.30642900	6.54599900	-0.72585600
C	7.39186000	-2.57646500	-0.72534300	C	7.82669600	0.36360400	-0.72585700
C	7.70914100	-1.38934900	1.40352200	C	7.67856900	1.58457100	1.40562600
H	7.04033100	3.40100400	2.49017900	H	5.27209200	5.78589000	2.49328200
C	5.65536400	5.42026600	1.40352400	C	3.23157300	7.14382600	1.40561500
C	5.65536400	5.42026500	-1.40352200	C	3.23157400	7.14382600	-1.40561300
H	5.64483000	5.41017500	2.49018100	H	3.22616700	7.13188900	2.49327500
H	5.64483000	5.41017600	-2.49017800	H	3.22616700	7.13189000	-2.49327300
C	7.39186000	-2.57646600	0.72534100	C	7.82669500	0.36360400	0.72585500
H	7.69479600	-1.38676700	-2.49017900	H	7.66579000	1.58193600	-2.49328700
H	7.04033200	3.40100500	-2.49017800	H	5.27209200	5.78589100	-2.49328100
H	7.69479400	-1.38676800	2.49017800	H	7.66578900	1.58193400	2.49328600
C	4.73460200	6.23396400	-0.72534200	C	2.07283600	7.55616900	-0.72584700
C	4.73460300	6.23396400	0.72534400	C	2.07283700	7.55616900	0.72584900
C	1.06091500	-7.76109700	-1.40352400	C	3.87992700	-6.81317600	-1.40562700
C	2.26042000	-7.49444200	-0.72534800	C	4.89474900	-6.11830500	-0.72585300
C	3.40734400	-7.05344900	-1.40352500	C	5.79560100	-5.28093500	-1.40562400
C	2.26042000	-7.49444300	0.72534300	C	4.89474900	-6.11830600	0.72584800
C	3.40734500	-7.05345100	1.40352000	C	5.79560300	-5.28093600	1.40561900
C	4.46576500	-6.42921400	0.72534300	C	6.54600100	-4.30642800	0.72585400
C	4.46576500	-6.42921300	-0.72534700	C	6.54600100	-4.30642700	-0.72585900
C	1.06091600	-7.76109900	1.40351900	C	3.87992900	-6.81317700	1.40562200
C	5.42026600	-5.65536500	1.40352100	C	7.14382700	-3.23157500	1.40561200
C	5.42026600	-5.65536400	-1.40352500	C	7.14382700	-3.23157400	-1.40561600
H	1.05894100	-7.74664500	-2.49018000	H	3.87345500	-6.80181400	-2.49328800
H	1.05894200	-7.74665000	2.49017500	H	3.87345800	-6.80181700	2.49328300
C	6.23396700	-4.73460200	-0.72534400	C	7.55617100	-2.07283600	-0.72584900
C	6.23396600	-4.73460300	0.72534100	C	7.55617100	-2.07283600	0.72584600
C	6.90258900	-3.70364200	1.40351700	C	7.79254900	-0.86584800	1.40562700
C	6.90258900	-3.70364200	-1.40351900	C	7.79254900	-0.86584800	-1.40563000
H	3.40100800	-7.04033200	2.49017600	H	5.78589300	-5.27209200	2.49327900
H	3.40100600	-7.04032900	-2.49018100	H	5.78589100	-5.27209000	-2.49328400
H	5.41017700	-5.64482900	-2.49018100	H	7.13189000	-3.22616600	-2.49327600
H	5.41017700	-5.64483000	2.49017800	H	7.13189100	-3.22616800	2.49327200
H	6.88976800	-3.69676100	2.49017400	H	7.77956400	-0.86441200	2.49328700

H	6.88976900	-3.69676000	-2.49017700	H	7.77956400	-0.86441100	-2.49329000
C	-6.90258800	3.70364300	-1.40351600	C	-7.79254800	0.86584900	-1.40562700
C	-7.39185900	2.57646400	-0.72534100	C	-7.82669400	-0.36360500	-0.72585500
C	-7.70914100	1.38935000	-1.40352100	C	-7.67857000	-1.58457000	-1.40562500
C	-7.39185900	2.57646400	0.72534400	C	-7.82669400	-0.36360500	0.72585800
C	-7.70914000	1.38934900	1.40352400	C	-7.67856900	-1.58457100	1.40562700
C	-7.82616600	0.16614700	0.72534600	C	-7.33120700	-2.76442300	0.72584400
C	-7.82616600	0.16614700	-0.72534400	C	-7.33120800	-2.76442300	-0.72584200
C	-6.23396500	4.73460200	-0.72534200	C	-7.55616900	2.07283600	-0.72584600
C	-6.90258800	3.70364100	1.40352000	C	-7.79254800	0.86584800	1.40563000
H	-7.69479400	1.38676500	2.49018000	H	-7.66578800	-1.58193700	2.49328800
C	-7.76109900	-1.06091600	1.40352200	C	-6.81317600	-3.87992800	1.40562500
C	-7.76110000	-1.06091400	-1.40352100	C	-6.81317800	-3.87992700	-1.40562400
H	-7.74664700	-1.05894300	2.49017800	H	-6.80181400	-3.87345700	2.49328600
H	-7.74665000	-1.05894100	-2.49017700	H	-6.80181600	-3.87345700	-2.49328500
C	-6.23396500	4.73460200	0.72534500	C	-7.55616900	2.07283600	0.72585000
H	-6.88976800	3.69676000	-2.49017400	H	-7.77956400	0.86441200	-2.49328700
H	-7.69479500	1.38676700	-2.49017700	H	-7.66579000	-1.58193600	-2.49328500
H	-6.88976700	3.69675900	2.49017700	H	-7.77956300	0.86441100	2.49329000
C	3.70364200	6.90258800	-1.40351700	C	0.86584800	7.79254800	-1.40562700
C	-1.06091500	7.76110000	-1.40351900	C	-3.87992800	6.81317800	-1.40562300
C	-1.06091600	7.76110000	1.40352300	C	-3.87992900	6.81317700	1.40562700
C	-2.26042000	7.49444500	0.72534600	C	-4.89475000	6.11830800	0.72585200
C	-2.26042000	7.49444500	-0.72534300	C	-4.89475000	6.11830800	-0.72584800
C	3.70364100	6.90258800	1.40352000	C	0.86584700	7.79254800	1.40563000
C	-3.40734500	7.05345000	1.40352400	C	-5.79560200	5.28093600	1.40562300
C	-3.40734500	7.05345100	-1.40352000	C	-5.79560200	5.28093600	-1.40562000
H	3.69676000	6.88976700	-2.49017400	H	0.86441100	7.77956400	-2.49328700
H	3.69676000	6.88976700	2.49017700	H	0.86441200	7.77956400	2.49329000
C	-4.46576400	6.42921400	-0.72534400	C	-6.54600000	4.30642900	-0.72585500
C	-4.46576400	6.42921400	0.72534700	C	-6.54600000	4.30642900	0.72585900
C	-5.42026600	5.65536400	1.40352500	C	-7.14382600	3.23157400	1.40561600
C	-5.42026500	5.65536500	-1.40352200	C	-7.14382700	3.23157500	-1.40561300
H	-1.05894200	7.74664900	2.49017900	H	-3.87345700	6.80181600	2.49328700
H	-1.05894200	7.74664900	-2.49017600	H	-3.87345700	6.80181600	-2.49328400
H	-3.40100600	7.04033200	-2.49017700	H	-5.78589200	5.27209300	-2.49328000
H	-3.40100600	7.04033100	2.49018000	H	-5.78589200	5.27209200	2.49328300
H	-5.41017600	5.64482900	2.49018100	H	-7.13189000	3.22616700	2.49327600
H	-5.41017600	5.64483000	-2.49017800	H	-7.13189000	3.22616700	-2.49327300
C	-3.70364100	-6.90258900	1.40351600	C	-0.86584700	-7.79254900	1.40562700
C	-3.70364300	-6.90258700	-1.40352000	C	-0.86584900	-7.79254800	-1.40563000
H	-3.69676000	-6.88976800	2.49017400	H	-0.86441100	-7.77956400	2.49328700
H	-3.69676100	-6.88976600	-2.49017700	H	-0.86441300	-7.77956300	-2.49329000
C	0.16614800	7.82616600	0.72534700	C	-2.76442200	7.33120800	0.72584500
C	1.38934900	7.70914100	1.40352400	C	-1.58457200	7.67857000	1.40562800
C	2.57646500	7.39185800	0.72534400	C	-0.36360400	7.82669500	0.72585800
C	2.57646500	7.39185800	-0.72534100	C	-0.36360400	7.82669500	-0.72585500
C	1.38934900	7.70914000	-1.40352100	C	-1.58457100	7.67857000	-1.40562400
C	0.16614700	7.82616600	-0.72534400	C	-2.76442300	7.33120800	-0.72584100
H	1.38676700	7.69479500	2.49018000	H	-1.58193600	7.66579000	2.49328800
H	1.38676700	7.69479500	-2.49017700	H	-1.58193600	7.66579000	-2.49328500
C	-7.49444500	-2.26042100	0.72534400	C	-6.11830700	-4.89475000	0.72585000
C	-7.05345000	-3.40734600	1.40352200	C	-5.28093500	-5.79560200	1.40562100
C	-7.05345200	-3.40734400	-1.40352200	C	-5.28093700	-5.79560200	-1.40562200
C	-7.49444500	-2.26042100	-0.72534400	C	-6.11830700	-4.89475000	-0.72585000
H	-7.04033000	-3.40100700	2.49017800	H	-5.27209000	-5.78589200	2.49328100
H	-7.04033300	-3.40100500	-2.49017900	H	-5.27209300	-5.78589100	-2.49328200
C	-6.42921500	-4.46576400	0.72534400	C	-4.30642900	-6.54600000	0.72585600
C	-5.65536400	-5.42026700	1.40352200	C	-3.23157300	-7.14382700	1.40561300
C	-4.73460400	-6.23396500	0.72534100	C	-2.07283700	-7.55617000	0.72584600

C	-4.73460400	-6.23396500	-0.72534400	C	-2.07283700	-7.55617000	-0.72584900
C	-5.65536600	-5.42026500	-1.40352400	C	-3.23157500	-7.14382600	-1.40561500
C	-6.42921500	-4.46576500	-0.72534600	C	-4.30642900	-6.54600100	-0.72585700
H	-5.64482900	-5.41017700	2.49017800	H	-3.22616600	-7.13189000	2.49327300
H	-5.64483100	-5.41017600	-2.49018000	H	-3.22616800	-7.13189000	-2.49327500
C	-2.57646600	-7.39185800	0.72534000	C	0.36360300	-7.82669500	0.72585400
C	-1.38934800	-7.70914100	1.40352000	C	1.58457300	-7.67857000	1.40562400
C	-0.16614900	-7.82616400	0.72534400	C	2.76442100	-7.33120800	0.72584100
C	-0.16614800	-7.82616300	-0.72534900	C	2.76442100	-7.33120700	-0.72584600
C	-1.38934900	-7.70913800	-1.40352500	C	1.58457100	-7.67856900	-1.40562900
C	-2.57646600	-7.39185800	-0.72534500	C	0.36360300	-7.82669400	-0.72585800
H	-1.38676800	-7.69479500	2.49017600	H	1.58193600	-7.66579100	2.49328400
H	-1.38676900	-7.69479200	-2.49018100	H	1.58193400	-7.66578800	-2.49328900
C	7.49444500	2.26041800	0.72534500	C	6.11830800	4.89474800	0.72585000
C	7.76109900	1.06091600	1.40352100	C	6.81317600	3.87992800	1.40562500
C	7.82616600	-0.16614900	0.72534500	C	7.33120800	2.76442100	0.72584200
C	7.82616700	-0.16614800	-0.72534500	C	7.33120900	2.76442200	-0.72584300
C	7.76110000	1.06091600	-1.40352100	C	6.81317700	3.87992800	-1.40562500
C	7.49444500	2.26041900	-0.72534400	C	6.11830800	4.89474900	-0.72584900
H	7.74664800	1.05894000	2.49017800	H	6.80181500	3.87345500	2.49328600
H	7.74665000	1.05894100	-2.49017700	H	6.80181600	3.87345700	-2.49328500

Table S10. Calculated Gibbs free energies (ΔG, 298.15 K) of [*n*]-cyclacenes ($6 \leq n \leq 20$), the corresponding tetrahydro-[*n*]-cyclacenes, and the Gibbs free energy changes for their hydrogenation (ΔG_{hyd}, kcal/mol), computed at the UB3LYP-D3(BJ)/6-31G(d) level of theory.

n	**G (Hartree)**	**G (Hydrogenated) (Hartree)**	**ΔG_{hyd} (kcal/mol)**
6	-921.3539	-923.79043	-51.8
7	-1075.0012	-1077.429	-46.3
8	-1228.6592	-1231.0621	-30.7
9	-1382.2897	-1384.6898	-29.0
10	-1535.9284	-1538.3149	-20.4
11	-1689.5553	-1691.9373	-17.6
12	-1843.1817	-1845.5583	-14.2
13	-1996.8074	-1999.1778	-10.3
14	-2150.4270	-2152.7964	-9.7
15	-2304.0512	-2306.414	-5.6
16	-2457.6671	-2460.0309	-6.2
17	-2611.2897	-2613.6472	-2.2
18	-2764.9038	-2767.2629	-3.2
19	-2918.5248	-2920.8783	0.3
20	-3072.1413	-3074.4933	1.2
**H_2 (Hartree)** -1.176976			
